# Chromosomal genome of *Triplophysa bleekeri* provides insights into its evolution and environmental adaptation

**DOI:** 10.1093/gigascience/giaa132

**Published:** 2020-11-24

**Authors:** Dengyue Yuan, Xuehui Chen, Haoran Gu, Ming Zou, Yu Zou, Jian Fang, Wenjing Tao, Xiangyan Dai, Shijun Xiao, Zhijian Wang

**Affiliations:** Key Laboratory of Freshwater Fish Reproduction and Development (Ministry of Education), Key Laboratory of Aquatic Science of Chongqing, School of Life Sciences, Southwest University, Chongqing 400715, China; Key Laboratory of Freshwater Fish Reproduction and Development (Ministry of Education), Key Laboratory of Aquatic Science of Chongqing, School of Life Sciences, Southwest University, Chongqing 400715, China; Key Laboratory of Freshwater Fish Reproduction and Development (Ministry of Education), Key Laboratory of Aquatic Science of Chongqing, School of Life Sciences, Southwest University, Chongqing 400715, China; School of Computer Science and Technology, Wuhan University of Technology, Wuhan, Hubei 430000, China; School of Computer Science and Technology, Wuhan University of Technology, Wuhan, Hubei 430000, China; School of Computer Science and Technology, Wuhan University of Technology, Wuhan, Hubei 430000, China; Key Laboratory of Freshwater Fish Reproduction and Development (Ministry of Education), Key Laboratory of Aquatic Science of Chongqing, School of Life Sciences, Southwest University, Chongqing 400715, China; Key Laboratory of Freshwater Fish Reproduction and Development (Ministry of Education), Key Laboratory of Aquatic Science of Chongqing, School of Life Sciences, Southwest University, Chongqing 400715, China; School of Computer Science and Technology, Wuhan University of Technology, Wuhan, Hubei 430000, China; College of Plant Protection, Jilin Agriculture University, Changchun, Jilin 130118, China; Key Laboratory of Freshwater Fish Reproduction and Development (Ministry of Education), Key Laboratory of Aquatic Science of Chongqing, School of Life Sciences, Southwest University, Chongqing 400715, China

**Keywords:** Triplophysa bleekeri, genome, genetic adaptation, population genomics

## Abstract

**Background:**

Intense stresses caused by high-altitude environments may result in noticeable genetic adaptions in native species. Studies of genetic adaptations to high elevations have been largely limited to terrestrial animals. How fish adapt to high-elevation environments is largely unknown. *Triplophysa bleekeri*, an endemic fish inhabiting high-altitude regions, is an excellent model to investigate the genetic mechanisms of adaptation to the local environment. Here, we assembled a chromosomal genome sequence of *T. bleekeri*, with a size of ∼628 Mb (contig and scaffold N50 of 3.1 and 22.9 Mb, respectively). We investigated the origin and environmental adaptation of *T. bleekeri* based on 21,198 protein-coding genes in the genome.

**Results:**

Compared with fish species living at low altitudes, gene families associated with lipid metabolism and immune response were significantly expanded in the *T. bleekeri* genome. Genes involved in DNA repair exhibit positive selection for *T. bleekeri, Triplophysa siluroides*, and *Triplophysa tibetana*, indicating that adaptive convergence in *Triplophysa* species occurred at the positively selected genes. We also analyzed whole-genome variants among samples from 3 populations. The results showed that populations separated by geological and artificial barriers exhibited obvious differences in genetic structures, indicating that gene flow is restricted between populations.

**Conclusions:**

These results will help us expand our understanding of environmental adaptation and genetic diversity of *T. bleekeri* and provide valuable genetic resources for future studies on the evolution and conservation of high-altitude fish species such as *T. bleekeri*.

## Introduction

The Qinghai-Tibetan Plateau (QTP), the largest and highest plateau in the world, is one of the most important world biodiversity centers [1]. The environments of QTP and its peripheral areas have been affected significantly by the continuing uplift, which is one of the most important driving forces for the biological evolution of organisms on the plateau [2]. The endemic species of the QTP present high adaptability to the harsh environmental conditions, such as low temperature, low oxygen supply, and high UV radiation, by exhibiting cold tolerance, hypoxia resistance, enhanced metabolic capacity, and increased body mass [3–6].

An investigation into the biological evolution of organisms residing on the QTP and its peripheral regions will broaden our understanding of essential evolutionary questions regarding mechanisms of environmental adaptation and speciation. Phenotype comparisons were frequently used to study environmental adaptations in previous studies [7, 8]. In recent years, advancing genomic technology, especially third-generation sequencing techniques, has presented novel opportunities to explore the genetic basis of environmental adaptations. Many genomic studies of terrestrial animals on the QTP and its peripheral regions revealed that genes involved in hypoxia response, energy metabolism, and DNA repair were under positive selection and rapid evolution [9–11]. In those studies, high-quality genome and population resources are essential to understand critical biological processes for adaptations [11–13].

The QTP boasts many highland fish species, especially in the family Sisoridae, subfamily Schizothoracinae, and genus *Triplophysa* [14]. To date, there have only been several high-quality highland fish genomes reported on the basis of long-read sequencing data, including *Glyptosternon maculatum* in the family Sisoridae, *Schizothorax o'connori* and *Oxygymnocypris stewartii* in the subfamily Schizothoracinae, and *Triplophysa tibetana* and *Triplophysa siluroides* in the genus *Triplophysa* [15–19]. *Triplophysa* is a highly diverse genus and the largest group of the subfamily Nemacheilinae [20]. There are 152 records for *Triplophysa* species in FishBase, and the majority are distributed on the QTP and its adjacent drainage areas from an elevation of 100 to >5,200 m [21]. Given the broad elevation distributions and species diversity, the *Triplophysa* genus offers an attractive study model not only to investigate the adaptive mechanisms of fish in high altitudes but also to examine the similarities and differences between the adaptive mechanisms in different *Triplophysa* species. Previous studies have reported the genomic data of *T. siluroides* and *T. tibetana* without any emphasis on the genetic basis of high-altitude adaption [17, 18]. To date, environmental adaptations of *Triplophysa* species to high altitudes are not fully understood, and the genetic resources for the reference genome and population data remain insufficient. *Triplophysa bleekeri*, another member of the Nemacheilidae family, is mainly distributed in the stem streams and tributaries of the Yangtze and Jinsha rivers [22]. It exhibits different ecological and physiological characteristics compared with its relatives, *T. siluroides* and *T. tibetana* [23]. *T. bleekeri* has a wide distribution, from 200 to 3,000 m [24], whereas *T. tibetana* and *T. siluroides* occur at elevations of ∼4,000–5,000 and ∼3,000 –4,000 m, respectively [17, 25]. Apart from altitude of habitation, there is a significant difference in habitat environments. *T. bleekeri* lives in the fast-flowing rivers, whereas *T. tibetana* and *T. siluroides* inhabit lakes and slow-flowing rivers [14]. Reproduction biology in these *Triplophysa* species is also different; *T. tibetana* and *T. siluroides* spawn once a year (June–July and July–August, respectively), whereas *T. bleekeri* can spawn twice a year, with peak breeding seasons occurring from October to December and March to April [24]. The primary food source of *T. bleekeri* and *T. tibetana* is *Chironomus* larvae, caddisfly larvae, and diatoms, whereas *T. siluroides* feeds on smaller fishes [25]. The genome resource for *T. bleekeri* will contribute to understanding its evolution and environmental adaption and explore the convergent genetic mechanisms of *Triplophysa* species in high-elevation adaption.

In this study, we generated the first chromosomal genome sequence of *T. bleekeri* using combined Illumina, PacBio, and Hi-C technology. Evolutionary and comparative genomic approaches were applied to clarify the origin of *T. bleekeri* and to investigate the potential signals of adaption. Furthermore, the population genetics of *T. bleekeri* were also investigated to reveal the genetic divergence among different populations.

## Materials and Methods

### Samples and tissue collection

**Figure 1:**
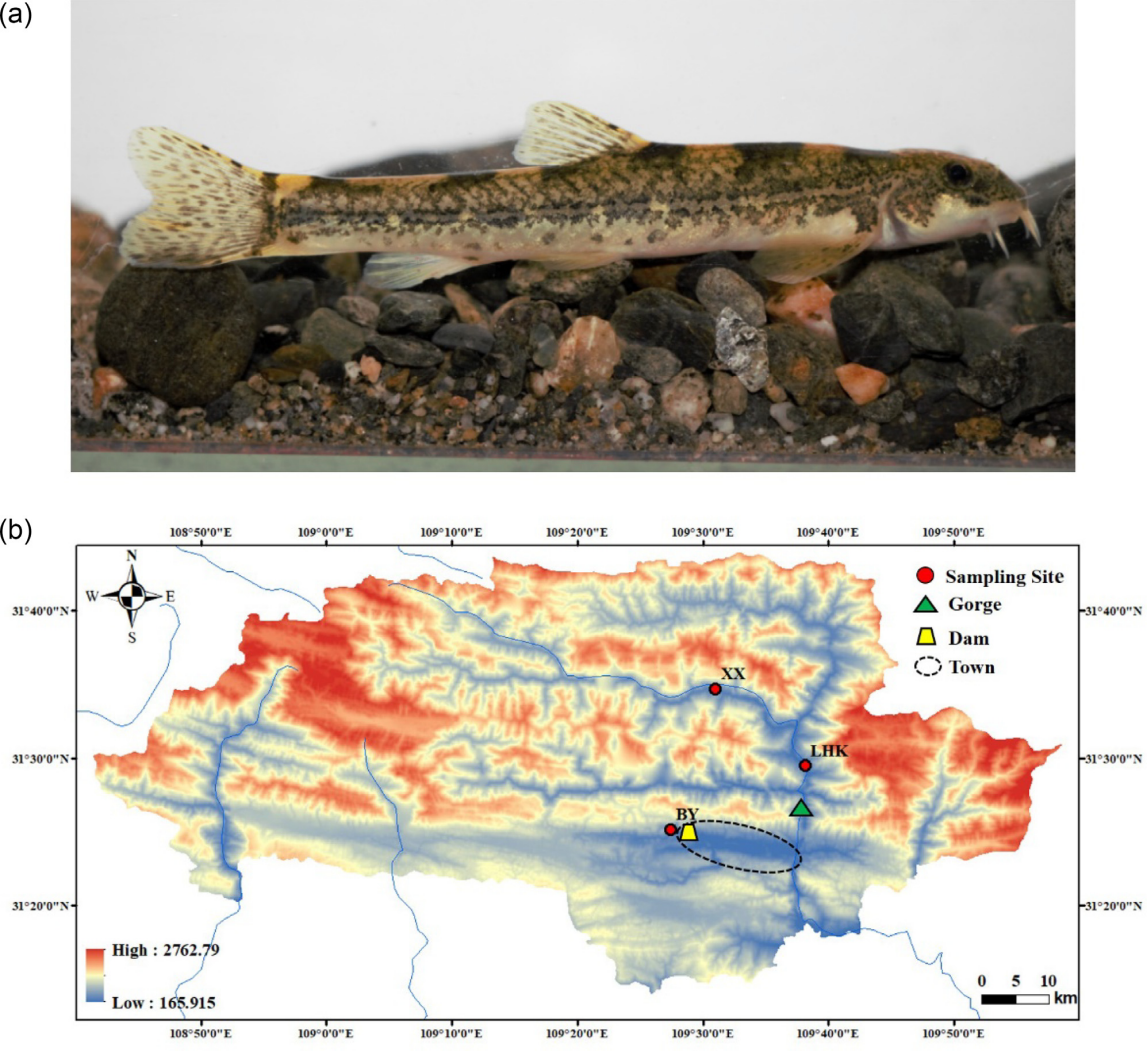
Morphology and geographic distribution of *T. bleekeri*. (a) *T. bleekeri* used in this study. (b) Geographic distribution of the sampling locations for *T. bleekeri*. The red circles, green triangle, yellow trapezoid, and dotted ellipse represent the sampling sites, gorge, artificial dam, and Wuxi Town, respectively.

*T. bleekeri* individuals (Fig. 1a; NCBI:txid595395; fishbase ID: 56059) were obtained from the Daning River (31.157383 N,109.892133 E.), a tributary in the upper reaches of the Yangtze River, using brail nets (Fig. 1b). The fish were then transferred to the Aquaculture Laboratory of Southwest University and reared in indoor tanks. To collect enough tissues for the genome and transcriptome sequencing, the largest female individual was used for library construction and sequencing. The fish was anesthetized with tricaine MS-222 and was immediately dissected to collect 12 types of tissues, viz., brain, eye, skin, gill, heart, liver, trunk kidney, spleen, gut, muscle, gallbladder, and gonad. Tissues were quickly frozen in liquid nitrogen for >1 hour and then stored at −80°C. Among these tissues, muscle tissue was used for genomic DNA sequencing and Hi-C library construction. Meanwhile, all tissue samples were used in the application of transcriptome sequencing to comprehensively characterize the transcriptome. To elucidate the population structures of *T. bleekeri*, a total of 28 individuals were collected from 3 different reaches of the Daning River, i.e., 11, 11, and 6 individuals from Lianghekou (LHK), Xixi (XX), and Baiyang (BY), respectively (Fig. 1b). These individuals were anesthetized with tricaine MS-222, and muscle tissue samples of each fish were collected in the aforementioned manner.

### Genome DNA extraction and sequencing library construction

DNA was extracted from muscle tissue using the phenol-chloroform DNA extraction method [26]. The Qubit (Thermo Fisher Scientific, Waltham, MA, USA) and Agilent Bioanalyzer 2100 (Agilent Technologies, Palo Alto, CA, USA) were used for evaluating the quantity and quality of DNA. For sequencing based on the Illumina HiSeq technology, a short-read sequencing library with an insert size of 250 bp was constructed using 1 μg of DNA. For sequencing on the PacBio Sequel platform (Pacific Biosciences [PacBio], Menlo Park, CA, USA), the muscle DNA was used to construct the long-read sequencing library. Briefly, 10 μg of *T. bleekeri* genomic DNA was used for 20-kb library preparation following the manufacturer's protocol (PacBio), and the BluePippin Size Selection system (Sage Science, Beverly, MA, USA) was used for library size selection. DNA molecules from the largest individual were sequenced using the PacBio and Illumina platforms for genome assembly, and other samples were subjected to short-read whole-genome resequencing on the Illumina platform.

### RNA extraction and sequencing library construction

RNA sequencing data provide important evidence for gene prediction in the genome [27]. To include as many expressed genes as possible, the 12 aforementioned tissue types were used for RNA sequencing library construction. RNA was isolated from the 12 tissue samples using TRIzol reagent (Invitrogen, USA). The quantity and quality of extracted RNA were determined using the Nanodrop ND-1000 spectrophotometer (LabTech, Holliston, MA, USA) and 2100 Bioanalyzer (Agilent Technologies, Palo Alto, CA, USA). Samples with a total RNA concentration ≥10 μg and RNA integrity number ≥8 were used for sequencing. RNA molecules extracted from tissues were mixed in equal proportions for the following RNA library construction. The RNA sequence library was constructed following the protocol of Paired-End Sample Preparation Kit (Illumina Inc., San Diego, CA, USA), which was identical to that used in our previous study [28].

### DNA and RNA library sequencing

The short-read DNA and RNA sequencing libraries were sequenced with the 150 bp paired-end (150PE) mode using the Illumina HiSeq X Ten platform (Illumina Inc.). The 20 kb long-read genome DNA SMRTbell libraries sequencing library was sequenced with the PacBio Sequel platform. The raw sequencing data were quality checked before the bioinformatics analysis. The HTQC v0.90.8 package [29] was used to filter low-quality bases and reads, and sequences with adapters or low quality (average quality score < 20) were removed.

### Genome size estimation

The genome size was estimated on the basis of Illumina sequencing data using the *k-*mer method before genome assembly. Raw Illumina reads were processed to remove adapter sequences, reads with >10% N bases, and reads with >50% low-quality bases (≤5). All filtered reads were used for *k*-mer frequency analysis [30]. Using *k*-mer size of 17, the *k*-mer frequencies were obtained using Jellyfish v2.0 software [31]. *k-*mers with a frequency <3 were eliminated because those likely resulted from sequencing errors. The genomic size was estimated on the basis of the following formula: *G* = (*L* − *K* + 1) × *n*_base_/(*C*_*k*-mer_ × *L*), in which *G* is the estimated genome size, *n*_base_ is the total count of bases, *C_k-_*_mer_ is the expectation of *k*-mer depth, *L* indicates the read length, and *K* represents *k-*mer size. The revised genome size was calculated as follows: Revised Genome size = Genome size × (1 − Error Rate).

### *De novo* assembly of the *T. bleekeri* genome

Long reads generated from the PacBio sequencing platform were used for *T. bleekeri* genome assembly with the Falcon v0.3.0 package [32]. The assembled genome sequences were further polished with Arrow using long-read sequencing [33]; thereafter, 2 rounds of polishing using next-generation sequencing short reads were performed with Pilon (Pilon, RRID:SCR_014731) v1.23 [34]. Finally, redundant genomic sequences were eliminated using Redundans v0.14a with the parameter overlap of 0.95 and an identity of 0.95 [35]. Completeness of the assembled genome was evaluated using BUSCO (BUSCO, RRID:SCR_015008) v3.0 [36]. The database of actinopterygii_odb9 was used for the BUSCO analysis.

### Chromosome assembly using Hi-C technology

Muscle tissue (1 g) of *T. bleekeri* was collected for PacBio sequencing and was used for Hi-C library construction. The Hi-C processes, including cross-linking, lysis, chromatin digestion, biotin marking, proximity ligations, cross-linking reversal, and DNA purification, were performed using the protocol described in previous studies [37]. The purified and enriched DNA was used for sequencing library construction. The library was sequenced using the Illumina HiSeq X Ten platform (Illumina), and the short-reads were then mapped to the polished genome of *T. bleekeri* with Bowtie (Bowtie, RRID:SCR_005476) v1.2.2. The chromosomal assembly using interaction frequency matrix extracted from the Hi-C read mapping was performed according to a previously reported methodology [37].

### Repetitive element annotation

The *de novo* prediction and homology prediction were combined to annotate the repetitive sequences in the *T. bleekeri* genome. RepeatModeler (RepeatModeler, RRID:SCR_015027) v2.0.1 [38] was used for the detection of *de novo* repetitive elements in the *T. bleekeri* genome. The detected genome repeats were combined with RepBase library [39], as a comprehensive library for the final prediction of repetitive elements in the *T. bleekeri* genome, using the RepeatMasker (RepeatMasker, RRID:SCR_012954) v4.1.1 software [40]. Transposons were predicted using ProteinMask, and the tandem repeats were identified in the genome using Tandem Repeats Finder v4.10 [41].

### Protein-coding and non-coding gene prediction

The *ab initio* prediction, homology prediction, and RNA-sequencing–based methods were used for protein-coding gene annotation. Gene models for protein-coding genes were first predicted in the *T. bleekeri* genome using Augustus (Augustus: Gene Prediction, RRID:SCR_008417) v2.5.5 [42]. Five closely related fish species, viz., common carp (*Cyprinus carpio*), zebrafish (*Danio rerio*), Japanese medaka (*Oryzias latipes*), green spotted puffer (*Tetraodon nigroviridis*), and three-spined sticklebacks (*Xiphophorus maculatus*), were used for the homology-based protein-coding gene prediction. Protein sequences from those species, available in public databases, were mapped to the genome using TBLASTN [43] and GeneWise (GeneWise, RRID:SCR_015054) [44]. Thereafter, comprehensive transcriptome sequencing data for multiple tissues were aligned to the genome, and gene models were generated using the TopHat (TopHat, RRID:SCR_013035) v2.1.1 package [45] and Cufflinks (Cufflinks, RRID:SCR_014597) v2.2.1 [46]. The integration and redundancy elimination for the gene models predicted using the above methods were performed using the MAKER package (MAKER, RRID:SCR_005309) [47, 48]. We only selected genes with start and stop codons, and genes with internal stop codons were removed. Only genes with complete sequences and 70% overlaps among different gene model prediction methods will be retained as high-quality gene models.

Four types of non-coding RNAs, including microRNAs, transfer RNAs (tRNA), ribosomal RNAs, and small nuclear RNAs, were also predicted in the *T. bleekeri* genome using tRNAscan-SE (tRNAscan-SE, RRID:SCR_010835) v1.3.1 [49] and using Infernal (Infernal, RRID:SCR_011809) v1.1.3 [50] with the Rfam database [51].

### Functional annotation of protein-coding genes

The NCBI non-redundant protein, SWISS-PROT, and TrEMBL databases [52] were used as protein databases for the biological function annotation using BLAST v2.10.1 packages [53]. AN E-value of 1e−5 was used as the threshold for homolog identification. Gene Ontology (GO) [54] and KEGG [55] assignments were performed using Blast2GO (Blast2GO, RRID:SCR_005828) software [56].

### Gene family clustering and phylogenetic analysis

Coding sequences annotated from whole-genome sequences for the closely related species were extracted from genome sequences. Gene family clustering was performed for *T. bleekeri* with 8 fish species living in non-QTP regions, viz., zebrafish, Japanese medaka, elephant shark (*Callorhinchus milii*), spotted gar (*Lepisosteus oculatus*), Atlantic cod (*Gadus morhua*), platyfish (*X. maculatus*), tiger puffer (*Takifugu rubripes*), and large yellow croaker (*Larimichthys corcea*) by the Orthomcl v1.2 pipeline [57] with default settings. The single-copy orthologs across all species were selected for gene family, phylogenetic, and evolutionary analyses. Briefly, proteins of these genes were aligned with MUSCLE (MUSCLE, RRID:SCR_011812) v3.8.31 [58] and were then transformed into alignments of nucleotide sequences with pal2nal [59] on the basis of the corresponding coding sequences. Next, non-conservative regions were removed using Gblocks (Gblocks, RRID:SCR_015945) [60] with default settings, and the conservative regions were concatenated and fed into RaxML (RAxML, RRID:SCR_006086) v8.2.10 [61] to deduce the phylogenetic relationships of these species using a GTRGAMMA model. Rapid bootstrap runs (100 times) were performed to test the robustness of the topology [62]. On the basis of the topology and the alignment matrix, their divergence times were deduced using MCMCTREE included in the PAML (PAML, RRID:SCR_014932) v1.3.1 package [63] with calibration points set by consulting the TimeTree database. *D. rerio* and *Larimichthys crocea* (255–205 Mya), *O. latipes* and *L. crocea* (115–105 Mya), *L. oculatus* and *D. rerio* (338–291 Mya), and *C. milii* and *D. rerio* (497–450 Mya) were used as calibration points for the divergence time estimation for other species.

To investigate the evolutionary relationships within genus *Triplophysa*, we also added another 4 *Triplophysa* genus fish species to the phylogenetic analysis. Because the genomes of *T. xichangensis* and *T. scleroptera* have not been reported, we downloaded the short reads of the transcriptomes of those 2 species from the NCBI SRA and conducted *de novo* assembly using Trinity (Trinity, RRID:SCR_013048) v2.11.0 [64] with default settings. The longest transcripts for each gene were used in the following phylogenetic analysis. The single-copy orthologs across all species were used for phylogenetic tree reconstruction and divergence time estimation following the aforementioned method.

### Gene family expansion and contraction in the *T. bleekeri* genome

To identify expanded and contracted gene families in the *T. bleekeri* genome, we compared gene families in the *T. bleekeri* genome to those fish species living in non-QTP regions, viz., including elephant shark, spotted gar, zebrafish, Japanese medaka, platyfish, tiger puffer, large yellow croaker, Atlantic cod, green spotted puffer, and three-spined sticklebacks. CAFE v4.2.1 [65] was used to analyze the expansion and contraction of gene clusters in the *T. bleekeri* genome using a probabilistic model. A GO enrichment analysis was performed on expanded and contracted genes using the topGO v2.40.0 package [66]. The enrichment of genes in KEGG pathways was also analyzed using KOBAS (KOBAS, RRID:SCR_006350) v1.2.0 [67].

### Positively selected genes in genomes of *Triplophysa* species

MUSCLE v3.8.31 was used for multi-protein sequence alignment among the *T. bleekeri* genes and their orthologs, and compared to 8 fish species living in non-QTP regions used in the gene family clustering analysis. Conserved coding sequence (CDS) alignments of each single-copy gene family were extracted using Gblocks [68] and used for further identification of positively selected genes (PSGs). The ratios of nonsynonymous to synonymous substitutions (K_A_/K_S_, or ω) were estimated for each single-copy orthologous gene using the CodeML program with the branch-site model as implemented in the PAML package. A likelihood ratio test was conducted, and the false discovery rate correction was performed for multiple comparisons. Genes with a corrected *P*-value <0.05 were defined as PSGs. The genes putatively influenced by positive natural selection of *T. tibetana* and *T. siluroides* were also identified using the identical method. The functional annotation of PSGs for *T. bleekeri, T. tibetana*, and *T. siluroides* was also conducted using the same approach with the gene family expansion and contraction analysis.

### Whole-genome resequencing and population genetics

Raw reads of samples subjected to resequencing were quality controlled as mentioned previously. The filtered short reads were mapped using BWA mem (BWA, RRID:SCR_010910) v0.7.17-r1188 with default settings for each individual, followed by the marking of duplicates with Picard (Picard, RRID:SCR_006525). Regions near INDELs were thought to be poorly aligned and were identified and realigned using GATK (GATK, RRID:SCR_001876) v4.1.8.1 [69]. GATK was also used to call single-nucleotide polymorphisms (SNPs) and INDELs based on the alignments. The SNPs and INDELs were then filtered by these parameters: QUAL (phred quality) > 30, QD (quality score divided by depth to comprehensively evaluate the quality and depth) > 2, DP (read depth) > 5, FS (Phred-scaled *P*-value using Fisher exact test to detect strand bias for reads) < 60, MQ (mapping quality to evaluate read alignment) > 40,  SOR (strand odds ratio to evaluate strand bias for reads) < 4.0. The identified SNPs were filtered using SNPhylo v20180901 [70] with default settings, except for LD_threshold and Minimum_depth_of_coverage, which were set to 0.8 and 5, respectively. Next, the principal component analysis (PCA) clusters and population structure for these individuals were deduced with Plink (PLINK, RRID:SCR_001757) v1.9 [71] and Admixture [72, 73] with default settings, respectively. Their phylogenetic relationships were recovered using the neighbor-joining method with MEGA4 [73], and bootstrap resampling (100 times) was performed to test the robustness of the tree topology.

### Historical effective population size inference for *T. bleekeri*

Historical effective population size of *T. bleekeri* was estimated using Pairwise Sequentially Markovian Coalescent (PSMC) v0.6.5 software [74]. We used the data for whole-genome variants of individuals for the genome assembly. The consensus sequences were generated using vcfutils.pl (vcf2fq -d 10 -D 300). The fq2psmcfa tool was used to create the input file for PSMC modeling. Sequences were used as the input for the PSMC estimates using “psmc” with the options -N25 -t15 -r5. The reconstructed population history was plotted using “psmc_plot.pl” with the generation time of 2 years and rate of 4 × 10^−9^ substitutions per synonymous site per year. The mutation rate was estimated from the gene comparison of *T. bleekeri* and *D. rerio*. Bootstrapping was conducted by randomly sampling with replacement 5‐Mb sequence segments and 100 bootstrap replicates were performed.

### Selection sweep analysis for populations

To identify genome-wide selective sweeps among populations, we calculated the genome-wide distribution of fixation index (F_ST_) values and θπ ratios using SNPs from different populations. The F_ST_ values were Z-transformed as follows: Z (F_ST_) = (F_ST_− µF_ST_)/σF_ST_, in which µF_ST_ is the mean F_ST_ and σF_ST_ is the standard deviation of F_ST_. The θπ ratios were log_2_-transformed. Subsequently, we scanned the genome in a 1-kb sliding scale, and estimated and ranked the empirical percentiles of Z (F_ST_) and log_2_ (θπ ratio) in each window. We considered the windows with the top 1% Z (F_ST_) and log_2_ (θπ ratio) as candidate outliers under strong selective sweeps. Genes residing in the outlier regions were considered as the candidate functional genes. The GO and KEGG enrichment were carried out by cluster Profiler v3.14.3 [75] and DAVID v6.8 [76].

## Results

### DNA and RNA library sequencing

We generated 81.69 Gb genomic (∼120×) and 10.6 Gb transcriptomic short reads for the following genome size estimation and annotation (Table 1). We also obtained 100.87 Gb genomic long reads from the PacBio platform, with a rough coverage of 160× for the *T. bleekeri* genome (Table 1). The mean and N50 length of the long reads were 5.8 and 16 kb, respectively (Table 1 and Supplementary Fig. S1).

**Table 1:** A summary of sequencing data used in genome assembly and gene annotation

Source	Platform	Clean data (Gb)	Mean read length (bp)	Sequence coverage (×)
Genome	Illumina HiSeq X Ten	81.7	150	129
	PacBio Sequel	100.87	5,827	160
Genome (Hi-C)	Illumina HiSeq X Ten	83.5	150	132
Transcriptome	Illumina HiSeq X Ten	11.1	150	

### Genome size estimation

To determine the possible sample contamination, 10,000 next-generation sequencing short reads were randomly selected for an NCBI nt database search. *Cyprinus, Danio*, and *Sinocyclocheilus* represent the top 3 sources of best hits, ruling out obvious contamination during library construction and sequencing. Using genomic short reads generated from the Illumina platform, 59.8 million *k*-mers were generated. The genome of *T. bleekeri* was estimated as 632.5 Mb, with a heterozygosity ratio of 0.26% and repeat content of 42.2% (Supplementary Fig. S2). Based on the above genome character estimation, the genome of *T. bleekeri* was mid-sized with low heterozygosity.

### *De novo* assembly of the *T. bleekeri* genome

Using genomic PacBio long reads for *T. bleekeri*, we assembled a 628-Mb genome with 856 contigs and an N50 length of 3.82 Mb (Table 2). Among these contigs, the longest contig for the genome was 15.5 Mb. The completeness of the assembled genome was evaluated using BUSCO v3.0 [36] with the actinopterygii_odb9 database, indicating that 92.9% of BUSCO genes were identified in the assembled genome (Supplementary Fig. S3).

**Table 2:** Length statistics for contig assembly for the *T. bleekeri* genome

Assembly	Total length (bp)	Sequence No.	Contig N50 (Mb)	Scaffold N50 (Mb)
Contig assembly using long-read data				
Falcon	657,392,105	1,357	3.31	3.31
Arrow	660,275,268	1,357	3.33	3.33
Pilon	659,964,583	1,357	3.33	3.33
Redundans	628,132,429	856	3.82	3.82
Chromosome assembly using Hi-C data				
All sequences	620,272,795	181	3.11	22.89
Chromosomes	596,964,218	25	3.23	23.21
Unanchored sequences	23,308,577	156	0.17	1.01

### Chromosome assembly using Hi-C technology

Hi-C technology recruits interaction information among different chromosome regions and assumes that the interactions for nearby regions are more prevalent than for distant regions. In this study, 82.9 Gb sequencing data were obtained via Hi-C library sequencing. On the basis of the interaction information, a chromosome assembly of 628 Mb with a scaffold N50 length of 22.9 Mb was obtained (Supplementary Fig. S4). More than 596.9 Mb sequences were anchored upon 25 chromosomes, highlighting a high chromosome anchoring rate of 96.2% on the base level.

### Repetitive element annotation

The annotation pipeline showed that >17.9 Mb of the genome sequences were predicted as tandem repeats, covering ∼2.8% of the genome, and finally 203.2 Mb, accounting for ∼32.4% of the genome, were annotated as repetitive elements in the *T. bleekeri* genome (Supplementary Table S1). Specifically, there are 17.2% DNA transposons (107.8 Mb), 5.8% of long interspersed nuclear elements (LINEs) (36.4 Mb), 0.68% short interspersed nuclear elements (SINEs) (4.3 Mb), and 6.93% long terminal repeats (43.5 Mb).

### Protein- and non-coding gene prediction, and functional annotation

For predicting protein-coding genes in the *de novo* assembled genome, 10.6 Gb short-read transcriptome data from 12 tissues were generated. Based on the *de novo*, homolog, and RNA-seq data methods, a total of 20,274, 27,243, and 15,875 protein-coding genes were predicted, respectively. After integration and redundancy elimination, 21,198 protein-coding genes were predicted in the *T. bleekeri* genome (Supplementary Table S2).

Of the 21,198 protein-coding genes, roughly 93.0%, 96.9%, and 90.9% displayed homologous sequences in the NCBI NR, TrEMBL, and Swissprot databases, respectively. Additionally, 89.2% contained InterPro domains, and 46.9% were assigned GO terms. Overall, >97.3% of the protein-coding genes were functionally annotated by ≥1 method (Supplementary Fig. S5). Non-coding genes have received increased attention in recent years because accumulating evidence suggests that many of them play crucial roles in a variety of biological processes [77]. In this study, all the possible non-coding DNA sequences were predicted based on the *de novo* prediction strategies and are summarized in Supplementary Table S3.

### Gene family clustering and phylogenetic analysis of *T. bleekeri*

Using the whole-genome and transcriptome data of 4 other *Triplophysa* species, viz., *T. tibetana, T. siluroides, T. scleroptera*, and *T. xichangensis*, and the 8 other fish species living in non-QTP regions, we performed gene family clustering for those species. As a result, we identified 1,364 single-copy orthologs among those fish species.

**Figure 2:**
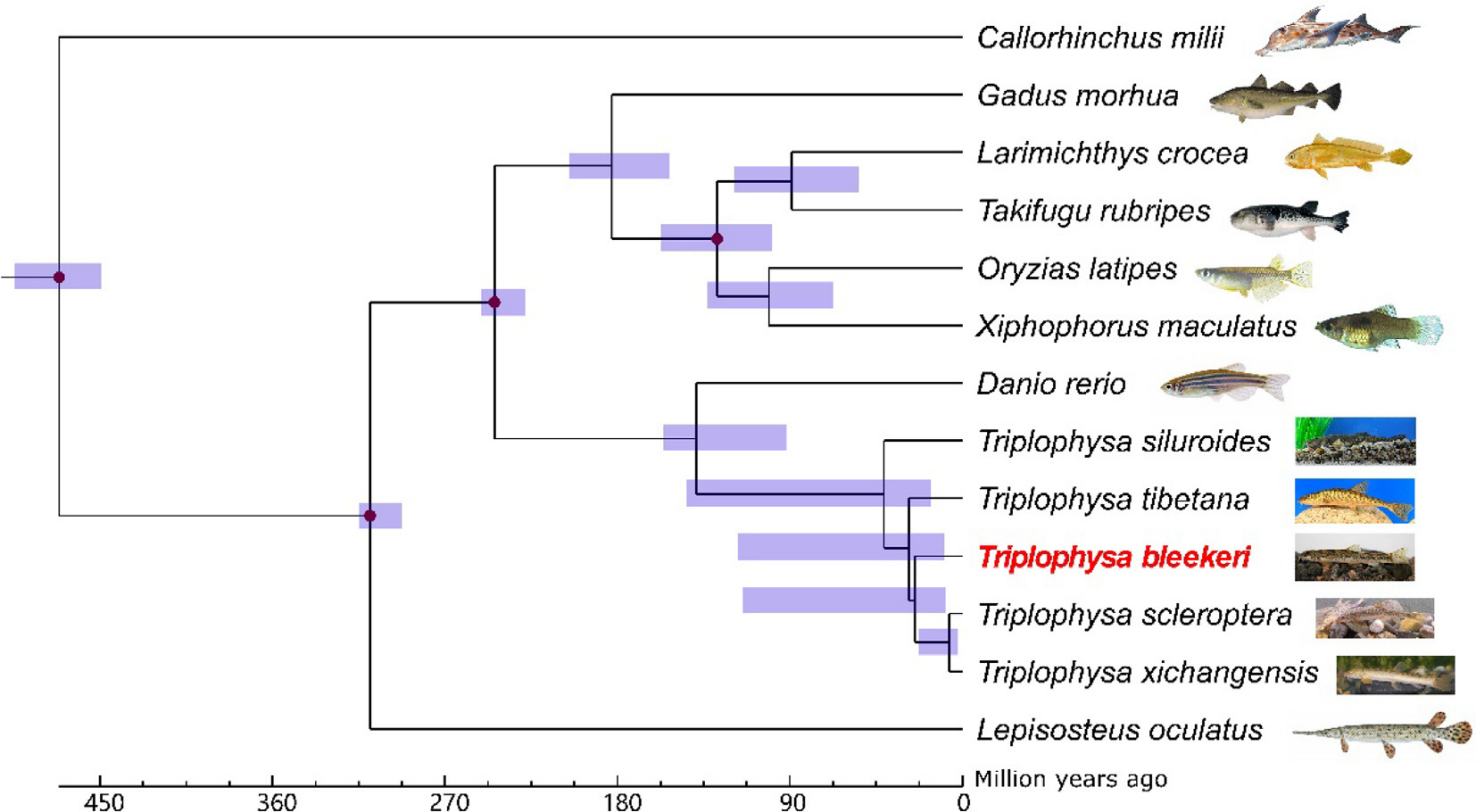
Phylogenetic relationships and divergence time estimation for *T. bleekeri* and other fish species. All nodes were completed and supported by 100 cycles of bootstrap resampling. Numbers near the nodes (shown in blue) indicate the estimated divergence times with a 95% confidence interval. Divergences used for the recalibration of time estimation are indicated with red dots.

We then investigated the evolutionary relationship of *T. bleekeri* with respect to other *Triplophysa* and the non-QTP species. Using single-copy genes among species, a concatenated alignment matrix was generated with a total length of 73,887 bp, which was used for the phylogenetic analysis and divergence time estimation. The result showed that *Triplophysa* species are phylogenetically closer to *D. rerio*and that *T. siluroides* is a basal species within the *Triplophysa* group. Divergence time estimation showed that *T. bleekeri* diverged from their common ancestor, *T. scleroptera* and *T. xichangensis*, ∼25.2 million years ago (Mya) (Fig. 2).

### Genes under natural positive selection

**Figure 3:**
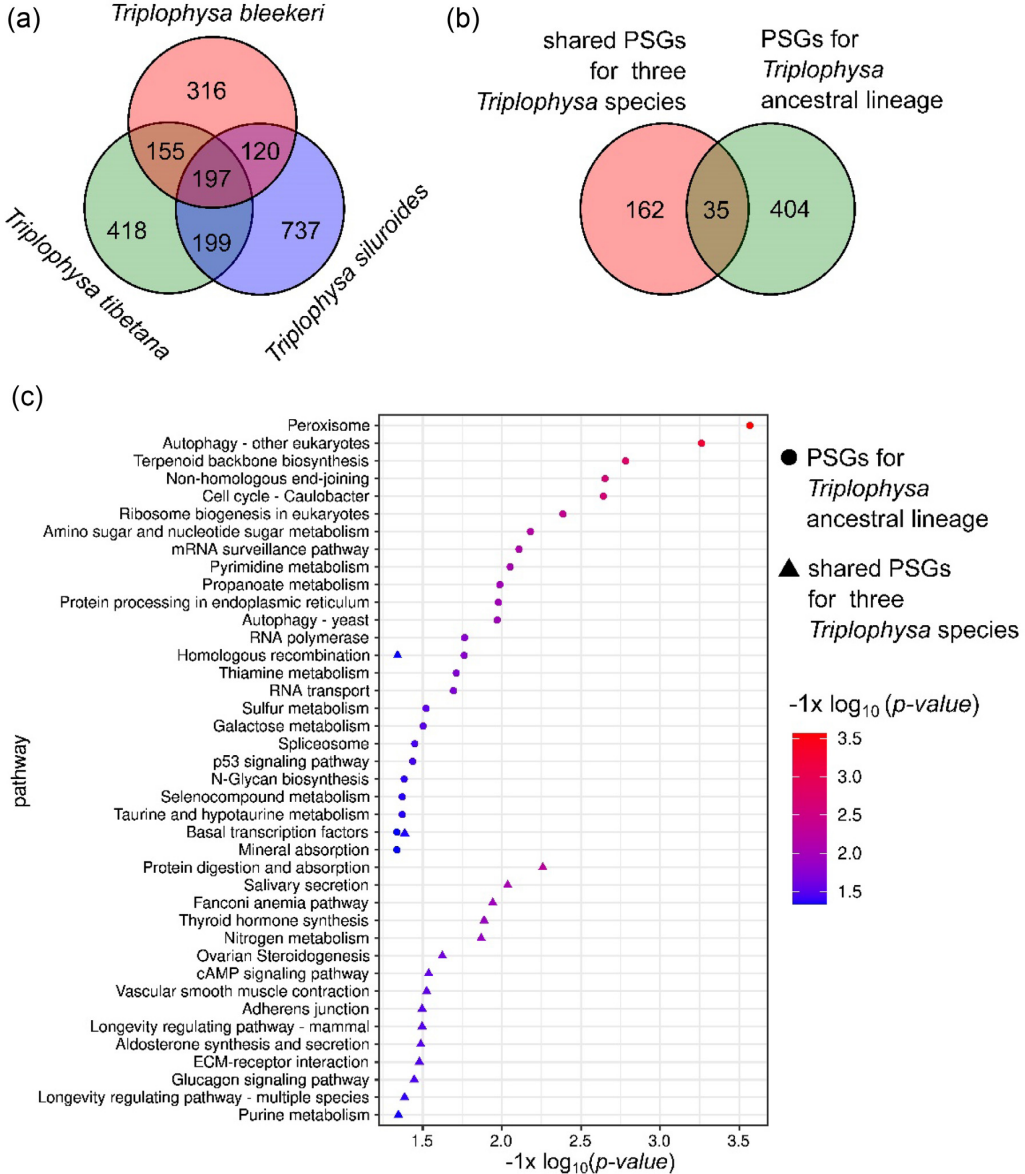
Natural positively selected gene (PSG) identification and functional analysis for *T. bleekeri, T. tibetana*, and *T. siluroides*. (a) Venn diagram for PSGs for the 3 fish species. (b) Venn diagram for PSs identified from species- and lineage-based method. (c) Enrichment analysis on the biological pathways for candidate PSGs identified from the species- and lineage-based method. mRNA: messenger RNA.

We identified 788 PSGs in the *T. bleekeri* genome. The functional analysis on the KEGG and GO parameters showed that several categories associated with nucleotide metabolism and DNA repair were significantly enriched (Supplementary Tables S4 and S5). Additionally, the PSGs were also enriched in immune response, such as MyD88-dependent Toll-like receptor signaling pathway (Supplementary Table S4). Concomitantly, 969 and 1,253 PSGs were identified for *T. tibetana* and *T. siluroides*, respectively. Among those genes, 197 genes were identified as shared PSGs for the 3 *Triplophysa* species (Fig. 3a).

To detect candidate PSGs for *Triplophysa* ancestral lineage, we also performed PSG identification for the common ancestor of the *Triplophysa* with the branch-site model in PAML. As a result, we identified 439 PSGs for the *Triplophysa* ancestral lineage. Interestingly, we found that only 35 shared PSGs for the 3 *Triplophysa* species were identical to *Triplophysa* lineage PSGs (Fig. 3b). The functional analysis with respect to biological pathways for the 3 *Triplophysa* species showed that those genes were significantly enriched for various processes including protein digestion and absorption, Fanconi anemia pathway, and salivary secretion (Fig. 3c). Twenty-five biological pathways, including peroxisome, autophagy, non-homologous end-joining, homologous recombination, basal transcription, ribosome biogenesis, and spliceosome, were enriched for PSGs of *Triplophysa* ancestral lineage (Fig. 3c). The homologous recombination and basal transcription factor pathways were both enriched for *Triplophysa* lineage PSGs and the 3 *Triplophysa* species shared PSGs (Fig. 3c).

### Gene family expansion and contraction in the *T. bleekeri* genome

Following the Orthomcl pipeline, 21,862 ortholog groups were obtained after gene family clustering with 10 fish species from non-QTP regions. Gene family analysis showed that 1,533 and 2,401 gene families were significantly expanded and contracted in *T. bleekeri*, respectively (Supplementary Fig. S6). The functional enrichment of expanded gene families was analyzed using GO and KEGG. The expanded gene families were primarily enriched in categories of metabolism and immune regulation (Supplementary Tables S6 and S7). The categories of metabolism include fatty acid metabolism (arachidonic acid metabolism and glycosphingolipid biosynthesis), carbohydrate metabolism (glycosaminoglycan biosynthesis and glycan degradation), and amino acid metabolism (RNA transport). The categories of immune regulation include the Hippo signaling pathway (corrected *P*-value = 2.40E−03), necroptosis, and vitamin B_6_ metabolism (corrected *P*-value = 8.90E−03). The contracted gene families were mainly made up of several signaling pathways, including the MAPK signaling pathway, calcium signaling pathway, adrenergic signaling in cardiomyocytes, GnRH signaling pathway, and retrograde endocannabinoid signaling (Supplementary Tables S8 and S9).

### Historical effective population size for *T. bleekeri* during formation of the QTP

**Figure 4:**
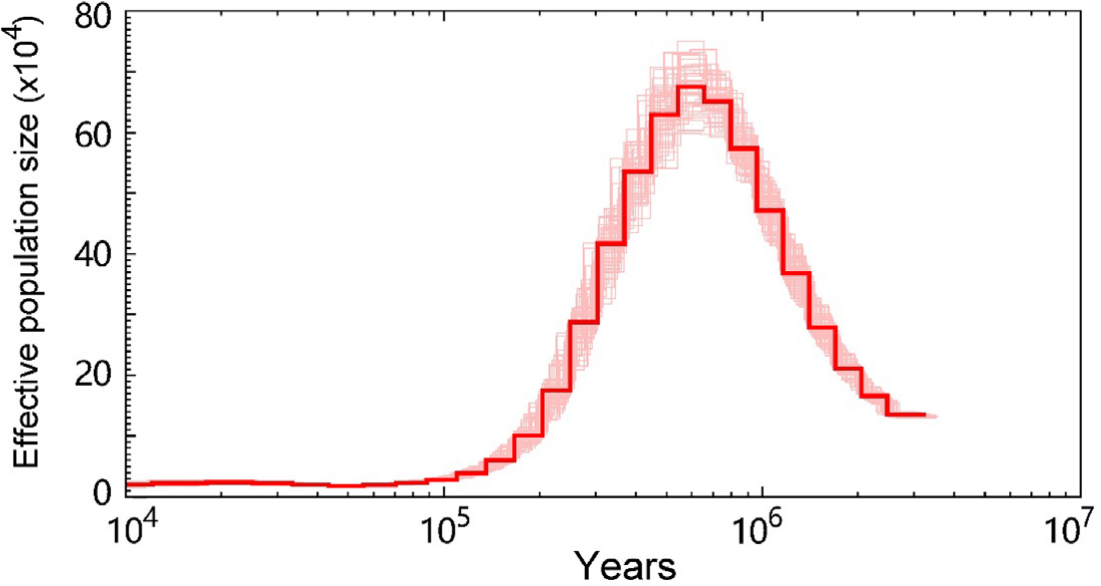
Historical effective population size profile deduced from the whole-genome sequencing data. One hundred bootstrap replicates were performed for the effective population size estimation.

We used the whole-genome short-read sequencing data based on the sample used for genome assembly to obtain the genome-wide genotype data. Then, those variants were used to probe the profiles of historical effective population size for *T. bleekeri* during the formation of the QTP. We used gene comparison between *T. bleekeri* and *D. rerio* to estimate the mutation rate. As a result, we estimated a mutation rate of 4 × 10^−9^ for *T. bleekeri*. PSMC analysis performed using the above data showed that the effective population size of *T. bleekeri* increased >0.7 Mya and reached a peak of 70 × 10^4^ ∼0.6–0.7 Mya. However, the *T. bleekeri* population size experienced a dramatic decrease afterwards to 1 × 10^4^ from 0.6 Mya to 60,000 years ago (Fig. 4). The effective population size decline was consistent with the accelerating QTP uplift ∼1 Mya [78] and the quaternary glaciation spanning the Pleistocene (2.6–0.11 Mya) and Holocene (0.11–0 Mya) [19, 79]. We speculate that both the geotectonic movements and temperature fluctuations during the period exerted intense survival pressure on the ancient *T. bleekeri* populations, leading to the ∼70 times effective population size drop during the period.

### Population genetics analysis of *T. bleekeri*

**Figure 5:**
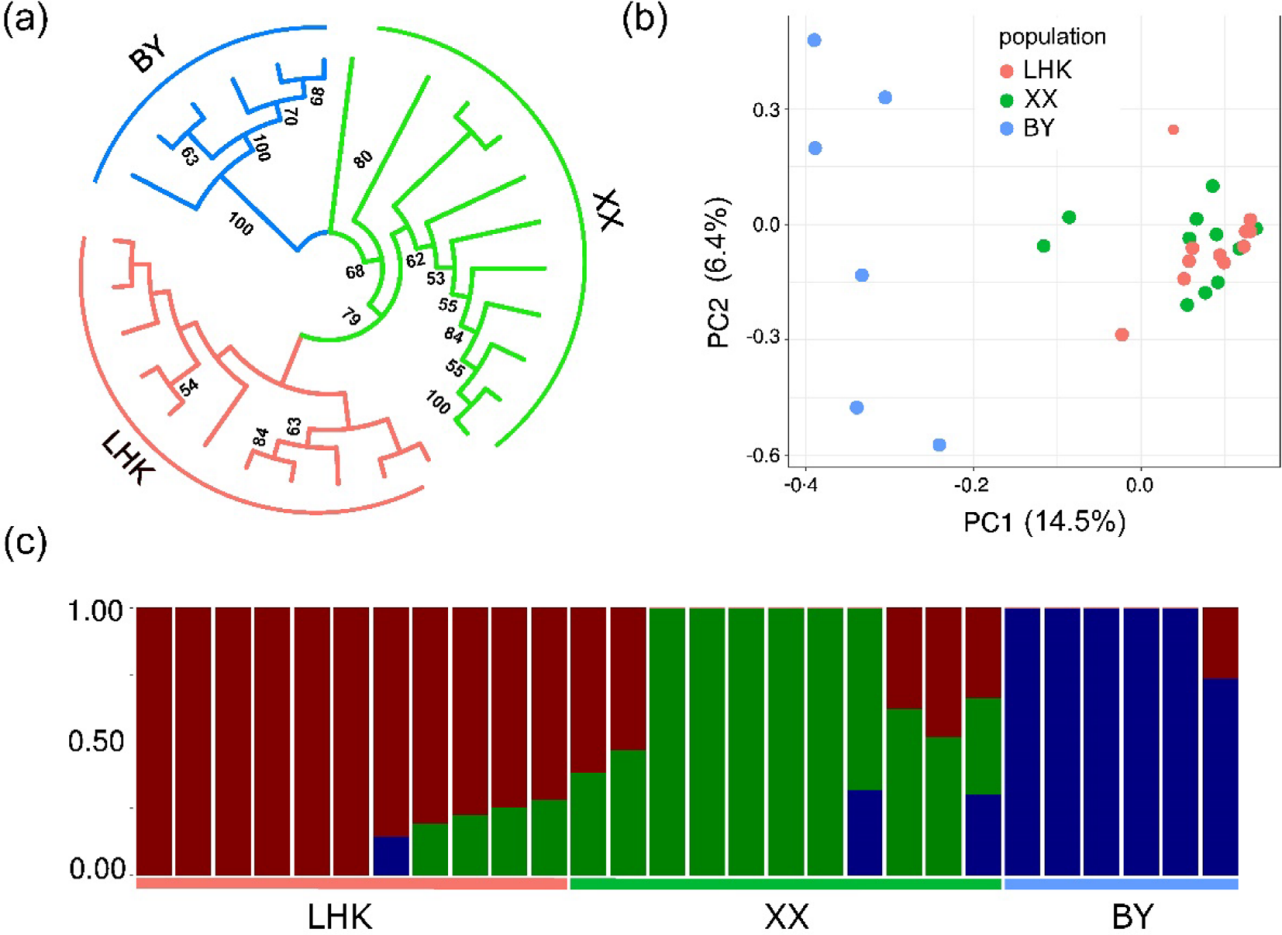
Population genetics analysis for *T. bleekeri*. (a) Neighbor-joining phylogenetic tree of individuals based on whole-genome SNP loci. Samples from population LHK, XX, and BY are labeled with red, green and blue, respectively. (b) Principal component (PC) analysis plots of the first 2 components. The fraction of the variance obtained was 14.5% for PC1 and 6.4% for PC2. (c) Population structure plots of *T. bleekeri*. The samples from population LHK, XX, and BY are represented by red, green, and blue color, respectively. We assume that there were 3 populations for the analysis (*K* = 3). The y axis quantifies the proportion of the individual's genome from inferred ancestral populations, and x axis shows the different populations.

The high-quality SNPs were obtained according to the filtering criteria set previously and were used to deduce the population structures of *T. bleekeri*. As a result, >34 million short reads were obtained for 28 individuals, and >3 million SNPs were detected for all individuals. The phylogeny reconstruction analyses based on whole-genome SNPs showed that individuals from populations LHK and XX clustered together forming 2 neighboring groups, whereas individuals from population BY formed another cluster (Fig. 5a). The PCA clusters (Fig. 5b) also suggested that the first 2 principal components could successfully separate the individuals in population BY from those in populations LHK and XX. In addition, genetic structure analysis also indicated that gene flow between population BY and the other 2 populations might be limited (Fig. 5c).

**Figure 6:**
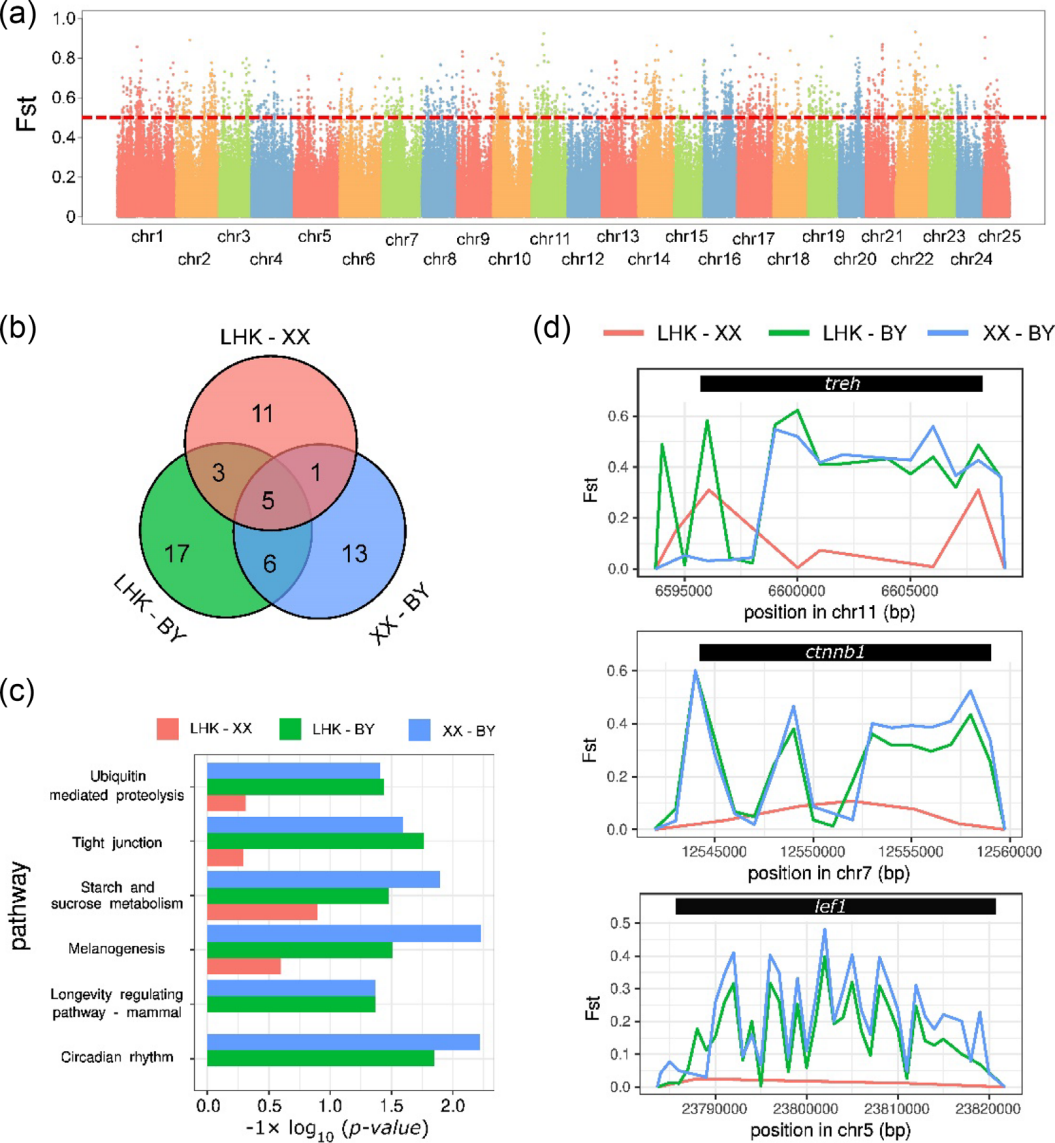
Selective sweep analysis to identify candidate selected functional genes among populations. (a) Manhattan plot to show the genome-wide differentiation between LHK and BY populations. (b) Venn plot for shared enriched biological pathway for candidate selected functional genes from the selective sweep analysis among population comparisons. (c) The shared enriched biological pathway from LHK-BY and XX-BY comparisons. (d) The Fst profiles for genomic regions containing *treh, ctnnb1*, and *lef1* genes.

To identify putative signals of differential selection among populations, we also performed selective sweep analysis for the BY, LHK, and XX populations (Fig. 6a). Based on Fst comparison among those populations (Supplementary Table S10), we identified genomic regions (∼1 kb in length) that scored in the top 1% (Supplementary Fig. S7). As a result, 1,734, 3,009, and 3,244 regions (1 kb) harboring 474, 878, and 957 functional candidate genes were identified to be significantly genetically differentiated for LHK-XX, LHK-BY, and XX-BY comparisons, respectively. Genomic regions with less differentiation identified in LHK-XX comparison were consistent with the above phylogenetic analysis. The GO and KEGG pathway functional analyses showed 20, 25, and 31 significant biological pathway enrichments for LHK-XX, XX-BY, and LHK-BY comparisons, respectively (Fig. 6b, Supplementary Tables S11–S13). Six enriched biological pathways were shared in the LHK-BY and XX-BY comparisons but not in LHK-XX, viz., ubiquitin-mediated proteolysis, tight junction, starch and sucrose metabolism, melanogenesis, longevity-regulating pathway—mammal, and circadian rhythm (Fig. 6c, Supplementary Tables S12 and S13). Five enriched biological pathways were shared for all comparisons, viz., axon guidance, long-term potentiation, Rap1 signaling pathway, circadian entrainment, and calcium signaling pathway (Fig. 6b). Besides, the α-trehalose glucohydrolase (treh), β-catenin (ctnnb1), and lymphoid enhancer-binding factor 1 (lef1) genes exhibited significant genetic differentiation in the LHK-BY and XX-BY comparisons but not in LHK-XX, implying that these genes might be related to the living environments for BY (Fig. 6d).

## Discussion

In this study, we presented the chromosome-level genome assembly of *T. bleekeri* with a contig N50 of 3.1 Mb and a scaffold N50 of 22.9 Mb. The N50 lengths of contigs of the *T. bleekeri* genome assembly were much longer than previously reported genome assemblies of *T. tibetana* [17]. Twenty-five chromosomes were obtained with the mounting rate up to 96.2%, and the assembled chromosome number was consistent with the karyotype of *T. bleekeri* (unpublished data), which suggests that the present analysis resulted in successful assembly of the *T. bleekeri* genome to the chromosome level. The completeness of the genome was also evaluated, confirming the high quality of the assembled *T. bleekeri* genome. The combined results of the homology-based and *de novo* predictions showed that repetitive sequences accounted for 32.4% of the genome. Among them, DNA transposons represented the most abundant tandem repeats, which was also observed in *T. tibetana* [17]. Within the genome, 21,198 protein-coding genes were predicted, of which 97.3% could be functionally annotated. Overall, this genome assembly and annotation provides valuable data to the genomic resources currently available for the study of phylogeny and environmental adaptations of *Triplophysa* species.

The phylogenetic analysis results indicated that the *Triplophysa* genus formed a clade with *D. rerio* and that *T. bleekeri* was most closely related to *T. tibetana* and *T. scleroptera*. The divergence time estimation indicated that *T. siluroides* diverged from their common ancestor roughly 38.8 Mya, occupying a basal position in the *Triplophysa* genus. The extensive QTP was elevated by >4,000 m ∼40 Mya [80], and this time is consistent with the divergence of *T. siluroides*. Therefore, we speculated that the speciation of *Triplophysa* was likely triggered by the uplifting of the QTP [81].

Uplift of the QTP induced profound climatic and environmental changes to the plateau and its peripheral regions, including low oxygen and low temperature [82]. The oxygen content of air is inadequate in the QTP, while investigations into water quality indicated that a high dissolved oxygen concentration exists in the QTP water [83–86]. This led us to speculate that thermal stress may present a major factor in natural selection for fish species in the QTP and its peripheral regions. Although *Triplophysa* species are widely distributed in different regions, these regions are all generally characterized by a cold environment [23, 87]. However, to our knowledge, only a few studies have been conducted to explore the genetic basis of adaptation of *Triplophysa* species to low temperatures. Through the comparative analysis of the genome with other fish species, we found that the expanded gene families of *T. bleekeri* were significantly (*P* < 0.05) enriched in fatty acid metabolism, including glycosphingolipid biosynthesis and arachidonic acid metabolism pathways. The glycosphingolipid located in the bilayer lipid membrane is a major structural component of cell membranes [88], whereas arachidonic acid, an integral constituent of biological cell membranes, aids in the maintenance of cell membrane fluidity even at low temperatures [89]. Our results suggest that the increased number of genes related to fatty acid metabolism might be responsible for maintaining membrane structure and improving membrane fluidity under cold environments.

In the genome of *T. bleekeri*, significant expansion was also observed in the Hippo signaling pathway gene family, which participates in regulating innate immunity [90, 91]. These results suggest that *T. bleekeri* may tend to increase gene numbers in certain families related to immune response for improving the defense against pathogens. It is notable that genes involved in innate immunity, such as Toll-like receptor signaling pathway genes, all underwent positive selection in *T. bleekeri, T. tibetana*, and *T. siluroides*. Similar results were also observed in previous transcriptomic studies of Tibetan Schizothoracinae species, *Gymnocypris przewalskii*, and *G. przewalskii ganzihonensis* [92, 93]. These results indicated that the adaptive evolution of innate immunity might play crucial roles in the highland adaptation of fish.

Low temperatures and UV radiation can cause DNA damage [94], and DNA damage response and repair pathways may show functional adaptation. Within the 3 *Triplophysa* species, the PSGs were enriched in the functional categories of nucleotide excision repair, non-homologous end-joining, homologous recombination, and Fanconi anemia pathways (Supplementary Table S4 and S5). These pathways all participate in DNA repair, of which non-homologous end-joining and homologous recombination are the 2 main pathways for repairing double-strand break [95], and the Fanconi anemia pathway is essential for the repair of DNA interstrand crosslinks [96]. PSGs influencing DNA repair may contribute to DNA integrity and genomic stability under high-altitude environments with low temperatures and intense UV radiation. Our results suggest that *Triplophysa* species have evolved an integrated DNA-repair mechanism to adapt to high-altitude environments. The previous studies also showed that genes involved in DNA repair were under positive selection pressure in many species living at high altitudes, such as the snub-nosed monkey [97] and the Tibetan hot-spring snake [11]. This indicated that DNA damage caused by the environment is a common stress that animals in high-altitude regions need to cope with. We also identified 197 PSGs shared by the 3 *Triplophysa* species (Fig. 3a), indicating that those naturally selected genes might have originated from their common ancestor and that *Triplophysa* species were genetically convergent on PSGs. We found many species-specific PSGs for the 3 *Triplophysa* species. The result implies the requirement of a distinct ecological niche for *T. bleekeri, T. tibetana*, and *T. siluroides*. On the basis of the generally used genomic comparison methods, hundreds of PSGs for *Triplophysa* species were identified in this investigation. However, a previous study has shown that ancient demographic fluctuation could generate severe overestimation of selective signatures [98]. Therefore, PSG identification in this work might have been influenced by the demographic scenarios of *Triplophysa* species. It is worth estimating the demographic fluctuation to PSG identification, and examining the present methods for potential biases.

In addition to comparative genomics analyses, the relationships among populations of *T. bleekeri* were analyzed to probe possible differences in genetic structures. Population structure analysis divided 28 *T. bleekeri* samples into 2 clusters, with individuals from the LHK and XX population grouped together, and individuals from BY population forming the other cluster. Both PCA and structure analyses corroborated these findings. The BY population was separated from the LHK and XX population, and the observed admixture of genetic lineages was limited (*K* = 3). These results could be because LHK and XX are directly connected by the river, and gene flow between individuals residing in the 2 places occurs more frequently. The difference between the BY population and the LHK and XX populations might be attributed to the relatively limited gene flow caused by natural and artificial barriers among those populations. The Daning River measures a height of up to 1,648 m, which flows through many narrower canyons [99]. Therefore, the geographical barriers formed by canyons and shallows could contribute to the diminished interaction among those populations. More importantly, artificial barriers, such as cities and dams, could also weaken the migrations between the BY and LHK/XX populations. Therefore, the whole-genome resequencing data of *T. bleekeri* provided a valuable genetic resource to reveal that geographical and artificial barriers could distinctly influence genetic exchange among populations.

The selective sweep analysis showed that genomic differentiation of LHK-XX was nonintensive compared to that of the BY population, which is consistent with the above population phylogenetic analysis. Notably, we identified 6 shared enriched biological pathways for LHK-BY and XX-BY comparisons but not in LHK-XX. The natural gorge might change the water flow and biodiversity of environments, and human activity could as well influence the nutrition supplies and circadian rhythm for local fish populations directly.

## Conclusions

We present a chromosomal-scale genome assembly of *T. bleekeri*, a representative high-altitude fish. Evolutionary, comparative, and population genomic analyses were performed to investigate the evolution, environmental adaptation, and genetic diversity of *T. bleekeri*. Our results provide insights into how fish adapt to the high-altitude environment, and the genomic data serve as a valuable resource for further study on functional validation of candidate genes contributing to environmental adaptation.

## Data Availability

The genomic, transcriptome, and Hi-C sequencing reads generated from the PacBio and Illumina platforms are available in the NCBI SRA database under the Accession No. SRP200140. The final chromosome assembly was submitted to NCBI (BioProject ID PRJNA545014, assembly VFQW00000000). Supporting data, including assembly and annotation files, are also available via the *GigaScience* database, GigaDB [100].

## Additional Files

Supplementary Figure S1. Read length distribution for PacBio long read sequencing.

Supplementary Figure S2. Kmer frequency distribution from NGS short-read sequencing data.

Supplementary Figure S3. The identification of BUSCO genes in the assembled genome.

Supplementary Figure S4. The interaction frequency among contigs for chromosome assembly.

Supplementary Figure S5. Functional annotations for predicted protein-coding genes for T. bleekeri.

Supplementary Figure S6. The number of expanded and contracted gene families deduced using cafe for each branch.

Supplementary Figure S7. Manhattan plot showing the genome-wide differentiation for LHK-XX (a) and XX-BY (b) comparisons.

Supplementary Table S1. The repetitive element annotation for the T. bleekeri genome.

Supplementary Table S2. The protein-coding gene annotation in the T. bleekeri genome.

Supplementary Table S3. The non-coding genes predicted in the genome.

Supplementary Table S4. GO pathway enrichment analyses for natural positively selected genes.

Supplementary Table S5. KEGG enrichment analyses for natural positively selected genes.

Supplementary Table S6. KEGG pathway enrichment analyses for the members of gene families subject to expansions during the evolution of the genome.

Supplementary Table S7. GO enrichment analyses for the members of gene families subject to expansions during the evolution of the genome.

Supplementary Table S8. KEGG pathway enrichment analyses for the members of gene families subject to contractions during the evolution of the genome.

Supplementary Table S9. GO enrichment analyses for the members of gene families subject to contractions during the evolution of the genome.

Supplementary Table S10. Fst calculation for populations.

Supplementary Table S11. KEGG pathway enrichment analyses for candidate selected genes by comparing LHK and XX populations.

Supplementary Table S12. KEGG pathway enrichment analyses for candidate selected genes by comparing LHK and BY populations.

Supplementary Table S13. KEGG pathway enrichment analyses for candidate selected genes by comparing XX and BY populations.

## Abbreviations

BLAST: Basic Local Alignment Search Tool; bp: base pairs; BUSCO: Benchmarking Universal Single-Copy Orthologs; BWA: Burrows-Wheeler Aligner; BY: Baiyang; CAFE: Computational Analysis of Gene Family Evolution; CDS: conserved coding sequence; GATK: Genome Analysis Toolkit; Gb: gigabase pairs; GO: Gene Ontology; Hi-C: high-throughput chromosome conformation capture; HTQC: High-Throughput Quality Control; kb: kilobase pairs; KEGG: Kyoto Encyclopedia of Genes and Genomes; LHK: Lianghekou; LINE: long interspersed nuclear elements; Mb: megabase pairs; Mya: million years ago; NCBI: National Center for Biotechnology Information; QTP: Qinghai-Tibetan Plateau; PacBio: Pacific Biosciences; PAML: Phylogenetic Analysis by Maximum Likelihood; PCA: principal component analysis; PSG: positively selected genes; PSMC: Pairwise Sequentially Markovian Coalescent; RAxML: Randomized Axelerated Maximum Likelihood; SINE: short interspersed nuclear elements; SNP: single-nucleotide polymorphism; SRA: Sequence Read Archive; XX: Xixi.

## Ethics Statement

All experimental protocols were approved by the School of Life Sciences, Southwest University (Chongqing, China), and the studies were carried out in accordance with the Guidelines of Experimental Animal Welfare from Ministry of Science and Technology of People's Republic of China (2006) and the Institutional Animal Care and Use Committee protocols from Southwest University (2007).

## Competing Interests

The authors declare that they have no competing interests.

## Authors' Contributions

Z.W. conceived and designed the study; D.Y., X.C. and H.G. collected the samples; D.Y and S.X. performed molecular experiments; S.X., M.Z., Y.Z. and J.F. performed the bioinformatics analysis, including genome size estimation, genome assembly, annotation, and gene prediction; D.Y., S.X., and Z.W. wrote the manuscript. W.T. and X.D. revised the manuscript. All authors read and approved the final manuscript for submission.

## Supplementary Material

giaa132_GIGA-D-20-00124_Original_Submission

giaa132_GIGA-D-20-00124_Revision_1

giaa132_GIGA-D-20-00124_Revision_2

giaa132_Response_to_Reviewer_Comments_Original_Submission

giaa132_Response_to_Reviewer_Comments_Revision_1

giaa132_Reviewer_1_Report_Original_Submission Anti VasemÃ¤gi -- 6/5/2020 Reviewed

giaa132_Reviewer_1_Report_Revision_1 Anti VasemÃ¤gi -- 10/8/2020 Reviewed

giaa132_Reviewer_2_Report_Original_Submission Alan Le Moan -- 6/17/2020 Reviewed

giaa132_Reviewer_3_Report_Original_Submission Paolo Franchini -- 6/18/2020 Reviewed

giaa132_Reviewer_3_Report_Revision_1 Paolo Franchini -- 10/4/2020 Reviewed

giaa132_Supplemental_Files
