# Supplementary material for: Chromosomal genome of Triplophysa bleekeri provides insights into its evolution and environmental adaptation
Source: Gigascience. 2020 Nov 24;9(11):giaa132. doi: 10.1093/gigascience/giaa132 (PMC7684707; doi:10.1093/gigascience/giaa132)
Supplement: giaa132_GIGA-D-20-00124_Original_Submission [file giaa132_giga-d-20-00124_original_submission.pdf]

GigaScience

Chromosomal genome of Triplophysa bleekeri provides insights into its evolution and environmental adaptation

--Manuscript Draft--

**Manuscript Number:**	GIGA-D-20-00124
**Full Title:**	Chromosomal genome of Triplophysa bleekeri provides insights into its evolution and environmental adaptation
**Article Type:**	Research
**Funding Information:**	Financial Program of Ministry of Agriculture and Rural Affairs of China (YYJZHC201921301350063) — Dr. Zhijian Wang<br>National Natural Science Foundation of China (31602207) — Dr. Shijun Xiao<br>Research Innovation Program for College Graduates of Chongqing (CYB19079) — Dr. Dengyue Yuan
**Abstract:**	The uplift of the Qinghai-Tibetan Plateau (QTP) resulted in profound climatic changes in both QTP and its adjacent regions. These environmental changes induced strong stresses for several organisms, especially fish, since fish are strictly constrained by the living conditions. Triplophysa bleekeri (T. bleekeri), an endemic highland fish, presents an excellent model to investigate the genetic mechanisms of adaptation to the local environment. Here, we assembled a chromosomal genome sequence of about 628 Mb with contig and scaffold N50 of 3.1 and 22.9 Mb, respectively. We investigated the evolution and environment adaptation of T. bleekeri based on 21,198 protein-coding genes in the genome. Phylogenetic analysis showed that T. bleekeri diverged 38.8 and 28.6 million years ago (Ma) from their common ancestors of Triplophysa siluroides and Triplophysa tibetana , respectively. Compared to fish species living at low altitudes, gene families associated with lipid metabolism, necroptosis, and immune response were significantly expanded in the T. bleekeri genome, and genes involved in DNA repair and protein digestion underwent strong natural positive selections for T. bleekeri , T. siluroides and T. tibetana . We also analyzed whole-genome variants among samples from populations, and showed that populations separated by geological and artificial barriers exhibited obvious different genetic structures, which can be attributed to the disrupted gene exchanges among those populations. The chromosomal genome and population data provided valuable genetic resources for the following evolutionary and environmental adaptation investigations for Triplophysa and other high-land fish species.
**Corresponding Author:**	Zhijian Wang<br>Southwest University<br>Chongqing, China CHINA
**Corresponding Author Secondary Information:**	
**Corresponding Author's Institution:**	Southwest University
**Corresponding Author's Secondary Institution:**	
**First Author:**	Dengyue Yuan
**First Author Secondary Information:**	
**Order of Authors:**	Dengyue Yuan<br>Xuehui Chen<br>Haoran Gu<br>Ming Zou<br>Yu Zou

Powered by Editorial Manager® and ProduXion Manager® from Aries Systems Corporation

	Jian Fang
	Wenjing Tao
	Xiangyan Dai
	Shijun Xiao
	Zhijian Wang
**Order of Authors Secondary Information:**	
**Additional Information:**	
**Question**	**Response**
Are you submitting this manuscript to a special series or article collection?	No
**Experimental design and statistics**<br><br>Full details of the experimental design and statistical methods used should be given in the Methods section, as detailed in our Minimum Standards Reporting Checklist. Information essential to interpreting the data presented should be made available in the figure legends.<br><br>Have you included all the information requested in your manuscript?	Yes
**Resources**<br><br>A description of all resources used, including antibodies, cell lines, animals and software tools, with enough information to allow them to be uniquely identified, should be included in the Methods section. Authors are strongly encouraged to cite Research Resource Identifiers (RRIDs) for antibodies, model organisms and tools, where possible.<br><br>Have you included the information requested as detailed in our Minimum Standards Reporting Checklist?	Yes
**Availability of data and materials**<br><br>All datasets and code on which the conclusions of the paper rely must be	Yes

either included in your submission or deposited in publicly available repositories (where available and ethically appropriate), referencing such data using a unique identifier in the references and in the "Availability of Data and Materials" section of your manuscript.<br><br>Have you have met the above requirement as detailed in our Minimum Standards Reporting Checklist?	

Manuscript
Click here to download Manuscript Manuscript-Triplophysa bleekeri.docx

Chromosomal genome of *Triplophysa bleekeri* provides insights into its evolution and environmental adaptation

Dengyue Yuan[1], Xuehui Chen[1], Haoran Gu[1], Ming Zou[2], Yu Zou[2], Jian Fang[2], Wenjing Tao[1], Xiangyan Dai[1], Shijun Xiao[2,3,*], Zhijian Wang[1,*]

[1] Key Laboratory of Freshwater Fish Reproduction and Development (Ministry of Education), Key Laboratory of Aquatic Science of Chongqing, School of Life Sciences, Southwest University, Chongqing 400715, China

[2] School of Computer Science and Technology, Wuhan University of Technology, Wuhan, Hubei 430000, China

[3] College of Plant Protection, Jilin Agriculture University, Changchun, Jilin 130118, China

* Correspondence to Prof. Dr. Zhijian Wang (wangzj1969@126.com) and Dr. Shijun Xiao (shijun_xiao@163.com)

Abstract

The uplift of the Qinghai-Tibetan Plateau (QTP) resulted in profound climatic changes in both QTP and its adjacent regions. These environmental changes induced strong stresses for several organisms, especially fish, since fish are strictly constrained by the living conditions. *Triplophysa bleekeri* (*T. bleekeri*), an endemic highland fish, presents an excellent model to investigate the genetic mechanisms of adaptation to the local environment. Here, we assembled a chromosomal genome sequence of about 628 Mb with contig and scaffold N50 of 3.1 and 22.9 Mb, respectively. We investigated the evolution and environment adaptation of *T. bleekeri* based on 21,198 protein-coding genes in the genome. Phylogenetic analysis showed that *T. bleekeri* diverged 38.8 and 28.6 million years ago (Ma) from their common ancestors of *Triplophysa siluroides* and *Triplophysa tibetana*, respectively. Compared to fish species living at low altitudes, gene families associated with lipid metabolism, necroptosis, and immune response were significantly expanded in the *T. bleekeri* genome, and genes involved in DNA repair and protein digestion underwent strong natural positive selections for *T. bleekeri*, *T. siluroides* and *T. tibetana*. We also analyzed whole-genome variants among samples from populations, and showed that populations separated by geological and artificial barriers exhibited obvious different genetic structures, which can be attributed to the disrupted gene exchanges among those populations. The chromosomal genome and population data provided valuable genetic resources for the following evolutionary and environmental adaptation investigations for *Triplophysa* and other high-land fish species.

Keywords: *Triplophysa bleekeri*, genome, adaptation, population

Introduction

The Qinghai-Tibetan Plateau (QTP), the largest and highest plateau in the world, is one of the most important world biodiversity centers (Myers et al., 2000). The environments of QTP and its adjacent areas were affected significantly by the continuing uplifts, which is one of the most important driving forces for biological evolution of organisms on the QTP (Zhao and Li, 2017). The endemic species of the QTP present a great adaptability to the harsh environments of low temperature and low oxygen (Beall, 2014).

An investigation into the biological evolution of organisms residing on the QTP would widen our understanding of essential evolutionary questions regarding mechanisms of environmental adaptation and speciation for organisms. Phenotype comparisons were frequently used to study environmental adaptations in previous studies (Deng et al., 2010; Ding et al., 2016). In recent years, advancing genomic technology, especially third generation sequencing techniques, has presented novel opportunities to explore the genetic basis of environmental adaptations. Many genomic studies of terrestrial animals on the QTP revealed that genes involved in hypoxia response, energy metabolism, and DNA repair were under positive selection and rapid evolution (Li et al., 2018a; Li et al., 2013; Qiu et al., 2012). In those studies, high-quality genome and population resources are essential to understand important biological processes for adaptations (Li et al., 2018a; Liu et al., 2016; Sun et al., 2018).

The QTP boasts many highland fish species, especially in the family Sisoridae, subfamily Schizothoracinae, and genus *Triplophysa* (Wu and Tan, 1991). To date, there have only been four high-quality highland fish genomes reported, based on long-read sequencing data, including *Glyptosternon maculatum* in the family Sisoridae, *Oxygymnocypris stewartii* in the subfamily Schizothoracinae, and *Triplophysa tibetana* and *Triplophysa siluroides* in the genus *Triplophysa* (Liu et al., 2018; Liu et al., 2019; Yang et al., 2019a; Yang et al., 2019b). *Triplophysa* is a strongly diverged species and the largest group of the subfamily Nemacheilinae (Nelson et al., 2016). There are 152 records for *Triplophysa* species in FishBase, and the majority are distributed on the QTP and its adjacent drainages from an elevation of 100 m to over 5,200 m (He et al., 2011). Due to the board elevation distributions, *Triplophysa* species are thought to present an excellent opportunity to explore evolution and environmental adaptation. However, the environmental adaptation mechanism of *Triplophysa* species is far from fully understood and the genetic resource for the reference genome and population data remains insufficient.

Triplophysa bleekeri (*T. bleekeri*), a typical species of *Triplophysa* family, is mainly distributed in the stem streams and tributaries of the Yangtze River and Jinsha River (He et al., 1999). Although *T. siluroides*, *Trilophysa bleekeri* and *Triplophysa tibetana* are fish in the *Trilophysa* genus, they exhibit distinct different ecological niches on the QTP and peripheral regions, leading to various physiological characters. *T. tibetana* occurs at elevations from 4,000 ~ 5,000 m, while *T. siluroides* lives at altitudes from 3,000 ~ 4,000 m (Yang et al., 2019b; Zhu, 1989). Compared to *T.*

tibetana and *T. siluroides*, *T. bleekeri* has a wide distribution from 200 m to 3,000 m (Wang et al., 2013). Apart from the living altitude, there was a significant difference in habitat environments. *T. tibetana* and *T. siluroides* inhabit in river and lake with slow flow, whereas *T. bleekeri* just lives in the fast-flowing river (Wu, 1991). Meanwhile, the reproduction biology for *Trilophysa* fishes is also different that the breeding season for *T. tibetana* is June to July, and *T. siluroides* is July to August. But *T. bleekeri* can spawn twice a year, and the peak seasons of breeding are October to December and March to April (Wang et al., 2013). The *Triplophysa* fishes also exhibit noticeable differences in feeding habits and morphologic characteristics. The primary foods of *T. bleekeri* and *T. tibetana* are chironomus larva, caddis fly larvae, and diatom, but *T. siluroides* feeding more on small fishes (Zhu, 1989). Therefore, although genomes for *T. siluroides* and *T. tibetana* has been reported before, genome resource for *T. bleekeri* is still crucial in environmental adaptation and evolution studies for *Triplophysa* fishes. In this work, we generated the first chromosomal genome sequence of *T. bleekeri* using the combined technology of the Illumina, PacBio, and Hi-C platforms. Based on these genome data, we studied the evolutionary relationship with closely-related species. We identified gene families under expansion, as well as positively selected genes (PSGs), and attempted to identify critical functional genes of *T. bleekeri* contributed to its adaptability. The population genetics of *T. bleekeri* were also investigated to reveal the genetic structures among different populations. Those relevant genomic data provide crucial resources for biological and evolutionary studies. This work also investigated the adaptive mechanism of highland fish in the cold environment and

demonstrates the geological and artificial barriers might influence the genetic structures of *T. bleekeri* populations.

Materials and Methods

Samples and tissue collection

T. bleekeri (**Fig.1**) samples were obtained from the Daning River (31°09′26.58″N, 109°53′31.68″E) (**Fig.2**), which is a tributary in the upper reaches of the Yangtze River. Fish were then transferred to the Aquaculture Laboratory of Southwest University, and reared in indoor tanks. To collect enough tissues for the genome and transcriptome sequencing, the largest female individual was used for the library construction and sequencing. The fish was anesthetized with tricaine MS-222 and was immediately dissected. Meanwhile, 12 tissue types were collected, including brain, eye, skin, gill, heart, liver, trunk kidney, spleen, gut, muscle, gallbladder, and gonad. Tissues were quickly frozen in liquid nitrogen for more than one hour, and then stored at −80℃. Among these tissues, muscle tissue was used for genomic DNA sequencing and Hi-C library construction. Meanwhile, all tissue samples were used in the application of transcriptome sequencing to obtain a comprehensive transcript. To understand the population structures of the species, 28 individuals, including eleven, seven, and six samples collected from population 1, 2, and 3 (**Fig. 2**), were sacrificed and muscle tissues were collected as aforementioned.

Genome DNA extraction and sequencing library construction

DNA molecules were extracted from muscle tissue using the phenol-chloroform DNA extraction method (Xiao et al., 2016). The Qubit (Thermo Fisher Scientific,

Waltham, MA, USA) and Agilent Bioanalyzer 2100 (Agilent Technologies, Palo Alto, CA, USA) was used for evaluating the quantity and quality of DNA. For sequencing based on the Illumina Hiseq technology, a short-read sequencing library with an insert size of 250 bp was constructed using 1 μg of DNA. For sequencing on the PacBio SEQUEL platform (Pacific Biosciences of California, Menlo Park, CA, USA), the DNA molecules from muscle tissue were also used to construct the long-read sequencing library for PacBio platform. Briefly, 10 μg of *T. bleekeri* genomic DNA were used for 20-kb library preparation following the manufacturer's protocol (Pacific Biosciences), and the BluePippin Size Selection system (Sage Science, Beverly, MA, USA) was used for library size selection. DNA molecules from the largest individual were sequenced using the PacBio and Illumina platform for genome assembly and other samples were subject to the short-read whole-genome resequencing on the Illumina platform.

RNA extraction and sequencing library construction

RNA sequencing data provides most important evidence for gene prediction in the genome (Denoeud et al., 2008). To include as many expressed genes as possible, 12 tissue types, mentioned above, were used for the RNA sequencing library construction. RNA was isolated from the 12 tissue samples using TRIZOL reagent (Invitrogen, USA). The quantity and quality of extracted RNA were determined using the Nanodrop ND-1000 spectrophotometer (LabTech, Holliston, MA, USA) and 2100 Bioanalyzer (Agilent Technologies, Palo Alto, CA, USA). Samples with a total RNA concentration ≥ 10 μg and RNA integrity number ≥ 8 were used for sequencing

experiments. RNA molecules extracted from tissues were equally mixed for the following RNA library construction. RNA sequence library was constructed under the guidance of protocol of the Paired-End Sample Preparation Kit (Illumina Inc., San Diego, CA, USA), which was identical to that employed in our previous study (Xiao et al., 2015).

DNA and RNA library sequencing

The short-read DNA and RNA sequencing libraries were sequenced with the 150 bp paired-end (150PE) mode using the Illumina HiSeq X Ten platform (Illumina Inc.). The 20kb long-read genome DNA SMRT bell libraries sequencing library was sequenced with the PacBio SEQUEL platform (Pacific Biosciences). The raw sequencing data was quality checked before the bioinformatics analysis. The HTQC package (Yang et al., 2013) was used to filter low-quality bases and reads, and the sequences with adapters or low quality (average quality score < 20) were removed.

Genome size estimation

The genome size was estimated based on Illumina sequencing data using the *K*mer method before genome assembly. Raw Illumina reads were processed to remove adapter sequences, the reads with more than 10% N bases and the reads with more than 50% low quality bases (≤ 5). All filtered reads were used for *K*mer frequency analysis (Liu et al., 2013). Using *K*mer size of 17, the *K*mer frequencies were obtained using jellyfish (Marcais and Kingsford, 2011). *K*mers with a frequency of lower than 3 were eliminated as those likely resulted from sequencing errors. The genomic size was estimated based on the following formula: $G = (L - K + 1) \times n_{base}/$

(C_{Kmer} × L), in which G is the estimated genome size, n_{base} is the total count of bases, C_{Kmer} is the expectation of *K*mer depth, L indicates the read length, and K represents *K*mer size. The calculated genome size required further revision, since *K*mers with a depth lower than three likely resulted from sequencing errors. The Revised Genome size = Genome size × (1-Error Rate).

***De novo* assembly of the *T. bleekeri* genome**

Long reads generated from the PacBio sequencing platform were used for T. bleekeri genome assembly with the Falcon package (Chin et al., 2016). The assembled genome sequences were further polished with Arrow using long-read sequencing (Chin et al., 2013); thereafter, two rounds of polishing using NGS short reads were performed with Pilon (Walker et al., 2014). Finally, redundant genomic sequences were eliminated using Redundans with the parameter overlap of 0.95 and identity of 0.95 (Pryszcz and Gabaldón, 2016). The completeness of the assembled genome was evaluated using BUSCO version 3.0 (Simão et al., 2015).

Chromosome assembly using Hi-C technology

Muscle tissue (1 g) was collected from the above fish for PacBio sequencing and was used for Hi-C library construction. The Hi-C processes, including crosslinking, lysis, chromatin digestion, biotin marking, proximity ligations, crosslinking reversal, and DNA purification, were performed using the protocol described in as previous studies (Gong et al., 2018). The purified and enriched DNA was used for sequencing library construction. The library was sequenced using the Illumina HiSeq X Ten platform (Illumina), and the short-reads were then mapped to the polished genome of

T. bleekeri with Bowtie 1.2.2. The chromosomal assembly using interaction frequency matrix extracted from the Hi-C read mapping were performed according to a previously-reported methodology (Gong et al., 2018).

Repetitive element annotation

The *de novo* prediction and homology prediction were combined to annotate the repetitive sequences in the *T. bleekeri* genome. RepeatModeler (http://www.repeatmasker.org/RepeatModeler.html) was used for the *de novo* repetitive elements detection in the *T. bleekeri* genome. The detected genome repeats were combined with repBase library (Jurka et al., 2005), as a comprehensive library for the final repetitive elements prediction in the *T. bleekeri* genome, using the Repeat Masker software (Tarailo- Graovac and Chen, 2009). Transposons were predicted using the ProteinMask and the tandem repeats were identified in the genome using Tandem Repeat Finder (Benson, 1999).

Protein coding and non-coding gene prediction

The *de novo* prediction, homology prediction, and RNA-sequencing-based methods were used for protein-coding gene annotation. Gene models for protein-coding genes were first predicted in the *T. bleekeri* genome using Augustus (Stanke et al., 2006). Five closely related fish species, including *Cyprinus carpio* (*C. carpio*), *Danio rerio* (*D. rerio*), *Oryzias latipes* (*O. latipes*), *Tetraodon nigroviridis* (*T. nigroviridis*), and *Xiphophorus maculatus* (*X. maculatus*), were used for the Homolog-based protein-coding gene prediction. The public protein sequences from those species were mapped to the genome using the TBLASTN utility (Lobo, 2008)

and GeneWise (Birney et al., 2004). Thereafter, comprehensive transcriptome sequencing data for multi-tissues were aligned to the genome, and gene models were generated using the TopHat package (Trapnell et al., 2009) and Cufflinks (Ghosh and Chan, 2016). The integration and redundancy elimination for the gene models predicted using the above methods were performed using the MAKER package (Campbell et al., 2014; Cantarel et al., 2008). Four types of non-coding RNAs, including microRNAs (miRNA), transfer RNAs (tRNA), ribosomal RNAs (rRNA), and small nuclear RNAs (snRNA), were also predicted in the *T. bleekeri* genome using tRNAscan-SE (Lowe and Eddy, 1997) and using Infernal (Nawrocki and Eddy, 2013) with the Rfam database (Griffithsjones et al., 2003).

Functional annotation of protein-coding genes

The NCBI non-redundant protein (Booke and E., 2011), Swissport, and TrEMBL databases (Boeckmann et al., 2003) were used as protein databases for the biological function annotation using BLAST packages (McGinnis and Madden, 2004). The E-value of 1e-5 was used as the threshold for homolog identification. Gene Ontology (GO) (Harris et al., 2004) and the Kyoto Encyclopedia of Genes and Genomes (KEGG) (Ogata et al., 2000) assignments were performed using Blast2GO software (Conesa et al., 2005).

Gene family clustering and phylogenetic analysis

Coding sequences annotated from whole genome sequences for the closely related species were extracted from genome sequences. Gene family clustering was performed for *T. bleekeri* with those fish species living in non-QTP regions, including

Callorhinchus milii (*C. milii*), *Lepisosteus oculatus* (*L. oculatus*), *Gadus morhua* (*G. morhua*), *D. rerio, O. latipes, X. maculatus, Takifugu rubripes* (*T. rubripes*), *T. nigroviridis, Larimichthys corcea* (*L. corcea*), *Gasterosteus aculeatus* (*G. aculeatus*) by the Orthomcl pipeline (Li et al., 2003) with default settings. The single-copy orthologs across all species were selected for gene family, phylogenetic, and evolutionary analysis. Briefly, proteins of these genes were aligned with muscle (Edgar, 2004) and were then transformed into alignments of nucleotide sequences with pal2nal (Suyama et al., 2006) on the basis of the corresponding coding sequences. Next, non-conservative regions were removed using Gblocks (Castresana, 2000) with default settings and the conservative regions were concatenated and fed in Raxml (Stamatakis, 2014) to deduce the phylogenetic relationships of these species. Rapid bootstrap runs (100 times) were performed to test robustness of the topology (Stamatakis et al., 2008). Based on the topology and the alignment matrix, their divergence times were deduced using MCMCTREE included in the PAML package (Yang, 2007) with calibration points set by consulting the TimeTree database.

Gene family expansion and contraction in the *T. bleekeri* genome

To identify expanded and contracted gene families in the *T. bleekeri* genome, we compared gene families in the *T. bleekeri* genome to *T. tibetana, T. siluroides, and* those fish species living in non-QTP regions, as in gene family clustering analysis. CAFÉ (De Bie et al., 2006) was used to analyze the expansion and contraction of gene clusters in the *T. bleekeri* genome using a probabilistic model. A GO enrichment analysis was performed on expanded and contracted genes using topGO package

(Alexa and Rahnenfuhrer, 2010). The enrichment of genes in KEGG pathways was also analyzed using the KOBAS (Xie et al., 2011).

Positively selected genes in the *T. bleekeri* genome

MUSCLE was used for multi-protein sequence alignment among the *T. bleekeri* genes and their orthologs, comparing to the fish species living in non-QTP regions used in the gene family clustering. Conserved CDS alignments of each single-copy gene family were extracted using Gblocks (Talavera and Castresana, 2007) and used for further identification of PSGs. The ratios of nonsynonymous to synonymous substitutions (K_A/K_S, or ω) were estimated for each single-copy orthologous gene using the CodeML program with the branch-site model as implemented in the PAML package. A likelihood ratio test was conducted, and the false discovery rate (FDR) correction was performed for multiple comparisons. Genes with a corrected P value < 0.05 were defined as PSGs. To compare the natural selected genes for *T. tibetana* and *T. siluroides*, the natural selected genes were also identified using the identical method. The functional annotation of PSGs for *T. bleekeri*, *T. tibetana* and *T. siluroides* was also conducted using the same approach with the gene family expansion and contraction analysis.

Evolution relationship and divergence time for *T. bleekeri* with closely-related fish species

To investigate the evolutionary relationship of *T. bleekeri* to closely-related fish species, we added another four *Triplophysa* genus fish species to the phylogenetic analysis. Since the genome of *T. xichangensis* and *T. scleroptera* have not been

reported, we downloaded the short-reads of the transcriptomes of those two species from the NCBI sequence read archive (SRA) and conducted *de novo* assembly using Trinity (Grabherr et al., 2011) with default settings. The assembled transcriptome sequences were also used in the analysis. The single-copy orthologs across all species were used for phylogenetic tree reconstruction and divergence time estimation.

Genetic structures for *T. bleekeri* populations

Raw reads of samples subject to resequencing were quality controlled as aforementioned. Thereafter, the filtered short reads were mapped using bowtie2 (Langmead and Salzberg, 2012) with default settings for each individual, followed by the marking duplicates with Picard. Regions near INDELs were thought to be poorly aligned and were identified and realigned using GATK (Mckenna et al., 2010). GATK was also used to call SNPs and INDELs based on the alignments. The identified SNPs were filtered using SNPhylo (Lee et al., 2014) v20180901 with default settings, except the LD_threshold and Minimum_depth_of_coverage were set to 0.8 and 3, respectively. Next, the PCA clusters and population structure for these individuals were deduced with plink v1.9 (Purcell et al., 2007) and Admixture (Alexander et al., 2009; Tamura et al., 2007) with default settings, respectively. Their phylogenetic relationships were recovered using the neighbor-joining method with MEGA4 (Tamura et al., 2007) and bootstrap resampling (100 times) was performed to test the topology robust.

Results

DNA and RNA library sequencing

81.69 Gb genomic (~120X) and 10.6 Gb transcriptome short-reads were generated for the following genome size estimation and annotation (**Table 1**). Meanwhile, we also obtained 100.87 Gb genomic long-reads from the PacBio platform, with a rough coverage of 160X for the *T. bleekeri* genome (**Table 1**). The mean and N50 length of the long-reads were 5.8 kb and 16 kb, respectively (**Table 1** and **Supplementary Fig. S1**).

Genome size estimation

To determine the possible sample contamination, 10,000 NGS short-reads were randomly selected for an NCBI nt database search. *Cyprinus*, *Danio*, and *Sinocyclocheilus* represent the top three sources of best hits, ruling out the obvious contamination during library construction and sequencing. Using genomic short-reads generated from the Illumina platform, a total of 59.8 million *K*mers were generated. The genome of *T. bleekeri* was estimated as 632.5 Mb with a heterozygosity ratio of 0.26% and repeat content of 42.2% (**Fig. 3**). Based on the above genome character estimation, the genome of *T. bleekeri* was mid-sized with low heterozygosity.

***De novo* assembly of the *T. bleekeri* genome**

Using genomic PacBio long-reads for *T. bleekeri*, we assembled a 628 Mb genome with 856 contigs and an N50 length of 3.82 Mb. Among these contigs, the longest contig for the genome was 15.5 Mb (**Table 2**). The completeness of the assembled genome was evaluated using BUSCO v3.0 (Simão et al., 2015) with the actinopterygii_odb9 database, indicating that 92.9% of BUSCO genes were identified in the assembled genome (**Supplementary Fig. S2**).

Chromosome assembly using Hi-C technology

Hi-C technology recruits interaction information among different chromosome regions and assumes that the interactions for nearby regions are more prevalent than distant regions. In this study, a total of 82.9 Gb sequencing data were obtained via Hi-C library sequencing. Based on the interacting information, a chromosome assembly of 628 Mb with a scaffold N50 length of 22.9 Mb was obtained (**Supplementary Fig. S3**). More than 596.9 Mb sequences were anchored upon 25 chromosomes, highlighting a high chromosome anchoring rate of 96.2% on the base level.

Repetitive element annotation

The annotation pipeline showed that more than 17.9 Mb of the genome sequences were predicted as tandem repeats, covering about 2.8% of the genome, and finally a total of 203.2 Mb, accounting for roughly 32.4% of the genome, were annotated as repetitive elements in the *T. bleekeri* genome (**Supplementary Table S1**). Specifically, there are 17.2% DNA transposons (107.8 Mb), 5.8% of long interspersed nuclear elements (LINE) (36.4 Mb), 0.68% short interspersed nuclear elements (SINE) (4.3 Mb), and 6.93% long terminal repeats (LTR) (43.5 Mb).

Protein and non-coding gene prediction and functional annotation

For predicting protein-coding genes in the *de novo* assembled genome, a total of 10.6 Gb short-read transcriptome data were generate from 12 tissues. Based on the *de novo*, homolog, and RNA-seq data methods, a total of 20,274, 27,243, and 15,875 protein-coding genes were predicted, respectively. After integration and redundancy

elimination, 21,198 protein-coding genes were predicted in the *T. bleekeri* genome (**Supplementary Table S2**).

Of the 21,198 protein-coding genes, roughly 93.0%, 96.9%, and 90.9% displayed homologous sequences in the NCBI NR, TrEMBL and Swissprot databases, respectively. Additionally, 89.2% contained InterPro domains, and 46.9% were assigned with GO terms. Overall, more than 97.3% of the protein-coding genes were functional annotated by at least one method (**Supplementary Fig. S4**). Meanwhile, the important function of the non-coding genes received further attention. Based on the *de novo* prediction strategies, all the possible non-coding gene loci were predicted, and the summary is listed in **Supplementary Table S3**.

Gene family expansion and contraction in the *T. bleekeri* genome

Following the Orthomcl pipeline, a total of 21,862 ortholog groups were obtained after gene family clustering with those fish species from non-QTP regions. A total of 1,533 significantly expanded and 2,401 significantly contracted gene families were observed for *T. bleekeri*, compared to other fish species (**Supplementary Fig. S5**). The functional enrichment of expanded gene families using was analyzed using GO and KEGG. The expanded gene families were primarily enriched in categories of metabolism and immune regulation (**Supplementary Tables S4** and **S5**). The category of metabolism includes fatty acid metabolism (arachidonic acid metabolism and glycosphingolipid biosynthesis), carbohydrate metabolism (glycosaminoglycan biosynthesis and glycan degradation), and amino acid metabolism (RNA transport). The category of immune regulation includes the Hippo signaling pathway (corrected *p*

value = 2.40E-03), necroptosis, and Vitamin B6 metabolism (corrected *p* value= 8.90E-03). The contracted gene families were mainly made up of several signaling pathways, including the MAPK signaling pathway, calcium signaling pathway, adrenergic signaling in cardiomyocytes, GnRH signaling pathway, and retrograde endocannabinoid signaling.

T. bleekeri **genes under natural positive selection**

We identified 788 positive selected genes (PSG) in the *T. bleekeri* genome. The functional analysis on the KEGG and GO terms showed that several categories associated with nucleotide metabolism and DNA repairs, including single strand break repair, DNA repair, base excision repair, were significantly enriched (**Supplementary Table S6**). Additionally, the PSGs were also enriched in biological pathways of non-homologous end-joining, fanconi anemia pathway, pyruvate metabolism, meiosis (**Supplementary Table S7)**. Meanwhile, 969 and 1,253 PSGs were identified for *T. tibetana* and *T. siluroides,* respectively. Among those genes, 197 genes were identified as shared PSGs for three *Triplophysa* species (**Fig. 4a**), indicating those functional genes might be naturally selected in their common ancestor. The functional analysis with respect to biological pathways for the shared genes showed that those genes were significantly enriched on the protein-digestion, salivary secretion, fanconi anemia pathways (**Fig. 4b**).

Phylogenetic relationships of *T. tibetana* with other fish species

Using the genome and transcriptome of other *Triplophysa* species, including *T. tibetana*, *T. siluroides, T. scleroptera,* and *T. xichangensis*, we investigated the

evolutionary relationship of *T. bleekeri* with respect to other fish species. Using single copy genes among species, a concatenated alignment matrix, using those single copy orthologs, was generated with a total length of 73,887 bps, which was used for the phylogenetic analysis and divergence time estimation. The result showed that *Triplophysa* species formed a sister group with *D. rerio*. The *T. siluroides* was a basal species in the *Triplophysa* group, which was consistent with previous studies that *T. siluroides* was an ancient *Triplophysa* species. Divergence time estimation showed that *T. bleekeri* diverged from their common ancestor of *T. scleroptera* and *T. xichangensis* about 25.2 Million years ago (**Fig. 5**).

Population structure of *T. bleekeri*

The high-quality SNPs were obtained according to serious filtering criteria and were used to deduce the population structures of *T. bleekeri*. As a result, more than 34 million short-reads were obtained for 28 individuals and a total of 3,025,149 SNPs were detected for all individuals. The phylogeny reconstruction analyses based on whole-genome SNPs showed that individuals from population 1 and 2 formed a sister group, with all 11 individuals from population 1 forming a monophyletic group and nine individuals from population 2 forming another monophyletic group. Six individuals from population 3 formed the third monophyletic group (**Fig. 6**). Structure analysis also indicated that genes flow between population 3 and the other two populations are limited (**Supplementary Fig. S6**). In addition, the PCA clusters (**Supplementary Fig. S7**) suggested that the first two components could successfully separate the individuals in population 3 from those in population 1 and 2.

Discussion

The *Triplophysa* species compose the predominant fish taxon in the drainages of the QTP. The wide range of altitudes for their distribution makes the species an excellent model for genome evolution and adaption studies. The genomic resource of *T. bleekeri* would not only help us to better understand its biological evolution and adaptation mechanisms, but also provide valuable conservation genetics data for species protection.

In this study, we presented the chromosome-level genome assembly of *T. bleekeri* with contig N50 of 3.82 Mb. The N50 lengths of contigs of *T. bleekeri* genome assembly were much longer than previously reported genome assemblies of *T. tibetana* (Yang et al., 2019b). The completeness and chromosome anchoring ratio of the genome were also evaluated, confirming the high quality of the *T. bleekeri* genome. The combined results of the homology-based and *de novo* predictions showed that repetitive sequences account for 32.4% of the genome. Among them, DNA transposons represent the most abundant tandem repeats, which were similarly observed in other teleosts, such as *T. tibetana* (Yang et al., 2019b), *Epinephelus akaara* (*E. akaara*) (Ge et al., 2019), and *Epinephelus lanceolatus* (*E. lanceolatus*) (Zhou et al., 2019). Within the genome, a total of 21,198 protein-coding genes were predicted, of which 97.3% could be functionally annotated. Overall, this genome assembly and annotation can provide valuable data to the genomic resources currently available for the study of phylogeny and environmental adaptations of *Triplophysa* species.

The phylogeny results indicated that the *Triplophysa* family formed a clade with *D. rerio* and *T. bleekeri* was most closely related to *T. tibetana* and *T. xichangenis*. The divergence time estimation indicated that *T. siluroides* diverged from their common ancestor roughly 38.8 Ma, suggesting that *T. siluroides* is a more primitive species compare with other species in the *Triplophysa* genus. The extensive QTP was elevated exceeding 4,000 m about 40 Ma (Valdes et al., 2019), and this time is consistent with the divergence of *T. siluroides*. Therefore, we speculated that the speciation of *Triplophysa* was enormously influenced by uplifts of the QTP (Chang and Miao, 2016).

Uplift of the QTP induced profound impacts on climatic and environmental changes of the plateau and its adjacent regions, including low oxygen and low temperature (Li et al., 2014). In fact, an investigation into water quality indicated that high dissolved oxygen concentration exists in the QTP water (Li et al., 2010; Li et al., 2018b; Murakami et al., 2007; Wei, 2009). Therefore, we speculated that thermal stress may present a major factor in natural selection for fish species in the QTP. Although *Triplophysa* species are widely distributed in different regions, these regions are all generally characterized by a cold environment (Chen et al., 1996; Xiao and Dai, 2011). However, to the best of our knowledge, few studies have addressed the mechanism by which *Triplophysa* species adapt to low temperatures.

Through the comparative analysis of the genome with other fish species, we found the expanded gene families of *T. bleekeri* were significantly ($p < 0.05$) enriched in arachidonic acid metabolism pathways. Arachidonic acid, an integral constituent of

biological cell membranes, aids in the maintenance of cell membrane fluidity even at low temperatures (Hanna and Hafez, 2018). Results suggest that the increased number of genes related to arachidonic acid metabolism might be responsible for improving membrane fluidity under cold environments. Low temperatures could also induce the accumulation of reactive oxygen species (ROS) and ion leakage, causing cellular injuries and apoptosis, which eventually leads to cell death (Pirzadah et al., 2014). Indeed, we found that expanded gene families in the *T. bleekeri* genome were enriched in necroptosis and apoptosis, implying the biological requirement for *T. bleekeri* to live in cold environments. Genes were expanded in the notch signaling pathway, which is involved in regulating cell proliferation, cell differentiation, and cell death to maintain tissue self-renewal (Kopan and Ilagan, 2009). While ROS can injure the host cell, ROS play a central role in the development of the antimicrobial innate immune responses (Kohchi et al., 2009). Our results also showed that many genes in the hippo signaling pathway, an essential signaling pathway to regulate innate immunity, were significantly expanded. In addition, the naturally selected genes were also enriched in nucleotide excision repair, mismatch repair, and base excision repair. Low temperatures, oxidative damage, and UV radiation can cause DNA damage. Many naturally selected genes influencing DNA repair may contribute to DNA integrity and stability for the *T. bleekeri* genome under extreme environments. Interestingly, natural positively selected genes for three *Triplophysa* species were all significantly enriched on fanconi anemia pathway, non-homologous end-joining and homologous recombination (**Supplementary Table S7**), indicating that *Triplophysa*

species might be under similar natural selections. Meanwhile, we found 197 natural positively selected genes for three *Triplophysa* species, and those naturally selected genes might originate from their common ancestor or independent selections. We also indeed found massive genes that uniquely naturally selected for three *Triplophysa* species. The result might imply the requirement of the distinction of the ecological niche for *Triplophysa bleekeri*, *T. tibetana* and *T. siluroides*.

The relationships among populations of *T. bleekeri* were analyzed to probe possible differences in genetic structures. The results showed that samples of population 1 and 2 formed a sister group, while samples of population 3 formed the third monophyletic group. This result could be because population 1 and 2 are directly connected by the river and gene flow between individuals residing in the two places occur more frequently. The difference between population 3 and population 1 and 2 might be attributed to the relatively limited gene flow hampered by natural and artificial barriers among those populations. The Daning River flows through many narrower gorges, and river measures a height of up to 1,648 m (Chongqing Water Resources Bureau, 2017), therefore, the geographical barriers formed by canyons and shallows could contribute to diminished exchanges among those populations. More importantly, artificial barriers, such as cities and dams, could also weaken the migrations between population 3 and 1/2. This research serves as a preliminary population genetics analysis for *T. bleekeri*. Further research is required to determine how geographical and artificial barriers precisely influence gene exchange among populations.

Conclusion

The QTP is the highest plateau world-wide and is characterized by low temperatures and hypoxia environment. The *Triplophysa* species is widely distributed, up to an elevation of 4,500 m. It is fascinating to investigate the molecular mechanisms for their environmental adaptation to the QTP. By combing multiple sequencing platforms and using the Hi-C technique, here we reported the first chromosomal genome for *T. bleekeri*. We obtained a genome of 628 Mb for *T. bleekeri*, containing 856 contigs with an N50 length of 3.82 Mb, with the longest contig of 15.5 Mb. More than 96% of bases were anchored upon 25 chromosomes, resulting in a scaffold N50 length of 22.9 Mb. A total of 203.2 Mb accounting for roughly 32.4% of the genome were annotated as repetitive elements, and 21,198 protein-coding genes were obtained eventually, and more than 97.3% of these genes can be functionally annotated. Gene families that underwent significant expansion and positive selected genes were enriched in pathways related to lipid metabolism, DNA repair, and immune response, implying the molecular requirements for adaptation of *T. bleekeri* to low temperatures and ultraviolet radiation. Population analyses suggested that genetic structures among *T. bleekeri* populations may be influenced by both geographical and artificial barriers. The genomic resource generated in this work and the analysis based on those data lay a solid foundation for further evolutionary environmental adaptation and conservation studies for *Triplophysa* and other highland fish species. However, fish are the most diversified group of vertebrates with enormous variation in their habitats and ecological niches as well as life history (Ravi and Venkatesh, 2008; Ronnestad et al., 2017). Therefore, more genomic data of high

land fish are needed to better understand their adaptive mechanism to the local environment.

Ethics Statement

All experimental protocols were approved by the School of Life Sciences, Southwest University (Chongqing, China), and the studies were carried out in accordance with the Guidelines of Experimental Animal Welfare from Ministry of Science and Technology of People's Republic of China (2006) and the Institutional Animal Care and Use Committee protocols from Southwest University (2007).

Acknowledgement

This work was supported by the Financial Program of Ministry of Agriculture and Rural Affairs of China (Grant No. YYJZHC201921301350063), National Natural Science Foundation of China (Grant No. 31602207), and Research Innovation Program for College Graduates of Chongqing (Grant No. CYB19079).

Author contributions

ZJ Wang conceived and designed the study; DY Yuan and SJ Xiao collected the samples; DY Yuan and SJ Xiao performed molecular experiments; SJ Xiao performed the bioinformatics analysis, including genome size estimation, genome assembly, annotation, and gene prediction; DY Yuan, SJ Xiao, and ZJ Wang wrote the manuscript. All authors read and approved the final manuscript for submission.

Competing interests

All authors declare that they have no competing interests.

Data accessibility

The genomic, transcriptome, and Hi-C sequencing reads generated from the PacBio and Illumina platforms are available in the NCBI SRA database under the Accession no. SRP200140. The final chromosome assembly was submitted to NCBI with the accession number of VFQW00000000.

References

Alexa, A., and Rahnenfuhrer, J. (2010). topGO: enrichment analysis for gene ontology. R package version *2*, 2010.

Alexander, D.H., Novembre, J., and Lange, K. (2009). Fast model-based estimation of ancestry in unrelated individuals. Genome Research *19*, 1655-1664.

Beall, C.M. (2014). Adaptation to high altitude: phenotypes and genotypes. Annual Review of Anthropology *43*, 251-272.

Benson, G. (1999). Tandem repeats finder: a program to analyze DNA sequences. Nucleic Acids Research *27*, 573.

Birney, E., Clamp, M., and Durbin, R.J. (2004). GeneWise and Genomewise. Genome Research *14*, 988.

Boeckmann, B., Bairoch, A., Apweiler, R., Blatter, M.-C., Estreicher, A., Gasteiger, E., Martin, M.J., Michoud, K., O'donovan, C., and Phan, I. (2003). The SWISS-PROT protein knowledgebase and its supplement TrEMBL in 2003. Nucleic acids research *31*, 365-370.

Booke, and E., H. (2011). Cytotaxonomic Studies of the Coregonine Fishes of the Great Lakes, USA: DNA and Karyotype Analysis. Journal of the Fisheries Research Board of Canada *25*, 1667-1687.

Campbell, M.S., Holt, C., Moore, B., and Yandell, M. (2014). Genome Annotation and Curation

Using MAKER and MAKER-P., Vol 48 (Current Prorocols in Bioinformatics).

Cantarel, B.L., Korf, I., Robb, S.M., Parra, G., Ross, E., Moore, B., Holt, C., Sánchez, A.A., and Yandell, M. (2008). MAKER: an easy-to-use annotation pipeline designed for emerging model organism genomes. Genome Research *18*, 188-196.

Castresana, J. (2000). Selection of Conserved Blocks from Multiple Alignments for Their Use in Phylogenetic Analysis. Molecular Biology and Evolution *17*, 540-552.

Chang, M.-M., and Miao, D. (2016). Review of the Cenozoic fossil fishes from the Tibetan Plateau and their bearings on paleoenvironment. Chinese Science Bulletin *61*, 981-995.

Chen, Y., Chen, Y., and Liu, H. (1996). Studies on the position of the Qinghai-Xizang Plateau region in zoogeographic divisions and its eastern demarcation line. Acta Hydrobiologica Sinica *20*, 97-103.

Chin, C.S., Alexander, D.H., Marks, P., Klammer, A.A., Drake, J., Heiner, C., Clum, A., Copeland, A., Huddleston, J., and Eichler, E.E. (2013). Nonhybrid, finished microbial genome assemblies from long-read SMRT sequencing data. Nature methods *10*, 563.

Chin, C.S., Peluso, P., Sedlazeck, F.J., Nattestad, M., Concepcion, G.T., Clum, A., Dunn, C., O'Malley, R., Figueroa-Balderas, R., and Morales-Cruz, A. (2016). Phased diploid genome assembly with single-molecule real-time sequencing. Nature methods *13*, 1050.

Chongqing Water Resources Bureau, C. (2017). Daning River

Conesa, A., Götz, S., García-Gómez, J.M., Terol, J., Talón, M., and Robles, M. (2005). Blast2GO: a universal tool for annotation, visualization and analysis in functional genomics research. Bioinformatics *21*, 3674-3676.

De Bie, T., Cristianini, N., Demuth, J.P., and Hahn, M.W. (2006). CAFE: a computational tool for

the study of gene family evolution. Bioinformatics *22*, 1269-1271.

Deng, H., Yue, X., Chen, D., Tian, H., and Liu, S. (2010). Growth characteristics and feed habit of *Triplophysa stenura* in Nujiang River. Freshwater Fisheries *40*, 26-33.

Denoeud, F., Aury, J.-M., Da Silva, C., Noel, B., Rogier, O., Delledonne, M., Morgante, M., Valle, G., Wincker, P., Scarpelli, C., *et al.* (2008). Annotating genomes with massive-scale RNA sequencing. Genome biology *9*, R175.

Ding, C.Z., Jiang, X.M., Chen, L., Juan, T., and Chen, Z. (2016). Growth variation of *Schizothorax dulongensis* Huang, 1985 along altitudinal gradients: implications for the Tibetan Plateau fishes under climate change. Journal of Applied Ichthyology *32*, 729-733.

Edgar, R.C. (2004). MUSCLE: multiple sequence alignment with high accuracy and high throughput. Nucleic Acids Research *32*, 1792-1797.

Ge, H., Lin, K., Shen, M., Wu, S., Wang, Y., Zhang, Z., Wang, Z., Zhang, Y., Huang, Z., and Zhou, C. (2019). De novo assembly of a chromosome‐ level reference genome of red‐ spotted grouper (*Epinephelus akaara*) using nanopore sequencing and Hi‐ C. Molecular Ecology Resources.

Ghosh, S., and Chan, C.K. (2016). Analysis of RNA-Seq Data Using TopHat and Cufflinks. Methods in Molecular Biology *1374*, 339.

Gong, G.R., Dan, C., Xiao, S.J., Guo, W.J., Huang, P.P., Xiong, Y., Wu, J.J., He, Y., Zhang, J.C., and Li, X.H. (2018). Chromosomal-level assembly of yellow catfish genome using third-generation DNA sequencing and Hi-C analysis. GigaScience *7*, giy120.

Grabherr, M., Haas, B.J., Yassour, M., Levin, J.Z., Thompson, D.A., Amit, I., Adiconis, X., Fan, L., Raychowdhury, R., and Zeng, Q. (2011). Full-length transcriptome assembly from RNA-Seq data without a reference genome. Nature Biotechnology *29*, 644-652.

Griffithsjones, S., Bateman, A., Marshall, M., Khanna, A., and Eddy, S.R. (2003). Rfam: an RNA family database. Nucleic Acids Research *31*, 439.

Hanna, V.S., and Hafez, E.A.A. (2018). Synopsis of arachidonic acid metabolism: A review. Journal of advanced research *11*, 23-32.

Harris, M.A., Clark, J., Ireland, A., Lomax, J., Ashburner, M., Foulger, R., Eilbeck, K., and …White, R. (2004). The Gene Ontology (GO) database and informatics resource. Nucleic Acids Research.

He, C.L., Song, Z.B., and Zhang, E. (2011). *Triplophysa* fishes in China and the status of its taxonomic studies. Sichuan Journal Zoology *30*, 150-155.

He, X.F., He, J.S., and Yan, T.M. (1999). Reproductive characteristic of *Triplophysa bleekeri* in mabian river. Journal of Southwest China Normal University *24*, 69-73.

Jurka, J., Kapitonov, V.V., Pavlicek, A., Klonowski, P., Kohany, O., and Walichiewicz, J. (2005). Repbase Update, a database of eukaryotic repetitive elements. Cytogenetic and genome research *110*, 462-467.

Kohchi, C., Inagawa, H., Nishizawa, T., and Soma, G.-I. (2009). ROS and innate immunity. Anticancer research *29*, 817-821.

Kopan, R., and Ilagan, M.X.G. (2009). The canonical Notch signaling pathway: unfolding the activation mechanism. Cell *137*, 216-233.

Langmead, B., and Salzberg, S.L. (2012). Fast gapped-read alignment with Bowtie 2. Nature Methods *9*, 357-359.

Lee, T., Guo, H., Wang, X., Kim, C., and Paterson, A.H. (2014). SNPhylo: a pipeline to construct a phylogenetic tree from huge SNP data. BMC Genomics *15*, 162-162.

Li, H., Zhang, N., and Lin, X. (2010). Spatio-Temporal Characteristics of Yarlung Zangbo River in Tibet. Journal of Henan Normal University.

Li, J., Fang, X., Song, C., Pan, B., Ma, Y., and Yan, M. (2014). Late Miocene–Quaternary rapid stepwise uplift of the NE Tibetan Plateau and its effects on climatic and environmental changes. Quaternary Research *81*, 400-423.

Li, J.T., Gao, Y.D., Xie, L., Deng, C., Shi, P., Guan, M.L., Huang, S., Ren, J.L., Wu, D.D., and Ding, L. (2018a). Comparative genomic investigation of high-elevation adaptation in ectothermic snakes. Proceedings of the National Academy of Sciences *115*, 8406-8411.

Li, L., Stoeckert, C.J., and Roos, D.S. (2003). OrthoMCL: identification of ortholog groups for eukaryotic genomes. Genome research *13*, 2178-2189.

Li, M., Tian, S., Jin, L., Zhou, G., Li, Y., Zhang, Y., Wang, T., Yeung, C.K., Chen, L., and Ma, J. (2013). Genomic analyses identify distinct patterns of selection in domesticated pigs and Tibetan wild boars. Nature genetics *45*, 1431.

Li, S., Xia, X., Zhou, B., Zhang, S., Zhang, L., and Mou, X. (2018b). Chemical balance of the Yellow River source region, the northeastern Qinghai-Tibetan Plateau: Insights about critical zone reactivity. Applied Geochemistry *90*, 1-12.

Liu, B., Shi, Y., Yuan, J., Hu, X., Zhang, H., Li, N., Li, Z., Chen, Y., Mu, D., and Fan, W. (2013). Estimation of genomic characteristics by analyzing k-mer frequency in de novo genome projects. arXiv: Genomics.

Liu, H.P., Liu, Q.Y., Chen, Z.Q., Liu, Y.C., Zhou, C.W., Liang, Q.Q., Ma, C.X., Zhou, J.S., Pan, Y.Z., and Chen, M.Q. (2018). Draft genome of *Glyptosternon maculatum*, an endemic fish from Tibet Plateau. GigaScience *7*, giy104.

Liu, H.P., Xiao, S.J., Wu, N., Wang, D., Liu, Y.C., Zhou, C.W., Liu, Q.Y., Yang, R.B., Jiang, W.K., and Liang, Q.Q. (2019). The sequence and de novo assembly of *Oxygymnocypris stewartii* genome. Scientific data *6*, 190009.

Liu, Z.J., Liu, S.K., Yao, J., Bao, L.S., Zhang, J.R., Li, Y., Jiang, C., Sun, L.Y., Wang, R.J., and Zhang, Y. (2016). The channel catfish genome sequence provides insights into the evolution of scale formation in teleosts. Nature communications *7*, 11757.

Lobo, I. (2008). Basic local alignment search tool (BLAST). Nature Education *1*.

Lowe, T.M., and Eddy, S.R. (1997). tRNAscan-SE: a program for improved detection of transfer RNA genes in genomic sequence. Nucleic Acids Research *25*, 955-964.

Marcais, G., and Kingsford, C. (2011). A fast, lock-free approach for efficient parallel counting of occurrences of k-mers. Bioinformatics *27*, 764-770.

McGinnis, S., and Madden, T.L. (2004). BLAST: at the core of a powerful and diverse set of sequence analysis tools. Nucleic acids research *32*, W20-W25.

Mckenna, A., Hanna, M., Banks, E., Sivachenko, A., Cibulskis, K., Kernytsky, A.M., Garimella, K., Altshuler, D., Gabriel, S., and Daly, M.J. (2010). The Genome Analysis Toolkit: A MapReduce framework for analyzing next-generation DNA sequencing data. Genome Research *20*, 1297-1303.

Murakami, T., Terai, H., Yoshiyama, Y., Tezuka, T., Zhu, L., Matsunaka, T., and Nishimura, M. (2007). The second investigation of Lake Puma Yum Co located in the Southern Tibetan Plateau, China. Limnology *8*, 331-335.

Myers, N., Mittermeier, R.A., Mittermeier, C.G., Da Fonseca, G.A., and Kent, J. (2000). Biodiversity hotspots for conservation priorities. Nature *403*, 853.

Nawrocki, E.P., and Eddy, S.R. (2013). Infernal 1.1: 100-fold faster RNA homology searches.

Bioinformatics *29*, 2933-2935.

Nelson, J.S., Grande, T.C., and Wilson, M.V. (2016). Fishes of the World (John Wiley & Sons).

Ogata, H., Goto, S., Sato, K., Fujibuchi, W., Bono, H., and Kanehisa, M. (2000). KEGG: Kyoto Encyclopedia of Genes and Genomes. Nucleic Acids Research, *27*, 29-34.

Pirzadah, T.B., Malik, B., Rehman, R.U., Hakeem, K.R., and Qureshi, M.I. (2014). Signaling in response to cold stress. In Plant signaling: Understanding the molecular crosstalk (Springer), pp. 193-226.

Pryszcz, L.P., and Gabaldón, T. (2016). Redundans: an assembly pipeline for highly heterozygous genomes. Nucleic acids research *44*, e113-e113.

Purcell, S., Neale, B.M., Toddbrown, K., Thomas, L., Ferreira, M.A.R., Bender, D., Maller, J., Sklar, P., De Bakker, P.I.W., and Daly, M.J. (2007). PLINK: A Tool Set for Whole-Genome Association and Population-Based Linkage Analyses. American Journal of Human Genetics *81*, 559-575.

Qiu, Q., Zhang, G., Ma, T., Qian, W., Wang, J., Ye, Z., Cao, C., Hu, Q., Kim, J., and Larkin, D.M. (2012). The yak genome and adaptation to life at high altitude. Nature genetics *44*, 946.

Ravi, V., and Venkatesh, B. (2008). Rapidly evolving fish genomes and teleost diversity. Current opinion in genetics & development *18*, 544-550.

Ronnestad, I., Gomes, A.S., Murashita, K., Angotzi, R., Jonsson, E., and Volkoff, H. (2017). Appetite-Controlling Endocrine Systems in Teleosts. Frontiers in Endocrinology *8*.

Simão, F.A., Waterhouse, R.M., Ioannidis, P., Kriventseva, E.V., and Zdobnov, E.M. (2015). BUSCO: assessing genome assembly and annotation completeness with single-copy orthologs. Bioinformatics *31*, 3210-3212.

Stamatakis, A. (2014). RAxML version 8: a tool for phylogenetic analysis and post-analysis of large

phylogenies. Bioinformatics *30*, 1312-1313.

Stamatakis, A., Hoover, P., and Rougemont, J. (2008). A Rapid Bootstrap Algorithm for the RAxML Web Servers. Systematic Biology *57*, 758-771.

Stanke, M., Keller, O., Gunduz, I., Hayes, A., Waack, S., and Morgenstern, B. (2006). AUGUSTUS: ab initio prediction of alternative transcripts. Nucleic acids research *34*, W435-W439.

Sun, Y.B., Fu, T.T., Jin, J.Q., Murphy, R.W., Hillis, D.M., Zhang, Y.P., and Che, J. (2018). Species groups distributed across elevational gradients reveal convergent and continuous genetic adaptation to high elevations. Proceedings of the National Academy of Sciences *115*, E10634-E10641.

Suyama, M., Torrents, D., and Bork, P. (2006). PAL2NAL: robust conversion of protein sequence alignments into the corresponding codon alignments. Nucleic Acids Research *34*, 609-612.

Talavera, G., and Castresana, J. (2007). Improvement of Phylogenies after Removing Divergent and Ambiguously Aligned Blocks from Protein Sequence Alignments. Systematic Biology *56*, 564-577.

Tamura, K., Dudley, J.T., Nei, M., and Kumar, S. (2007). MEGA4: Molecular Evolutionary Genetics Analysis (MEGA) Software Version 4.0. Molecular Biology and Evolution *24*, 1596-1599.

Tarailo‐ Graovac, M., and Chen, N.S. (2009). Using RepeatMasker to identify repetitive elements in genomic sequences. Current protocols in bioinformatics *25*, 4.10. 11-14.10. 14.

Trapnell, C., Pachter, L., and Salzberg, S.L. (2009). TopHat: discovering splice junctions with RNA-Seq. Bioinformatics *25*,, 1105-1111.

Valdes, P.J., Lin, D., Farnsworth, A., Spicer, R.A., Li, S.-H., and Tao, S. (2019). Comment on “Revised paleoaltimetry data show low Tibetan Plateau elevation during the Eocene”. Science *365*, eaax8474.

Walker, B.J., Abeel, T., Shea, T., Priest, M., Abouelliel, A., Sakthikumar, S., Cuomo, C.A., Zeng,

Q., Wortman, J., and Young, S.K. (2014). Pilon: an integrated tool for comprehensive microbial variant detection and genome assembly improvement. PloS one *9*, e112963.

Wang, Z.J., Huang, J., and Zhang, Y.G. (2013). The reproductive traits of *Triplophysa bleekeri* in the Daning River. Freshwater Fisheries *43*, 8-12.

Wei, L. (2009). Spatio-Temporal Characteristics of Niyang River in Tibet. Journal of Henan Normal University.

Wu, Y.F., and Tan, Q.J. (1991). Characteristics of the fish-fauna of the characteristics of Qinghai-Xizang plateau and its geological distribution and formation. Acta Zoologica Sinica *37*, 135-152.

Wu, Y.T., QJ (1991). Characteristics of the fish-fauna of the characteristics of Qinghai-Xizang Plateau and its geological distribution and formation [J]. Acta Zoologica Sinica *2*.

Xiao, H., and Dai, Y.G. (2011). A Review of Study on Diversity of *Triplophysa* in China. Fisheries Science *30*, 53-57.

Xiao, S.J., Han, Z.F., Wang, P.P., Han, F., Liu, Y., Li, J.T., and Wang, Z.Y. (2015). Functional marker detection and analysis on a comprehensive transcriptome of large yellow croaker by next generation sequencing. PloS one *10*, e0124432.

Xiao, S.J., Wang, P.P., Dong, L.S., Zhang, Y.G., Han, Z.F., Wang, Q.R., and Wang, Z.Y. (2016). Whole-genome single-nucleotide polymorphism (SNP) marker discovery and association analysis with the eicosapentaenoic acid (EPA) and docosahexaenoic acid (DHA) content in *Larimichthys crocea*. PeerJ *4*, e2664.

Xie, C., Mao, X., Huang, J., Ding, Y., Wu, J., Dong, S., Kong, L., Gao, G., Li, C.-Y., and Wei, L. (2011). KOBAS 2.0: a web server for annotation and identification of enriched pathways and diseases.

Nucleic acids research *39*, W316-W322.

Yang, L., Wang, Y., Wang, T., Duan, S., Dong, Y., Zhang, Y., and He, S. (2019a). A chromosome-scale reference assembly of a Tibetan loach, *Triplophysa siluroides*. Frontiers in genetics *10*, 991.

Yang, X., Liu, D., Liu, F., Wu, J., Zou, J., Xiao, X., Zhao, F.Q., and Zhu, B.L. (2013). HTQC: a fast quality control toolkit for Illumina sequencing data. BMC bioinformatics *14*, 33.

Yang, X., Liu, H., Ma, Z., Zou, Y., and Yang, R. (2019b). The chromosome-level genome assembly of *Triplophysa tibetana* , a fish adapted to the harsh high-altitude environment of the Tibetan plateau. Molecular Ecology Resources *19*, 1027-1036.

Yang, Z. (2007). PAML 4: Phylogenetic Analysis by Maximum Likelihood. Molecular Biology and Evolution *24*, 1586-1591.

Zhao, Z., and Li, S. (2017). Extinction vs. Rapid radiation: The juxtaposed evolutionary histories of coelotine spiders support the Eocene–Oligocene orogenesis of the Tibetan Plateau. Systematic Biology *66*, 988-1006.

Zhou, Q., Gao, H., Zhang, Y., Fan, G., Xu, H., Zhai, J., Xu, W., Chen, Z., Zhang, H., and Liu, S. (2019). A chromosome- level genome assembly of the giant grouper (*Epinephelus lanceolatus*) provides insights into its innate immunity and rapid growth. Molecular Ecology Resources.

Zhu, S. (1989). The loaches of the subfamily Nemacheilinae in China (Cypriniformes: Cobitidae) (Jiangsu Science and Technology Publishing House).

Figure titles and legends

Figure 1. A picture of *T. bleekeri* used for genome sequencing and assembly.

Figure 2. The geographic distribution of the sampling locations for *T. bleekeri*. The red point, green triangle, yellow trapezoid, and ellipse represent the sampling sites, gorge, artificial dam, and Wuxi Town, respectively.

Figure 3. *K*mer frequency distribution from NGS short-read sequencing data. The peak around the depth of 100X depicts the main Kmer species frequency used for the genome size estimation.

Figure 4. Natural positively selected genes (PSGs) identification and functional analysis for *T. bleekeri, T. tibetana,* and *T. siluroides*. (a) Venn diagram for PSGs for three fish species. (b) Enrichment analysis on the biological pathways for shared PSGs for three species.

Figure 5. Phylogenetic relationships and divergence time estimation for *T. bleekeri* and other fish species. All nodes were completed and supported by the 100 times bootstrap resampling. Blue numbers near the nodes indicate the estimated divergence times with a 95% confidence interval. Divergences used for the recalibration of time estimation are indicated with red dots.

Figure 6. The neighbor-joining phylogenetic tree of *T. bleekeri* individuals based on whole-genome SNP loci. Note that samples from population one, two, and three are labeled with red, green and blue, respectively.

Table 1 Click here to download Table Table 1-Triplophysa bleekeri.docx

Table 1. A summary of sequencing data used in genome assembly and gene annotation.

Source	Platform	Clean Data (Gb)	Mean Read Length (bp)	Sequence Coverage (X)
genome	Illumina HiSeq X Ten	81.7	150	129
genome	PacBio SEQUEL	100.87	5,827	160
genome (Hi-C)	Illumina HiSeq X Ten	83.5	150	132
transcriptome	Illumina HiSeq X Ten	11.1	150	-

Click here to download Table Table 2-Triplophysa bleekeri.docx

Table 2. The length statistics for contig assembly for the *T. bleekeri* genome

	Assemble	Total Length (bp)	Sequence Number	Contig N50 (Mb)	Scaffold N50 (Mb)
contig assembly using long-read data	Falcon	657,392,105	1,357	3.31	3.31
	Arrow	660,275,268	1,357	3.33	3.33
	Pilon	659,964,583	1,357	3.33	3.33
	Redundans	628,132,429	856	3.82	3.82
chromosome assembly using Hi-C data	all sequences	620,272,795	181	3.11	22.89
	chromosomes	596,964,218	25	3.23	23.21
	unanchored sequences	23,308,577	156	0.17	1.01

Figure 1 Click here to download Figure Figure 1-Triplophysa bleekeri.docx

Figure 1. A picture of *T. bleekeri* used for genome sequencing and assembly.

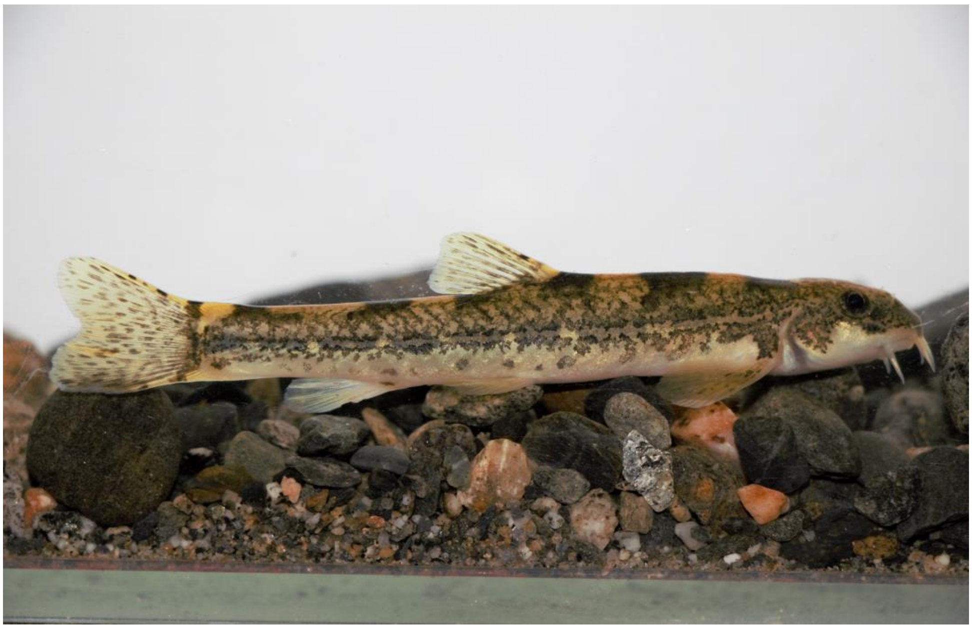

Figure 2

Click here to download Figure Figure 2-Triplophysa bleekeri.docx

Figure 2. The geographic distribution of the sampling locations for *T. bleekeri*.

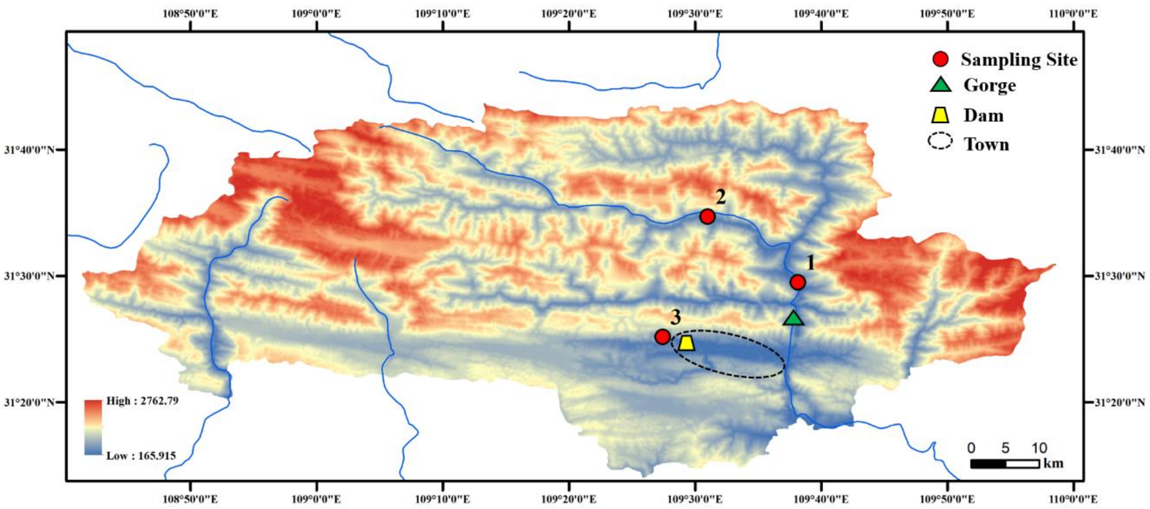

Click here to download Figure Figure 3-Triplophysa bleekeri.docx

Figure 3. *K*mer frequency distribution from NGS short-read sequencing data.

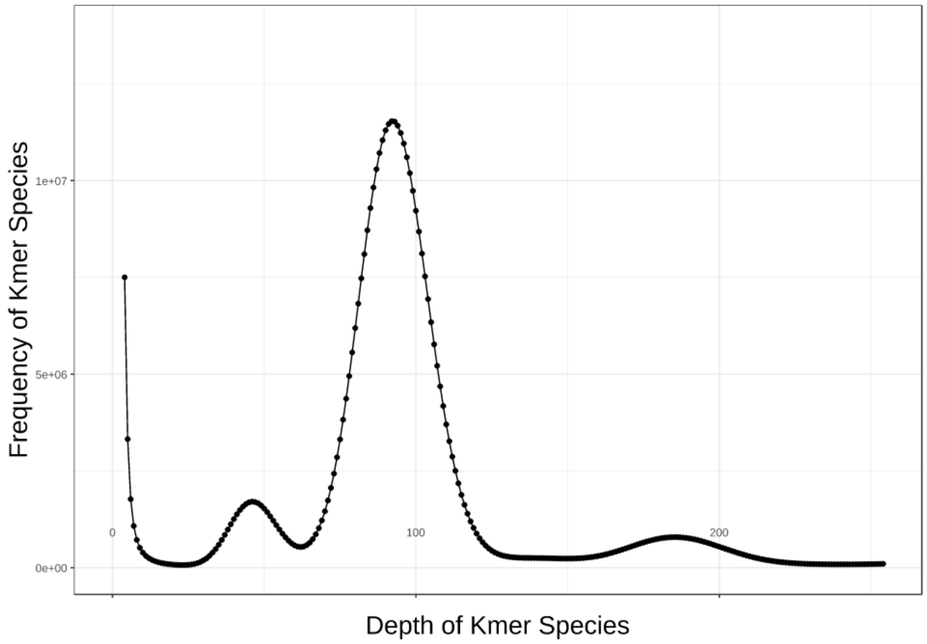

 Click here to download Figure Figure 4-Triplophysa bleekeri.docx

Figure 4. Natural positively selected genes (PSGs) identification and functional analysis for *T. bleekeri*, *T. tibetana*, and *T. siluroides*.

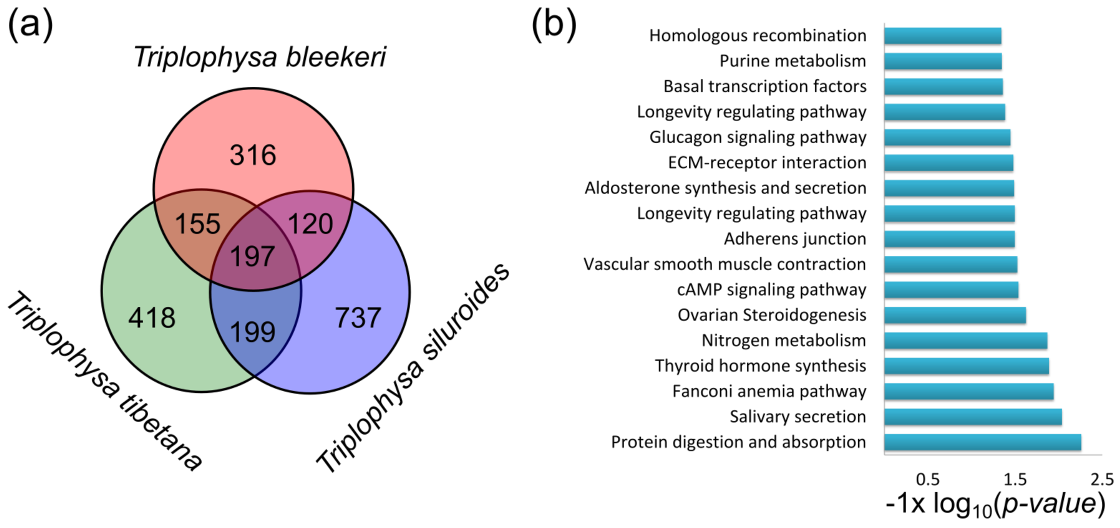

Click here to download Figure Figure 5-Triplophysa bleekeri.docx

Figure 5. Phylogenetic relationships and divergence time estimation for *T. bleekeri* and other fish species.

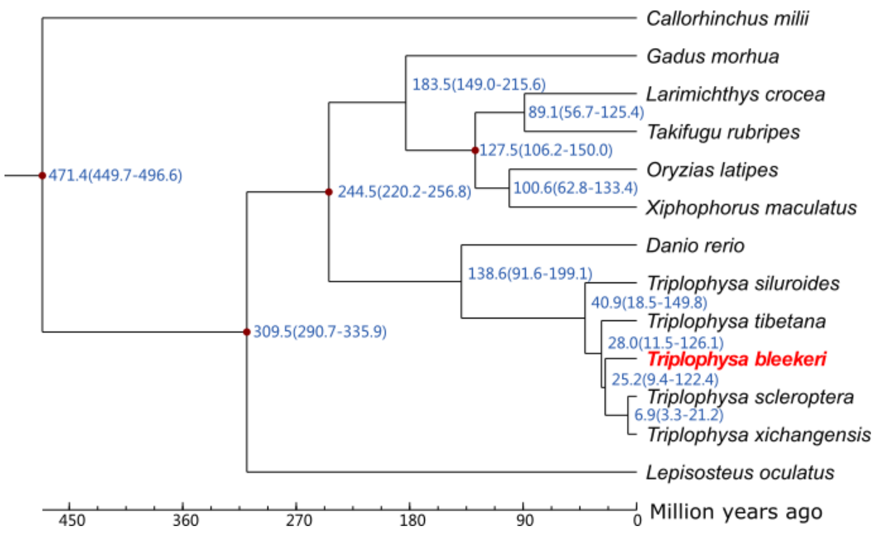

Figure 6 Click here to download Figure Figure 6-Triplophysa bleekeri.docx

Figure 6. The neighbor-joining phylogenetic tree of *T. bleekeri* individuals based on whole-genome SNP loci.

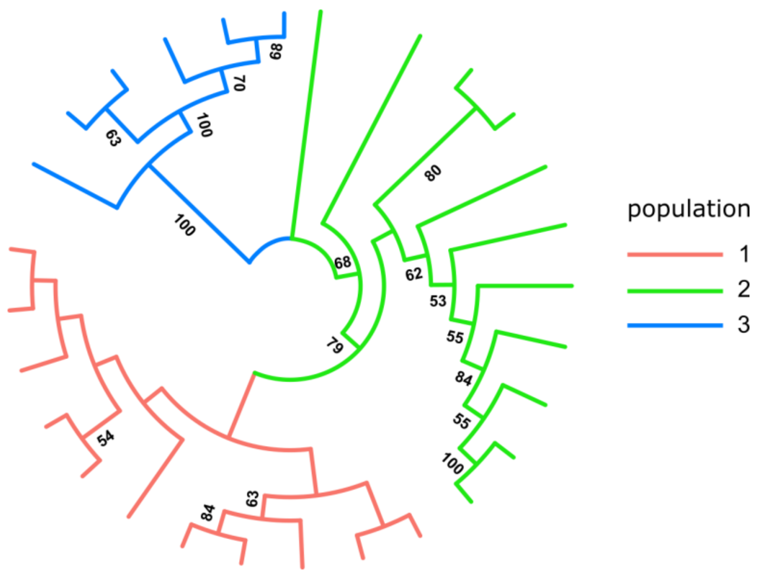

Additional file 1

Click here to access/download
Supplementary Material
Additional file 1.docx

Additional file 2

Click here to access/download
Supplementary Material
Additional file 2-Supplementary Table 6.xls

Additional file 3

Click here to access/download

Supplementary Material

Additional file 3-Supplementary Table 7.xls

Cover letter
Click here to download Personal Cover Cover letter-Triplophysa bleekeri.docx

Dear Editor,

Attached is our recent manuscript entitled "**Chromosomal genome assembly of *Triplophysa bleekeri* provides insights into its evolution and environmental adaptation on the Qinghai-Tibetan Plateau**." We would like to send for your consideration to publish as a resource article in ***GigaScience***.

The continuous uplifts and climatic changes during the formation of Qinghai-Tibetan Plateau (QTP) posed profound influences on the evolution of endemic organisms. Fish species on the QTP are more susceptible to tectonic movements and temperature fluctuations since it strictly constrained by water ecology and drainage connectivity in habitats. Living in extreme environments, including low temperature and high UV exposure, highland endemic fishes have been subjected to severe natural selections; however, few studies were performed to investigate the molecular mechanism of the adaptation formation for fish species on the QTP. The genome and population resources of endemic fish species play an essential role in adaptive evolution studies. Although several genomes of fish species on the QTP, including *Glyptosternon maculatum, Oxygymnocypris stewartii*, *Triplophysa tibetana*, and *Triplophysa siluroides*, have been reported, the genetic resource, especially for whole-genome population data, is still insufficient, largely hindered the evolution and conservation genetics studies of endemic fish.

Triplophysa bleekeri (*T. bleekeri*), a typical fish species occurring at an elevation from 200 m to 3,000 m, provides us an excellent model to investigate the adaption mechanism and population genetics for fish on the QTP. In this study, we assembled a chromosome genome for *T. bleekeri* using Illumina, PacBio sequencing platform, and Hi-C technique. Based on more than 160 X coverage of long sequencing data, we generated a 628 Mb *T. bleekeri* genome with a contig N50 length of 3.82 Mb. Using interaction frequencies among contigs from the Hi-C technique, a chromosome genome was assembled with a scaffold N50 length of 22.9 Mb, and more than 96.2% of the genome on the base level was successfully anchored upon 25 chromosomes. A

total of 21,198 protein-coding genes were predicted in the *T. bleekeri* genome, of which 97.3% of the protein-coding genes were functionally annotated.

We explored the environmental adaptation of *T. bleekeri* from the perspective of functional genes in the genome. We found that gene families related to lipid metabolism, necroptosis, and immune response were significantly expanded in the *T. bleekeri* genome, comparing to those non-highland fish species. Genes involved in DNA repair and protein digestion underwent strong natural positive selections for *T. bleekeri*, *T. siluroides* and *T. tibetana*. Our result implied that *T. bleekeri* might under severe stress with the cold environment, and *Triplophysa* species might be under similar natural selections. We also performed whole-genome resequencing for 28 samples from three populations in the Daning River. We illuminated the difference between their genetic structures, which can be explained by the relatively limited gene flow hampered by natural gorges and artificial barriers, such as cities and dams, among those populations.

Our work provided important reference genome and population variation resource and preliminary investigation of the environmental adaptation and population structures of *T. bleekeri*. Those data will not only be used for further ecological and conservation studies for the species but also offered valuable information for the evolutionary researches based on the comparative analysis among endemic fish and vertebrates.

All authors have read and approved this version of the article. No part of this paper has been published or submitted elsewhere. No conflict of interest exists in the submission of this manuscript.

We are looking forward to receiving a favorable response from you regarding the acceptance of the manuscript. Thank you for your help.

Sincerely Yours,

Prof. Zhijian Wang
School of Life Sciences, Southwest University
Beibei, Chongqing, PR China 400715
Tel: 86-023-68253005
Fax: 86-023-68253005
Email: wangzj1969@126.com
