# Supplementary material for: Chromosomal genome of Triplophysa bleekeri provides insights into its evolution and environmental adaptation
Source: Gigascience. 2020 Nov 24;9(11):giaa132. doi: 10.1093/gigascience/giaa132 (PMC7684707; doi:10.1093/gigascience/giaa132)
Supplement: giaa132_GIGA-D-20-00124_Revision_1 [file giaa132_giga-d-20-00124_revision_1.pdf]

GigaScience

Chromosomal genome of Triplophysa bleekeri provides insights into its evolution and environmental adaptation

--Manuscript Draft--

**Manuscript Number:**	GIGA-D-20-00124R1
**Full Title:**	Chromosomal genome of Triplophysa bleekeri provides insights into its evolution and environmental adaptation
**Article Type:**	Data Note
**Funding Information:**	Financial Program of Ministry of Agriculture and Rural Affairs of China (YYJZHC201921301350063) — Dr. Zhijian Wang<br>National Natural Science Foundation of China (31602207) — Dr. Shijun Xiao<br>Research Innovation Program for College Graduates of Chongqing (CYB19079) — Dr. Dengyue Yuan
**Abstract:**	Background: Intense stresses caused by high-altitude environments may result in noticeable genetic adaptions in native species. Studies of genetic adaptations to high elevations have been largely limited to terrestrial animals. How fish adapt to the high-elevation environments is largely unknown. Triplophysa bleekeri , an endemic fish inhabiting high-altitude regions, presents an excellent model to investigate the genetic mechanisms of adaptation to the local environment. Here, we assembled a chromosomal genome sequence of about 628 Mb with a contig and scaffold N50 of 3.1 and 22.9 Mb, respectively. We investigated the origin and environmental adaptation of T. bleekeri based on 21,198 protein-coding genes in the genome.<br>Results: Compared to fish species living at low altitudes, gene families associated with lipid metabolism and immune response were significantly expanded in the T. bleekeri genome. Genes involved in DNA repair exhibit positive selection for T. bleekeri , T. siluroides , and T. tibetana , indicating that adaptive convergence in Triplophysa species occurred at the positively selected genes. We also analyzed whole-genome variants among samples from three populations. The results showed that populations separated by geological and artificial barriers exhibited obvious differences in genetic structures, indicating that gene flow is restricted between populations.<br>Conclusions: These results will help us expand our understanding of environmental adaptation and genetic diversity of T. bleekeri , and provide valuable genetic resources for future studies on the evolution and conservation of high-altitude fish species such as T. bleekeri .
**Corresponding Author:**	Zhijian Wang<br>Southwest University<br>Chongqing, China CHINA
**Corresponding Author Secondary Information:**	
**Corresponding Author's Institution:**	Southwest University
**Corresponding Author's Secondary Institution:**	
**First Author:**	Dengyue Yuan
**First Author Secondary Information:**	
**Order of Authors:**	Dengyue Yuan<br>Xuehui Chen<br>Haoran Gu<br>Ming Zou<br>Yu Zou

Powered by Editorial Manager® and ProduXion Manager® from Aries Systems Corporation

	Jian Fang
	Wenjing Tao
	Xiangyan Dai
	Shijun Xiao
	Zhijian Wang
**Order of Authors Secondary Information:**	

Response to Reviewers:

Manuscript number: GIGA-D-20-00124
Article Type: DataNote
Title: Chromosomal genome of Triplophysa bleekeri provides insights into its evolution and environmental adaptation
Correspondence Author: Shijun Xiao; Zhijian Wang

Dear editor,
Thank you very much for your supervision in the reviewing process of my manuscript. We also highly appreciate the reviewer's carefulness, conscientiousness, and broad knowledge of the relevant research fields since they have given us many beneficial suggestions. Based on these comments and suggestions, we have made careful modifications to the original manuscript. Besides, the language was improved by Wiley Editing Services. The Enclosed herein, please find the revised manuscript entitled "Chromosomal genome of Triplophysa bleekeri provides insights into its evolution and environmental adaptation" (MS: GIGA-D-20-00124), and the modified parts of the manuscript were highlighted by using the track changes mode in MS Word. We sincerely hope this manuscript will be finally acceptable to be published on GigaScience. Thank you very much for all your help, and looking forward to hearing from you soon.
Best regards.
Sincerely Yours,
Dr. Zhijian Wang

Detailed responses to Reviewers
Reviewer #1
Major Comment:
The manuscript by Ding et al describes the chromosome-level assembly of an endemic highland fish Triplophysa bleekeri. The authors used the combined technology of the Illumina, PacBio, and Hi-C platforms and both DNA and RNA sequencing from 12 tissues for generating 628 Mb highly continuous genome assembly with the scaffold and contig N50 values reaching 22.9 Mb and 3.1 Mb, respectively. More than 596.9 Mb sequences were anchored upon 25 chromosomes. The authors identified 21,198 protein-coding genes, of which 97.3% genes were functionally annotated. The manuscript further characterises the phylogenetic relationships between T. bleekeri and other species with the genus Triplophysa, describes gene family expansion and contraction in the T. bleekeri genome, identifies genes potentially affected by positive selection by estimating the ratios of nonsynonymous to synonymous substitutions and estimates population divergence among three populations based on short-read sequencing data of 28 individuals.
Overall, the assembly and annotation of the genome is well-described and current manuscript is one of several similar works on fish species living on Tibetan plateau. In contrast to genome assembly and annotations, some additional analysis parts of the manuscript, such as population genetic analysis of 28 are rather brief and do not add considerably to the current manuscript. Hence, it would have been interesting if the authors would put more emphasis here, for example by using demographic modelling (e.g. using fastsimcoal2 or other software). Also, it would be interesting if the authors would carry out PSMC analysis to further shed light on temporal changes of effective population size in T. bleekeri which would be very relevant in the context of Qinghai-Tibetan Plateau uplift. My main criticism of the manuscript relates to language and

grammar. Clearly, the language of the manuscript needs serious editing. In the detailed comments/suggestions, I have pinpointed some, but not all, of the most obvious cases. I suggest that the authors will use competent native-speaker language assistance to improve the language throughout the paper. In addition, several conclusions that are based on weak reasoning should be removed or softened (see below).
Response: Dear reviewer, thank you for your kind comments for our manuscript. We appreciate your valuable comments and suggestions to improve our paper. With regard to your comments and suggestions, we wish to reply as follows:
(1) To take further advantage of the whole-genome resequencing data, we have performed the PSMC analysis to investigate the historical effective population size dynamics for T. bleekeri. Meanwhile, we also carried out selective sweep analysis to identify selected genes among the population.
(2) As you pointed out, the grammatical, typographical, and formatting errors in this manuscript have been revised. Additionally, the language of this manuscript was improved by Wiley Editing Services.
(3) According to your kind suggestions, some inappropriate conclusions were revised. The specific changes were marked by using the track changes mode in MS Word and were elucidated in the part of the response to your detailed comments.
Overall, we hope that the changes having been made to the manuscript meet to your satisfaction. Special thanks to you for all your good comments.

Detailed comments:
Comment 1: Lines 25-26: Consider rewording, "These environmental changes induced strong stresses for several organisms,.."
Response: Thanks for your suggestion. We revised this sentences in the abstract (lines: 28~29, Marked MS).

Comment 2: Lines 37-38: Replace "underwent strong natural positive selections..." with "have experienced positive selection".
Line 39: replace "samples from populations" to "samples from three populations".
Line 41: Replace "can be attributed to the disrupted gene exchanges among those populations" to alternative description that gene flow is restricted between populations. Based on the data from 3 locations, it is not possible to identify if these changes have been caused by man made changes (dam) or are occurring naturally.
Response: We revised this sentences mentioned above in lines 43, 46, and 48~49 (Marked MS). Special thanks to you for your good suggestions.

Comment 3: Lines 51-53: Consider rewording "The endemic species of the QTP present a great adaptability to the harsh environments of low temperature and low oxygen". It is not clear what does "a great adaptability" means in this context. Also, if the land rise have increased the river slopes at QTP, it is possible that the oxygen levels in the water are actually reduced. Do you have data on oxygen concentration in QTP rivers to demonstrate that high altitude causes low concentration of O2 in water? I think this is actually quite important, as the high-altutude adaptations in fish because of low oxygen maybe not so widespread at all (you introduce the theme later in the intro).
Response: Thanks for your kind comments. We are very sorry for our inappropriate description, and we revised this sentence in lines 64~67 (Marked MS).
QTP is the highest and largest plateau in the world, imposing an extremely inhospitable environment, including low temperature, oxidative stress, and UV radiation, on native creatures. This harsh environment has resulted in the evolution of similar phenotypic adaptations in humans, mammals, birds, and so on, for example, cold tolerance, hypoxic resistance, enhanced metabolic capacity, and increased body masses 1-3. However, different organisms adapt to high altitudes via multiple genetic routes 4-6. The dissolved oxygen level in the water of QTP rivers is not as low as we predicted. Reaeration is one of the main sources of dissolved oxygen in the water of QTP rivers, given that the flow velocity is relatively fast 7. The previous studies showed that the minimum dissolved oxygen level is about 6.5 mg/L even in the elevation of 4,000 m in many rivers of QTP 7-9. Therefore, we think that low temperature is a typical feature of the river environment on the QTP, which faced up this challenge for all aquatic animals. We also explain this viewpoint in the part of the Discussion (Lines 625~629, Marked MS).

Comment 4: Lines 79-80: Consider rewording "environmental adaptation mechanism of Triplophysa species is far from fully understood".

Powered by Editorial Manager® and ProduXion Manager® from Aries Systems Corporation

Response: Thanks for your kind suggestions. We reworded this sentence in lines 98~99 (Marked MS).

Comment 5: Line 90-109: This section need substantial rewriting as it contains numerous language issues. E.g. Line 90-109: Replace "was" to "is"; Line 92: Remove "just", replace foods to food, is still crucial to is crucial, larva to larvae etc.
Response: These sentences were revised based on your kind suggestions (lines: 112, 115, 123~124, 127, and 124; Marked MS).

Comment 6: Line 115: How were the individuals caught?
Response: The fish were caught by brail nets, and this information was added in the manuscript (line: 148; Marked MS).

Comment 7: Line 120: Did you anesthetize fish or use overdosage of MS22 to euthanize fish?
Response: Yes. We use the tricaine MS-222 (60 mg/L) to anesthetize fish before dissection and sampling. We referenced the previous study 10 and conducted the preliminary experiment to determine the dose.

Comment 8: Line 126: Replace "to obtain a comprehensive transcript" with "to comprehensively characterize transcriptome".
Response: Thanks for your kind suggestion. This sentence was revised in lines 158~159.

Comment 9: Lines 128-129: Did you use any anesthetic? Did you use samples from multiple individuals as indicated in text i.e. "muscle tissues were collected"
Response: Yes. In this case, we also use the tricaine MS-222 to anesthetize fish, as mentioned before. Besides, the muscle tissue of each fish was collected for resequencing. We are sorry for our unclear delineation, and we added the related information in the manuscript (line: 164~165; Marked MS).

Comment 10: Lines 183-184: Change T. bleekeri to italics.
Line 192: Change wording "from the above fish"
Lines 243-245: Remove the Latin names in brackets
Line 261 Remove dot.
Line 276-277: Confusing sentence, consider rewording "the natural selected genes" to " "genes putatively influenced by positive natural selection".
Line 282: Change to "Evolutionary relationships"
Line 292: Replace "genetic structures" to "genetic divergence among T. bleekeri populations"
Response: Thank you very much for your kind review. We revised these sentences according to your kind suggestions (lines: 221, 230, 254~257, 317, 333~334, 338 and 348; Marked MS).

Comment 11: Line 300: The minimum depth seems very low (3) which suggests that heterozygote genotypes are not called correctly.
Response: Thanks a lot for reminding. This is the depth filtering parameters for the SNPhylo, but not for filtering parameters during the SNP calling process. We have added the detailed parameters used for the SNP calling. As we showed in the revised manuscript, the minimal depth for SNP calling is 5 in this analysis, which is higher than the Minimum_depth_of_coverage of 3 in SNPhylo analysis. We have increased the minimal depth requirement to 5 and re-preform the analysis, leading to an identical result. Meanwhile, the parameters of SNPhylo would not influence the phylogenetic relationship, and the following population genetics analysis were not significantly influenced (line: 362; Marked MS).

Comment 12: Lines 305-306: Replace "was performed to test the topology robust" to " was performed to test the robustness of the tree topology"
Line 309: Change "transcriptome" to "transcriptomic"
Line 350: Change to "transcriptome data from 12 tissues was generated".
Lines 359-360: Consider rewording: "Meanwhile, the important function of the non-coding genes received further attention."
Line 361: Replace "non-coding gene loci" to "non-coding DNA sequences"
Line 366: no need to repeat two times significantly

Powered by Editorial Manager® and ProduXion Manager® from Aries Systems Corporation

Response: Thanks for your kind suggestions. We revised these sentences in lines 367~368, 394, 435, 445~447, 449, 514~516 (Marked MS).

Comment 13: Both Supplementary Table S4 and S5 indcate gene family expansions in the heading. Should one of the table represent gene family contractions? With Rich factor, do you mean enrichment factor?
Supplementary Table S4. In the first row, both the gene number and background gene number is one and Q value is 0?
Response: We are very sorry for our negligence. We added the tables of gene family contractions in the Additional File.
(1) Yes, the rich factor here is the enrichment factor, indicating the ratio of selected genes with one GO/KEGG annotation divided by the total number of genes with the identical annotation.
(2) Yes. Both the gene number (in Table S5) and background gene number (in Table S4) are one means the function was extremely enriched. However, the statistics here are not reliable since the sample size is too small here. We have removed the lines with gene number smaller than 5 in Supplementary Table S4 and S5.

Comment 14: Line 381: As this type of analysis relies heavily on what species were included to the analysis, please describe here what species were used.
Response: To identify positively selected genes (PSGs) in the T. bleekeri genome, we compared gene families in the T. bleekeri genome to eight fish species living in non-QTP regions, including Callorhinchus milii, Lepisosteus oculatus, Gadus morhua, Danio rerio, Oryzias latipes, Xiphophorus maculatus, Takifugu rubripes, and Larimichthys corcea. Similarly, the PSGs of T. tibetana and T. siluroides were obtained in the same way. We introduced this information in the section of materials and methods (lines: 324~325, 333~335; Marked MS). Thank you for your kind review.

Comment 15: Line 407: Please change "obtained according to serious filtering criteria". It is not clear what "serious" filtering means here. Based on the relaxed coverage, actually the filtering seems to be very relaxed? Also, did you checked for Hardy-Weinberg equilibrium and was that included as a filtering parameter?
Response: We have applied the filtering criterion during SNP calling according to the suggestion from GATK manual: QUAL (phred quality) > 30, QD (quality score divided by depth to comprehensively evaluate the quality and depth) > 2, read depth (DP > 5), FS (phred-scaled p-value using Fisher's Exact Test to detect strand bias for reads) < 60, MQ (mapping quality to evaluate read alignment) >40, SOR (strand odds ratio to evaluate strand bias for reads) < 4.0. We have added detailed SNP filtering during SNP calling process in the revised manuscript (lines: 355~360; Marked MS). We did not include the Hardy-Weinberg equilibrium test in the SNP filtering.

Comment 16: Lines 411-414: IN the population genetics, the term sister group is not frequently used. Also, what do you mean with monophyly here? I suggest removing this term.
Response: Thanks a lot for the reviewer's suggestion. We have changed the "sister group" to "neighboring group", and we have removed the term of monophyly from the revised manuscript (lines: 566~567; Marked MS).
Comment 17: Line 448: Try to avoid superlatives like "was enormously influenced by uplifts"
Response: Thank you for your kind suggestion. We deleted the inappropriate rhetoric in lines 625~626 (Marked MS).

Comment 18: Line 451: Again, it is not clear to me if the oxygen concentrations in the water have actually deceased in the fast flowing rivers. One can argue that eutrophication likely creates much more frequently decrease of O2 in aquatic environments.
Response: Thank you for your kind review. As I mentioned before, the dissolved oxygen level of the water of QTP rivers is high. The fast flow rate can contribute to the high dissolved oxygen level in the water since reaeration is a primary source of dissolved oxygen. Additionally, a smaller number of aquatic plants and phytoplankton in the water of QTP rivers, especially in some big rivers, such as the Shiquan River 11. Therefore, the eutrophication barely occurs in the water of QTP rivers.

Comment 19: Lines 452-454: This is very confusing and contradictory, you state here

Powered by Editorial Manager® and ProduXion Manager® from Aries Systems Corporation

that in QTP rivers the oxygen concentrations are high! (which supports my earlier point).
Response: Thank you for your kind review. We are very sorry for making you confused due to our imperfect information. We added the related information in lines 629~631 (Marked MS).
The oxygen concentration of air is scarce in the QTP. However, many previous studies showed that the dissolved oxygen in the water of QTP rives is sufficient7-9. Hypoxia is a considerable living challenge for the terrestrial animal of QTP. Compared with the terrestrial animal, the primary living challenge for the fish is low temperature rather than hypoxia, given that the dissolved oxygen level is not low, and that the body temperature of fish depends on water temperature.

Comment 20: Lines 458-459: This study does not really study "the mechanisms by which Triplophysa species adapt to low temperatures". Consider rewording.
Response: We revised this part according to the reviewer's kind suggestion (lines 635~637; Marked MS).

Comment 21: Lines 466-475: The description of the links between low temperatures, ROS and apoptosis is quite speculative and not easy to follow.
Response: Thank you for your kind review. It is true that the link between low temperature and ROS/apoptosis is weak. We have deleted the part of ROS and apoptosis in the manuscript (lines 657~667; Marked MS).

Comment 22: Line 462: Based on supplementary tables and results section, there were also many other gene families that were enriched?
Response: Thanks for your kind review. According to your instruction, we added some key information about expanded gene families in this paragraph (lines 640~642, 648~649; Marked MS).

Comment 23: Lines 484-485: This is highly speculative. Instead, common ancestry seems to be more parsimonious explanation.
Response: Thanks for your kind comments. We have revised this whole paragraph and pointed out that those naturally selected genes might originate from their common ancestor (lines 670~694; Marked MS).

Comment 24: Line 488: Again, please try to avoid superlatives. Not sure what "massive genes" means. Also not sure what "uniquely naturally selected" means.
Response: According to your kind suggestions, we deleted this inappropriate modifier in the sentences (line: 696; Marked MS).

Comment 25: Line 493: Consider removing "sister group".
Response: We deleted these words and reworded the sentence in lines 709~710 (Marked MS).
Comment 26: Line 508: Reword "hypoxia environment". The question is if the habitat of T. bleekeri really has low concentrations of dissolved oxygen.
Response: Thanks for your kind suggestion. We deleted the inappropriate description and reworded the whole paragraph of the Conclusion in lines 756~762 (Marked MS).

Comment 27: Lines 509-510: Consider rewording "It is fascinating to investigate the molecular mechanisms for their environmental adaptation to the QTP" as this sentence does not fit conclusions section.
Response: Thanks for your kind suggestion. We deleted this sentence, and revised the whole paragraph of Conclusion (lines: 756~762; Marked MS).

Comment 28: Lines 520-521: Consider softening the claim as the evidence to link genomic features and environmental parameters is weak at best.
Response: Thanks for your kind suggestion. We deleted the inappropriate description and reworded the whole paragraph of the Conclusion (lines: 759~762; Marked MS).

Comment 29: Lines 521-523: Consider rewording. This claim can be states basically for every freshwater fish without any genetic data.
Response: Thanks for the referee's kind advice. We revised these sentences in the part of the Conclusion (lines 759~762; Marked MS).

Comment 30: Lines 528-529: Consider rewording high land fish"
Response: As the reviewer suggested that we deleted the inappropriate description and reworded the whole paragraph of the Conclusion (lines: 759~762; Marked MS).

Reviewer #2
Major comment:
The paper by D. Yuan and collaborator entitled "Chromosomal genome of Triplophysa bleekeri provides insights into its evolution and environmental adaptation" is a "genome" paper aiming to published a good reference genome for the species T. bleekeri. It also provides a description of potential adaptation to altitude/temperature in Tibetan plateau.

I think that the main aims of producing a good genome for the species is achieved by this paper. However, I find the analyses rather descriptive and therefore the paper is a bit heavy to read. In addition, the gene with potential signal of positive selection should be discuss with more caution see https://royalsocietypublishing.org/doi/full/10.1098/rsbl.2018.0055 that describe some bias of the method use in this paper, but many other paper like this exist)

As the introduction rely a lot on the comparison of different Triplophysa species; I was hoping more comparative discussion in between the different species of Triplophysa that is not really done at the moment, or at least in a confusing way.

In addition, I think that the population genomics analyses could be pushed further. At the moment, the 28 WGS are basically not used in the current paper. I think that classical pop. genomics analyses such as detection of selective sweep, Fst outlier scan and visual variation of statistic such as pi or Fst between pair of populations across T. bleekeri genome would help to frame a nice storytelling to describe the adaptation to the altitude. They would be obvious comparison to do with their other selection test based on dN/dS ratio.
However, this would need some substantial modification of the paper. If producing a genome is still enough to publish a paper nowadays, then, you don't necessarily need to include this change. However, I feel that including better pop. genomic analysis would make the paper more interesting.

Some sentences are also a little bit wordy sometime. I suggested some changes below but I might not be the best candidate for this as English is not my native language.
Response: Dear reviewer, we sincerely appreciate the valuable comments and suggestions.
Thanks a lot for the reviwer’s comments. The caution should be taken when interpreting the positively selected genes since the biases of selection signature generated from the ancient demographic fluctuation might lead to false positive of selected gene identification. To comprehensively describe the natural selection result of genomic comparison and to avoid unnecessary misunderstanding, we have included a statement to reveal the potential biases from this study in the Discussion section. According to your kind suggestions, we added some comparative discussion in between the different species of Triplophysa in the Discussion section. Besides, we performed the PSMC and selective sweep analysis using genome resequencing data. Meanwhile, we have tried our best to polish the language in the revised manuscripts, and the language was edited by Wiley Editing Services. We hope that our manuscript can meet your standards. Thanks again.

Detailed comments:
Comment 1: line 26 : why especially fish ? this is not clear to me. Because they are ectotherm? Maybe this is not really interesting here, every organism are constrain in one way or another by their environment.
Response: Thank you for your kind review. We deleted the inappropriate description and revised this part according to your comment (lines 28~31; Marked MS).

Comment 2: line 82: what is a "typical" species of a given family? maybe remove this.
Response: Thanks for the referee’s kind advice. We deleted this word in lines 101~102

(Marked MS).

Comment 3: Line 84: this sentence doesn't flow correctly we the one above. Considered changing "although" by something else.
Response: Thanks for referee's kind suggestion. We deleted this word and revised the sentences in lines 106~107 (Marked MS).

Comment 4: line 86: need a ref here
Response: Thanks for your kind review. We added the reference in the line 107 (Marked MS).

Comment 5: line 92-> 96= this is wordy, rephrase.
Suggestion: The reproduction biology of Trilophysa fishes is also different, while T. tibetana and T. siluroides spawns once a year (from XX to XX and XX to XX, respectively), T. bleekeri can spawn twice a year with peak breeding seasons occurring from October to December and March to April (Wang et al., 2013)
Response: We revised this sentence according to your kind suggestion (lines 118~122; Marked MS). Special thanks to you.

Comment 6: line 100: why is this "crucial"? I would like to see the specific aims of the study mentioned in here before summarizing the results.
Response: Thanks for your comment. According to your kind guidance, we reworded this part in lines 128~130 (Marked MS).

Comment 7: line 108-109 crucial again. This sentence could be removed as it doesn't provide any relevant information. In my opinion, we should always let reader make their mind about what is "crucial" or not…
Response: We deleted this sentence according to your kind suggestions (lines 139~143; Marked MS). Thanks.

Comment 8: line 128: reword sacrificed: suggestion: To understand the population structures of the species, a total of 28 individuals were collected from three sampling sites, including eleven, seven, and six individuals from populations 1, 2 and 3, respectively (Fig. 2).
Response: Thanks for your kind review. According to your suggestions, we reworded these sentences (lines 161~164; Marked MS).

Comment 9: line 174: precise that this is a software
Response: Thanks for your review. We added the related description in line 211 (Marked MS).

Comment 10: I think that line 179-180 repeat line 174-175
Response: Thanks for the referee's kind comment. We are very sorry for our negligence, and this sentence was deleted (lines 215~217; Marked MS).

Comment 11: line 300: need more explanation about the filtration step. You should not do filtration for LD but for physical linkage. Lots of information are found in the LD information. Was LD performed overall? A depth of 3 is also really low
Response: Thanks for your kind review. Sorry that we have missed the SNP filtering details in the manuscript. According to the suggestion from GATK manual, the filtering criterion was QUAL (phred quality) > 30, QD (quality score divided by depth to comprehensively evaluate the quality and depth) > 2, DP (read depth) > 5, FS (phred-scaled p-value using Fisher's Exact Test to detect strand bias for reads) < 60, MQ (mapping quality to evaluate read alignment) >40, SOR (strand odds ratio to evaluate strand bias for reads) < 4.0. We have added detailed SNP filtering during SNP calling process in the revised manuscript (lines: 357~361; Marked MS). Since the minimal depth parameters of 3 for SNPhylo is smaller than the depth requirement of 5 during SNP calling, therefore the minimal depth parameters of 3 for SNPhylo does not work. We have increased the minimal depth parameters to 5 and reperformed the SNPhylo analysis and obtained identical result. We have revised the manuscript for the depth of 5 (line: 362; Marked MS).
Meanwhile, we are sorry that our method description for SNP filtering is confusing. The Linkage Disequilibrium (LD) filtering was only used for phylogenetic tree construction using SNPhylo utility.

Powered by Editorial Manager® and ProduXion Manager® from Aries Systems Corporation

Comment 12: I would like to see a PCA in your main paper and more analyses showing the variation of classical pop. gen. statistic such as Fst and pi along the genome.
Response: Thanks for the comment. We have added the statistics analysis in the revised manuscript (SI Table 10).

Comment 13: line 444: Nothing is "primitive" when species are still living nowadays, refers as "outgroup", "most divergence species" or something else.
Response: Thanks for the referee's kind advice. We reworded this sentence in line 622~623 (Marked MS).

Comment 14: line 448 : double space. You could also rephrase this sentence
Response: We revised the format and rephrased this sentence according to your suggest (line 626; Marked MS).

Comment 15: line 494:498 this should be back up by value of average Fst
Response: Thanks for the comment. We have calculated the Fst value for LHK-XX, LHK-BY, BY-XX population comparison. As a result, 1,734, 3,009 and 3,244 regions (1 kb), harboring 474, 878, and 957 candidate functional selected genes, were identified to be the significantly genetic differentiated among LHK-XX, LHK-BY and BY-XX comparisons, respectively. Less genomic differentiated regions and candidate genes identified in LHK - XX comparison were consistent with our previous phylogenetic analysis. We have added the result in the revised manuscript (lines: 576~584; Marked MS).

Comment 16: line 503: this is preliminary but you have the data to explore this further. I don't understand why this is not done. At the moment, the population genetic analyses add really little to no information to your current paper.
Response: Thanks a lot for reviewer's comments. We have added a selective sweep analysis using genome resequencing data and identified candidate functional genes in differentiated genomic regions. The GO and KEGG pathway functional analyses were performed to identify the functional enrichment of those candidate genes. The relationship of gene functions to local environments was discussed in the Discussed section (lines: 728~752; Marked MS).

Reviewer #3
General comment:
In this manuscript Yuan and colleagues report the chromosome-level genome assembly of the fish species Triplophysa bleekeri and carried out several comparative genomics analyses in order to investigate the genomic mechanisms that facilitated its adaptation to high-altitude environment. To date, two other chromosome-level genome assemblies are available for the Triplophysa genus, but I found the addition of the T. bleekeri genome a valuable resource considering its wider altitudinal distribution range.

Powered by Editorial Manager® and ProduXion Manager® from Aries Systems Corporation

The authors used a state-of-the-art "recipe" to obtain chromosome-level assemblies by relying on the use of PacBio long reads, followed by Illumina short reads quality correction and Hi-C scaffolding technique. Notably, very high depth of coverage was used for the three sequencing types applied, and it allowed Yuan et al. to obtain very impressive statistics (e.g. contigs N50 = 3.1 Mb, scaffold N50 = 22.9 Mb).
The authors then predicted protein-coding and non-coding genes in the newly generated assembly and they included these data in a phylogenetic framework. Among the main findings, they identified several genes under positive selection in T. bleekeri (many of them also positively selected in the other two species of the same genus) and a bunch of expanded and contracted gene families with functions potentially related to the adaptation to high-altitude environment. Finally, they re-sequenced 28 T. bleekeri individuals representative of three different populations to provide preliminary data on the population structure of this species. In general, the methods used are sound.

That said, I see room for improvement. The manuscript currently is plagued by some disorganization in presented materials and suffers from some clarity issues in analysis descriptions. Additionally, I feel that the manuscript is not particularly well written and I find very hard to follow some sections. Many sentences would benefit from being re-written and in general all the manuscript should be proofread before to be resubmitted.
Response: Thanks a lot for reviewer’s comments and suggestions. We have added more details for the method and re-phased the whole manuscript for a better understanding. Sentences that might lead to obscurity for readers have been rewritten in the revised manuscript. Additionally, the language of this manuscript was improved by Wiley Editing Services. We hope that our manuscript can meet your standards. Special thanks to you.

Major comment:
Comment 1: The methods used are robust for the many analyses the authors carried out. However, I think the results could be presented in a better way by at least re-organizing the order of appearance of the different sections, including more information and refining the main figures. The Results section starts with the genome assembly, but I would then describe ortholog detection immediately followed by the phylogenetic analysis. It would help the reader to contextualize their species with the others included in the phylogeny. I would then go with positive selected genes and only after them describe the expansion/contraction of gene families.
Response: Thanks for your kind suggestions. We re-organized the order of the Results section and revised figures and languages. We very appreciate your suggestion and review.

Comment 2: I am not sure I see the value of the re-sequencing analysis. How the three populations were selected? Do they live in different environments? I would have chosen three populations living at different altitudes (T. bleekeri has a wide distribution ranging from 200 to 3,000 m), as it would have helped, for example, to identify genomic regions of higher divergence in comparison to the background (is it the case?). This in turn would have helped to identify candidate regions potentially harboring genes responsible for adaptation to different high-altitude environment. These candidate genes could have then been compared to the ones identified with the positive selection analyses using the multi-species approach. To sum up, the rationale behind populations selection needs to be better described, and this dataset better explored (e.g. PCA and Admixture are OK, but I would like to see some basic population genetic metrics like e.g. Fst, and Fst values can be shown using Manhattan plot-like style for each pairwise comparison). Finally, I am not sure the tree of Fig. 3 (quite poor graphically) is the best way to report these results. I would suggest the author to produce a more comprehensive new Figure made of 3 panels showing the tree plus the PCA and the Admixture plot (now Suppl. Fig 6 and 7).
Response: Thanks for your kind review.
For the genome sequencing, we collected the T. bleekeri from the high altitude region of the Daning River. For the population re-sequencing, T. bleekeri populations were selected from three different reaches of the Daning River (i.e., Lianghekou, Xixi, and Baiyang) because they are separated by natural or artificial barriers. We would like to investigate how those barriers block gene exchange and diversity among those populations. We have performed the PSMC and selective sweep analysis using the population data and added results into the revised manuscript.

Powered by Editorial Manager® and ProduXion Manager® from Aries Systems Corporation

	We also had planned to collect T. bleekeri from regions with different altitudes, such as the Zhougong River (~2,000 m) and Qingyi River (~3,000 m). However, it is hard to get a permit to fish in these areas because fishing is forbidden for the whole year. In Daning River (~1,000 m), fishing is just banned in spring, so we chose to collect fish in the Daning River. For the genome sequencing, we collected the fish from the high altitude region of the Daning River.<br>We have performed selective sweep analysis, and Manhattan plot was generated to show the result. We also re-generated a figure for the population genetics analysis according to the reviewer's suggestion.<br>If there is any chance, we'd like to collect fishes living at different altitudes to study the genetic divergence among different populations. Thanks for your good suggestion.<br><br><br>Comment 3: The Discussion is very redundant. Frequently the results are reported exactly as they are reported in the Results section, and only very briefly discussed. I would shorten the Discussion by highlighting the main points and including more interpretation of the results and real discussion. The Conclusion section, again, is a repetition of what have been said before in the Discussion. I would delete this section and implement the few original sentences in the Discussion.<br>Response: Thanks for your kind suggestions. We deleted the redundant part and revised the whole section of the Discussion and Conclusion. The modified parts of the manuscript were highlighted by using the track changes mode. We hope that the correction will meet with approval.<br><br>Minor points:<br>Comment 1: L. 27: "Triplophysa bleekeri (T. bleekeri)"<br>The "(T. bleekeri)" is not necessary here. Use T. bleekeri later in the text in the same section, and maybe re-use the full name the first time you mention it in a new section.<br>Response: We deleted the "(T. bleekeri)" in the sentences in accordance with your advice (line 31; Marked MS).<br><br>Comment 2: L. 127.128: 11+7+6=24, but here I read 28 individuals.<br>Response: We are very sorry for our negligence. We sampled eleven, eleven, and six individuals from three reaches of Daning Rivers (i.e., Lianghekou, Xixi, and Baiyang). We revised this clerical error in lines 161~164 (Marked MS).<br><br>Comment 3: L. 32-34: "Phylogenetic analysis showed that T. bleekeri diverged 38.8 and 28.6 million years ago (Ma) from their common ancestors of Triplophysa siluroides and Triplophysa tibetana, respectively"<br>This sentence can be removed from the abstract, or simply replaced with something mentioning that you resolved the phylogeny of the genus Triplophysa, without numbers.<br>Response: We removed this sentence from the abstract in conformity with your kind instructions (lines 37~39; Marked MS). Thanks very much.<br><br>Comment 4: L. 77: change "board" to broad"<br>Response: We are very sorry for our negligence, and we revised this spelling mistake (line 91; Marked MS).<br><br>Comment 5: L. 82 and 84-85: see my comment above.<br>Response: We have made corrections according to your comments (lines 101, 107; Marked MS).<br><br>Comment 6: L. 103: Hi-C is not a platform, it is a method. Please rephrase it.<br>Response: We deleted this word in the sentence (line 132; Marked MS). Thanks for your advice.<br><br>Comment 7: L. 115: Full name here.<br>Response: We changed "T. bleekeri" to "Triplophysa bleekeri" in conformity with your kind instructions (line 146; Marked MS). Thanks very much.<br><br>Comment 8: L. 126: change "comprehensive transcript" to "comprehensive transcript set" or "comprehensive transcriptome".<br>Response: Thanks for the referee's kind advice. We reworded these words in line

Powered by Editorial Manager® and ProduXion Manager® from Aries Systems Corporation

158~159 (Marked MS).

Comment 9: L. 128: See my main comment above. Again, can you use a name for the populations instead of 1, 2 and 3? Both here and in the Figure (now Fig. 6).
Response: Thanks for your kind suggestions. We fished the T. bleekeri form different river sections, including Lianghekou (LHK), Xixi (XX), and Baiyang (BY). Therefore, we use the LHK, XX, and BY instead of 1, 2, and 3. We revised the population names in the whole manuscript.

Comment 10: Most of the versions of the program used are missing. Please revise it, choose a style (e.g. v1.2; version 1.2; v 1.2 etc) and be consistent.
Response: Thanks a lot for reviwers' reminding. We have added software version information in the revised manuscript.

Comment 11: L. 135: Change "Hiseq" to "HiSeq", and check this and other names for consistency along the text.
Response: We revised this clerical error and checked the all names of software in the manuscript (line 171; Marked MS).

Comment 12: L. 137: "Pacific Biosciences of California," "of California" not required.
Response: We deleted these words in the line 173 (Marked MS). Thanks for your kind review.

Comment 13: L. 183: species name in italics
Response: We are very sorry for our incorrect writing, and we revised this error (line 221; Marked MS).

Comment 14: L. 190: The database used for the BUSCO analysis should be specified here.
Response: The database of actinopterygii_odb9 was used for the BUSCO analysis. We have added it to the revised manuscript (lines: 227~228). Thanks for reminding.

Comment 15: L. 213: change "de novo" with "ab initio"
Response: Thanks for your kind suggestion. We changed "de novo" to "ab initio" in the line 251 (Marked MS).

Comment 16: L 226: No details are reported on how you filtered the genes predicted by Maker. I would add the quality filtering step here (genes predicted in repetitive regions were removed? Any overlapping threshold used? Genes with internal stop codons removed? etc).
Response: Thanks for your comment. The quality of gene prediction was filtered as the following steps: We only selected genes with the start and stop codons, and gene with internal stop codons was removed. Only genes with completed sequences and 70% overlap among different gene model prediction methods will be retained as high-quality gene models. We have added the filtering details in the revised manuscript (lines 265~267; Marked MS).

Comment 17: L. 243-245: I would use common names for each species here, and scientific full names in parenthesis.
Response: Thanks for your kind advice. We revised this part according to your instructions (lines 282~285; Marked MS).

Comment 18: L. 253: Which model did you use in RaxML?
Response: Thanks for reminding. Phylogenetic analyses were performed using RAxML with a GTRGAMMA model. We have added the information to the revised manuscript (line 294; Marked MS).

Comment 19: L. 257: This is too generic. Which calibration points did you use?
Response: We used the divergence time of Danio rerio and Larimichthys crocea (255-205 Ma), Oryzias latipes and Larimichthys crocea (115-105 Ma), Lepisosteus oculatus and Danio rerio (338-291 Ma), and Callorhinchus milii and Danio rerio (497-450 Ma) as calibration points for the divergence time estimation for other species. We have added the information to the revised manuscript (lines 297~301; Marked MS).

Powered by Editorial Manager® and ProduXion Manager® from Aries Systems Corporation

Comment 20: L. 288-291: I would include more details here. For example, which Trinity transcripts did you use for the ortholog detection analysis? The longest transcript for each gene? The pipeline you used should be better described.
Response: Thanks for your reminding. The longest transcripts for each gene were used for the phylogenetic analysis. We have reordered sections in the method and added more details for the analysis pipeline (lines 302~310; Marked MS).

Comment 21: L. 426: In the abstract the contigs N50 is 3.1, here 3.82. Please use the right value and be consistent. Again, I would report the scaffold N50 here? Talking about chromosomes: is the karyotypic number known for this genus or this species, for example from cytogenetic studies? In case, I think it is worth mentioning and discussing it (karyotypic number from cytogenetic vs. assembled chromosomes).
Response: Sorry that we should clarify here that contig N50 of 3.82 Mb is the assembly result from sole PacBio sequencing data. However, contigs might be broken during the scaffolding procedure using Hi-C data analysis. Therefore, we reported the contig N50 after Hi-C based assembly in the abstract. To clarify and to keep the consistent description of the assembly, we revised the contig N50 length and added scaffold N50 value here (line 601; Marked MS).
Our laboratory studied the karyotype of Triplophysa bleekeri, revealing that Triplophysa bleekeri has 50 chromosomes (2n=50). The results are as follows:

We referenced this results when we assembled the genome of Triplophysa bleekeri. Finally, 96.2% of the total contig bases were anchored to the 25 chromosomes. According to your suggestion, we added the related discussion in lines 603~606 (Marked MS).
Comment 21: L. 444: what "primitive" means? Do you mean "basal"?
Response: We are very sorry for our incorrect writing. The phylogeny results indicated Triplophysa siluroides is a basal species in the Triplophysa genus. We revised the sentence in line 622~623 (Marked MS) according to your kind instruction.

Comment 22: I would combine Fig. 1 and 2 in a single figure.
Response: Thanks for your kind suggestion. We combined Fig.1 and Fig.2 into one figure.

Comment 23: Fig. 3: can be moved to supplementary material.
Response: We moved the Fig.3 to supplementary material according to your kind suggestion.

Comment 24: Fig. 4: Did you carry out the positive selection analysis for each of the three species independently and then check for overlap? If so, I would also include a PAML analysis to identify positive selected genes in the lineage that includes all Triplophysa species.
Response: Yes, we performed the positive selection analysis for each of the three species independently. According to the reviewer's suggestion, we also performed the PAML analysis and identified 439 PSGs for Triplophysa ancestral lineage. We have compared the species- and lineage-based PSGs and found that only 35 PSGs from lineage-based identified PSGs were identical with those from species-based identified ones. The homologous recombination and basal transcription factor pathways were both enriched for lineage- and species-based PSG identifications. We have added the new results in the revised manuscript (lines 496~508; Marked MS).

Comment 25: I would suggest to make the phylogenetic tree the new Fig 2 and improve its quality. Small images of fishes could make the figure nicer and easier to read. Additionally, the confidence intervals at each node could be shown graphically and not with numbers (very difficult to read and interpret).
Response: We have revised the time bars in phylogenetic tree and added the fish images in Figure 2.

References
1Monge, C. & Leonvelarde, F. Physiological adaptation to high altitude: oxygen transport in mammals and birds. Physiological Reviews 71, 1135-1172 (1991).
2Wu, T. & Kayser, B. High Altitude Adaptation in Tibetans. High Altitude Medicine &

Powered by Editorial Manager® and ProduXion Manager® from Aries Systems Corporation

Biology 7, 193-208 (2006).
3Beall, C. M. Two routes to functional adaptation: Tibetan and Andean high-altitude natives. Proceedings of the National Academy of Sciences of the United States of America 104, 8655-8660 (2007).
4Li, M. et al. Genomic analyses identify distinct patterns of selection in domesticated pigs and Tibetan wild boars. Nature genetics 45, 1431 (2013).
5Hao, Y. et al. Comparative transcriptomics of 3 high-altitude passerine birds and their low-altitude relatives. Proceedings of the National Academy of Sciences of the United States of America 116, 11851-11856 (2019).
6Li, J. et al. Comparative genomic investigation of high-elevation adaptation in ectothermic snakes. Proceedings of the National Academy of Sciences of the United States of America 115, 8406-8411 (2018).
7Lyu, L., Li, Z., Cui, C., amp, T. A. & University, A. H. Study on the Variation of Dissolved Oxygen in the Plateau River. Environmental Science & Technology (2018).
8Zhang, N. et al. Spatio-Temporal Characteristics of Niyang River in Tibet. Journal of Henan Normal University (2009).
9Li, H., Zhang, N. & Lin, X. Spatio-Temporal Characteristics of Yarlung Zangbo River in Tibet. Journal of Henan Normal University (2010).
10Zhang, J. M., Guo, B. F., Yuan, T. & Tian, T. Anesthetic Effect of MS-222 on Elongate Loach Leptobotia elongata. Chinese Journal of Fisheries (2014).
11Wang, D. The geography of aquatic vascular plants of Qinghai-Xizang (Tibet) Plateau (in Chinese). Dissertation for the Doctoral Degree. Wuhan: Wuhan University 85 (2003).

Additional Information:	
**Question**	**Response**
Are you submitting this manuscript to a special series or article collection?	No
**Experimental design and statistics**<br><br>Full details of the experimental design and statistical methods used should be given in the Methods section, as detailed in our Minimum Standards Reporting Checklist. Information essential to interpreting the data presented should be made available in the figure legends.<br><br>Have you included all the information requested in your manuscript?	Yes
**Resources**<br><br>A description of all resources used, including antibodies, cell lines, animals and software tools, with enough information to allow them to be uniquely identified, should be included in the Methods section. Authors are strongly encouraged to cite Research Resource Identifiers (RRIDs) for antibodies, model organisms and tools, where possible.	Yes

Have you included the information requested as detailed in our Minimum Standards Reporting Checklist?	
**Availability of data and materials**<br><br>All datasets and code on which the conclusions of the paper rely must be either included in your submission or deposited in publicly available repositories (where available and ethically appropriate), referencing such data using a unique identifier in the references and in the “Availability of Data and Materials” section of your manuscript.<br><br>Have you have met the above requirement as detailed in our Minimum Standards Reporting Checklist?	Yes

Powered by Editorial Manager® and ProduXion Manager® from Aries Systems Corporation

Manuscript (Marked)
Click here to access/download;Manuscript;Manuscript (Marked) -Triplophysa bleekeri.docx

Chromosomal genome of *Triplophysa bleekeri* provides insights into its evolution and environmental adaptation

Dengyue Yuan[1], Xuehui Chen[1], Haoran Gu[1], Ming Zou[2], Yu Zou[2], Jian Fang[2], Wenjing Tao[1], Xiangyan Dai[1], Shijun Xiao[2,3,*], Zhijian Wang[1,*]

[1] Key Laboratory of Freshwater Fish Reproduction and Development (Ministry of Education), Key Laboratory of Aquatic Science of Chongqing, School of Life Sciences, Southwest University, Chongqing 400715, China

[2] School of Computer Science and Technology, Wuhan University of Technology, Wuhan, Hubei 430000, China

[3] College of Plant Protection, Jilin Agriculture University, Changchun, Jilin 130118, China

* Correspondence to Prof. Dr. Zhijian Wang (wangzj1969@126.com) and Dr. Shijun Xiao (shijun_xiao@163.com)

Abstract

~~The uplift of the Qinghai-Tibetan Plateau (QTP) resulted in profound climatic changes in both QTP and its adjacent regions. These environmental changes induced strong stresses for several organisms, especially fish, since fish are strictly constrained by the living conditions.~~

Background: Intense stresses caused by high-altitude environments may result in noticeable genetic adaptions in native species. Studies of genetic adaptations to high elevations have been largely limited to terrestrial animals. How fish adapt to the high-elevation environments is largely unknown. *Triplophysa bleekeri* ~~(*T. bleekeri*)~~, an endemic ~~highland~~ fish inhabiting high-altitude regions, presents an excellent model to investigate the genetic mechanisms of adaptation to the local environment. Here, we assembled a chromosomal genome sequence of about 628 Mb with a contig and scaffold N50 of 3.1 and 22.9 Mb, respectively. We investigated the origin ~~evolution~~ and environmental adaptation of *T. bleekeri* based on 21,198 protein-coding genes in the genome. ~~Phylogenetic analysis showed that *T. bleekeri* diverged 38.8 and 28.6 million years ago (Ma) from their common ancestors of *Triplophysa siluroides* and *Triplophysa tibetana*, respectively.~~

Results: Compared to fish species living at low altitudes, gene families associated with lipid metabolism~~, necroptosis,~~ and immune response were significantly expanded in the *T. bleekeri* genome~~,~~. ~~and genes~~ Genes involved in DNA repair ~~and protein digestion underwent strong natural positive selections~~ exhibit positive selection for *T. bleekeri*, *T. siluroides*, and *T. tibetana*, indicating that adaptive convergence in *Triplophysa*

species occurred at the positively selected genes. We also analyzed whole-genome variants among~~ samples from populations~~ samples from three populations.~~, and ~~ The results showed that populations separated by geological and artificial barriers exhibited obvious ~~different ~~differences in genetic structures, indicating that gene flow is restricted between populations.~~which can be attributed to the disrupted gene exchanges among those populations. ~~

Conclusions: These results will help us expand our understanding of environmental adaptation and genetic diversity of *T. bleekeri*, and provide valuable genetic resources for future studies on the evolution and conservation of high-altitude fish species such as *T. bleekeri*.

~~The chromosomal genome and population data provided valuable genetic resources for the following evolutionary and environmental adaptation investigations for *Triplophysa* and other high-land fish species.~~

Keywords: *Triplophysa bleekeri*, genome, genetic adaptation, population genomics

Introduction

The Qinghai-Tibetan Plateau (QTP), the largest and highest plateau in the world, is one of the most important world biodiversity centers [1]. The environments of QTP and its peripheral~~adjacent~~ areas were affected significantly by the continuing uplifts, which is one of the most important driving forces for the biological evolution of organisms on the plateau~~QTP~~ [2]. The endemic species of the QTP present ~~a great~~high adaptability to the harsh~~ environments~~ environmental conditions, such as low temperature, low oxygen supply, and high UV radiation by exhibiting cold tolerance, hypoxia resistance,

enhanced metabolic capacity, and increased body mass ~~of low temperature and low oxygen~~ [3-6].

An investigation into the biological evolution of organisms residing on the QTP and its peripheral regions will broaden ~~would widen~~ our understanding of essential evolutionary questions regarding mechanisms of environmental adaptation and speciation~~ for organisms~~. Phenotype comparisons were frequently used to study environmental adaptations in previous studies [7, 8]. In recent years, advancing genomic technology, especially ~~third~~ third-generation sequencing techniques, has presented novel opportunities to explore the genetic basis of environmental adaptations. Many genomic studies of terrestrial animals on the QTP and its peripheral regions revealed that genes involved in hypoxia response, energy metabolism, and DNA repair were under positive selection and rapid evolution [9-11]. In those studies, high-quality genome and population resources are essential to understand ~~important~~ critical biological processes for adaptations [11-13].

The QTP boasts of having many highland fish species, especially in the family Sisoridae, subfamily Schizothoracinae, and genus *Triplophysa* [14]. To date, there have only been several~~four~~ high-quality highland fish genomes reported~~,~~ based on long-read sequencing data, including *Glyptosternon maculatum* in the family Sisoridae, *Schizothorax o'connori* and *Oxygymnocypris stewartii* in the subfamily Schizothoracinae, and *Triplophysa tibetana* and *Triplophysa siluroides* in the genus *Triplophysa* [15-19]. *Triplophysa* is a ~~strongly diverged species~~ highly diverse genus and the largest group of the subfamily Nemacheilinae [20]. There are 152 records for

Triplophysa species in FishBase, and the majority are distributed on the QTP and its adjacent drainage~~s~~ areas from an elevation of 100 m to over 5,200 m [21]. Given ~~Due to~~ the broad ~~board~~ elevation distributions and species diversity, the *Triplophysa* ~~species~~ genus offers an attractive study model not only to investigate the adaptive mechanisms of fish in high altitudes, but also to examine the similarities and differences between the adaptive mechanisms in different *Triplophysa* species. ~~are thought to present an excellent opportunity to explore evolution and environmental adaptation. However, the environmental adaptation mechanism of *Triplophysa* species~~ Previous studies have reported the genomic data of *T. siluroides* and *T. tibetana* without any emphasis on the genetic basis of high-altitude adaption [17, 18]. To date, environmental adaptations of *Triplophysa* species to high altitudes are not ~~is far from~~ fully understood, and the genetic resources for the reference genome and population data remain~~s~~ insufficient.

Triplophysa bleekeri ~~(*T. bleekeri*), a typical species of~~ , another member of the Nemacheilidae~~*Triplophysa*~~ family, is mainly distributed in the stem streams and tributaries of the Yangtze ~~River~~ and Jinsha rivers ~~River~~[22]. ~~Although *T. siluroides*, *Trilophysa bleekeri* and *Triplophysa tibetana* are fish in the *Trilophysa* genus, they exhibit distinct different ecological niches on the QTP and peripheral regions, leading to various physiological characters~~It exhibits different ecological and physiological characteristics compared with its relatives, *T. siluroides* and *T. tibetana* [23]. *T. bleekeri* has a wide distribution, from 200 m to 3,000 m [24], whereas *T. tibetana* and *T. siluroides* occur at elevations of 4,000 ~ 5,000 m, and 3,000 ~ 4,000 m, respectively. ~~*T. tibetana* occurs at elevations from 4,000 ~ 5,000 m, while *T. siluroides* lives at altitudes~~

~~from 3,000 ~ 4,000 m~~[17, 25]. ~~Compared to *T. tibetana* and *T. siluroides*,~~ Apart from ~~the living~~ altitude of habitation, there ~~was~~ is a significant difference in habitat environments. *T. bleekeri* lives in the fast-flowing rivers, whereas *T. tibetana* and *T. siluroides* inhabit lakes and slow flowing ~~in~~ rivers ~~and lake with slow flow, whereas *T. bleekeri* just lives in the fast-flowing river~~[26]. ~~Meanwhile, the reproduction biology for *Trilophysa* fishes is also different that the breeding season for *T. tibetana* is June to July, and *T. siluroides* is July to August. But *T. bleekeri* can spawn twice a year, and the peak seasons of breeding are October to December and March to April~~ Reproduction biology in these *Triplophysa* species is also different; *T. tibetana* and *T. siluroides* spawn once a year (from June to July, and July to August, respectively), whereas *T. bleekeri* can spawn twice a year, with peak breeding seasons occurring from October to December and March to April [24]. ~~The *Triplophysa* fishes also exhibit noticeable differences in feeding habits and morphologic characteristics.~~ The primary ~~foods~~ food source of *T. bleekeri* and *T. tibetana* are ~~chironomus~~ *Chironomus* larvae~~larva~~, caddis fly larvae, and diatoms, whereas~~but~~ *T. siluroides* ~~feeding~~ feeds ~~more on~~ smaller fishes [25]. ~~Therefore, although genomes for *T. siluroides* and *T. tibetana* has been reported before, genome resource for *T. bleekeri* is still crucial in environmental adaptation and evolution studies for *Triplophysa* fishes.~~ The genome resource for *T. bleekeri* will contribute to understanding its evolution and environmental adaption and explore the convergent genetic mechanisms of *Triplophysa* species in high-elevation adaption.

In this ~~work~~study, we generated the first chromosomal genome sequence of *T. bleekeri* using the combined technology of the Illumina, PacBio, and Hi-C ~~platforms~~.

~~Based on these genome data, we studied the evolutionary relationship with closely related species. We identified gene families under expansion, as well as positively selected genes (PSGs), and attempted to identify critical functional genes of *T. bleekeri* contributed to its adaptability.~~ Evolutionary and comparative genomic approaches were applied to clarify the origin of *T. bleekeri*, and to investigate the potential signals of adaption. Further, ~~The~~ the population genetics of *T. bleekeri* were also investigated to reveal the genetic divergence ~~structures~~ among different populations. ~~Those relevant genomic data provide crucial resources for biological and evolutionary studies. This work also investigated the adaptive mechanism of highland fish in the cold environment and demonstrates the geological and artificial barriers might influence the genetic structures of *T. bleekeri* populations.~~

Materials and Methods

Samples and tissue collection

Triplophysa bleekeri~~*T. bleekeri*~~ ~~(**Fig.1**) samples~~ individuals (**Fig. 1a**) were obtained from the Daning River (31°09′26.58″N, 109°53′31.68″E) ~~(**Fig.2**)~~, ~~which is~~ a tributary in the upper reaches of the Yangtze River, using brail nets (**Fig. 1b**). ~~Fish~~ The fish were then transferred to the Aquaculture Laboratory of Southwest University~~,~~ and reared in indoor tanks. To collect enough tissues for the genome and transcriptome sequencing, the largest female individual was used for ~~the~~ library construction and sequencing. The fish was anesthetized with tricaine MS-222, and was immediately dissected~~. Meanwhile,~~ to collect 12 types of tissues viz., ~~types were collected, including~~ brain, eye, skin, gill, heart, liver, trunk kidney, spleen, gut, muscle, gallbladder, and gonad. Tissues were

quickly frozen in liquid nitrogen for more than one hour, and then stored at −80℃. Among these tissues, muscle tissue was used for genomic DNA sequencing and Hi-C library construction. Meanwhile, all tissue samples were used in the application of transcriptome sequencing~~ to obtain a comprehensive transcript~~ to comprehensively characterize transcriptome. ~~To understand the population structures of the species, 28 individuals, including eleven, seven, and six samples collected from population 1, 2, and 3 (**Fig. 2**),~~ To understand the population structures of *T. bleekeri*, a total of 28 individuals were collected from three different reaches of Daning River, i.e., eleven, eleven, and six individuals from Lianghekou (LHK), Xixi (XX), and Baiyang (BY), respectively (**Fig. 1b**). These individuals were anesthetized~~sacrificed~~ with tricaine MS-222, and muscle tissue~~s~~ of each fish was ~~were~~ collected as aforementioned.

Genome DNA extraction and sequencing library construction

DNA was ~~molecules were~~ extracted from muscle tissue using the phenol-chloroform DNA extraction method [27]. The Qubit (Thermo Fisher Scientific, Waltham, MA, USA) and Agilent Bioanalyzer 2100 (Agilent Technologies, Palo Alto, CA, USA) ~~was~~ were used for evaluating the quantity and quality of DNA. For sequencing based on the Illumina ~~Hiseq~~ HiSeq technology, a short-read sequencing library with an insert size of 250 bp was constructed using 1 μg of DNA. For sequencing on the PacBio SEQUEL platform (Pacific Biosciences~~ of California~~, Menlo Park, CA, USA), the muscle DNA ~~molecules from muscle tissue were~~ was ~~also~~ used to construct the long-read sequencing library~~ for PacBio platform~~. Briefly, 10 μg of *T. bleekeri* genomic DNA ~~were~~ was used for 20-kb library preparation following the

manufacturer's protocol (Pacific Biosciences), and the BluePippin Size Selection system (Sage Science, Beverly, MA, USA) was used for library size selection. DNA molecules from the largest individual were sequenced using the PacBio and Illumina platforms for genome assembly, and other samples were subjected to ~~the~~ short-read whole-genome resequencing on the Illumina platform.

RNA extraction and sequencing library construction

RNA sequencing data provide~~s~~ ~~most~~ important evidences for gene prediction in the genome [28]. To include as many expressed genes as possible, 12 tissue types, mentioned above, were used for the RNA sequencing library construction. RNA was isolated from the 12 tissue samples using ~~TRIZOL~~ TRIzol reagent (Invitrogen, USA). The quantity and quality of extracted RNA were determined using the Nanodrop ND-1000 spectrophotometer (LabTech, Holliston, MA, USA) and 2100 Bioanalyzer (Agilent Technologies, Palo Alto, CA, USA). Samples with a total RNA concentration ≥ 10 μg, and RNA integrity number ≥ 8 were used for sequencing ~~experiments~~. RNA molecules extracted from tissues were ~~equally~~ mixed in equal proportions for the following RNA library construction. RNA sequence library was constructed following~~under~~ the ~~guidance of~~ protocol of ~~the~~ Paired-End Sample Preparation Kit (Illumina Inc., San Diego, CA, USA), which was identical to that employed in our previous study [29].

DNA and RNA library sequencing

The short-read DNA and RNA sequencing libraries were sequenced with the 150 bp paired-end (150PE) mode using the Illumina HiSeq X Ten platform (Illumina Inc.).

The 20_kb long-read genome DNA SMRT–bell libraries sequencing library was sequenced with the PacBio SEQUEL platform (Pacific Biosciences). The raw sequencing data ~~was~~ were quality checked before the bioinformatics analysis. The high-throughput quality control (HTQC v0.90.8) package [30] was used to filter low-quality bases and reads, and ~~the~~ sequences with adapters or low quality (average quality score < 20) were removed.

Genome size estimation

The genome size was estimated based on Illumina sequencing data using the *K*mer method before genome assembly. Raw Illumina reads were processed to remove adapter sequences, ~~the~~ reads with more than 10% N bases, and ~~the~~ reads with more than 50% ~~low~~ low-quality bases (≤5). All filtered reads were used for *K*mer frequency analysis [31]. Using *K*mer size of 17, the *K*mer frequencies were obtained using ~~jellyfish~~ Jellyfish v2.0 software [32]. *K*mers with a frequency of lower than 3 were eliminated as those likely resulted from sequencing errors. The genomic size was estimated based on the following formula: $G = (L - K + 1) \times n_{base} / (C_{Kmer} \times L)$, in which G is the estimated genome size, n_{base} is the total count of bases, C_{Kmer} is the expectation of *K*mer depth, L indicates the read length, and K represents *K*mer size. ~~The calculated genome size required further revision, since *K*mers with a depth lower than three likely resulted from sequencing errors.~~ The revised genome size was calculated as follows: Revised Genome size = Genome size × (1-Error Rate).

***De novo* assembly of the *T. bleekeri* genome**

Long reads generated from the PacBio sequencing platform were used for ~~*T.*~~

~~bleekeri~~ *T. bleekeri* genome assembly with the Falcon v0.3.0 package [33]. The assembled genome sequences were further polished with Arrow using long-read sequencing [34]; thereafter, two rounds of polishing using next-generation sequencing (NGS) short reads were performed with Pilon v1.23 [35]. Finally, redundant genomic sequences were eliminated using Redundans v0.14a with the parameter overlap of 0.95 and an identity of 0.95 [36]. ~~The c~~Completeness of the assembled genome was evaluated using BUSCO ~~version~~ v3.0 [37]. The database of actinopterygii_odb9 was used for the BUSCO analysis.

Chromosome assembly using Hi-C technology

Muscle tissue (1 g) of *T. bleekeri* was collected ~~from the above fish~~ for PacBio sequencing and was used for Hi-C library construction. The Hi-C processes, including crosslinking, lysis, chromatin digestion, biotin marking, proximity ligations, crosslinking reversal, and DNA purification, were performed using the protocol described in ~~as~~ previous studies [38]. The purified and enriched DNA was used for sequencing library construction. The library was sequenced using the Illumina HiSeq X Ten platform (Illumina), and the short-reads were then mapped to the polished genome of *T. bleekeri* with Bowtie v1.2.2. The chromosomal assembly using interaction frequency matrix extracted from the Hi-C read mapping ~~were~~ was performed according to a previously-reported methodology [38].

Repetitive element annotation

The *de novo* prediction and homology prediction were combined to annotate the repetitive sequences in the *T. bleekeri* genome. RepeatModeler v2.0.1

~~(http://www.repeatmasker.org/RepeatModeler.html)~~[39] was used for the detection of *de novo* repetitive elements ~~detection~~ in the *T. bleekeri* genome. The detected genome repeats were combined with ~~repBase~~ RepBase library [40], as a comprehensive library for the final repetitive elements prediction in the *T. bleekeri* genome, using the Repeat Masker v4.1.1 software [41]. Transposons were predicted using ~~the~~ ProteinMask, and the tandem repeats were identified in the genome using Tandem Repeat Finder v4.10 [42].

Protein coding and non-coding gene prediction

The ~~*de novo*~~ *ab initio* prediction, homology prediction, and RNA-sequencing-based methods were used for protein-coding gene annotation. Gene models for protein-coding genes were first predicted in the *T. bleekeri* genome using Augustus v2.5.5 [43]. Five closely related fish species, ~~including~~ viz., common carp (*Cyprinus carpio*) ~~(*C. carpio*)~~, zebrafish (*Danio rerio*) ~~(*D. rerio*)~~, Japanese medaka (*Oryzias latipes*) ~~(*O. latipes*)~~, green spotted puffe (*Tetraodon nigroviridis*) ~~(*T. nigroviridis*)~~, and threes pined sticklebacks (*Xiphophorus maculatus*) ~~(*X. maculatus*)~~, were used for the ~~Homologh~~homology-based protein-coding gene prediction. ~~The public p~~Protein sequences from those species, available in public databases, were mapped to the genome using ~~the~~ TBLASTN ~~utility~~ [44] and GeneWise [45]. Thereafter, comprehensive transcriptome sequencing data for multiple-tissues were aligned to the genome, and gene models were generated using the TopHat v2.1.1 package [46] and Cufflinks v2.2.1 [47]. The integration and redundancy elimination for the gene models predicted using the above methods were performed using the MAKER package [48,

49]. We only selected genes with start and stop codons, and genes with internal stop codons were removed. Only genes with completed sequences and 70% overlaps among different gene model prediction methods will be retained as high-quality gene models.

Four types of non-coding RNAs, including microRNAs (miRNA), transfer RNAs (tRNA), ribosomal RNAs (rRNA), and small nuclear RNAs (snRNA), were also predicted in the *T. bleekeri* genome using tRNAscan-SE v1.3.1 [50] and using Infernal v1.1.3 [51] with the Rfam database [52].

Functional annotation of protein-coding genes

The NCBI non-redundant protein, Swissport, and TrEMBL databases [53] were used as protein databases for the biological function annotation using BLAST v2.10.1 packages [54]. The E-value of 1e-5 was used as the threshold for homolog identification. Gene Ontology (GO) [55] and ~~the~~ Kyoto Encyclopedia of Genes and Genomes (KEGG) [56] assignments were performed using Blast2GO software [57].

Gene family clustering and phylogenetic analysis

Coding sequences annotated from ~~whole~~ whole-genome sequences for the closely related species were extracted from genome sequences. Gene family clustering was performed for *T. bleekeri* with ~~those~~ eight fish species living in non-QTP regions, viz., zebrafish, Japanese medaka, ~~including~~ elephant shark (*Callorhinchus milii*) ~~(*C. milii*)~~, spotted gar (*Lepisosteus oculatus*) ~~(*L. oculatus*)~~, Atlantic cod (*Gadus morhua*), ~~(*G. morhua*) *D. rerio, O. latipes*~~ platyfish (*X. maculatus*), tiger puffer (*Takifugu rubripes*), ~~(*T. rubripes*), *T. nigroviridis*,~~ and large yellow croaker (*Larimichthys corcea*) ~~(*L. corcea*), *Gasterosteus aculeatus* (*G. aculeatus*)~~ by the Orthomcl v1.2 pipeline [58] with default

settings. The single-copy orthologs across all species were selected for gene family, phylogenetic, and evolutionary analys~~i~~es. Briefly, proteins of these genes were aligned with ~~muscle~~ MUSCLE v3.8.31 [59] and were then transformed into alignments of nucleotide sequences with pal2nal [60] on the basis of the corresponding coding sequences. Next, non-conservative regions were removed using Gblocks [61] with default settings, and the conservative regions were concatenated and fed in Rax~~ml~~ML v8.2.10 [62] to deduce the phylogenetic relationships of these species using a GTRGAMMA model. Rapid bootstrap runs (100 times) were performed to test the robustness of the topology [63]. Based on the topology and the alignment matrix, their divergence times were deduced using MCMCTREE included in the PAML v1.3.1 package [64] with calibration points set by consulting the TimeTree database. *Danio rerio* and *Larimichthys crocea* (255-205 Ma), *Oryzias latipes* and *Larimichthys crocea* (115-105 Ma), *Lepisosteus oculatus* and *Danio rerio* (338-291 Ma) and *Callorhinchus milii* and *Danio rerio* (497-450 Ma) as calibration points for the divergence time estimation for other species.

To investigate the evolutionary relationship of species in *Triplophysa* genus, we also added another four *Triplophysa* genus fish species to the phylogenetic analysis. Since the genome of *T. xichangensis* and *T. scleroptera* have not been reported, we downloaded the short-reads of the transcriptomes of those two species from the NCBI sequence read archive (SRA) and conducted *de novo* assembly using Trinity v2.11.0 [65] with default settings. Longest transcripts for each gene were used in the following phylogenetic analysis. The single-copy orthologs across all species were used for

phylogenetic tree reconstruction and divergence time estimation following the same method as described above.

Gene family expansion and contraction in the *T. bleekeri* genome

To identify expanded and contracted gene families in the *T. bleekeri* genome, we compared gene families in the *T. bleekeri* genome to ~~*T. tibetana, T. siluroides, and*~~ those fish species living in non-QTP regions viz., including elephant shark, spotted gar, zebrafish, Japanese medaka, platyfish, tiger puffer, large yellow croaker, Atlantic cod, green spotted puffe, and threes pined sticklebacks. ~~, as in gene family clustering analysis. CAFÉ~~CAFE v4.2.1 [66] was used to analyze the expansion and contraction of gene clusters in the *T. bleekeri* genome using a probabilistic model. A GO enrichment analysis was performed on expanded and contracted genes using the topGO v2.40.0 package [67]. The enrichment of genes in KEGG pathways was also analyzed using the KOBAS v1.2.0 [68].

Positively selected genes (PSGs) in ~~the *T. bleekeri*~~ genomes of *Triplophysa* species

MUSCLE v3.8.31 was used for multi-protein sequence alignment among the *T. bleekeri* genes and their orthologs, and ~~comparing~~ compared to ~~the~~ eight fish species living in non-QTP regions used in the gene family clustering analysis. Conserved coding sequence (CDS) alignments of each single-copy gene family were extracted using Gblocks [69] and used for further identification of PSGs. The ratios of nonsynonymous to synonymous substitutions (K_A/K_S, or ω) were estimated for each single-copy orthologous gene using the CodeML program with the branch-site model as implemented in the PAML package. A likelihood ratio test was conducted, and the

false discovery rate (FDR) correction was performed for multiple comparisons. Genes with a corrected *P*-value < 0.05 were defined as PSGs. ~~To compare the natural selected genes for *T. tibetana* and *T. siluroides*, the natural selected genes~~ The genes putatively influenced by positive natural selection of *T. tibetana* and *T. siluroides* were also identified using the identical method. The functional annotation of PSGs for *T. bleekeri*, *T. tibetana*, and *T. siluroides* was also conducted using the same approach with the gene family expansion and contraction analysis.

~~Evolutionary relationships and divergence time for *T. bleekeri* with closely-related fish species~~

~~To investigate the evolutionary relationship of *T. bleekeri* to closely-related fish species, we added another four *Triplophysa* genus fish species to the phylogenetic analysis. Since the genome of *Triplophysa xichangensis* and *Triplophysa scleroptera* have not been reported, we downloaded the short-reads of the transcriptomes of those two species from the NCBI sequence read archive (SRA) and conducted *de novo* assembly using Trinity~~ [65] ~~with default settings. The assembled transcriptome sequences were also used in the analysis. The single-copy orthologs across all species were used for phylogenetic tree reconstruction and divergence time estimation.~~

Whole-genome re-sequencing and population genetics

~~Genetic structures for *T. bleekeri* populations~~

Raw reads of samples subjected to resequencing were quality controlled as ~~afore~~mentioned previously. ~~Thereafter, the~~ The filtered short reads were mapped using ~~bowtie2~~ BWA mem v0.7.17-r1188 with default settings for each individual, followed

by the marking of duplicates with Picard. Regions near INDELs were thought to be poorly aligned and were identified and realigned using GATK v4.1.8.1 [70]. GATK was also used to call SNPs and INDELs based on the alignments. The SNPs and INDELs were then filtered by these parameters: QUAL (phred quality) > 30, QD (quality score divided by depth to comprehensively evaluate the quality and depth) > 2, DP (read depth)> 5, FS (phred-scaled p-value using Fisher's Exact Test to detect strand bias for reads) < 60, MQ (mapping quality to evaluate read alignment) >40, SOR (strand odds ratio to evaluate strand bias for reads) < 4.0. The identified SNPs were filtered using SNPhylo v20180901 [71] ~~v20180901~~ with default settings, except for ~~the~~ LD_threshold and Minimum_depth_of_coverage, which were set to 0.8 and ~~3~~ 5, respectively. Next, the principal components analysis (PCA) clusters and population structure for these individuals were deduced with ~~plink~~ Plink v1.9 [72] and Admixture [73, 74] with default settings, respectively. Their phylogenetic relationships were recovered using the neighbor-joining (NJ) method with MEGA4 [74], and bootstrap resampling (100 times) ~~was performed to test the topology robust~~ was performed to test the robustness of the tree topology.

Historical effective population size inference for *T. bleekeri*

Historical effective population size of *T. bleekeri* was estimated based using Pairwise Sequentially Markovian Coalescent (PSMC) v0.6.5 software [75].We used the data for whole-genome variants of individuals for the genome assembly. The consensus sequences were generated using vcfutils.pl (vcf2fq -d 10 -D 300). The fq2psmcfa tool was used to create the input file for PSMC modelling. Sequences were used as the input

for the PSMC estimates using 'psmc' with the options -N25 -t15 -r5. The reconstructed population history was plotted using 'psmc_plot.pl' with the generation time of 2 year and rate of 4 × 10^{-9} substitutions per synonymous site per year. The mutation rate was estimated from the gene comparison of *T. bleekeri* and *D. rerio*. Bootstrapping was conducted by randomly sampling with replacement 5- Mb sequence segments and 100 bootstrap replicates were performed.

Selection sweep analysis for populations

To identify genome-wide selective sweeps among populations, we calculated the genome-wide distribution of fixation index (F_{ST}) values and θπ ratios using SNPs from different populations. The F_{ST} values were Z-transformed as follows: Z (F_{ST}) = (F_{ST} - μF_{ST}) / σF_{ST}, in which μF_{ST} was the mean F_{ST}, and σF_{ST} was the standard deviation of F_{ST}. The θπ ratios were log2-transformed. Subsequently, we scanned the genome in a 1 kb sliding scale, and estimated and ranked the empirical percentiles of Z (F_{ST}) and $\log_2$ (θπ ratio) in each window. We considered the windows with the top 1% Z (F_{ST}) and $\log_2$ (θπ ratio) as candidate outliers under strong selective sweeps. Genes residing in the outlier regions were considered as the candidate functional genes. The GO and KEGG enrichment were carried out by cluster Profiler v3.14.3 [76] and DAVID v6.8 [77].

Results

DNA and RNA library sequencing

81.69 Gb genomic (~120X) and 10.6 Gb ~~transcriptome~~ transcriptomic short-reads were generated for the following genome size estimation and annotation (**Table 1**). ~~Meanwhile, we~~ We also obtained 100.87 Gb genomic long-reads from the PacBio

platform, with a rough coverage of 160X for the *T. bleekeri* genome (**Table 1**). The mean and N50 length of the long-reads were 5.8 kb and 16 kb, respectively (**Table 1** and **Supplementary Fig. S1**).

Genome size estimation

To determine the possible sample contamination, 10,000 NGS short-reads were randomly selected for an NCBI nt database search. *Cyprinus*, *Danio*, and *Sinocyclocheilus* represent the top three sources of best hits, ruling out the obvious contamination during library construction and sequencing. Using genomic short-reads generated from the Illumina platform, ~~a total of~~ 59.8 million *K*mers were generated. The genome of *T. bleekeri* was estimated as 632.5 Mb, with a heterozygosity ratio of 0.26% and repeat content of 42.2% (**Supplementary Fig. S2**~~**Fig. 3**~~). Based on the above genome character estimation, the genome of *T. bleekeri* was mid-sized with low heterozygosity.

De novo assembly of the *T. bleekeri* genome

Using genomic PacBio long-reads for *T. bleekeri*, we assembled a 628 Mb genome with 856 contigs and an N50 length of 3.82 Mb (**Table 2**). Among these contigs, the longest contig for the genome was 15.5 Mb~~ (**Table 2**)~~. The completeness of the assembled genome was evaluated using BUSCO v3.0 [37] with the actinopterygii_odb9 database, indicating that 92.9% of BUSCO genes were identified in the assembled genome (**Supplementary Fig. S3~~2~~**).

Chromosome assembly using Hi-C technology

Hi-C technology recruits interaction information among different chromosome regions and assumes that the interactions for nearby regions are more prevalent than distant regions. In this study, ~~a total of~~ 82.9 Gb sequencing data were obtained via Hi-C library sequencing. Based on the interacting information, a chromosome assembly of 628 Mb with a scaffold N50 length of 22.9 Mb was obtained (**Supplementary Fig. S4~~3~~**). More than 596.9 Mb sequences were anchored upon 25 chromosomes, highlighting a high chromosome anchoring rate of 96.2% on the base level.

Repetitive element annotation

The annotation pipeline showed that more than 17.9 Mb of the genome sequences were predicted as tandem repeats, covering about 2.8% of the genome, and finally ~~a total of~~ 203.2 Mb, accounting for roughly 32.4% of the genome, were annotated as repetitive elements in the *T. bleekeri* genome (**Supplementary Table S1**). Specifically, there are 17.2% DNA transposons (107.8 Mb), 5.8% of long interspersed nuclear elements (LINE) (36.4 Mb), 0.68% short interspersed nuclear elements (SINE) (4.3 Mb), and 6.93% long terminal repeats (LTR) (43.5 Mb).

Protein- and non-coding gene prediction, and functional annotation

For predicting protein-coding genes in the *de novo* assembled genome, ~~a total of~~ 10.6 Gb short-read transcriptome data from 12 tissues was generated ~~transcriptome data were generate from 12 tissues~~. Based on the *de novo*, homolog, and RNA-seq data methods, a total of 20,274, 27,243, and 15,875 protein-coding genes were predicted, respectively. After integration and redundancy elimination, 21,198 protein-coding genes were predicted in the *T. bleekeri* genome (**Supplementary Table S2**).

Of the 21,198 protein-coding genes, roughly 93.0%, 96.9%, and 90.9% displayed homologous sequences in the NCBI NR, TrEMBL, and Swissprot databases, respectively. Additionally, 89.2% contained InterPro domains, and 46.9% were assigned with GO terms. Overall, more than 97.3% of the protein-coding genes were functionally annotated by at least one method (**Supplementary Fig. ~~S4~~S5**). ~~Meanwhile, the important function of the non-coding genes received further attention.~~ The non-coding genes have received increased attention in the recent year, since accumulating evidence suggests that many of them play crucial roles in a variety of biological process. ~~Based on the *de novo* prediction strategies,~~ In this study, all the possible ~~non-coding gene loci~~ non-coding DNA sequences were predicted based on the *de novo* prediction strategies, and ~~the summary is listed~~ are summarized in **Supplementary Table S3**.

~~Gene family expansion and contraction in the *T. bleekeri* genome~~

~~Following the Orthomcl pipeline, a total of 21,862 ortholog groups were obtained after gene family clustering with those fish species from non-QTP regions. A total of 1,533 significantly expanded and 2,401 significantly contracted gene families were observed for *T. bleekeri*, compared to other fish species (**Supplementary Fig. S5**). The functional enrichment of expanded gene families using was analyzed using GO and KEGG. The expanded gene families were primarily enriched in categories of metabolism and immune regulation (**Supplementary Tables S4** and **S5**). The category of metabolism includes fatty acid metabolism (arachidonic acid metabolism and glycosphingolipid biosynthesis), carbohydrate metabolism (glycosaminoglycan biosynthesis and glycan degradation), and amino acid metabolism (RNA transport). The~~

~~category of immune regulation includes the Hippo signaling pathway (corrected *p* value = 2.40E-03), necroptosis, and Vitamin B6 metabolism (corrected *p* value= 8.90E-03). The contracted gene families were mainly made up of several signaling pathways, including the MAPK signaling pathway, calcium signaling pathway, adrenergic signaling in cardiomyocytes, GnRH signaling pathway, and retrograde endocannabinoid signaling .~~

Gene family clustering and phylogenetic analysis of *T. bleekeri*

Using the whole-genome and transcriptome data of four other *Triplophysa* species, viz., *T. tibetana*, *T. siluroides*, *T. scleroptera*, and *T. xichangensis*, and the eight other fish species living in non-QTP regions, we performed the gene family clustering for those species. As a result, we identified 1, 364 single-copy orthologs among those fish species.

We then investigated the evolutionary relationship of *T. bleekeri* with respect to other *Triplophysa* and the non-QTP species. Using single-copy genes among species, a concatenated alignment matrix, was generated with a total length of 73,887 bps, which was used for the phylogenetic analysis and divergence time estimation. The result showed that *Triplophysa* species are phylogenetically closer to *D. rerio*, and that *T. siluroides* is a basal species within the *Triplophysa* group. Divergence time estimation showed that *T. bleekeri* diverged from their common ancestor, *T. scleroptera* and *T. xichangensis*, around 25.2 million years ago (Ma) (**Fig. 2**).

~~*T. bleekeri* g~~Genes under natural positive selection

We identified 788 ~~positive selected genes (~~PSGs~~)~~ in the *T. bleekeri* genome. The

functional analysis on the KEGG and GO parameters~~terms~~ showed that several categories associated with nucleotide metabolism and DNA repair~~s, including single strand break repair, DNA repair, base excision repair,~~ were significantly enriched (**Supplementary Table S4 and S5~~6~~**). Additionally, the PSGs were also enriched in immune response, such as MyD88-dependent toll-like receptor signaling pathway ~~biological pathways of non-homologous end-joining, fanconi anemia pathway, pyruvate metabolism, meiosis~~(**Supplementary Table S4~~7~~**). Concomitantly ~~Meanwhile~~, 969 and 1,253 PSGs were identified for *T. tibetana* and *T. siluroides*, respectively. Among those genes, 197 genes were identified as shared PSGs for the three *Triplophysa* species ~~Among those genes, 197 genes were identified as shared PSGs for three *Triplophysa* species~~ (**Fig. ~~4a~~3a**)~~, indicating those functional genes might be naturally selected in their common ancestor.~~

To detect candidate PSGs for *Triplophysa* ancestral lineage, we also performed the PSG identification for the common ancestor of the *Triplophysa* with the branch-site model in the PAML. As a result, we identified 439 PSGs for *Triplophysa* ancestral lineage. Interestingly, we found that only 35 shared PSGs for the three *Triplophysa* species were identical with *Triplophysa* lineage PSGs (**Fig. 3b**). The functional analysis with respect to biological pathways for the three *Triplophysa* species showed that those genes were significantly enriched for various processes including protein digestion and absorption, Fanconi anemia pathway, and salivary secretion (**Fig. 3c**). Twenty-five biological pathways, including peroxisome, autophagy, non-homologous end-joining, homologous recombination, basal transcription, ribosome biogenesis and spliceosome,

were enriched for PSGs of *Triplophysa* ancestral lineage (**Fig. 3c**). The homologous recombination and basal transcription factor pathways were both enriched for *Triplophysa* lineage PSGs and the three *Triplophysa* species shared PSGs (**Fig. 3c**).

~~The functional analysis with respect to biological pathways for the shared genes showed that those genes were significantly enriched on the protein-digestion, salivary secretion, fanconi anemia pathways (Fig. 4b).~~

Gene family expansion and contraction in the *T. bleekeri* genome

Following the Orthomcl pipeline, 21,862 ortholog groups were obtained after gene family clustering with ten fish species from non-QTP regions. Gene family analysis showed that 1,533 and 2,401 gene families were significantly expanded and contracted in *T. bleekeri*, respectively (**Supplementary Fig. S6**). The functional enrichment of expanded gene families was analyzed using GO and KEGG. The expanded gene families were primarily enriched in categories of metabolism and immune regulation (**Supplementary Tables S6** and **S7**). The categories of metabolism include fatty acid metabolism (arachidonic acid metabolism and glycosphingolipid biosynthesis), carbohydrate metabolism (glycosaminoglycan biosynthesis and glycan degradation), and amino acid metabolism (RNA transport). The categories of immune regulation include the Hippo signaling pathway (corrected *p*-value = 2.40E-03), necroptosis, and Vitamin B6 metabolism (corrected *p*-value = 8.90E-03). The contracted gene families were mainly made up of several signaling pathways, including the MAPK signaling pathway, calcium signaling pathway, adrenergic signaling in cardiomyocytes, GnRH signaling pathway, and retrograde endocannabinoid signaling (**Supplementary Tables**

S8 and **S9**).

Historical effective population size for *T. bleekeri* during the QTP formation

We used the whole-genome short-read sequencing data of the sample for genome assembly to obtain the genome-wide heterozygotic genotypes. Then, those variants were used to probe the profiles of historical effective population size for *T. bleekeri* during the QTP formation. We used the gene comparison between *T. bleekeri* and *D. rerio* to estimate the mutation rate. As a result, we estimated the mutation rate of 4×10^{-9} for *T. bleekeri*. PSMC analysis performed using the above data showed that the effective population size of *T. bleekeri* increased more than 0.7 Ma, and reached a peak of 70×10^{4} around 0.6–0.7 Ma. However, the *T. bleekeri* population size experienced a dramatic drop afterwards to 1×10^{4} from 0.6 Ma to 60, 000 years ago (**Fig. 4**). The effective population size decline was consistent with the accelerating QTP uplifts around 1 Ma [78] and the quaternary glaciation spaning the Pleistocene (2.6–0.11 Ma) and Holocene (0.11–0 Ma) [19, 79]. We speculate that both the geotectonic movements and temperature fluctuations during the period exerted intense survival pressure for the ancient *T. bleekeri* populations, leading to the roughly 70 times effective population size drop during the period.

~~**Phylogenetic relationships of *T. tibetana* with other fish species**~~

~~Using the genome and transcriptome of other *Triplophysa* species, including *T. tibetana*, *T. siluroides*, *T. scleroptera*, and *T. xichangensis*, we investigated the evolutionary relationship of *T. bleekeri* with respect to other fish species. Using single copy genes among species, a concatenated alignment matrix, using those single copy~~

~~orthologs, was generated with a total length of 73,887 bps, which was used for the phylogenetic analysis and divergence time estimation. The result showed that *Triplophysa* species formed a sister group with *D. rerio*. The *T. siluroides* was a basal species in the *Triplophysa* group, which was consistent with previous studies that *T. siluroides* was an ancient *Triplophysa* species. Divergence time estimation showed that *T. bleekeri* diverged from their common ancestor of *T. scleroptera* and *T. xichangensis* about 25.2 Million years ago (**Fig. 5**).~~

Population genetics ~~structure~~ analysis of *T. bleekeri*

The high-quality SNPs were obtained according to ~~serious~~ the filtering criteria set previously, and were used to deduce the population structures of *T. bleekeri*. As a result, more than 34 million short-reads were obtained for 28 individuals, and more than 3 million~~a total of 3,025,149~~ SNPs were detected for all individuals. The phylogeny reconstruction analyses based on whole-genome SNPs showed that individuals from population~~ 1 and 2 formed a sister group, with all 11 individuals from population 1 forming a monophyletic group and nine individuals from population 2 forming another monophyletic group. Six individuals from population 3 formed the third monophyletic group~~ LHK and XX clustered together forming two neighboring groups, whereas individuals from population BY formed another cluster (**Fig. ~~6~~5a**). The PCA clusters (**Fig. 5b**) also suggested that the first two principal components could successfully separate the individuals in population BY from those in population LHK and XX. In addition, genetic structure analysis also indicated that gene flow between population BY and the other two populations was might be limited (**Fig. 5c**).

Structure analysis also indicated that genes flow between population 3 and the other two populations are limited (**Supplementary Fig. S6**). In addition, the PCA clusters (**Supplementary Fig. S7**) suggested that the first two components could successfully separate the individuals in population 3 from those in population 1 and 2.

To investigate the possible natural selection among populations, we also performed the selective sweep analysis for BY, LHK and XX populations (**Fig. 6a**). Based on Fst comparison among those populations (**Supplementary Table S10**), we identified genomic regions (~1 kb in length) that scored in the top 1% (**Supplementary Fig. S7**). As a result, 1, 734, 3, 009, and 3, 244 regions (1 kb), harboring 474, 878, and 957 functional candidate genes were identified to be significantly genetically differentiated for LHK-XX, LHK-BY, and XX- BY comparisons, respectively. Genomic regions with less differentiation identified in LHK-XX comparison were consistent with the above phylogenetic analysis. The GO and KEGG pathway functional analyses showed 20, 25, and 31 significant biological pathway enrichments for LHK-XX, XX-BY, and LHK-BY comparisons, respectively (**Fig. 6b**, **Supplementary Table S11, S12, and S13**). Six enriched biological pathways were shared in LHK-BY and XX-BY comparisons but not in LHK-XX one, viz., ubiquitin mediated proteolysis, tight junction, starch and sucrose metabolism, melanogenesis, longevity regulating pathway – mammal, and circadian rhythm (**Fig. 6c, Supplementary Table S12 and S13**). Five enriched biological pathways were shared for all comparisons, viz., axon guidance, long-term potentiation, Rap1 signaling pathway, circadian entrainment, and calcium signaling pathway (**Fig. 6b**).

Discussion

~~The *Triplophysa* species compose the predominant fish taxon in the drainages of the QTP. The wide range of altitudes for their distribution makes the species an excellent model for genome evolution and adaption studies. The genomic resource of *T. bleekeri* would not only help us to better understand its biological evolution and adaptation mechanisms, but also provide valuable conservation genetics data for species protection.~~

In this study, we presented the chromosome-level genome assembly of *T. bleekeri* with a contig N50 of ~~3.82~~3.1 Mb and a scaffold N50 of 22.9 Mb. The N50 lengths of contigs of *T. bleekeri* genome assembly were much longer than previously reported genome assemblies of *T. tibetana* [17]. Twenty-five chromosomes were obtained with the mounting rate up to 96.2%, and the assembled chromosome number was consistent with the karyotype of *T. bleekeri* (unpublished data), which suggests that the present analysis resulted in successful assembly of *T. bleekeri* genome to the chromosome level. The completeness ~~and chromosome anchoring ratio~~ of the genome ~~were~~ was also evaluated, confirming the high quality of the assembled *T. bleekeri* genome. The combined results of the homology-based and *de novo* predictions showed that repetitive sequences accounted for 32.4% of the genome. Among them, DNA transposons represented the most abundant tandem repeats, which ~~were similarly~~ was also observed in ~~other teleosts, such as~~ *T. tibetana* [17]~~, *Epinephelus akaara* (*E. akaara*), and *Epinephelus lanceolatus* (*E. lanceolatus*)~~. Within the genome, ~~a total of~~ 21,198 protein-coding genes were predicted, of which 97.3% could be functionally annotated. Overall, this genome assembly and annotation ~~can~~ provides valuable data to the genomic

resources currently available for the study of phylogeny and environmental adaptations of *Triplophysa* species.

The phylogenetic analysisphylogeny results indicated that the *Triplophysa* family genus formed a clade with *D. rerio*, and that *T. bleekeri* was most closely related to *T. tibetana* and *T. xichangenis* *T. scleroptera*. The divergence time estimation indicated that *T. siluroides* diverged from their common ancestor roughly 38.8 Ma, suggesting that *T. siluroides* is a more primitive species compare with other species occupying a basal position in the *Triplophysa* genus. The extensive QTP was elevated by more than exceeding 4,000 m about 40 Ma [80], and this time is consistent with the divergence of *T. siluroides*. Therefore, we speculated that the speciation of *Triplophysa* was likely triggered enormously influenced by the upliftinguplifts of the QTP [81].

Upliftment of the QTP induced profoundly induced impacts on climatic and environmental changes of the plateau and its peripheraladjacent regions, including low oxygen and low temperature [82]. The oxygen content of air is inadequate in the QTP, In fact, an investigation while investigations into water quality indicated that high dissolved oxygen concentration exists in the QTP water [83-86]. Therefore, we speculated that thermal stress may present a major factor in natural selection for fish species in the QTP and its peripheral regions. Although *Triplophysa* species are widely distributed in different regions, these regions are all generally characterized by a cold environment [23, 87]. However, to the best of our knowledge, only a few studies have been conducted to explore the genetic basis of adaptation of *Triplophysa* species to low temperatures. have addressed the mechanism by which *Triplophysa* species adapt to

low temperatures. Through the comparative analysis of the genome with other fish species, we found that the expanded gene families of *T. bleekeri* were significantly ($p < 0.05$) enriched in fatty acid metabolism, including glycosphingolipid biosynthesis and arachidonic acid metabolism pathways. The glycosphingolipid located in the bilayer lipid membrane is a major structural component of cell membranes [88], whereas Arachidonic arachidonic acid, an integral constituent of biological cell membranes, aids in the maintenance of cell membrane fluidity even at low temperatures [89]. Our Results results suggest that the increased number of genes related to arachidonic fatty acid metabolism might be responsible for maintaining membrane structure and improving membrane fluidity under cold environments.

In the genome of *T. bleekeri*, significant expansion was also observed in Hippo signaling pathway gene family which participates in regulating innate immunity [90, 91]. These results suggest that *T. bleekeri* may tend to increase gene numbers in certain families related to immune response for improving the defense against pathogens. It is notable that genes involved in innate immunity, such as toll-like receptor signaling pathway genes, all underwent positive selection in *T. bleekeri*, *T. tibetana*, and *T. siluroides*. Similar results were also observed in previous transcriptomic studies of Tibetan Schizothoracinae species, *Gymnocypris przewalskii*, and *G. przewalskii ganzihonensis* [92, 93]. These results indicated that the adaptive evolution of innate immunity might play crucial roles in the highland adaption of fish. Low temperatures could also induce the accumulation of reactive oxygen species (ROS) and ion leakage, causing cellular injuries and apoptosis, which eventually leads to cell deathIndeed, we

~~found that expanded gene families in the *T. bleekeri* genome were enriched in necroptosis and apoptosis, implying the biological requirement for *T. bleekeri* to live in cold environments. Genes were expanded in the notch signaling pathway, which is involved in regulating cell proliferation, cell differentiation, and cell death to maintain tissue self-renewaWhile ROS can injure the host cell, ROS play a central role in the development of the antimicrobial innate immune responses Our results also showed that many genes in the hippo signaling pathway, an essential signaling pathway to regulate innate immunity, were significantly expanded.~~

Low temperatures and UV radiation can cause DNA damage [94], and DNA damage response and repair pathways may show functional adaption. Within the three *Triplophysa* species~~Additionally~~, the PSGs ~~naturally selected genes~~ were ~~also~~ enriched in the functional categories of nucleotide excision repair, ~~mismatch repair, and base excision repair~~non-homologous end-joining, homologous recombination, and Fanconi anemia pathways (**Supplementary Table S4 and S5**). These pathways all participate in DNA repair, of which non-homologous end-joining and homologous recombination are the two main pathways for repairing double-strand break [95], and Fanconi anemia pathway is essential for the repair of DNA interstrand crosslinks [96]. PSGs influencing DNA repair may contribute to DNA integrity and genomic stability under high-altitude environments with low temperatures and intense UV radiation. Our results suggest that *Triplophysa* species have evolved an integrated DNA-repair mechanism to adapt to high-altitude environments. ~~Low temperatures, oxidative damage, and UV radiation can cause DNA damage.~~ The previous studies also showed that genes involved in the

DNA repair were under positive selection pressure in many species living at high altitudes, such as the snub-nosed monkey [97] and the Tibetan hot-spring snake [11]. It indicated that DNA damage caused by the environment is a common stress that animals in high-altitude regions need to cope with. ~~Many naturally selected genes influencing DNA repair may contribute to DNA integrity and stability for the *T. bleekeri* genome under extreme environments. Interestingly, natural positively selected genes for three *Triplophysa* species were all significantly enriched on fanconi anemia pathway, non-homologous end-joining and homologous recombination (**Supplementary Table S7**), indicating that *Triplophysa* species might be under similar natural selections. Meanwhile, we found~~ We also identified 197 ~~natural positively selected genes for~~ PSGs shared by the three *Triplophysa* species (**Fig. 3A**), indicating that those naturally selected genes might have originated from their common ancestor, and that *Triplophysa* species were genetically convergent on PSGs. ~~and those naturally selected genes might originate from their common ancestor or independent selections.~~ We ~~also indeed~~ found ~~massive~~ many species-specific PSGs ~~genes that uniquely naturally selected~~ for three *Triplophysa* species. The result ~~might imply~~ implies the requirement of ~~the~~ a distinct~~ion of the~~ ecological niche for ~~*Triplophysa*~~ *T. bleekeri*, *T. tibetana*, and *T. siluroides*. Based on the generally used genomic comparison methods, hundreds of PSGs for *Triplophysa* species were identified in this investigation. However, a previous study has shown that ancient demographic fluctuation could generate severe overestimation of selective signatures [98]. Therefore, PSG identification in this work might have been influenced by the demographic scenarios of *Triplophysa* species. It is

worth estimating the demographic fluctuation to PSG identification, and comparing the current methods for potential biases.

In addition to comparative genomics analyses, ~~The~~ the relationships among populations of *T. bleekeri* were analyzed to probe possible differences in genetic structures~~. The results showed that samples of population 1 and 2 formed a sister group, while samples of population 3 formed the third monophyletic group~~. Population structure analysis divided 28 *T. bleekeri* samples into two clusters, with individuals from the LHK and XX population grouped together, and individuals from BY population forming the other cluster. Both PCA and structure analyses corroborated these findings. The BY population was separated from the LHK and XX population, and the observed admixture of genetic lineages was limited (*K*=3). ~~This result~~ These results could be because ~~population 1 and 2~~ LHK and XX are directly connected by the river, and gene flow between individuals residing in the two places occurs more frequently. The difference between BY population ~~3~~ and the LHK and XX populations ~~1 and 2~~ might be attributed to the relatively limited gene flow caused~~hampered~~ by natural and artificial barriers among those populations. The Daning River measures a height of up to 1,648 m, which flows through many narrower canyons~~gorges, and river measures a height of up to 1,648 m~~ [99]~~,~~. ~~therefore~~Therefore, the geographical barriers formed by canyons and shallows could contribute to the diminished interaction~~exchanges~~ among those populations. More importantly, artificial barriers, such as cities and dams, could also weaken the migrations between the ~~population 3~~ BY and LHK/XX~~1/2~~ populations. Therefore, the whole-genome resequencing data of *T.*

bleekeri provided a valuable genetic resource to reveal that geographical and artificial barriers could distinctly influence genetic exchange among populations.

The selective sweep analysis showed that genomic differentiation of LHK-XX was nonintensive compared to that of the BY population, which is consistent with the above population phylogenetic analysis. Notably, we identified six shared enriched biological pathways for LHK-BY and XX-BY comparisons but not in LHK-XX. Among those pathways, melanogenesis, circadian rhythm, and starch and sucrose metabolism might be closely related to the living environment differences caused by the presence of gorges, as well as human activity. The natural gorge might change the water flow and biodiversity of environments, and human activity could as well influence the nutrition supplies and circadian rhythm for local fish populations directly. We found that genomic regions in chromosome 11 containing alpha-trehalose glucohydrolase (*treh*) gene exhibited significant genetic differentiation in LHK-BY and XX-BY comparison but not in LHK-XX, imply *treh* gene might be related to the living environment for BY (**Fig. 6d**). Previous studies showed that trehalose plays an important role as a protectant to preserve membrane organization and protein structure during abiotic stress, such as starvation and cold, and as a glycolytic input to maintain energy homeostasis [100]. Further, β-catenin (*ctnnb1*) and lymphoid enhancer-binding factor 1 (*lef1*) gene were also candidate functional genes related to local environment of XX because of gorges, artificial dam and human activity, since the genomic regions of those two genes for individuals in BY were significantly differentiated from those in LHK and XX (**Fig. 6d**). *Ctnnb1* has been reported as an important gene in colorectal

tumorigenesis [101] and the interaction of *ctnnb1* and *lef1* could influence the down-stream gene regulation of cell profiles and developments [102]. The multiple-functions of *ctnnb1* present it as promising gene in maintain physiology and adaptation formation. Interestingly, a previous study has showed that *ctnnb1* might contribute to the high altitude environments environment adaptation for Tibetan pig– [103].

This research serves as a preliminary population genetics analysis for *T. bleekeri*. Further research is required to determine how geographical and artificial barriers precisely influence gene exchange among populations.

In conclusion, we present a chromosomal-scale genome assembly of *T. bleekeri*, a representative high-altitude fish. Evolutionary, comparative, and population genomic analyses were performed to investigate the evolution, environmental adaption, and genetic diversity of *T. bleekeri*. Our results provide insights into how fish adapt to the high-altitude environment, and the genomic data serves as a valuable resource for further study on functional validation of candidate genes contributing to environmental adaption.

Conclusion

The QTP is the highest plateau world-wide and is characterized by low temperatures and hypoxia environment. The *Triplophysa* species is widely distributed, up to an elevation of 4,500 m. It is fascinating to investigate the molecular mechanisms for their environmental adaptation to the QTP. By combing multiple sequencing platforms and using the Hi-C technique, here we reported the first chromosomal genome for *T. bleekeri*. We obtained a genome of 628 Mb for *T. bleekeri*, containing

~~856 contigs with an N50 length of 3.82 Mb, with the longest contig of 15.5 Mb. More than 96% of bases were anchored upon 25 chromosomes, resulting in a scaffold N50 length of 22.9 Mb. A total of 203.2 Mb accounting for roughly 32.4% of the genome were annotated as repetitive elements, and 21,198 protein-coding genes were obtained eventually, and more than 97.3% of these genes can be functionally annotated. Gene families that underwent significant expansion and positive selected genes were enriched in pathways related to lipid metabolism, DNA repair, and immune response, implying the molecular requirements for adaptation of *T. bleekeri* to low temperatures and ultraviolet radiation. Population analyses suggested that genetic structures among *T. bleekeri* populations may be influenced by both geographical and artificial barriers. The genomic resource generated in this work and the analysis based on those data lay a solid foundation for further evolutionary environmental adaptation and conservation studies for *Triplophysa* and other highland fish species. However, fish are the most diversified group of vertebrates with enormous variation in their habitats and ecological niches as well as life history. Therfore, more genomic data of high land fish are needed to better understand their adaptive mechanism to the local environment.~~

Ethics Statement

All experimental protocols were approved by the School of Life Sciences, Southwest University (Chongqing, China), and the studies were carried out in accordance with the Guidelines of Experimental Animal Welfare from Ministry of Science and Technology of People's Republic of China (2006) and the Institutional Animal Care and Use Committee protocols from Southwest University (2007).

Acknowledgement

This work was supported by the Financial Program of Ministry of Agriculture and Rural Affairs of China (Grant No. YYJZHC201921301350063), National Natural Science Foundation of China (Grant No. 31602207), and Research Innovation Program for College Graduates of Chongqing (Grant No. CYB19079).

Author contributions

ZJ Wang conceived and designed the study; DY Yuan and SJ Xiao collected the samples; DY Yuan and SJ Xiao performed molecular experiments; SJ Xiao performed the bioinformatics analysis, including genome size estimation, genome assembly, annotation, and gene prediction; DY Yuan, SJ Xiao, and ZJ Wang wrote the manuscript. All authors read and approved the final manuscript for submission.

Competing interests

All authors declare that they have no competing interests.

Data accessibility

The genomic, transcriptome, and Hi-C sequencing reads generated from the PacBio and Illumina platforms are available in the NCBI SRA database under the Accession no. SRP200140. The final chromosome assembly was submitted to NCBI with the accession number of VFQW00000000.

Abbreviations

BUSCO: Benchmarking universal single-copy orthologs; CAFE: Computational analysis of gene family evolution; CDS: Conserved coding sequence; FDR: False

discovery rate; GO: Gene ontology; Hi-C: High-throughput chromosome conformation capture; KEGG: Kyoto encyclopedia of genes and genomes; LINE: Long interspersed nuclear elements; LTR: Long terminal repeats; QTP: Qinghai-Tibetan Plateau; PAML: Phylogenetic analysis by maximum likelihood; PCA, Principal components analysis; PSG: Positively selected genes; PSMC: Pairwise Sequentially Markovian Coalescent; SINE: Short interspersed nuclear elements; SRA: Sequence read archive.

References

1. Myers N, Mittermeier RA, Mittermeier CG, et al. Biodiversity hotspots for conservation priorities. *Nature*. 2000;403(6772):853.
2. Zhao Z and Li S. Extinction vs. Rapid radiation: The juxtaposed evolutionary histories of coelotine spiders support the Eocene–Oligocene orogenesis of the Tibetan Plateau. *Syst Biol*. 2017;66(6):988-1006.
3. Beall CM. Adaptation to high altitude: phenotypes and genotypes. *Annu Rev Anthropol*. 2014;43:251-72.
4. Monge C and Leonvelarde F. Physiological adaptation to high altitude: oxygen transport in mammals and birds. *Physiol Rev*. 1991;71(4):1135-72.
5. Wu T and Kayser B. High Altitude Adaptation in Tibetans. *High Alt Med Biol*. 2006;7(3):193-208.
6. Beall CM. Two routes to functional adaptation: Tibetan and Andean high-altitude natives. *Proc Natl Acad Sci U.S.A*. 2007;104:8655-60.
7. Ding CZ, Jiang XM, Chen L, et al. Growth variation of *Schizothorax dulongensis* Huang, 1985 along altitudinal gradients: implications for the Tibetan Plateau fishes under climate change. *J Appl Ichthyol*. 2016;32(4):729-33.
8. Deng H, Yue X, Chen D, et al. Growth characteristics and feed habit of *Triplophysa stenura* in Nujiang River. *Freshw Fisheries*. 2010;40(1):26-33.
9. Li M, Tian S, Jin L, et al. Genomic analyses identify distinct patterns of selection in domesticated pigs and Tibetan wild boars. *Nat Genet*. 2013;45(12):1431.
10. Qiu Q, Zhang G, Ma T, et al. The yak genome and adaptation to life at high altitude. *Nat Genet*. 2012;44(8):946.
11. Li JT, Gao YD, Xie L, et al. Comparative genomic investigation of high-elevation adaptation in ectothermic snakes. *Proc Natl Acad Sci U.S.A*. 2018;115(33):8406-11.
12. Liu ZJ, Liu SK, Yao J, et al. The channel catfish genome sequence provides insights into the evolution of scale formation in teleosts. *Nat Commun*. 2016;7:11757.
13. Sun YB, Fu TT, Jin JQ, et al. Species groups distributed across elevational gradients reveal convergent and continuous genetic adaptation to high elevations. *Proc Natl*

Acad Sci U.S.A. 2018;115(45):E10634-E41.

14. Wu YF and Tan QJ. Characteristics of the fish-fauna of the characteristics of Qinghai-Xizang plateau and its geological distribution and formation. *Acta Zool Sinica*. 1991;37:135-52.

15. Liu HP, Liu QY, Chen ZQ, et al. Draft genome of *Glyptosternon maculatum*, an endemic fish from Tibet Plateau. *GigaScience*. 2018;7(9):giy104.

16. Liu HP, Xiao SJ, Wu N, et al. The sequence and de novo assembly of *Oxygymnocypris stewartii* genome. *Sci Data*. 2019;6:190009.

17. Yang X, Liu H, Ma Z, et al. The chromosome-level genome assembly of *Triplophysa tibetana* , a fish adapted to the harsh high-altitude environment of the Tibetan plateau. *Mol Ecol Resour*. 2019;19(4):1027-36.

18. Yang L, Wang Y, Wang T, et al. A chromosome-scale reference assembly of a Tibetan loach, *Triplophysa siluroides*. *Front Genet*. 2019;10:991.

19. Xiao S, Mou Z, Fan D, et al. Genome of Tetraploid Fish Schizothorax o'connori Provides Insights into Early Re-diploidization and High-Altitude Adaptation. *iScience*. 2020;23(9):101497.

20. Nelson JS, Grande TC and Wilson MV. Fishes of the World. John Wiley & Sons; 2016.

21. He CL, Song ZB and Zhang E. *Triplophysa* fishes in China and the status of its taxonomic studies. *Sichuan J Zool*. 2011;30(1):150-5.

22. He XF, He JS and Yan TM. Reproductive characteristic of *Triplophysa bleekeri* in mabian river. *J Southwest China Norm Univ*. 1999;24(1):69-73.

23. Xiao H and Dai YG. A Review of Study on Diversity of *Triplophysa* in China. *Fisheries Sci*. 2011;30(1):53-7.

24. Wang ZJ, Huang J and Zhang YG. The reproductive traits of *Triplophysa bleekeri* in the Daning River. *Freshw Fisheries*. 2013;43(5):8-12.

25. Zhu S. The loaches of the subfamily Nemacheilinae in China (Cypriniformes: Cobitidae). Jiangsu Science and Technology Publishing House; 1989.

26. Wu YT, Tang QJ. Characteristics of the fish-fauna of the characteristics of Qinghai-Xizang Plateau and its geological distribution and formation. *Acta Zool Sinica*. 1991;2:135-152.

27. Xiao SJ, Wang PP, Dong LS, et al. Whole-genome single-nucleotide polymorphism (SNP) marker discovery and association analysis with the eicosapentaenoic acid (EPA) and docosahexaenoic acid (DHA) content in *Larimichthys crocea*. *PeerJ*. 2016;4:e2664.

28. Denoeud F, Aury J-M, Da Silva C, et al. Annotating genomes with massive-scale RNA sequencing. *Genome Bio*. 2008;9(12):R175.

29. Xiao SJ, Han ZF, Wang PP, et al. Functional marker detection and analysis on a comprehensive transcriptome of large yellow croaker by next generation sequencing. *PloS One*. 2015;10(4):e0124432.

30. Yang X, Liu D, Liu F, et al. HTQC: a fast quality control toolkit for Illumina sequencing data. *BMC Bioinformatics*. 2013;14(1):33.

31. Liu B, Shi Y, Yuan J, et al. Estimation of genomic characteristics by analyzing k-mer frequency in de novo genome projects. *arXiv: Genomics*. 2013.

32. Marcais G and Kingsford C. A fast, lock-free approach for efficient parallel counting of occurrences of k-mers. *Bioinformatics*. 2011;27(6):764-70.

33. Chin CS, Peluso P, Sedlazeck FJ, et al. Phased diploid genome assembly with single-molecule real-time sequencing. *Nat Methods*. 2016;13(12):1050.

34. Chin CS, Alexander DH, Marks P, et al. Nonhybrid, finished microbial genome assemblies from long-read SMRT sequencing data. *Nat Methods*. 2013;10(6):563.

35. Walker BJ, Abeel T, Shea T, et al. Pilon: an integrated tool for comprehensive microbial variant detection and genome assembly improvement. *PloS One*. 2014;9(11):e112963.

36. Pryszcz LP and Gabaldón T. Redundans: an assembly pipeline for highly heterozygous genomes. *Nucleic acids Res*. 2016;44(12):e113.

37. Simão FA, Waterhouse RM, Ioannidis P, et al. BUSCO: assessing genome assembly and annotation completeness with single-copy orthologs. *Bioinformatics*. 2015;31(19):3210-2.

38. Gong GR, Dan C, Xiao SJ, et al. Chromosomal-level assembly of yellow catfish genome using third-generation DNA sequencing and Hi-C analysis. *GigaScience*. 2018;7(11):giy120.

39. Smit A, Hubley R and Green P. RepeatModeler Open-1.0. 2008–2015. http://www repeatmasker.org. Accessed 1 May, 2018.

40. Jurka J, Kapitonov VV, Pavlicek A, et al. Repbase Update, a database of eukaryotic repetitive elements. *Cytogenet Genome Res*. 2005;110(1-4):462-7.

41. Tarailo- Graovac M and Chen NS. Using RepeatMasker to identify repetitive elements in genomic sequences. *Curr Protoc Bioinform*. 2009;25(1):4.10.1- 4.10.14.

42. Benson G. Tandem repeats finder: a program to analyze DNA sequences. *Nucleic Acids Res*. 1999;27:573.

43. Stanke M, Keller O, Gunduz I, et al. AUGUSTUS: ab initio prediction of alternative transcripts. *Nucleic Acids Res*. 2006;34(suppl_2):W435-W9.

44. Lobo I. Basic local alignment search tool (BLAST). *Nat Educ*. 2008;1(1).

45. Birney E, Clamp M and Durbin RJ. GeneWise and Genomewise. *Genome Res*. 2004;14(5):988.

46. Trapnell C, Pachter L and Salzberg SL. TopHat: discovering splice junctions with RNA-Seq. *Bioinformatics*. 2009;25:1105-11.

47. Ghosh S and Chan CK. Analysis of RNA-Seq Data Using TopHat and Cufflinks. *Methods Mol Biol*. 2016;1374:339.

48. Campbell MS, Holt C, Moore B, et al. Genome Annotation and Curation Using MAKER and MAKER-P. *Curr Protoc Bioinform*; 2014;48(1): 4.11.1-4.11.39.

49. Cantarel BL, Korf I, Robb SM, et al. MAKER: an easy-to-use annotation pipeline designed for emerging model organism genomes. *Genome Res*. 2008;18(1):188-96.

50. Lowe TM and Eddy SR. tRNAscan-SE: a program for improved detection of transfer RNA genes in genomic sequence. *Nucleic Acids Res*. 1997;25(5):955-64.

51. Nawrocki EP and Eddy SR. Infernal 1.1: 100-fold faster RNA homology searches. *Bioinformatics*. 2013;29(22):2933-5.

52. Griffiths-Jones S, Bateman A, Marshall M, et al. Rfam: an RNA family database. *Nucleic Acids Res*. 2003;31(1):439-41.

53. Boeckmann B, Bairoch A, Apweiler R, et al. The SWISS-PROT protein knowledgebase and its supplement TrEMBL in 2003. *Nucleic Acids Res*. 2003;31(1):365-70.

54. McGinnis S and Madden TL. BLAST: at the core of a powerful and diverse set of sequence analysis tools. *Nucleic Acids Res*. 2004;32(suppl_2):W20-W5.

55. Harris MA, Clark J, Ireland A, et al. The Gene Ontology (GO) database and informatics resource. *Nucleic Acids Res*. 2004; 32(suppl_1): D258-D61.

56. Ogata H, Goto S, Sato K, et al. KEGG: Kyoto Encyclopedia of Genes and Genomes. *Nucleic Acids Res*. 2000; 27:29-34.

57. Conesa A, Götz S, García-Gómez JM, et al. Blast2GO: a universal tool for annotation, visualization and analysis in functional genomics research. *Bioinformatics*. 2005;21(18):3674-6.

58. Li L, Stoeckert CJ and Roos DS. OrthoMCL: identification of ortholog groups for eukaryotic genomes. *Genome Res*. 2003;13(9):2178-89.

59. Edgar RC. MUSCLE: multiple sequence alignment with high accuracy and high throughput. *Nucleic Acids Res*. 2004;32(5):1792-7.

60. Suyama M, Torrents D and Bork P. PAL2NAL: robust conversion of protein sequence alignments into the corresponding codon alignments. *Nucleic Acids Res*. 2006;34:609-12.

61. Castresana J. Selection of Conserved Blocks from Multiple Alignments for Their Use in Phylogenetic Analysis. *Mol Biol Evol*. 2000;17(4):540-52.

62. Stamatakis A. RAxML version 8: a tool for phylogenetic analysis and post-analysis of large phylogenies. *Bioinformatics*. 2014;30(9):1312-3.

63. Stamatakis A, Hoover P and Rougemont J. A Rapid Bootstrap Algorithm for the RAxML Web Servers. *Syst Biol*. 2008;57(5):758-71.

64. Yang Z. PAML 4: Phylogenetic Analysis by Maximum Likelihood. *Mol Biol Evol*. 2007;24(8):1586-91.

65. Grabherr M, Haas BJ, Yassour M, et al. Full-length transcriptome assembly from RNA-Seq data without a reference genome. *Nat Biotechnol*. 2011;29(7):644-52.

66. De Bie T, Cristianini N, Demuth JP, et al. CAFE: a computational tool for the study of gene family evolution. *Bioinformatics*. 2006;22(10):1269-71.

67. Alexa A and Rahnenfuhrer J. topGO: enrichment analysis for gene ontology. https://bioconductor.org/packages/topGO. Accessed 12 September 2020.

68. Xie C, Mao X, Huang J, et al. KOBAS 2.0: a web server for annotation and identification of enriched pathways and diseases. *Nucleic Acids Res*. 2011;39(suppl_2):W316-W22.

69. Talavera G and Castresana J. Improvement of Phylogenies after Removing Divergent and Ambiguously Aligned Blocks from Protein Sequence Alignments. *Syst Biol*. 2007;56(4):564-77.

70. Mckenna A, Hanna M, Banks E, et al. The Genome Analysis Toolkit: A MapReduce framework for analyzing next-generation DNA sequencing data. *Genome Res*. 2010;20(9):1297-303.

71. Lee T, Guo H, Wang X, et al. SNPhylo: a pipeline to construct a phylogenetic tree from huge SNP data. *BMC Genomics*. 2014;15(1):162.

72. Purcell S, Neale BM, Toddbrown K, et al. PLINK: A Tool Set for Whole-Genome

Association and Population-Based Linkage Analyses. *Am J Hum Genet*. 2007;81(3):559-75.

73. Alexander DH, Novembre J and Lange K. Fast model-based estimation of ancestry in unrelated individuals. *Genome Res*. 2009;19(9):1655-64.

74. Tamura K, Dudley JT, Nei M, et al. MEGA4: Molecular Evolutionary Genetics Analysis (MEGA) Software Version 4.0. *Mol Biol Evol*. 2007;24(8):1596-9.

75. Liu S and Hansen MM. PSMC (pairwise sequentially Markovian coalescent) analysis of RAD (restriction site associated DNA) sequencing data. *Mol Ecol Resour*. 2017;17(4):631-41.

76. Yu M and He S. Phylogenetic relationships and estimation of divergence times among Sisoridae catfishes. *Sci China Life Sci*. 2012;55(4):312-20.

77. Huang DW, Sherman BT and Lempicki RA. Systematic and integrative analysis of large gene lists using DAVID bioinformatics resources. *Nat Protoc*. 2009;4(1):44-57.

78. Fang XM. Phased uplift of the Tibetan Plateau. *Sci Technol Rev*. 2017;6:42-50.

79. Ehlers J and Gibbard P. Quaternary Glaciation. In: Singh VP, Singh P and Haritashya UK, editors. Encyclopedia of Snow, Ice and Glaciers. Dordrecht: Springer Netherlands. 2011. p. 873-82.

80. Valdes PJ, Lin D, Farnsworth A, Spicer RA, Li S-H and Tao S. Comment on "Revised paleoaltimetry data show low Tibetan Plateau elevation during the Eocene". *Science*. 2019;365(6459):eaax8474.

81. Chang MM and Miao D. Review of the Cenozoic fossil fishes from the Tibetan Plateau and their bearings on paleoenvironment. *Chinese Sci Bull*. 2016;61(9):981-95.

82. Li J, Fang X, Song C, et al. Late Miocene–Quaternary rapid stepwise uplift of the NE Tibetan Plateau and its effects on climatic and environmental changes. *Quaternary Res*. 2014;81(3):400-23.

83. Murakami T, Terai H, Yoshiyama Y, et al. The second investigation of Lake Puma Yum Co located in the Southern Tibetan Plateau, China. *Limnology*. 2007;8(3):331-5.

84. Li S, Xia X, Zhou B, et al. Chemical balance of the Yellow River source region, the northeastern Qinghai-Tibetan Plateau: Insights about critical zone reactivity. *Appl Geochem*. 2018;90:1-12.

85. Li H, Zhang N and Lin X. Spatio-Temporal Characteristics of Yarlung Zangbo River in Tibet. *J Henan Norm Univ*. 2010;38(002):126-130.

86. Zhang N, Li H, Wen Z, et al. Spatio-Temporal Characteristics of Niyang River in Tibet. *J Henan Norm Univ*. 2009; 037(006):79-82.

87. Chen Y, Chen Y and Liu H. Studies on the position of the Qinghai-Xizang Plateau region in zoogeographic divisions and its eastern demarcation line. *Acta Hydrobiol Sinica*. 1996;20(2):97-103.

88. Van Meer G, Voelker DR and Feigenson GW. Membrane lipids: where they are and how they behave. *Nat Rev Mol Cell Biol*. 2008;9(2):112-24.

89. Hanna VS and Hafez EAA. Synopsis of arachidonic acid metabolism: A review. *J Adv Res*. 2018;11:23-32.

90. Liu B, Zheng Y, Yin F, et al. Toll receptor-mediated Hippo signaling controls innate immunity in Drosophila. *Cell*. 2016;164(3):406-19.

91. Hong L, Li X, Zhou D, et al. Role of Hippo signaling in regulating immunity. *Cell Mol Immunol*. 2018;15(12):1003-9.

92. Tong C, Tian F and Zhao K. Genomic signature of highland adaptation in fish: a case study in Tibetan Schizothoracinae species. *BMC genomics*. 2017;18(1):1-9.

93. Tong C, Fei T, Zhang C, et al. Comprehensive transcriptomic analysis of Tibetan Schizothoracinae fish *Gymnocypris przewalskii* reveals how it adapts to a high altitude aquatic life. *BMC Evol Biol*. 2017;17(1):1-11.

94. Macfadyen EJ, Williamson CE, Grad G, et al. Molecular response to climate change: temperature dependence of UV- induced DNA damage and repair in the freshwater crustacean *Daphnia pulicaria*. *Global Change Biol*. 2004;10(4):408-16.

95. Ensminger M and Lobrich M. One end to rule them all: Non-homologous end-joining and homologous recombination at DNA double-strand breaks. *Brit J Radiol*. 2020; 93: 20191054.

96. Kim H and Dandrea AD. Regulation of DNA cross-link repair by the Fanconi anemia/BRCA pathway. *Gene Dev*. 2012;26(13):1393-408.

97. Yu L, Wang G, Ruan J, et al. Genomic analysis of snub-nosed monkeys (*Rhinopithecus*) identifies genes and processes related to high-altitude adaptation. *Nat Genet*. 2016;48(8):947-52.

98. Rousselle M, Mollion M, Nabholz B, et al. Overestimation of the adaptive substitution rate in fluctuating populations. *Biol Letters*. 2018;14(5):20180055.

99. Chongqing Water Resources Bureau: Daning River http://www.cqwater.gov.cn/swgg/hkgk/Pages/2017/08/20170807165431.aspx Accessed 7 August 2017.

100. Hibshman JD, Doan AE, Moore BT, et al. daf-16/FoxO promotes gluconeogenesis and trehalose synthesis during starvation to support survival. *eLife*. 2017;6:e30057.

101. Kaidi A, Williams AC and Paraskeva C. Interaction between β-catenin and HIF-1 promotes cellular adaptation to hypoxia. *Nat Cell Biol*. 2007;9(2):210-7.

102. Sun Y, Zhou Y, Msuthwana P, et al. The role of CTNNB1 and LEF1 in feather follicles development of Anser cygnoides and Anser anser. *Genes Genom*. 2020;42(7):761-71.

103. Dong K, Pu Y, Wang Y, et al. Selective signatures reveal candidate genes for altitude adaptation and body size in Chinese native pig breeds. http://cast.cau.edu.cn/2010amab/2010AMABPage. Accessed 1 November 2014.

Figure ~~titles and~~ legends

Figure 1. Morphology and geographic distribution of *T. bleekeri*. (a) *T. bleekeri* used in this study. (b) Geographic distribution of the sampling locations for *T. bleekeri*.

~~**Figure 1. A picture of *T. bleekeri* used for genome sequencing and assembly.**~~

~~Figure 2. The geographic distribution of the sampling locations for *T. bleekeri*.~~

The red circles~~point~~, green triangle, yellow trapezoid, and dotted ellipse represent the sampling sites, gorge, artificial dam, and Wuxi Town, respectively.

~~**Figure 3. *K*mer frequency distribution from NGS short-read sequencing data.** The peak around the depth of 100X depicts the main Kmer species frequency used for the genome size estimation.~~

Figure 2. Phylogenetic relationships and divergence time estimation for *T. bleekeri* and other fish species. All nodes were completed and supported by 100 cycles of bootstrap resampling. Numbers near the nodes (shown in blue) indicate the estimated divergence times with a 95% confidence interval. Divergences used for the recalibration of time estimation are indicated with red dots.

Figure ~~4~~3. Natural positively selected gene~~s~~ (PSG~~s~~) identification and functional analysis for *T. bleekeri*, *T. tibetana*, and *T. siluroides*. (a) Venn diagram for PSGs for the three fish species. (b) Venn diagram for PSs identified from species- and lineage-based method. (c~~b~~) Enrichment analysis on the biological pathways for candidate ~~shared~~ PSGs identified from the species- and lineage-based method.~~for three species.~~

Figure 4. Historical effective population size profile deduced from the whole-genome sequencing data. One hundred bootstrap replicates were performed for the effective population size estimation.

~~**Figure 5. Phylogenetic relationships and divergence time estimation for *T. bleekeri* and other fish species.** All nodes were completed and supported by the 100 times bootstrap resampling. Blue numbers near the nodes indicate the estimated divergence times with a 95% confidence interval. Divergences used for the recalibration of time estimation are indicated with red dots.~~

Figure ~~6~~5. The population genetics analysis for *T. bleekeri*. (a) ~~neighbor~~Neighbor-joining phylogenetic tree of ~~*T. bleekeri*~~ individuals based on whole-genome SNP loci. Note that samples from population LHK, XX-, and BY~~one, two, and three~~ are labeled with red, green and blue, respectively. (b) Principal component (PC) analysis plots of the first two components. The fraction of the variance obtained was 14.5% for PC1 and 6.4% for PC2. The samples from population LHK, XX and BY are represented by red, green and blue color, respectively. (c) Population structure plots of *T. bleekeri*. The samples from population LHK, XX and BY are represented by red, green and blue color, respectively. We assume that there were three populations for the analysis (*K*=3). The y axis quantifies the proportion of the individual's genome from inferred ancestral populations, and x axis shows the different populations.

Figure 6. Selective sweep analysis to identify candidate selected functional genes among populations. (a) Manhattan plot to show the whole-wide genomic differentiation between LHK and BY populations. (b) The venn plot for shared enriched biological pathway for candidate selected functional genes from the selective sweep analysis among population comparisons. (c) The shared enriched biological pathway from LHK-BY and XX-BY comparisons. (d) The Fst profiles for genomic regions

containing *treh*, *ctnnb1* and *lef1* gene. Note that color scheme for population comparison is identical for (a), (b) and (c).

Manuscript (Unmarked) Click here to access/download;Manuscript;Manuscript (Unmarked) -Triplophysa bleekeri.docx

Chromosomal genome of *Triplophysa bleekeri* provides insights into its evolution and environmental adaptation

Dengyue Yuan[1], Xuehui Chen[1], Haoran Gu[1], Ming Zou[2], Yu Zou[2], Jian Fang[2], Wenjing Tao[1], Xiangyan Dai[1], Shijun Xiao[2,3,*], Zhijian Wang[1,*]

[1] Key Laboratory of Freshwater Fish Reproduction and Development (Ministry of Education), Key Laboratory of Aquatic Science of Chongqing, School of Life Sciences, Southwest University, Chongqing 400715, China

[2] School of Computer Science and Technology, Wuhan University of Technology, Wuhan, Hubei 430000, China

[3] College of Plant Protection, Jilin Agriculture University, Changchun, Jilin 130118, China

* Correspondence to Prof. Dr. Zhijian Wang (wangzj1969@126.com) and Dr. Shijun Xiao (shijun_xiao@163.com)

Abstract

Background: Intense stresses caused by high-altitude environments may result in noticeable genetic adaptions in native species. Studies of genetic adaptations to high elevations have been largely limited to terrestrial animals. How fish adapt to the high-elevation environments is largely unknown. *Triplophysa bleekeri*, an endemic fish inhabiting high-altitude regions, presents an excellent model to investigate the genetic mechanisms of adaptation to the local environment. Here, we assembled a chromosomal genome sequence of about 628 Mb with a contig and scaffold N50 of 3.1 and 22.9 Mb, respectively. We investigated the origin and environmental adaptation of *T. bleekeri* based on 21,198 protein-coding genes in the genome.

Results: Compared to fish species living at low altitudes, gene families associated with lipid metabolism and immune response were significantly expanded in the *T. bleekeri* genome. Genes involved in DNA repair exhibit positive selection for *T. bleekeri*, *T. siluroides*, and *T. tibetana*, indicating that adaptive convergence in *Triplophysa* species occurred at the positively selected genes. We also analyzed whole-genome variants among samples from three populations. The results showed that populations separated by geological and artificial barriers exhibited obvious differences in genetic structures, indicating that gene flow is restricted between populations.

Conclusions: These results will help us expand our understanding of environmental adaptation and genetic diversity of *T. bleekeri*, and provide valuable genetic resources for future studies on the evolution and conservation of high-altitude fish species such as *T. bleekeri*.

Keywords: *Triplophysa bleekeri*, genome, genetic adaptation, population genomics

Introduction

The Qinghai-Tibetan Plateau (QTP), the largest and highest plateau in the world, is one of the most important world biodiversity centers [1]. The environments of QTP and its peripheral areas were affected significantly by the continuing uplifts, which is one of the most important driving forces for the biological evolution of organisms on the plateau [2]. The endemic species of the QTP present high adaptability to the harsh environmental conditions, such as low temperature, low oxygen supply, and high UV radiation by exhibiting cold tolerance, hypoxia resistance, enhanced metabolic capacity, and increased body mass[3-6].

An investigation into the biological evolution of organisms residing on the QTP and its peripheral regions will broaden our understanding of essential evolutionary questions regarding mechanisms of environmental adaptation and speciation. Phenotype comparisons were frequently used to study environmental adaptations in previous studies [7, 8]. In recent years, advancing genomic technology, especially third-generation sequencing techniques, has presented novel opportunities to explore the genetic basis of environmental adaptations. Many genomic studies of terrestrial animals on the QTP and its peripheral regions revealed that genes involved in hypoxia response, energy metabolism, and DNA repair were under positive selection and rapid evolution [9-11]. In those studies, high-quality genome and population resources are essential to understand critical biological processes for adaptations [11-13].

The QTP boasts of having many highland fish species, especially in the family Sisoridae, subfamily Schizothoracinae, and genus *Triplophysa* [14]. To date, there have only been several high-quality highland fish genomes reported based on long-read sequencing data, including *Glyptosternon maculatum* in the family Sisoridae, *Schizothorax o'connori* and *Oxygymnocypris stewartii* in the subfamily Schizothoracinae, and *Triplophysa tibetana* and *Triplophysa siluroides* in the genus *Triplophysa* [15-19]. *Triplophysa* is a highly diverse genus and the largest group of the subfamily Nemacheilinae [20]. There are 152 records for *Triplophysa* species in FishBase, and the majority are distributed on the QTP and its adjacent drainage areas from an elevation of 100 m to over 5,200 m [21]. Given the broad elevation distributions and species diversity, the *Triplophysa* genus offers an attractive study model not only to investigate the adaptive mechanisms of fish in high altitudes, but also to examine the similarities and differences between the adaptive mechanisms in different *Triplophysa* species. Previous studies have reported the genomic data of *T. siluroides* and *T. tibetana* without any emphasis on the genetic basis of high-altitude adaption [17, 18]. To date, environmental adaptations of *Triplophysa* species to high altitudes are not fully understood, and the genetic resources for the reference genome and population data remain insufficient. *Triplophysa bleekeri*, another member of the Nemacheilidae family, is mainly distributed in the stem streams and tributaries of the Yangtze and Jinsha rivers [22]. It exhibits different ecological and physiological characteristics compared with its relatives, *T. siluroides* and *T. tibetana* [23]. *T. bleekeri* has a wide distribution, from 200 m to 3,000 m [24], whereas *T. tibetana* and *T. siluroides* occur at elevations of 4,000 ~

5,000 m, and 3,000 ~ 4,000 m, respectively [17, 25]. Apart from altitude of habitation, there is a significant difference in habitat environments. *T. bleekeri* lives in the fast-flowing rivers, whereas *T. tibetana* and *T. siluroides* inhabit lakes and slow flowing rivers [26]. Reproduction biology in these *Triplophysa* species is also different; *T. tibetana* and *T. siluroides* spawn once a year (from June to July, and July to August, respectively), whereas *T. bleekeri* can spawn twice a year, with peak breeding seasons occurring from October to December and March to April [24]. The primary food source of *T. bleekeri* and *T. tibetana* are *Chironomus* larvae, caddis fly larvae, and diatoms, whereas *T. siluroides* feeds smaller fishes [25]. The genome resource for *T. bleekeri* will contribute to understanding its evolution and environmental adaption and explore the convergent genetic mechanisms of *Triplophysa* species in high-elevation adaption.

In this study, we generated the first chromosomal genome sequence of *T. bleekeri* using the combined technology of the Illumina, PacBio, and Hi-C. Evolutionary and comparative genomic approaches were applied to clarify the origin of *T. bleekeri*, and to investigate the potential signals of adaption. Further, the population genetics of *T. bleekeri* were also investigated to reveal the genetic divergence among different populations.

Materials and Methods

Samples and tissue collection

Triplophysa bleekeri individuals (**Fig. 1a**) were obtained from the Daning River (31°09′26.58″N, 109°53′31.68″E), a tributary in the upper reaches of the Yangtze River, using brail nets (**Fig. 1b**). The fish were then transferred to the Aquaculture Laboratory

of Southwest University and reared in indoor tanks. To collect enough tissues for the genome and transcriptome sequencing, the largest female individual was used for library construction and sequencing. The fish was anesthetized with tricaine MS-222, and was immediately dissected to collect 12 types of tissues viz., brain, eye, skin, gill, heart, liver, trunk kidney, spleen, gut, muscle, gallbladder, and gonad. Tissues were quickly frozen in liquid nitrogen for more than one hour, and then stored at −80℃. Among these tissues, muscle tissue was used for genomic DNA sequencing and Hi-C library construction. Meanwhile, all tissue samples were used in the application of transcriptome sequencing to comprehensively characterize transcriptome. To understand the population structures of *T. bleekeri*, a total of 28 individuals were collected from three different reaches of Daning River, i.e., eleven, eleven, and six individuals from Lianghekou (LHK), Xixi (XX), and Baiyang (BY), respectively (**Fig. 1b**). These individuals were anesthetized with tricaine MS-222, and muscle tissue of each fish was collected as aforementioned.

Genome DNA extraction and sequencing library construction

DNA was extracted from muscle tissue using the phenol-chloroform DNA extraction method [27]. The Qubit (Thermo Fisher Scientific, Waltham, MA, USA) and Agilent Bioanalyzer 2100 (Agilent Technologies, Palo Alto, CA, USA) were used for evaluating the quantity and quality of DNA. For sequencing based on the Illumina HiSeq technology, a short-read sequencing library with an insert size of 250 bp was constructed using 1 μg of DNA. For sequencing on the PacBio SEQUEL platform (Pacific Biosciences, Menlo Park, CA, USA), the muscle DNA was used to construct

the long-read sequencing library. Briefly, 10 μg of *T. bleekeri* genomic DNA was used for 20-kb library preparation following the manufacturer's protocol (Pacific Biosciences), and the BluePippin Size Selection system (Sage Science, Beverly, MA, USA) was used for library size selection. DNA molecules from the largest individual were sequenced using the PacBio and Illumina platforms for genome assembly, and other samples were subjected to short-read whole-genome resequencing on the Illumina platform.

RNA extraction and sequencing library construction

RNA sequencing data provide important evidences for gene prediction in the genome [28]. To include as many expressed genes as possible, 12 tissue types, mentioned above, were used for the RNA sequencing library construction. RNA was isolated from the 12 tissue samples using TRIzol reagent (Invitrogen, USA). The quantity and quality of extracted RNA were determined using the Nanodrop ND-1000 spectrophotometer (LabTech, Holliston, MA, USA) and 2100 Bioanalyzer (Agilent Technologies, Palo Alto, CA, USA). Samples with a total RNA concentration ≥ 10 μg, and RNA integrity number ≥ 8 were used for sequencing. RNA molecules extracted from tissues were mixed in equal proportions for the following RNA library construction. RNA sequence library was constructed following the protocol of Paired-End Sample Preparation Kit (Illumina Inc., San Diego, CA, USA), which was identical to that employed in our previous study [29].

DNA and RNA library sequencing

The short-read DNA and RNA sequencing libraries were sequenced with the 150 bp paired-end (150PE) mode using the Illumina HiSeq X Ten platform (Illumina Inc.). The 20 kb long-read genome DNA SMRTbell libraries sequencing library was sequenced with the PacBio SEQUEL platform (Pacific Biosciences). The raw sequencing data were quality checked before the bioinformatics analysis. The high-throughput quality control (HTQC v0.90.8) package [30] was used to filter low-quality bases and reads, and sequences with adapters or low quality (average quality score < 20) were removed.

Genome size estimation

The genome size was estimated based on Illumina sequencing data using the *K*mer method before genome assembly. Raw Illumina reads were processed to remove adapter sequences, reads with more than 10% N bases, and reads with more than 50% low-quality bases (≤5). All filtered reads were used for *K*mer frequency analysis [31]. Using *K*mer size of 17, the *K*mer frequencies were obtained using Jellyfish v2.0 software [32]. *K*mers with a frequency of lower than 3 were eliminated as those likely resulted from sequencing errors. The genomic size was estimated based on the following formula: $G = (L - K + 1) \times n_{base} / (C_{Kmer} \times L)$, in which G is the estimated genome size, n_{base} is the total count of bases, C_{Kmer} is the expectation of *K*mer depth, L indicates the read length, and K represents *K*mer size. The revised genome size was calculated as follows: Revised Genome size = Genome size × (1-Error Rate).

***De novo* assembly of the *T. bleekeri* genome**

Long reads generated from the PacBio sequencing platform were used for *T.*

bleekeri genome assembly with the Falcon v0.3.0 package [33]. The assembled genome sequences were further polished with Arrow using long-read sequencing [34]; thereafter, two rounds of polishing using next-generation sequencing (NGS) short reads were performed with Pilon v1.23 [35]. Finally, redundant genomic sequences were eliminated using Redundans v0.14a with the parameter overlap of 0.95 and an identity of 0.95 [36]. Completeness of the assembled genome was evaluated using BUSCO v3.0 [37]. The database of actinopterygii_odb9 was used for the BUSCO analysis.

Chromosome assembly using Hi-C technology

Muscle tissue (1 g) of *T. bleekeri* was collected for PacBio sequencing and was used for Hi-C library construction. The Hi-C processes, including crosslinking, lysis, chromatin digestion, biotin marking, proximity ligations, crosslinking reversal, and DNA purification, were performed using the protocol described in previous studies [38]. The purified and enriched DNA was used for sequencing library construction. The library was sequenced using the Illumina HiSeq X Ten platform (Illumina), and the short-reads were then mapped to the polished genome of *T. bleekeri* with Bowtie v1.2.2. The chromosomal assembly using interaction frequency matrix extracted from the Hi-C read mapping was performed according to a previously-reported methodology [38].

Repetitive element annotation

The *de novo* prediction and homology prediction were combined to annotate the repetitive sequences in the *T. bleekeri* genome. RepeatModeler v2.0.1 [39] was used for the detection of *de novo* repetitive elements in the *T. bleekeri* genome. The detected genome repeats were combined with RepBase library [40], as a comprehensive library

for the final repetitive elements prediction in the *T. bleekeri* genome, using the RepeatMasker v4.1.1 software [41]. Transposons were predicted using ProteinMask, and the tandem repeats were identified in the genome using Tandem Repeat Finder v4.10 [42].

Protein coding and non-coding gene prediction

The *ab initio* prediction, homology prediction, and RNA-sequencing-based methods were used for protein-coding gene annotation. Gene models for protein-coding genes were first predicted in the *T. bleekeri* genome using Augustus v2.5.5 [43]. Five closely related fish species, viz., common carp (*Cyprinus carpio*), zebrafish (*Danio rerio*), Japanese medaka (*Oryzias latipes*), green spotted puffe (*Tetraodon nigroviridis*), and threes pined sticklebacks (*Xiphophorus maculatus*), were used for the homology-based protein-coding gene prediction. Protein sequences from those species, available in public databases, were mapped to the genome using TBLASTN [44] and GeneWise [45]. Thereafter, comprehensive transcriptome sequencing data for multiple-tissues were aligned to the genome, and gene models were generated using the TopHat v2.1.1 package [46] and Cufflinks v2.2.1 [47]. The integration and redundancy elimination for the gene models predicted using the above methods were performed using the MAKER package [48, 49]. We only selected genes with start and stop codons, and genes with internal stop codons were removed. Only genes with completed sequences and 70% overlaps among different gene model prediction methods will be retained as high-quality gene models.

Four types of non-coding RNAs, including microRNAs (miRNA), transfer RNAs (tRNA), ribosomal RNAs (rRNA), and small nuclear RNAs (snRNA), were also predicted in the *T. bleekeri* genome using tRNAscan-SE v1.3.1 [50] and using Infernal v1.1.3 [51] with the Rfam database [52].

Functional annotation of protein-coding genes

The NCBI non-redundant protein, Swissport, and TrEMBL databases [53] were used as protein databases for the biological function annotation using BLAST v2.10.1 packages [54]. The E-value of 1e-5 was used as the threshold for homolog identification. Gene Ontology (GO) [55] and Kyoto Encyclopedia of Genes and Genomes (KEGG) [56] assignments were performed using Blast2GO software [57].

Gene family clustering and phylogenetic analysis

Coding sequences annotated from whole-genome sequences for the closely related species were extracted from genome sequences. Gene family clustering was performed for *T. bleekeri* with eight fish species living in non-QTP regions, viz., zebrafish, Japanese medaka, elephant shark (*Callorhinchus milii*), spotted gar (*Lepisosteus oculatus*), Atlantic cod (*Gadus morhua*), platyfish (*X. maculatus*), tiger puffer (*Takifugu rubripes*), and large yellow croaker (*Larimichthys corcea*) by the Orthomcl v1.2 pipeline [58] with default settings. The single-copy orthologs across all species were selected for gene family, phylogenetic, and evolutionary analyses. Briefly, proteins of these genes were aligned with MUSCLE v3.8.31 [59] and were then transformed into alignments of nucleotide sequences with pal2nal [60] on the basis of the corresponding coding sequences. Next, non-conservative regions were removed

using Gblocks [61] with default settings, and the conservative regions were concatenated and fed in RaxML v8.2.10 [62] to deduce the phylogenetic relationships of these species using a GTRGAMMA model. Rapid bootstrap runs (100 times) were performed to test the robustness of the topology [63]. Based on the topology and the alignment matrix, their divergence times were deduced using MCMCTREE included in the PAML v1.3.1 package [64] with calibration points set by consulting the TimeTree database. *Danio rerio* and *Larimichthys crocea* (255-205 Ma), *Oryzias latipes* and *Larimichthys crocea* (115-105 Ma), *Lepisosteus oculatus* and *Danio rerio* (338-291 Ma) and *Callorhinchus milii* and *Danio rerio* (497-450 Ma) as calibration points for the divergence time estimation for other species.

To investigate the evolutionary relationship of species in *Triplophysa* genus, we also added another four *Triplophysa* genus fish species to the phylogenetic analysis. Since the genome of *T. xichangensis* and *T. scleroptera* have not been reported, we downloaded the short-reads of the transcriptomes of those two species from the NCBI sequence read archive (SRA) and conducted *de novo* assembly using Trinity v2.11.0 [65] with default settings. Longest transcripts for each gene were used in the following phylogenetic analysis. The single-copy orthologs across all species were used for phylogenetic tree reconstruction and divergence time estimation following the same method as described above.

Gene family expansion and contraction in the *T. bleekeri* genome

To identify expanded and contracted gene families in the *T. bleekeri* genome, we compared gene families in the *T. bleekeri* genome to those fish species living in non-

QTP regions viz., including elephant shark, spotted gar, zebrafish, Japanese medaka, platyfish, tiger puffer, large yellow croaker, Atlantic cod, green spotted puffe, and threes pined sticklebacks. CAFE v4.2.1 [66] was used to analyze the expansion and contraction of gene clusters in the *T. bleekeri* genome using a probabilistic model. A GO enrichment analysis was performed on expanded and contracted genes using the topGO v2.40.0 package [67]. The enrichment of genes in KEGG pathways was also analyzed using the KOBAS v1.2.0 [68].

Positively selected genes (PSGs) in genomes of *Triplophysa* species

MUSCLE v3.8.31 was used for multi-protein sequence alignment among the *T. bleekeri* genes and their orthologs, and compared to eight fish species living in non-QTP regions used in the gene family clustering analysis. Conserved coding sequence (CDS) alignments of each single-copy gene family were extracted using Gblocks [69] and used for further identification of PSGs. The ratios of nonsynonymous to synonymous substitutions (K_A/K_S, or ω) were estimated for each single-copy orthologous gene using the CodeML program with the branch-site model as implemented in the PAML package. A likelihood ratio test was conducted, and the false discovery rate (FDR) correction was performed for multiple comparisons. Genes with a corrected *P*-value < 0.05 were defined as PSGs. The genes putatively influenced by positive natural selection of *T. tibetana* and *T. siluroides* were also identified using the identical method. The functional annotation of PSGs for *T. bleekeri*, *T. tibetana*, and *T. siluroides* was also conducted using the same approach with the gene family expansion and contraction analysis.

Whole-genome re-sequencing and population genetics

Raw reads of samples subjected to resequencing were quality controlled as mentioned previously. The filtered short reads were mapped using BWA mem v0.7.17-r1188 with default settings for each individual, followed by the marking of duplicates with Picard. Regions near INDELs were thought to be poorly aligned and were identified and realigned using GATK v4.1.8.1 [70]. GATK was also used to call SNPs and INDELs based on the alignments. The SNPs and INDELs were then filtered by these parameters: QUAL (phred quality) > 30, QD (quality score divided by depth to comprehensively evaluate the quality and depth) > 2, DP (read depth)> 5, FS (phred-scaled p-value using Fisher's Exact Test to detect strand bias for reads) < 60, MQ (mapping quality to evaluate read alignment) >40, SOR (strand odds ratio to evaluate strand bias for reads) < 4.0. The identified SNPs were filtered using SNPhylo v20180901 [71] with default settings, except for LD_threshold and Minimum_depth_of_coverage, which were set to 0.8 and 5, respectively. Next, the principal components analysis (PCA) clusters and population structure for these individuals were deduced with Plink v1.9 [72] and Admixture [73, 74] with default settings, respectively. Their phylogenetic relationships were recovered using the neighbor-joining (NJ) method with MEGA4 [74], and bootstrap resampling (100 times) was performed to test the robustness of the tree topology.

Historical effective population size inference for *T. bleekeri*

Historical effective population size of *T. bleekeri* was estimated based using Pairwise Sequentially Markovian Coalescent (PSMC) v0.6.5 software [75].We used the

data for whole-genome variants of individuals for the genome assembly. The consensus sequences were generated using vcfutils.pl (vcf2fq -d 10 -D 300). The fq2psmcfa tool was used to create the input file for PSMC modelling. Sequences were used as the input for the PSMC estimates using ‘psmc’ with the options -N25 -t15 -r5. The reconstructed population history was plotted using ‘psmc_plot.pl’ with the generation time of 2 year and rate of 4×10^{-9} substitutions per synonymous site per year. The mutation rate was estimated from the gene comparison of *T. bleekeri* and *D. rerio*. Bootstrapping was conducted by randomly sampling with replacement 5- Mb sequence segments and 100 bootstrap replicates were performed.

Selection sweep analysis for populations

To identify genome-wide selective sweeps among populations, we calculated the genome-wide distribution of fixation index (F_{ST}) values and θπ ratios using SNPs from different populations. The F_{ST} values were Z-transformed as follows: $Z(F_{ST}) = (F_{ST} - \mu F_{ST}) / \sigma F_{ST}$, in which μF_{ST} was the mean F_{ST}, and σF_{ST} was the standard deviation of F_{ST}. The θπ ratios were log2-transformed. Subsequently, we scanned the genome in a 1 kb sliding scale, and estimated and ranked the empirical percentiles of $Z(F_{ST})$ and $\log_2$ (θπ ratio) in each window. We considered the windows with the top 1% $Z(F_{ST})$ and $\log_2$ (θπ ratio) as candidate outliers under strong selective sweeps. Genes residing in the outlier regions were considered as the candidate functional genes. The GO and KEGG enrichment were carried out by cluster Profiler v3.14.3 [76] and DAVID v6.8 [77].

Results

DNA and RNA library sequencing

81.69 Gb genomic (~120X) and 10.6 Gb transcriptomic short-reads were generated for the following genome size estimation and annotation (**Table 1**). We also obtained 100.87 Gb genomic long-reads from the PacBio platform, with a rough coverage of 160X for the *T. bleekeri* genome (**Table 1**). The mean and N50 length of the long-reads were 5.8 kb and 16 kb, respectively (**Table 1** and **Supplementary Fig. S1**).

Genome size estimation

To determine the possible sample contamination, 10,000 NGS short-reads were randomly selected for an NCBI nt database search. *Cyprinus*, *Danio*, and *Sinocyclocheilus* represent the top three sources of best hits, ruling out the obvious contamination during library construction and sequencing. Using genomic short-reads generated from the Illumina platform, 59.8 million *K*mers were generated. The genome of *T. bleekeri* was estimated as 632.5 Mb, with a heterozygosity ratio of 0.26% and repeat content of 42.2% (**Supplementary Fig. S2**). Based on the above genome character estimation, the genome of *T. bleekeri* was mid-sized with low heterozygosity.

***De novo* assembly of the *T. bleekeri* genome**

Using genomic PacBio long-reads for *T. bleekeri*, we assembled a 628 Mb genome with 856 contigs and an N50 length of 3.82 Mb (**Table 2**). Among these contigs, the longest contig for the genome was 15.5 Mb. The completeness of the assembled genome was evaluated using BUSCO v3.0 [37] with the actinopterygii_odb9 database, indicating that 92.9% of BUSCO genes were identified in the assembled genome (**Supplementary Fig. S3**).

Chromosome assembly using Hi-C technology

Hi-C technology recruits interaction information among different chromosome regions and assumes that the interactions for nearby regions are more prevalent than distant regions. In this study, 82.9 Gb sequencing data were obtained via Hi-C library sequencing. Based on the interacting information, a chromosome assembly of 628 Mb with a scaffold N50 length of 22.9 Mb was obtained (**Supplementary Fig. S4**). More than 596.9 Mb sequences were anchored upon 25 chromosomes, highlighting a high chromosome anchoring rate of 96.2% on the base level.

Repetitive element annotation

The annotation pipeline showed that more than 17.9 Mb of the genome sequences were predicted as tandem repeats, covering about 2.8% of the genome, and finally 203.2 Mb, accounting for roughly 32.4% of the genome, were annotated as repetitive elements in the *T. bleekeri* genome (**Supplementary Table S1**). Specifically, there are 17.2% DNA transposons (107.8 Mb), 5.8% of long interspersed nuclear elements (LINE) (36.4 Mb), 0.68% short interspersed nuclear elements (SINE) (4.3 Mb), and 6.93% long terminal repeats (LTR) (43.5 Mb).

Protein- and non-coding gene prediction, and functional annotation

For predicting protein-coding genes in the *de novo* assembled genome, 10.6 Gb short-read transcriptome data from 12 tissues was generated. Based on the *de novo*, homolog, and RNA-seq data methods, a total of 20,274, 27,243, and 15,875 protein-coding genes were predicted, respectively. After integration and redundancy elimination, 21,198 protein-coding genes were predicted in the *T. bleekeri* genome (**Supplementary Table S2**).

Of the 21,198 protein-coding genes, roughly 93.0%, 96.9%, and 90.9% displayed homologous sequences in the NCBI NR, TrEMBL, and Swissprot databases, respectively. Additionally, 89.2% contained InterPro domains, and 46.9% were assigned with GO terms. Overall, more than 97.3% of the protein-coding genes were functionally annotated by at least one method (**Supplementary Fig. S5**). The non-coding genes have received increased attention in the recent year, since accumulating evidence suggests that many of them play crucial roles in a variety of biological process. In this study, all the possible non-coding DNA sequences were predicted based on the *de novo* prediction strategies, and are summarized in **Supplementary Table S3**.

Gene family clustering and phylogenetic analysis of *T. bleekeri*

Using the whole-genome and transcriptome data of four other *Triplophysa* species, viz., *T. tibetana*, *T. siluroides*, *T. scleroptera,* and *T. xichangensis,* and the eight other fish species living in non-QTP regions, we performed the gene family clustering for those species. As a result, we identified 1, 364 single-copy orthologs among those fish species.

We then investigated the evolutionary relationship of *T. bleekeri* with respect to other *Triplophysa* and the non-QTP species. Using single-copy genes among species, a concatenated alignment matrix, was generated with a total length of 73,887 bps, which was used for the phylogenetic analysis and divergence time estimation. The result showed that *Triplophysa* species are phylogenetically closer to *D. rerio*, and that *T. siluroides* is a basal species within the *Triplophysa* group. Divergence time estimation showed that *T. bleekeri* diverged from their common ancestor, *T. scleroptera* and *T.*

xichangensis, around 25.2 million years ago (Ma) (**Fig. 2**).

Genes under natural positive selection

We identified 788 PSGs in the *T. bleekeri* genome. The functional analysis on the KEGG and GO parameters showed that several categories associated with nucleotide metabolism and DNA repair were significantly enriched (**Supplementary Table S4 and S5**). Additionally, the PSGs were also enriched in immune response, such as MyD88-dependent toll-like receptor signaling pathway (**Supplementary Table S4)**. Concomitantly, 969 and 1,253 PSGs were identified for *T. tibetana* and *T. siluroides*, respectively. Among those genes, 197 genes were identified as shared PSGs for the three *Triplophysa* species (**Fig. 3a**).

To detect candidate PSGs for *Triplophysa* ancestral lineage, we also performed the PSG identification for the common ancestor of the *Triplophysa* with the branch-site model in the PAML. As a result, we identified 439 PSGs for *Triplophysa* ancestral lineage. Interestingly, we found that only 35 shared PSGs for the three *Triplophysa* species were identical with *Triplophysa* lineage PSGs (**Fig. 3b**). The functional analysis with respect to biological pathways for the three *Triplophysa* species showed that those genes were significantly enriched for various processes including protein digestion and absorption, Fanconi anemia pathway, and salivary secretion (**Fig. 3c**). Twenty-five biological pathways, including peroxisome, autophagy, non-homologous end-joining, homologous recombination, basal transcription, ribosome biogenesis and spliceosome, were enriched for PSGs of *Triplophysa* ancestral lineage (**Fig. 3c**). The homologous recombination and basal transcription factor pathways were both enriched for

Triplophysa lineage PSGs and the three *Triplophysa* species shared PSGs (**Fig. 3c**).

Gene family expansion and contraction in the *T. bleekeri* genome

Following the Orthomcl pipeline, 21,862 ortholog groups were obtained after gene family clustering with ten fish species from non-QTP regions. Gene family analysis showed that 1,533 and 2,401 gene families were significantly expanded and contracted in *T. bleekeri*, respectively (**Supplementary Fig. S6**). The functional enrichment of expanded gene families was analyzed using GO and KEGG. The expanded gene families were primarily enriched in categories of metabolism and immune regulation (**Supplementary Tables S6** and **S7**). The categories of metabolism include fatty acid metabolism (arachidonic acid metabolism and glycosphingolipid biosynthesis), carbohydrate metabolism (glycosaminoglycan biosynthesis and glycan degradation), and amino acid metabolism (RNA transport). The categories of immune regulation include the Hippo signaling pathway (corrected *p*-value = 2.40E-03), necroptosis, and Vitamin B6 metabolism (corrected *p*-value = 8.90E-03). The contracted gene families were mainly made up of several signaling pathways, including the MAPK signaling pathway, calcium signaling pathway, adrenergic signaling in cardiomyocytes, GnRH signaling pathway, and retrograde endocannabinoid signaling (**Supplementary Tables S8** and **S9**).

Historical effective population size for *T. bleekeri* during the QTP formation

We used the whole-genome short-read sequencing data of the sample for genome assembly to obtain the genome-wide heterozygotic genotypes. Then, those variants were used to probe the profiles of historical effective population size for *T. bleekeri*

during the QTP formation. We used the gene comparison between *T. bleekeri* and *D. rerio* to estimate the mutation rate. As a result, we estimated the mutation rate of 4×10^{-9} for *T. bleekeri*. PSMC analysis performed using the above data showed that the effective population size of *T. bleekeri* increased more than 0.7 Ma, and reached a peak of 70×10^{4} around 0.6–0.7 Ma. However, the *T. bleekeri* population size experienced a dramatic drop afterwards to 1×10^{4} from 0.6 Ma to 60, 000 years ago (**Fig. 4**). The effective population size decline was consistent with the accelerating QTP uplifts around 1 Ma [78] and the quaternary glaciation spaning the Pleistocene (2.6–0.11 Ma) and Holocene (0.11–0 Ma) [19, 79]. We speculate that both the geotectonic movements and temperature fluctuations during the period exerted intense survival pressure for the ancient *T. bleekeri* populations, leading to the roughly 70 times effective population size drop during the period.

Population genetics analysis of *T. bleekeri*

The high-quality SNPs were obtained according to the filtering criteria set previously, and were used to deduce the population structures of *T. bleekeri*. As a result, more than 34 million short-reads were obtained for 28 individuals, and more than 3 million SNPs were detected for all individuals. The phylogeny reconstruction analyses based on whole-genome SNPs showed that individuals from population LHK and XX clustered together forming two neighboring groups, whereas individuals from population BY formed another cluster (**Fig. 5a**). The PCA clusters (**Fig. 5b**) also suggested that the first two principal components could successfully separate the individuals in population BY from those in population LHK and XX. In addition, genetic structure analysis also

indicated that gene flow between population BY and the other two populations was might be limited (**Fig. 5c**).

To investigate the possible natural selection among populations, we also performed the selective sweep analysis for BY, LHK and XX populations (**Fig. 6a**). Based on Fst comparison among those populations (**Supplementary Table S10**), we identified genomic regions (~1 kb in length) that scored in the top 1% (**Supplementary Fig. S7**). As a result, 1, 734, 3, 009, and 3, 244 regions (1 kb), harboring 474, 878, and 957 functional candidate genes were identified to be significantly genetically differentiated for LHK-XX, LHK-BY, and XX- BY comparisons, respectively. Genomic regions with less differentiation identified in LHK-XX comparison were consistent with the above phylogenetic analysis. The GO and KEGG pathway functional analyses showed 20, 25, and 31 significant biological pathway enrichments for LHK-XX, XX-BY, and LHK-BY comparisons, respectively **(Fig. 6b**, **Supplementary Table S11, S12, and S13)**. Six enriched biological pathways were shared in LHK-BY and XX-BY comparisons but not in LHK-XX one, viz., ubiquitin mediated proteolysis, tight junction, starch and sucrose metabolism, melanogenesis, longevity regulating pathway – mammal, and circadian rhythm (**Fig. 6c, Supplementary Table S12 and S13**). Five enriched biological pathways were shared for all comparisons, viz., axon guidance, long-term potentiation, Rap1 signaling pathway, circadian entrainment, and calcium signaling pathway (**Fig. 6b**).

Discussion

In this study, we presented the chromosome-level genome assembly of *T. bleekeri*

with a contig N50 of 3.1 Mb and a scaffold N50 of 22.9 Mb. The N50 lengths of contigs of *T. bleekeri* genome assembly were much longer than previously reported genome assemblies of *T. tibetana* [17]. Twenty-five chromosomes were obtained with the mounting rate up to 96.2%, and the assembled chromosome number was consistent with the karyotype of *T. bleekeri* (unpublished data), which suggests that the present analysis resulted in successful assembly of *T. bleekeri* genome to the chromosome level. The completeness of the genome was also evaluated, confirming the high quality of the assembled *T. bleekeri* genome. The combined results of the homology-based and *de novo* predictions showed that repetitive sequences accounted for 32.4% of the genome. Among them, DNA transposons represented the most abundant tandem repeats, which was also observed in *T. tibetana* [17]. Within the genome, 21,198 protein-coding genes were predicted, of which 97.3% could be functionally annotated. Overall, this genome assembly and annotation provides valuable data to the genomic resources currently available for the study of phylogeny and environmental adaptations of *Triplophysa* species.

The phylogenetic analysis results indicated that the *Triplophysa* genus formed a clade with *D. rerio*, and that *T. bleekeri* was most closely related to *T. tibetana* and *T. scleroptera*. The divergence time estimation indicated that *T. siluroides* diverged from their common ancestor roughly 38.8 Ma, occupying a basal position in the *Triplophysa* genus. The extensive QTP was elevated by more than 4,000 m about 40 Ma [80], and this time is consistent with the divergence of *T. siluroides*. Therefore, we speculated that the speciation of *Triplophysa* was likely triggered by the uplifting of the QTP [81].

Upliftment of the QTP profoundly induced on climatic and environmental changes of the plateau and its peripheral regions, including low oxygen and low temperature [82]. The oxygen content of air is inadequate in the QTP, while investigations into water quality indicated that high dissolved oxygen concentration exists in the QTP water [83-86]. Therefore, we speculated that thermal stress may present a major factor in natural selection for fish species in the QTP and its peripheral regions. Although *Triplophysa* species are widely distributed in different regions, these regions are all generally characterized by a cold environment [23, 87]. However, to the best of our knowledge, only a few studies have been conducted to explore the genetic basis of adaptation of *Triplophysa* species to low temperatures. Through the comparative analysis of the genome with other fish species, we found that the expanded gene families of *T. bleekeri* were significantly ($p < 0.05$) enriched in fatty acid metabolism, including glycosphingolipid biosynthesis and arachidonic acid metabolism pathways. The glycosphingolipid located in the bilayer lipid membrane is a major structural component of cell membranes [88], whereas arachidonic acid, an integral constituent of biological cell membranes, aids in the maintenance of cell membrane fluidity even at low temperatures [89]. Our results suggest that the increased number of genes related to fatty acid metabolism might be responsible for maintaining membrane structure and improving membrane fluidity under cold environments.

In the genome of *T. bleekeri*, significant expansion was also observed in Hippo signaling pathway gene family which participates in regulating innate immunity [90, 91]. These results suggest that *T. bleekeri* may tend to increase gene numbers in certain

families related to immune response for improving the defense against pathogens. It is notable that genes involved in innate immunity, such as toll-like receptor signaling pathway genes, all underwent positive selection in *T. bleekeri*, *T. tibetana*, and *T. siluroides*. Similar results were also observed in previous transcriptomic studies of Tibetan Schizothoracinae species, *Gymnocypris przewalskii*, and *G. przewalskii ganzihonensis* [92, 93]. These results indicated that the adaptive evolution of innate immunity might play crucial roles in the highland adaption of fish.

Low temperatures and UV radiation can cause DNA damage [94], and DNA damage response and repair pathways may show functional adaption. Within the three *Triplophysa* species, the PSGs were enriched in the functional categories of nucleotide excision repair, non-homologous end-joining, homologous recombination, and Fanconi anemia pathways (**Supplementary Table S4 and S5**). These pathways all participate in DNA repair, of which non-homologous end-joining and homologous recombination are the two main pathways for repairing double-strand break [95], and Fanconi anemia pathway is essential for the repair of DNA interstrand crosslinks [96]. PSGs influencing DNA repair may contribute to DNA integrity and genomic stability under high-altitude environments with low temperatures and intense UV radiation. Our results suggest that *Triplophysa* species have evolved an integrated DNA-repair mechanism to adapt to high-altitude environments. The previous studies also showed that genes involved in the DNA repair were under positive selection pressure in many species living at high altitudes, such as the snub-nosed monkey [97] and the Tibetan hot-spring snake [11]. It indicated that DNA damage caused by the environment is a common stress that animals

in high-altitude regions need to cope with. We also identified 197 PSGs shared by the three *Triplophysa* species (**Fig. 3A**), indicating that those naturally selected genes might have originated from their common ancestor, and that *Triplophysa* species were genetically convergent on PSGs. We found many species-specific PSGs for three *Triplophysa* species. The result implies the requirement of a distinct ecological niche for *T. bleekeri, T. tibetana,* and *T. siluroides*. Based on the generally used genomic comparison methods, hundreds of PSGs for *Triplophysa* species were identified in this investigation. However, a previous study has shown that ancient demographic fluctuation could generate severe overestimation of selective signatures [98]. Therefore, PSG identification in this work might have been influenced by the demographic scenarios of *Triplophysa* species. It is worth estimating the demographic fluctuation to PSG identification, and comparing the current methods for potential biases.

In addition to comparative genomics analyses, the relationships among populations of *T. bleekeri* were analyzed to probe possible differences in genetic structures. Population structure analysis divided 28 *T. bleekeri* samples into two clusters, with individuals from the LHK and XX population grouped together, and individuals from BY population forming the other cluster. Both PCA and structure analyses corroborated these findings. The BY population was separated from the LHK and XX population, and the observed admixture of genetic lineages was limited (K=3). These results could be because LHK and XX are directly connected by the river, and gene flow between individuals residing in the two places occurs more frequently. The difference between BY population and the LHK and XX populations might be attributed to the relatively

limited gene flow caused by natural and artificial barriers among those populations. The Daning River measures a height of up to 1,648 m, which flows through many narrower canyons[99]. Therefore, the geographical barriers formed by canyons and shallows could contribute to the diminished interaction among those populations. More importantly, artificial barriers, such as cities and dams, could also weaken the migrations between the BY and LHK/XX populations. Therefore, the whole-genome resequencing data of *T. bleekeri* provided a valuable genetic resource to reveal that geographical and artificial barriers could distinctly influence genetic exchange among populations.

The selective sweep analysis showed that genomic differentiation of LHK-XX was nonintensive compared to that of the BY population, which is consistent with the above population phylogenetic analysis. Notably, we identified six shared enriched biological pathways for LHK-BY and XX-BY comparisons but not in LHK-XX. Among those pathways, melanogenesis, circadian rhythm, and starch and sucrose metabolism might be closely related to the living environment differences caused by the presence of gorges, as well as human activity. The natural gorge might change the water flow and biodiversity of environments, and human activity could as well influence the nutrition supplies and circadian rhythm for local fish populations directly. We found that genomic regions in chromosome 11 containing alpha-trehalose glucohydrolase (*treh*) gene exhibited significant genetic differentiation in LHK-BY and XX-BY comparison but not in LHK-XX, imply *treh* gene might be related to the living environment for BY (**Fig. 6d**). Previous studies showed that trehalose plays an important role as a

protectant to preserve membrane organization and protein structure during abiotic stress, such as starvation and cold, and as a glycolytic input to maintain energy homeostasis [100]. Further, β-catenin (*ctnnb1*) and lymphoid enhancer-binding factor 1 (*lef1*) gene were also candidate functional genes related to local environment of XX because of gorges, artificial dam and human activity, since the genomic regions of those two genes for individuals in BY were significantly differentiated from those in LHK and XX (**Fig. 6d**). *Ctnnb1* has been reported as an important gene in colorectal tumorigenesis [101] and the interaction of *ctnnb1* and *lef1* could influence the down-stream gene regulation of cell profiles and developments [102]. The multiple-functions of *ctnnb1* present it as promising gene in maintain physiology and adaptation formation. Interestingly, a previous study has showed that *ctnnb1* might contribute to the high altitude environments environment adaptation for Tibetan pig [103].

In conclusion, we present a chromosomal-scale genome assembly of *T. bleekeri*, a representative high-altitude fish. Evolutionary, comparative, and population genomic analyses were performed to investigate the evolution, environmental adaption, and genetic diversity of *T. bleekeri*. Our results provide insights into how fish adapt to the high-altitude environment, and the genomic data serves as a valuable resource for further study on functional validation of candidate genes contributing to environmental adaption.

Ethics Statement

All experimental protocols were approved by the School of Life Sciences, Southwest University (Chongqing, China), and the studies were carried out in

accordance with the Guidelines of Experimental Animal Welfare from Ministry of Science and Technology of People's Republic of China (2006) and the Institutional Animal Care and Use Committee protocols from Southwest University (2007).

Acknowledgement

This work was supported by the Financial Program of Ministry of Agriculture and Rural Affairs of China (Grant No. YYJZHC201921301350063), National Natural Science Foundation of China (Grant No. 31602207), and Research Innovation Program for College Graduates of Chongqing (Grant No. CYB19079).

Author contributions

ZJ Wang conceived and designed the study; DY Yuan and SJ Xiao collected the samples; DY Yuan and SJ Xiao performed molecular experiments; SJ Xiao performed the bioinformatics analysis, including genome size estimation, genome assembly, annotation, and gene prediction; DY Yuan, SJ Xiao, and ZJ Wang wrote the manuscript. All authors read and approved the final manuscript for submission.

Competing interests

All authors declare that they have no competing interests.

Data accessibility

The genomic, transcriptome, and Hi-C sequencing reads generated from the PacBio and Illumina platforms are available in the NCBI SRA database under the Accession no. SRP200140. The final chromosome assembly was submitted to NCBI with the accession number of VFQW00000000.

Abbreviations

BUSCO: Benchmarking universal single-copy orthologs; CAFE: Computational analysis of gene family evolution; CDS: Conserved coding sequence; FDR: False discovery rate; GO: Gene ontology; Hi-C: High-throughput chromosome conformation capture; KEGG: Kyoto encyclopedia of genes and genomes; LINE: Long interspersed nuclear elements; LTR: Long terminal repeats; QTP: Qinghai-Tibetan Plateau; PAML: Phylogenetic analysis by maximum likelihood; PCA, Principal components analysis; PSG: Positively selected genes; PSMC: Pairwise Sequentially Markovian Coalescent; SINE: Short interspersed nuclear elements; SRA: Sequence read archive.

References

1. Myers N, Mittermeier RA, Mittermeier CG, et al. Biodiversity hotspots for conservation priorities. *Nature*. 2000;403(6772):853.
2. Zhao Z and Li S. Extinction vs. Rapid radiation: The juxtaposed evolutionary histories of coelotine spiders support the Eocene–Oligocene orogenesis of the Tibetan Plateau. *Syst Biol*. 2017;66(6):988-1006.
3. Beall CM. Adaptation to high altitude: phenotypes and genotypes. *Annu Rev Anthropol*. 2014;43:251-72.
4. Monge C and Leonvelarde F. Physiological adaptation to high altitude: oxygen transport in mammals and birds. *Physiol Rev*. 1991;71(4):1135-72.
5. Wu T and Kayser B. High Altitude Adaptation in Tibetans. *High Alt Med Biol*. 2006;7(3):193-208.
6. Beall CM. Two routes to functional adaptation: Tibetan and Andean high-altitude natives. *Proc Natl Acad Sci U.S.A*. 2007;104:8655-60.
7. Ding CZ, Jiang XM, Chen L, et al. Growth variation of *Schizothorax dulongensis* Huang, 1985 along altitudinal gradients: implications for the Tibetan Plateau fishes under climate change. *J Appl Ichthyol*. 2016;32(4):729-33.
8. Deng H, Yue X, Chen D, et al. Growth characteristics and feed habit of *Triplophysa stenura* in Nujiang River. *Freshw Fisheries*. 2010;40(1):26-33.
9. Li M, Tian S, Jin L, et al. Genomic analyses identify distinct patterns of selection in domesticated pigs and Tibetan wild boars. *Nat Genet*. 2013;45(12):1431.
10. Qiu Q, Zhang G, Ma T, et al. The yak genome and adaptation to life at high altitude. *Nat Genet*. 2012;44(8):946.

11. Li JT, Gao YD, Xie L, et al. Comparative genomic investigation of high-elevation adaptation in ectothermic snakes. *Proc Natl Acad Sci U.S.A*. 2018;115(33):8406-11.

12. Liu ZJ, Liu SK, Yao J, et al. The channel catfish genome sequence provides insights into the evolution of scale formation in teleosts. *Nat Commun*. 2016;7:11757.

13. Sun YB, Fu TT, Jin JQ, et al. Species groups distributed across elevational gradients reveal convergent and continuous genetic adaptation to high elevations. *Proc Natl Acad Sci U.S.A*. 2018;115(45):E10634-E41.

14. Wu YF and Tan QJ. Characteristics of the fish-fauna of the characteristics of Qinghai-Xizang plateau and its geological distribution and formation. *Acta Zool Sinica*. 1991;37:135-52.

15. Liu HP, Liu QY, Chen ZQ, et al. Draft genome of *Glyptosternon maculatum*, an endemic fish from Tibet Plateau. *GigaScience*. 2018;7(9):giy104.

16. Liu HP, Xiao SJ, Wu N, et al. The sequence and de novo assembly of *Oxygymnocypris stewartii* genome. *Sci Data*. 2019;6:190009.

17. Yang X, Liu H, Ma Z, et al. The chromosome-level genome assembly of *Triplophysa tibetana* , a fish adapted to the harsh high-altitude environment of the Tibetan plateau. *Mol Ecol Resour*. 2019;19(4):1027-36.

18. Yang L, Wang Y, Wang T, et al. A chromosome-scale reference assembly of a Tibetan loach, *Triplophysa siluroides*. *Front Genet*. 2019;10:991.

19. Xiao S, Mou Z, Fan D, et al. Genome of Tetraploid Fish Schizothorax o'connori Provides Insights into Early Re-diploidization and High-Altitude Adaptation. *iScience*. 2020;23(9):101497.

20. Nelson JS, Grande TC and Wilson MV. Fishes of the World. John Wiley & Sons; 2016.

21. He CL, Song ZB and Zhang E. *Triplophysa* fishes in China and the status of its taxonomic studies. *Sichuan J Zool*. 2011;30(1):150-5.

22. He XF, He JS and Yan TM. Reproductive characteristic of *Triplophysa bleekeri* in mabian river. *J Southwest China Norm Univ*. 1999;24(1):69-73.

23. Xiao H and Dai YG. A Review of Study on Diversity of *Triplophysa* in China. *Fisheries Sci*. 2011;30(1):53-7.

24. Wang ZJ, Huang J and Zhang YG. The reproductive traits of *Triplophysa bleekeri* in the Daning River. *Freshw Fisheries*. 2013;43(5):8-12.

25. Zhu S. The loaches of the subfamily Nemacheilinae in China (Cypriniformes: Cobitidae). Jiangsu Science and Technology Publishing House; 1989.

26. Wu YT, Tang QJ. Characteristics of the fish-fauna of the characteristics of Qinghai-Xizang Plateau and its geological distribution and formation. *Acta Zool Sinica*. 1991;2:135-152.

27. Xiao SJ, Wang PP, Dong LS, et al. Whole-genome single-nucleotide polymorphism (SNP) marker discovery and association analysis with the eicosapentaenoic acid (EPA) and docosahexaenoic acid (DHA) content in *Larimichthys crocea*. *PeerJ*. 2016;4:e2664.

28. Denoeud F, Aury J-M, Da Silva C, et al. Annotating genomes with massive-scale RNA sequencing. *Genome Bio*. 2008;9(12):R175.

29. Xiao SJ, Han ZF, Wang PP, et al. Functional marker detection and analysis on a

comprehensive transcriptome of large yellow croaker by next generation sequencing. *PloS One*. 2015;10(4):e0124432.

30. Yang X, Liu D, Liu F, et al. HTQC: a fast quality control toolkit for Illumina sequencing data. *BMC Bioinformatics*. 2013;14(1):33.

31. Liu B, Shi Y, Yuan J, et al. Estimation of genomic characteristics by analyzing k-mer frequency in de novo genome projects. *arXiv: Genomics*. 2013.

32. Marcais G and Kingsford C. A fast, lock-free approach for efficient parallel counting of occurrences of k-mers. *Bioinformatics*. 2011;27(6):764-70.

33. Chin CS, Peluso P, Sedlazeck FJ, et al. Phased diploid genome assembly with single-molecule real-time sequencing. *Nat Methods*. 2016;13(12):1050.

34. Chin CS, Alexander DH, Marks P, et al. Nonhybrid, finished microbial genome assemblies from long-read SMRT sequencing data. *Nat Methods*. 2013;10(6):563.

35. Walker BJ, Abeel T, Shea T, et al. Pilon: an integrated tool for comprehensive microbial variant detection and genome assembly improvement. *PloS One*. 2014;9(11):e112963.

36. Pryszcz LP and Gabaldón T. Redundans: an assembly pipeline for highly heterozygous genomes. *Nucleic acids Res*. 2016;44(12):e113.

37. Simão FA, Waterhouse RM, Ioannidis P, et al. BUSCO: assessing genome assembly and annotation completeness with single-copy orthologs. *Bioinformatics*. 2015;31(19):3210-2.

38. Gong GR, Dan C, Xiao SJ, et al. Chromosomal-level assembly of yellow catfish genome using third-generation DNA sequencing and Hi-C analysis. *GigaScience*. 2018;7(11):giy120.

39. Smit A, Hubley R and Green P. RepeatModeler Open-1.0. 2008–2015. http://www repeatmasker.org. Accessed 1 May, 2018.

40. Jurka J, Kapitonov VV, Pavlicek A, et al. Repbase Update, a database of eukaryotic repetitive elements. *Cytogenet Genome Res*. 2005;110(1-4):462-7.

41. Tarailo- Graovac M and Chen NS. Using RepeatMasker to identify repetitive elements in genomic sequences. *Curr Protoc Bioinform*. 2009;25(1):4.10.1- 4.10.14.

42. Benson G. Tandem repeats finder: a program to analyze DNA sequences. *Nucleic Acids Res*. 1999;27:573.

43. Stanke M, Keller O, Gunduz I, et al. AUGUSTUS: ab initio prediction of alternative transcripts. *Nucleic Acids Res*. 2006;34(suppl_2):W435-W9.

44. Lobo I. Basic local alignment search tool (BLAST). *Nat Educ*. 2008;1(1).

45. Birney E, Clamp M and Durbin RJ. GeneWise and Genomewise. *Genome Res*. 2004;14(5):988.

46. Trapnell C, Pachter L and Salzberg SL. TopHat: discovering splice junctions with RNA-Seq. *Bioinformatics*. 2009;25:1105-11.

47. Ghosh S and Chan CK. Analysis of RNA-Seq Data Using TopHat and Cufflinks. *Methods Mol Biol*. 2016;1374:339.

48. Campbell MS, Holt C, Moore B, et al. Genome Annotation and Curation Using MAKER and MAKER-P. *Curr Protoc Bioinform*; 2014;48(1): 4.11.1-4.11.39.

49. Cantarel BL, Korf I, Robb SM, et al. MAKER: an easy-to-use annotation pipeline designed for emerging model organism genomes. *Genome Res*. 2008;18(1):188-96.

50. Lowe TM and Eddy SR. tRNAscan-SE: a program for improved detection of transfer RNA genes in genomic sequence. *Nucleic Acids Res*. 1997;25(5):955-64.

51. Nawrocki EP and Eddy SR. Infernal 1.1: 100-fold faster RNA homology searches. *Bioinformatics*. 2013;29(22):2933-5.

52. Griffiths-Jones S, Bateman A, Marshall M, et al. Rfam: an RNA family database. *Nucleic Acids Res*. 2003;31(1):439-41.

53. Boeckmann B, Bairoch A, Apweiler R, et al. The SWISS-PROT protein knowledgebase and its supplement TrEMBL in 2003. *Nucleic Acids Res*. 2003;31(1):365-70.

54. McGinnis S and Madden TL. BLAST: at the core of a powerful and diverse set of sequence analysis tools. *Nucleic Acids Res*. 2004;32(suppl_2):W20-W5.

55. Harris MA, Clark J, Ireland A, et al. The Gene Ontology (GO) database and informatics resource. *Nucleic Acids Res*. 2004; 32(suppl_1): D258-D61.

56. Ogata H, Goto S, Sato K, et al. KEGG: Kyoto Encyclopedia of Genes and Genomes. *Nucleic Acids Res*. 2000; 27:29-34.

57. Conesa A, Götz S, García-Gómez JM, et al. Blast2GO: a universal tool for annotation, visualization and analysis in functional genomics research. *Bioinformatics*. 2005;21(18):3674-6.

58. Li L, Stoeckert CJ and Roos DS. OrthoMCL: identification of ortholog groups for eukaryotic genomes. *Genome Res*. 2003;13(9):2178-89.

59. Edgar RC. MUSCLE: multiple sequence alignment with high accuracy and high throughput. *Nucleic Acids Res*. 2004;32(5):1792-7.

60. Suyama M, Torrents D and Bork P. PAL2NAL: robust conversion of protein sequence alignments into the corresponding codon alignments. *Nucleic Acids Res*. 2006;34:609-12.

61. Castresana J. Selection of Conserved Blocks from Multiple Alignments for Their Use in Phylogenetic Analysis. *Mol Biol Evol*. 2000;17(4):540-52.

62. Stamatakis A. RAxML version 8: a tool for phylogenetic analysis and post-analysis of large phylogenies. *Bioinformatics*. 2014;30(9):1312-3.

63. Stamatakis A, Hoover P and Rougemont J. A Rapid Bootstrap Algorithm for the RAxML Web Servers. *Syst Biol*. 2008;57(5):758-71.

64. Yang Z. PAML 4: Phylogenetic Analysis by Maximum Likelihood. *Mol Biol Evol*. 2007;24(8):1586-91.

65. Grabherr M, Haas BJ, Yassour M, et al. Full-length transcriptome assembly from RNA-Seq data without a reference genome. *Nat Biotechnol*. 2011;29(7):644-52.

66. De Bie T, Cristianini N, Demuth JP, et al. CAFE: a computational tool for the study of gene family evolution. *Bioinformatics*. 2006;22(10):1269-71.

67. Alexa A and Rahnenfuhrer J. topGO: enrichment analysis for gene ontology. https://bioconductor.org/packages/topGO. Accessed 12 September 2020.

68. Xie C, Mao X, Huang J, et al. KOBAS 2.0: a web server for annotation and identification of enriched pathways and diseases. *Nucleic Acids Res*. 2011;39(suppl_2):W316-W22.

69. Talavera G and Castresana J. Improvement of Phylogenies after Removing Divergent and Ambiguously Aligned Blocks from Protein Sequence Alignments. *Syst Biol*. 2007;56(4):564-77.

70. Mckenna A, Hanna M, Banks E, et al. The Genome Analysis Toolkit: A MapReduce framework for analyzing next-generation DNA sequencing data. *Genome Res*. 2010;20(9):1297-303.

71. Lee T, Guo H, Wang X, et al. SNPhylo: a pipeline to construct a phylogenetic tree from huge SNP data. *BMC Genomics*. 2014;15(1):162.

72. Purcell S, Neale BM, Toddbrown K, et al. PLINK: A Tool Set for Whole-Genome Association and Population-Based Linkage Analyses. *Am J Hum Genet*. 2007;81(3):559-75.

73. Alexander DH, Novembre J and Lange K. Fast model-based estimation of ancestry in unrelated individuals. *Genome Res*. 2009;19(9):1655-64.

74. Tamura K, Dudley JT, Nei M, et al. MEGA4: Molecular Evolutionary Genetics Analysis (MEGA) Software Version 4.0. *Mol Biol Evol*. 2007;24(8):1596-9.

75. Liu S and Hansen MM. PSMC (pairwise sequentially Markovian coalescent) analysis of RAD (restriction site associated DNA) sequencing data. *Mol Ecol Resour*. 2017;17(4):631-41.

76. Yu M and He S. Phylogenetic relationships and estimation of divergence times among Sisoridae catfishes. *Sci China Life Sci*. 2012;55(4):312-20.

77. Huang DW, Sherman BT and Lempicki RA. Systematic and integrative analysis of large gene lists using DAVID bioinformatics resources. *Nat Protoc*. 2009;4(1):44-57.

78. Fang XM. Phased uplift of the Tibetan Plateau. *Sci Technol Rev*. 2017;6:42-50.

79. Ehlers J and Gibbard P. Quaternary Glaciation. In: Singh VP, Singh P and Haritashya UK, editors. Encyclopedia of Snow, Ice and Glaciers. Dordrecht: Springer Netherlands. 2011. p. 873-82.

80. Valdes PJ, Lin D, Farnsworth A, Spicer RA, Li S-H and Tao S. Comment on "Revised paleoaltimetry data show low Tibetan Plateau elevation during the Eocene". *Science*. 2019;365(6459):eaax8474.

81. Chang MM and Miao D. Review of the Cenozoic fossil fishes from the Tibetan Plateau and their bearings on paleoenvironment. *Chinese Sci Bull*. 2016;61(9):981-95.

82. Li J, Fang X, Song C, et al. Late Miocene–Quaternary rapid stepwise uplift of the NE Tibetan Plateau and its effects on climatic and environmental changes. *Quaternary Res*. 2014;81(3):400-23.

83. Murakami T, Terai H, Yoshiyama Y, et al. The second investigation of Lake Puma Yum Co located in the Southern Tibetan Plateau, China. *Limnology*. 2007;8(3):331-5.

84. Li S, Xia X, Zhou B, et al. Chemical balance of the Yellow River source region, the northeastern Qinghai-Tibetan Plateau: Insights about critical zone reactivity. *Appl Geochem*. 2018;90:1-12.

85. Li H, Zhang N and Lin X. Spatio-Temporal Characteristics of Yarlung Zangbo River in Tibet. *J Henan Norm Univ*. 2010;38(002):126-130.

86. Zhang N, Li H, Wen Z, et al. Spatio-Temporal Characteristics of Niyang River in Tibet. *J Henan Norm Univ*. 2009; 037(006):79-82.

87. Chen Y, Chen Y and Liu H. Studies on the position of the Qinghai-Xizang Plateau region in zoogeographic divisions and its eastern demarcation line. *Acta Hydrobiol Sinica*. 1996;20(2):97-103.

88. Van Meer G, Voelker DR and Feigenson GW. Membrane lipids: where they are and how they behave. *Nat Rev Mol Cell Biol*. 2008;9(2):112-24.

89. Hanna VS and Hafez EAA. Synopsis of arachidonic acid metabolism: A review. *J Adv Res*. 2018;11:23-32.

90. Liu B, Zheng Y, Yin F, et al. Toll receptor-mediated Hippo signaling controls innate immunity in Drosophila. *Cell*. 2016;164(3):406-19.

91. Hong L, Li X, Zhou D, et al. Role of Hippo signaling in regulating immunity. *Cell Mol Immunol*. 2018;15(12):1003-9.

92. Tong C, Tian F and Zhao K. Genomic signature of highland adaptation in fish: a case study in Tibetan Schizothoracinae species. *BMC genomics*. 2017;18(1):1-9.

93. Tong C, Fei T, Zhang C, et al. Comprehensive transcriptomic analysis of Tibetan Schizothoracinae fish *Gymnocypris przewalskii* reveals how it adapts to a high altitude aquatic life. *BMC Evol Biol*. 2017;17(1):1-11.

94. Macfadyen EJ, Williamson CE, Grad G, et al. Molecular response to climate change: temperature dependence of UV- induced DNA damage and repair in the freshwater crustacean *Daphnia pulicaria*. *Global Change Biol*. 2004;10(4):408-16.

95. Ensminger M and Lobrich M. One end to rule them all: Non-homologous end-joining and homologous recombination at DNA double-strand breaks. *Brit J Radiol*. 2020; 93: 20191054.

96. Kim H and Dandrea AD. Regulation of DNA cross-link repair by the Fanconi anemia/BRCA pathway. *Gene Dev*. 2012;26(13):1393-408.

97. Yu L, Wang G, Ruan J, et al. Genomic analysis of snub-nosed monkeys (*Rhinopithecus*) identifies genes and processes related to high-altitude adaptation. *Nat Genet*. 2016;48(8):947-52.

98. Rousselle M, Mollion M, Nabholz B, et al. Overestimation of the adaptive substitution rate in fluctuating populations. *Biol Letters*. 2018;14(5):20180055.

99. Chongqing Water Resources Bureau: Daning River http://www.cqwater.gov.cn/swgg/hkgk/Pages/2017/08/20170807165431.aspx Accessed 7 August 2017.

100. Hibshman JD, Doan AE, Moore BT, et al. daf-16/FoxO promotes gluconeogenesis and trehalose synthesis during starvation to support survival. *eLife*. 2017;6:e30057.

101. Kaidi A, Williams AC and Paraskeva C. Interaction between β-catenin and HIF-1 promotes cellular adaptation to hypoxia. *Nat Cell Biol*. 2007;9(2):210-7.

102. Sun Y, Zhou Y, Msuthwana P, et al. The role of CTNNB1 and LEF1 in feather follicles development of Anser cygnoides and Anser anser. *Genes Genom*. 2020;42(7):761-71.

103. Dong K, Pu Y, Wang Y, et al. Selective signatures reveal candidate genes for altitude adaptation and body size in Chinese native pig breeds. http://cast.cau.edu.cn/2010amab/2010AMABPage. Accessed 1 November 2014.

Figure legends

Figure 1. Morphology and geographic distribution of *T. bleekeri*. (a) *T. bleekeri* used in this study. (b) Geographic distribution of the sampling locations for *T. bleekeri*. The red circles, green triangle, yellow trapezoid, and dotted ellipse represent the sampling sites, gorge, artificial dam, and Wuxi Town, respectively.

Figure 2. Phylogenetic relationships and divergence time estimation for *T. bleekeri* and other fish species. All nodes were completed and supported by 100 cycles of bootstrap resampling. Numbers near the nodes (shown in blue) indicate the estimated divergence times with a 95% confidence interval. Divergences used for the recalibration of time estimation are indicated with red dots.

Figure 3. Natural positively selected gene (PSG) identification and functional analysis for *T. bleekeri*, *T. tibetana*, and *T. siluroides*. (a) Venn diagram for PSGs for the three fish species. (b) Venn diagram for PSs identified from species- and lineage-based method. (c) Enrichment analysis on the biological pathways for candidate PSGs identified from the species- and lineage-based method.

Figure 4. Historical effective population size profile deduced from the whole-genome sequencing data. One hundred bootstrap replicates were performed for the effective population size estimation.

Figure 5. The population genetics analysis for *T. bleekeri*. (a) Neighbor-joining phylogenetic tree of individuals based on whole-genome SNP loci. Note that samples from population LHK, XX, and BY are labeled with red, green and blue, respectively. (b) Principal component (PC) analysis plots of the first two components. The fraction

of the variance obtained was 14.5% for PC1 and 6.4% for PC2. The samples from population LHK, XX and BY are represented by red, green and blue color, respectively. (c) Population structure plots of *T. bleekeri*. The samples from population LHK, XX and BY are represented by red, green and blue color, respectively. We assume that there were three populations for the analysis (*K*=3). The y axis quantifies the proportion of the individual's genome from inferred ancestral populations, and x axis shows the different populations.

Figure 6. Selective sweep analysis to identify candidate selected functional genes among populations. (a) Manhattan plot to show the whole-wide genomic differentiation between LHK and BY populations. (b) The venn plot for shared enriched biological pathway for candidate selected functional genes from the selective sweep analysis among population comparisons. (c) The shared enriched biological pathway from LHK-BY and XX-BY comparisons. (d) The Fst profiles for genomic regions containing *treh*, *ctnnb1* and *lef1* gene. Note that color scheme for population comparison is identical for (a), (b) and (c).

Table 1 Click here to access/download;Table;Table 1.docx

Table 1. A summary of sequencing data used in genome assembly and gene annotation.

Source	Platform	Clean Data (Gb)	Mean Read Length (bp)	Sequence Coverage (X)
genome	Illumina HiSeq X Ten	81.7	150	129
genome	PacBio SEQUEL	100.87	5,827	160
genome (Hi-C)	Illumina HiSeq X Ten	83.5	150	132
transcriptome	Illumina HiSeq X Ten	11.1	150	-

 Click here to access/download;Table;Table 2.docx

Table 2. The length statistics for contig assembly for the *T. bleekeri* genome

	Assemble	Total Length (bp)	Sequence Number	Contig N50 (Mb)	Scaffold N50 (Mb)
contig assembly using long-read data	Falcon	657,392,105	1,357	3.31	3.31
	Arrow	660,275,268	1,357	3.33	3.33
	Pilon	659,964,583	1,357	3.33	3.33
	Redundans	628,132,429	856	3.82	3.82
chromosome assembly using Hi-C data	all sequences	620,272,795	181	3.11	22.89
	chromosomes	596,964,218	25	3.23	23.21
	unanchored sequences	23,308,577	156	0.17	1.01

Figure 1

Click here to access/download;Figure;Figure 1.docx

Figure 1. Morphology and geographic distribution of *T. bleekeri*

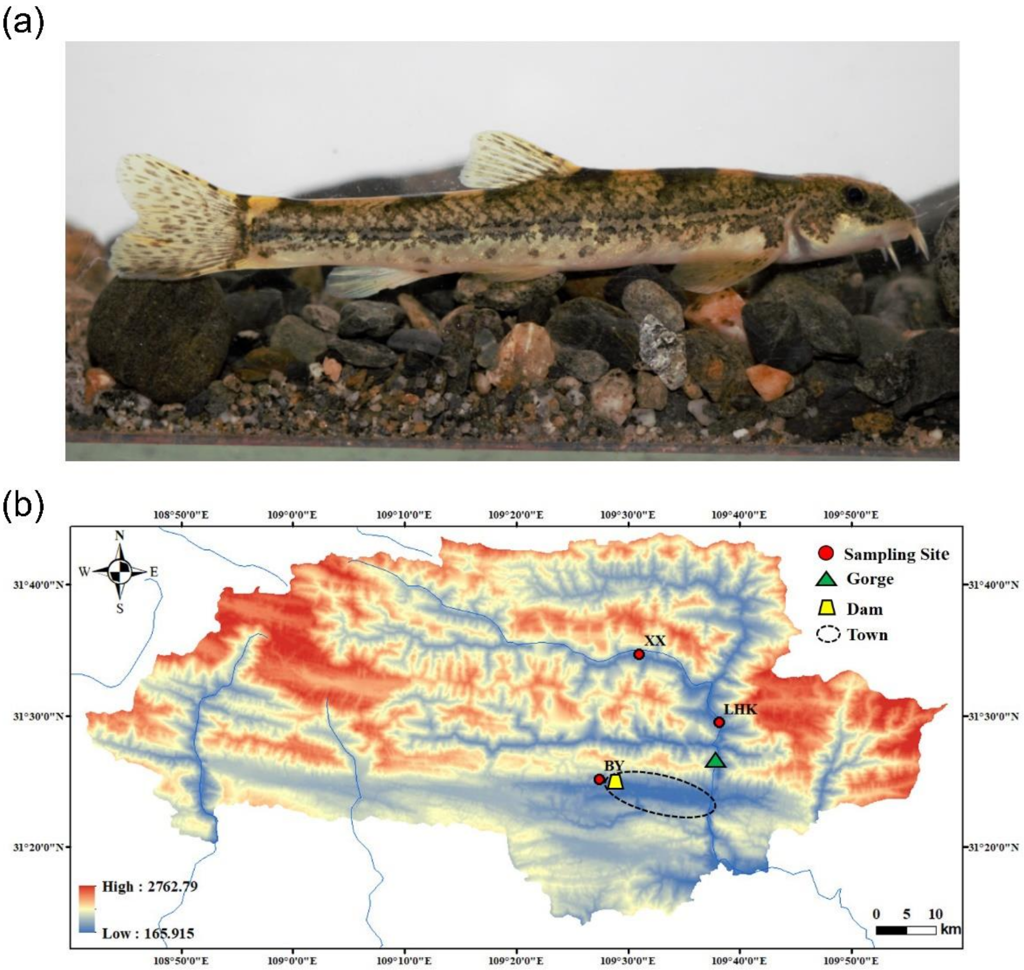

Figure 2 Click here to access/download;Figure;Figure 2.docx

Figure 2. Phylogenetic relationships and divergence time estimation for *T. bleekeri* and other fish species.

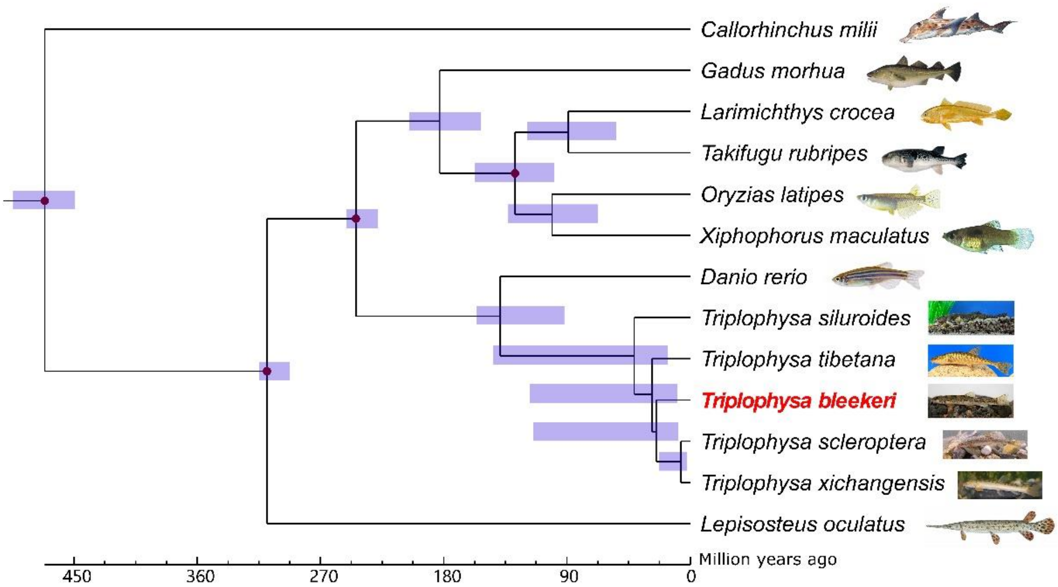

Figure 3 Click here to access/download;Figure;Figure 3.docx

Figure 3. Natural positively selected gene (PSG) identification and functional analysis for *T. bleekeri*, *T. tibetana*, and *T. siluroides*.

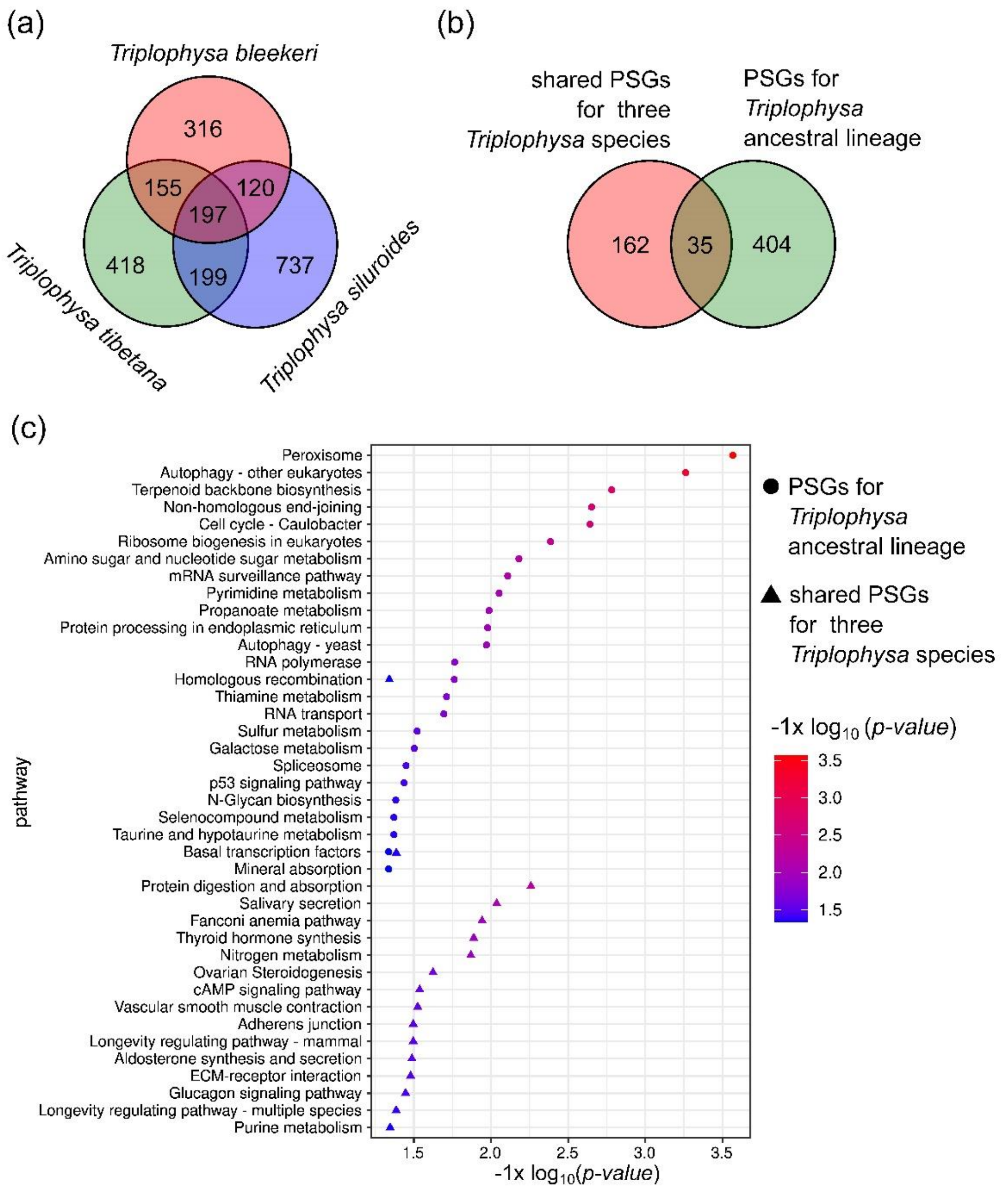

 Click here to access/download;Figure;Figure 4.docx

Figure 4. Historical effective population size profile deduced from the whole-genome sequencing data.

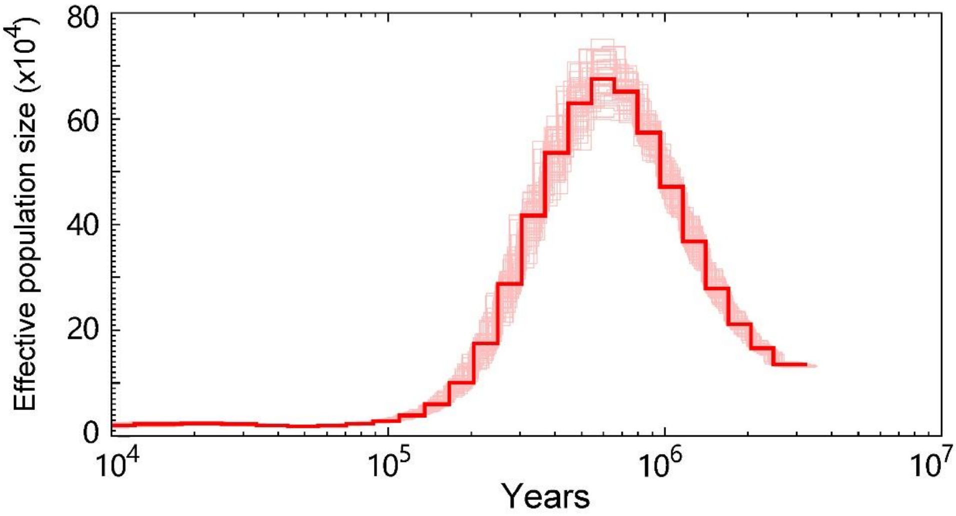

Figure 5 Click here to access/download;Figure;Figure 5.docx

Figure 5. The population genetics analysis for *T. bleekeri*.

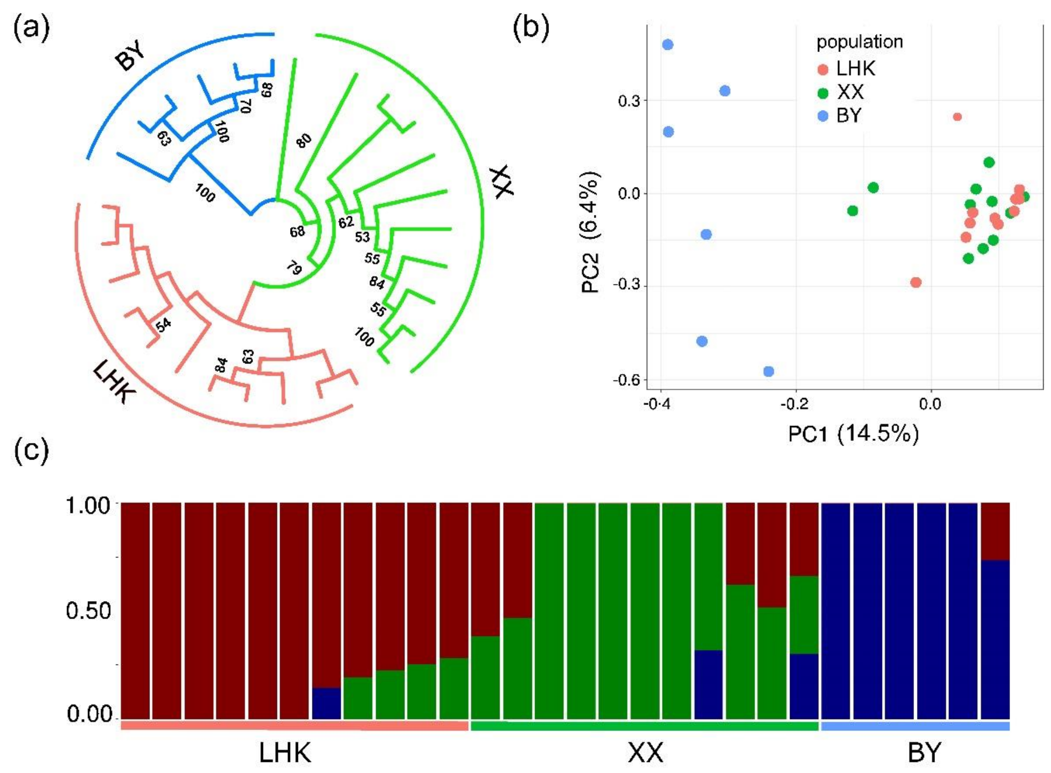

Click here to access/download;Figure;Figure 6.docx

Figure 6. Selective sweep analysis to identify candidate selected functional genes among populations.

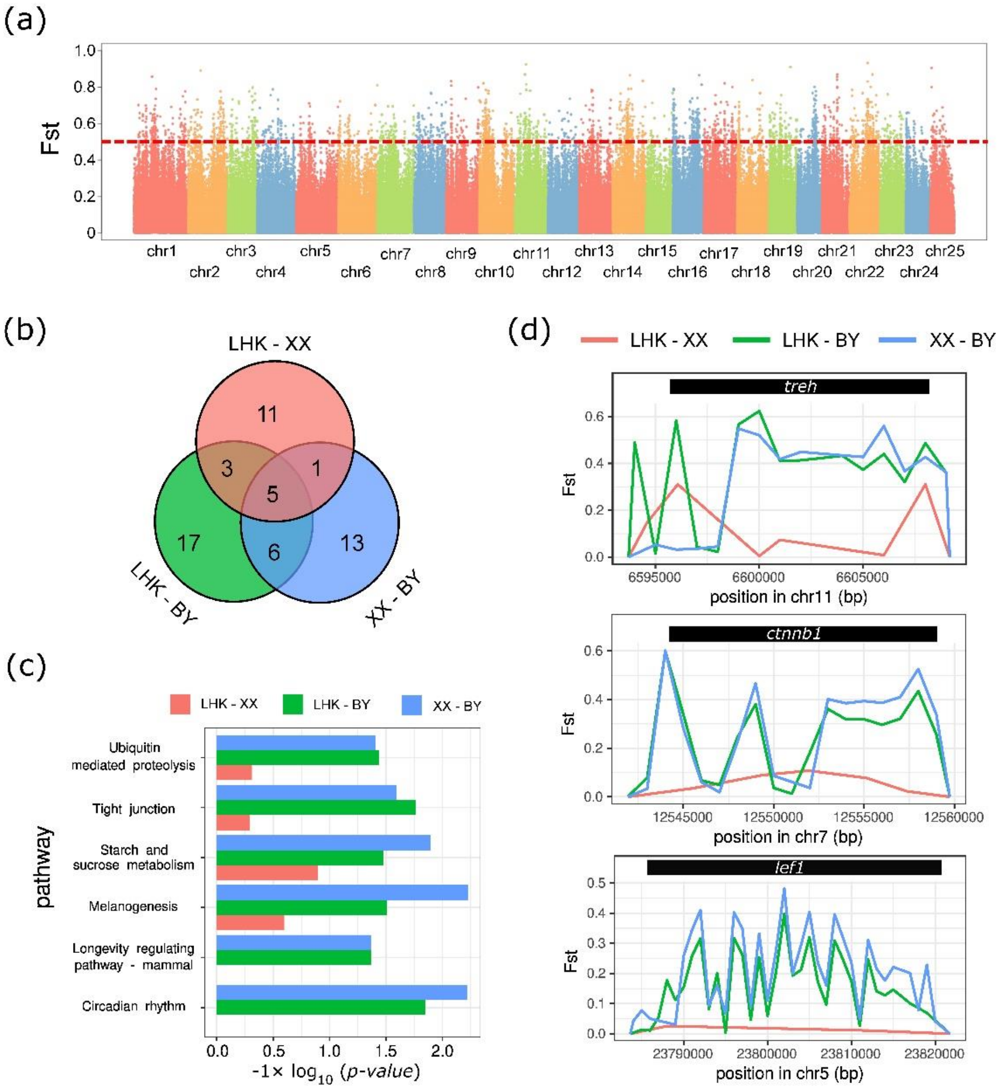

Supplementary Material 1

Click here to access/download
Supplementary Material
Additional file 1.docx

Supplementary Material 2

Click here to access/download

Supplementary Material

Additional file 2-Supplementary Table 4.xls

Click here to access/download
Supplementary Material
Additional file 3-Supplementary Table 5.xls

Click here to access/download
Supplementary Material
Additional file 4-Supplementary Table 11.xlsx

Click here to access/download
Supplementary Material
Additional file 5-Supplementary Table 12.xlsx

Click here to access/download
Supplementary Material
Additional file 6-Supplementary Table 13.xlsx

Personal Cover

Click here to access/download;Personal Cover;Cover letter-Triplophysa bleekeri.docx

Dear Editor,

Attached is our recent manuscript entitled "**Chromosomal genome assembly of *Triplophysa bleekeri* provides insights into its evolution and environmental adaptation on the Qinghai-Tibetan Plateau**." We would like to send for your consideration to publish as a resource article in ***GigaScience***.

The continuous uplifts and climatic changes during the formation of Qinghai-Tibetan Plateau (QTP) posed profound influences on the evolution of endemic organisms. Fish species on the QTP are more susceptible to tectonic movements and temperature fluctuations since it strictly constrained by water ecology and drainage connectivity in habitats. Living in extreme environments, including low temperature and high UV exposure, highland endemic fishes have been subjected to severe natural selections; however, few studies were performed to investigate the molecular mechanism of the adaptation formation for fish species on the QTP. The genome and population resources of endemic fish species play an essential role in adaptive evolution studies. Although several genomes of fish species on the QTP, including *Glyptosternon maculatum, Oxygymnocypris stewartii*, *Triplophysa tibetana*, and *Triplophysa siluroides*, have been reported, the genetic resource, especially for whole-genome population data, is still insufficient, largely hindered the evolution and conservation genetics studies of endemic fish.

Triplophysa bleekeri (*T. bleekeri*), a typical fish species occurring at an elevation from 200 m to 3,000 m, provides us an excellent model to investigate the adaption mechanism and population genetics for fish on the QTP. In this study, we assembled a chromosome genome for *T. bleekeri* using Illumina, PacBio sequencing platform, and Hi-C technique. Based on more than 160 X coverage of long sequencing data, we generated a 628 Mb *T. bleekeri* genome with a contig N50 length of 3.82 Mb. Using interaction frequencies among contigs from the Hi-C technique, a chromosome genome was assembled with a scaffold N50 length of 22.9 Mb, and more than 96.2% of the genome on the base level was successfully anchored upon 25 chromosomes. A

total of 21,198 protein-coding genes were predicted in the *T. bleekeri* genome, of which 97.3% of the protein-coding genes were functionally annotated.

We explored the environmental adaptation of *T. bleekeri* from the perspective of functional genes in the genome. We found that gene families related to lipid metabolism, necroptosis, and immune response were significantly expanded in the *T. bleekeri* genome, comparing to those non-highland fish species. Genes involved in DNA repair and protein digestion underwent strong natural positive selections for *T. bleekeri*, *T. siluroides* and *T. tibetana*. Our result implied that *T. bleekeri* might under severe stress with the cold environment, and *Triplophysa* species might be under similar natural selections. We also performed whole-genome resequencing for 28 samples from three populations in the Daning River. We illuminated the difference between their genetic structures, which can be explained by the relatively limited gene flow hampered by natural gorges and artificial barriers, such as cities and dams, among those populations.

Our work provided important reference genome and population variation resource and preliminary investigation of the environmental adaptation and population structures of *T. bleekeri*. Those data will not only be used for further ecological and conservation studies for the species but also offered valuable information for the evolutionary researches based on the comparative analysis among endemic fish and vertebrates.

All authors have read and approved this version of the article. No part of this paper has been published or submitted elsewhere. No conflict of interest exists in the submission of this manuscript.

We are looking forward to receiving a favorable response from you regarding the acceptance of the manuscript. Thank you for your help.

Sincerely Yours,

Prof. Zhijian Wang
School of Life Sciences, Southwest University

Beibei, Chongqing, PR China 400715
Tel: 86-023-68253005
Fax: 86-023-68253005
Email: wangzj1969@126.com
