# Supplementary material for: Chromosomal genome of Triplophysa bleekeri provides insights into its evolution and environmental adaptation
Source: Gigascience. 2020 Nov 24;9(11):giaa132. doi: 10.1093/gigascience/giaa132 (PMC7684707; doi:10.1093/gigascience/giaa132)
Supplement: giaa132_GIGA-D-20-00124_Revision_2 [file giaa132_giga-d-20-00124_revision_2.pdf]

GigaScience

Chromosomal genome of Triplophysa bleekeri provides insights into its evolution and environmental adaptation

--Manuscript Draft--

**Manuscript Number:**	GIGA-D-20-00124R2
**Full Title:**	Chromosomal genome of Triplophysa bleekeri provides insights into its evolution and environmental adaptation
**Article Type:**	Data Note
**Funding Information:**	Financial Program of Ministry of Agriculture and Rural Affairs of China (YYJZHC201921301350063) — Dr. Zhijian Wang<br>National Natural Science Foundation of China (31602207) — Dr. Shijun Xiao<br>Research Innovation Program for College Graduates of Chongqing (CYB19079) — Dr. Dengyue Yuan
**Abstract:**	Background: Intense stresses caused by high-altitude environments may result in noticeable genetic adaptions in native species. Studies of genetic adaptations to high elevations have been largely limited to terrestrial animals. How fish adapt to the high-elevation environments is largely unknown. Triplophysa bleekeri , an endemic fish inhabiting high-altitude regions, presents an excellent model to investigate the genetic mechanisms of adaptation to the local environment. Here, we assembled a chromosomal genome sequence of about 628 Mb with a contig and scaffold N50 of 3.1 and 22.9 Mb, respectively. We investigated the origin and environmental adaptation of T. bleekeri based on 21,198 protein-coding genes in the genome.<br>Results: Compared to fish species living at low altitudes, gene families associated with lipid metabolism and immune response were significantly expanded in the T. bleekeri genome. Genes involved in DNA repair exhibit positive selection for T. bleekeri , T. siluroides , and T. tibetana , indicating that adaptive convergence in Triplophysa species occurred at the positively selected genes. We also analyzed whole-genome variants among samples from three populations. The results showed that populations separated by geological and artificial barriers exhibited obvious differences in genetic structures, indicating that gene flow is restricted between populations.<br>Conclusions: These results will help us expand our understanding of environmental adaptation and genetic diversity of T. bleekeri , and provide valuable genetic resources for future studies on the evolution and conservation of high-altitude fish species such as T. bleekeri .
**Corresponding Author:**	Zhijian Wang<br>Southwest University<br>Chongqing, China CHINA
**Corresponding Author Secondary Information:**	
**Corresponding Author's Institution:**	Southwest University
**Corresponding Author's Secondary Institution:**	
**First Author:**	Dengyue Yuan
**First Author Secondary Information:**	
**Order of Authors:**	Dengyue Yuan<br>Xuehui Chen<br>Haoran Gu<br>Ming Zou<br>Yu Zou

Powered by Editorial Manager® and ProduXion Manager® from Aries Systems Corporation

	Jian Fang
	Wenjing Tao
	Xiangyan Dai
	Shijun Xiao
	Zhijian Wang
**Order of Authors Secondary Information:**	
**Response to Reviewers:**	Manuscript number: GIGA-D-20-00124<br>Article Type: Data Note<br>Title: Chromosomal genome of Triplophysa bleekeri provides insights into its evolution and environmental adaptation<br>Correspondence Author: Shijun Xiao; Zhijian Wang<br><br>Dear editor,<br>Thank you very much for your hard work in processing our manuscript. We have carefully read the suggestions from the reviewer and modified the manuscript based on these suggestions. We submit a revised manuscript here. The following are the correspondences to your reviewer concerning the comments and suggestions about the manuscript.<br>We wish to take this opportunity to thank your consideration of our paper published in your journal, GigaScience.<br><br>Best regards.<br>Sincerely Yours,<br>Dr. Zhijian Wang<br><br>Detailed responses to Reviewers<br>Reviewer #1<br>General Comment:<br>Compared to previous version, the authors have considerably improved both the content, presentation and readability of the manuscript. However, there are still issues that should be solved. For example, the discussion of the top 1% signals of the selection among 3 populations and specific candidate genes sounds extremely subjective and speculative. (see below). Hence I suggest to delete these parts in the discussion. In addition, there are still some issues with the language (see some suggestions for correction below, line numbers correspond to the version with changes visible).<br>Response: Dear reviewer, thank you for your kind comments for our manuscript. We appreciate your valuable comments and suggestions to improve our paper. We carefully revised the manuscript based on your kind suggestions. We hope that changes having been made to the manuscript meet to your satisfaction.<br><br>Detailed comments:<br>Comment 1: Line 266: Consider replacing „Only genes with completed sequences.." with „Only genes with complete sequences...".<br>Response: Thanks for your suggestion. We have revised this sentence (line 215, Marked MS).<br><br>Comment 2: Line 300: Consider replacing „...milii and Danio rerio (497-450 Ma) as calibration points for the divergence time..." with „...milii and Danio rerio (497-450 Ma) were used as calibration points for the divergen"ce time..."<br>Response: We revised this sentence based on your good suggestion (line 249, Marked MS).<br><br>Comment 3: Line 302: Consider replacing „To investigate the evolutionary relationship of species in Triplophysa genus.." with „To investigate the evolutionary relationships within genus Triplophysa...."<br>Response: Thanks for your kind comments. We revised this sentence in line 251 (Marked MS).<br><br>Comment 4: Line 446-447: Add references to following claim and replace „The non-coding genes have received increased attention in the recent year, since accumulating

	evidence suggests that many of them play crucial roles in a variety of biological process." With "The non-coding genes have received increased attention in the recent years, since accumulating evidence suggests that many of them play crucial roles in a variety of biological process.<br>Response: Thanks for your kind suggestions. We revised this sentence and added the reference (lines: 378~381, Marked MS).<br><br>Comment 5: Line 530: Do you mean: "We used the whole-genome short-read sequencing data based on the sample used for genome assembly to obtain the genome-wide genotype data."<br>Response: We are very sorry for our inappropriate description. We revised this sentence base on your comment (lines: 441~443, Marked MS).<br><br>Comment 6: Lines 534-537: I am not sure if I understood it correctly. If the authors used gene regions to estimate mutation rate between T. bleekeri and D. rerio and applied this estimate to analyze SNP data from the whole genome, it is certainly an underestimate for whole genome as gene regions are under functional constraint.<br>Response: Thanks for your good comment. It is a widely used model to estimate the mutation rate from the Ks estimation based on $Ks=2\mu T$, where Ks is the synonymous mutation rate, T is the divergence time between species, and μ is the mutation rate (Eyre-Walker, 2000; Koch et al., 2000; Zhang et al., 2013).<br><br>Comment 7: Lines 576: Consider replacing: "To investigate the possible natural selection among populations" with " To identify putative signals of differential selection among populations".<br>Response: We revised this sentence based on your excellent suggestion (line 468, Marked MS).<br><br>Comment 8: Lines 731-734: Pinpointing süecific selection agents as gorge or human activities is highly speculative given that only 3 populations were studied. I suggest to remove this part.<br>Response: We have removed this part according to the reviewer's suggestion (lines 592~595, Marked MS).<br><br>Comment 9: Lines 736-752: Give that the selection threshold for identifying potential footprints of selection was rather relaxed (the empirical top 1% of the distribution) and not based on any simulations to identify the likelihood of being under selection, I think the discussion on individual selection targets is way too speculative and selective (why the authors selected to discuss these few genes out of all other 1% genes?). Hence, I suggest to remove it.<br>Response: We have removed this part according to the reviewer's suggestion (lines 597~613, Marked MS).<br><br>Special thanks for your good comments and suggestions.<br><br><br><br>References<br>Eyre-Walker, A. (2000). Fundamentals of Molecular Evolution (2nd edn). Heredity 84, 735-735.<br>Koch, M.A., Haubold, B., and Mitchell-Olds, T. (2000). Comparative evolutionary analysis of chalcone synthase and alcohol dehydrogenase loci in Arabidopsis, Arabis, and related genera (Brassicaceae). Molecular biology and evolution 17, 1483-1498.<br>Zhang, L., Yan, H.-F., Wu, W., Yu, H., and Ge, X.-J. (2013). Comparative transcriptome analysis and marker development of two closely related Primrose species (Primula poissonii and Primula wilsonii). BMC genomics 14, 329.
**Additional Information:**	
**Question**	**Response**
Are you submitting this manuscript to a special series or article collection?	No

Powered by Editorial Manager® and ProduXion Manager® from Aries Systems Corporation

**Experimental design and statistics**<br><br>Full details of the experimental design and statistical methods used should be given in the Methods section, as detailed in our Minimum Standards Reporting Checklist. Information essential to interpreting the data presented should be made available in the figure legends.<br><br>Have you included all the information requested in your manuscript?	Yes
**Resources**<br><br>A description of all resources used, including antibodies, cell lines, animals and software tools, with enough information to allow them to be uniquely identified, should be included in the Methods section. Authors are strongly encouraged to cite Research Resource Identifiers (RRIDs) for antibodies, model organisms and tools, where possible.<br><br>Have you included the information requested as detailed in our Minimum Standards Reporting Checklist?	Yes
**Availability of data and materials**<br><br>All datasets and code on which the conclusions of the paper rely must be either included in your submission or deposited in publicly available repositories (where available and ethically appropriate), referencing such data using a unique identifier in the references and in the "Availability of Data and Materials" section of your manuscript.<br><br>Have you have met the above requirement as detailed in our Minimum Standards Reporting Checklist?	Yes

Powered by Editorial Manager® and ProduXion Manager® from Aries Systems Corporation

Manuscript

Click here to access/download;Manuscript;Manuscript (Unmarked)Tbleekeri_production.docx

Chromosomal genome of *Triplophysa bleekeri* provides insights into its evolution and environmental adaptation

Dengyue Yuan[1], Xuehui Chen[1], Haoran Gu[1], Ming Zou[2], Yu Zou[2], Jian Fang[2], Wenjing Tao[1], Xiangyan Dai[1], Shijun Xiao[2,3,*], Zhijian Wang[1, *] ORCID 0000-0001-9826-2399

[1] Key Laboratory of Freshwater Fish Reproduction and Development (Ministry of Education), Key Laboratory of Aquatic Science of Chongqing, School of Life Sciences, Southwest University, Chongqing 400715, China

[2] School of Computer Science and Technology, Wuhan University of Technology, Wuhan, Hubei 430000, China

[3] College of Plant Protection, Jilin Agriculture University, Changchun, Jilin 130118, China

* Correspondence to Prof. Dr. Zhijian Wang (wangzj1969@126.com) and Dr. Shijun Xiao (shijun_xiao@163.com)

Abstract

Background: Intense stresses caused by high-altitude environments may result in noticeable genetic adaptions in native species. Studies of genetic adaptations to high elevations have been largely limited to terrestrial animals. How fish adapt to high-elevation environments is largely unknown. *Triplophysa bleekeri*, an endemic fish inhabiting high-altitude regions, is an excellent model to investigate the genetic mechanisms of adaptation to the local environment. Here, we assembled a chromosomal genome sequence of *Triplophysa bleekeri*, with a size of about 628 Mb (contig and scaffold N50 of 3.1 and 22.9 Mb, respectively). We investigated the origin and environmental adaptation of *T. bleekeri* based on 21,198 protein-coding genes in the genome.

Results: Compared to fish species living at low altitudes, gene families associated with lipid metabolism and immune response were significantly expanded in the *T. bleekeri* genome. Genes involved in DNA repair exhibit positive selection for *T. bleekeri*, *T. siluroides*, and *T. tibetana*, indicating that adaptive convergence in *Triplophysa* species occurred at the positively selected genes. We also analyzed whole-genome variants among samples from three populations. The results showed that populations separated by geological and artificial barriers exhibited obvious differences in genetic structures, indicating that gene flow is restricted between populations.

Conclusions: These results will help us expand our understanding of environmental adaptation and genetic diversity of *T. bleekeri*, and provide valuable genetic resources for future studies on the evolution and conservation of high-altitude fish species such

as *T. bleekeri*.

Keywords: *Triplophysa bleekeri*, genome, genetic adaptation, population genomics

Introduction

The Qinghai-Tibetan Plateau (QTP), the largest and highest plateau in the world, is one of the most important world biodiversity centers [1]. The environments of QTP and its peripheral areas were affected significantly by the continuing uplifts, which is one of the most important driving forces for the biological evolution of organisms on the plateau [2]. The endemic species of the QTP present high adaptability to the harsh environmental conditions, such as low temperature, low oxygen supply, and high UV radiation by exhibiting cold tolerance, hypoxia resistance, enhanced metabolic capacity, and increased body mass [3-6].

An investigation into the biological evolution of organisms residing on the QTP and its peripheral regions will broaden our understanding of essential evolutionary questions regarding mechanisms of environmental adaptation and speciation. Phenotype comparisons were frequently used to study environmental adaptations in previous studies [7, 8]. In recent years, advancing genomic technology, especially third-generation sequencing techniques, has presented novel opportunities to explore the genetic basis of environmental adaptations. Many genomic studies of terrestrial animals on the QTP and its peripheral regions revealed that genes involved in hypoxia response, energy metabolism, and DNA repair were under positive selection and rapid evolution [9-11]. In those studies, high-quality genome and population resources are essential to understand critical biological processes for adaptations [11-13].

The QTP boasts of having many highland fish species, especially in the family Sisoridae, subfamily Schizothoracinae, and genus *Triplophysa* [14]. To date, there have only been several high-quality highland fish genomes reported based on long-read sequencing data, including *Glyptosternon maculatum* in the family Sisoridae, *Schizothorax o'connori* and *Oxygymnocypris stewartii* in the subfamily Schizothoracinae, and *Triplophysa tibetana* and *Triplophysa siluroides* in the genus *Triplophysa* [15-19]. *Triplophysa* is a highly diverse genus and the largest group of the subfamily Nemacheilinae [20]. There are 152 records for *Triplophysa* species in FishBase, and the majority are distributed on the QTP and its adjacent drainage areas from an elevation of 100 m to over 5,200 m [21]. Given the broad elevation distributions and species diversity, the *Triplophysa* genus offers an attractive study model not only to investigate the adaptive mechanisms of fish in high altitudes, but also to examine the similarities and differences between the adaptive mechanisms in different *Triplophysa* species. Previous studies have reported the genomic data of *T. siluroides* and *T. tibetana* without any emphasis on the genetic basis of high-altitude adaption [17, 18]. To date, environmental adaptations of *Triplophysa* species to high altitudes are not fully understood, and the genetic resources for the reference genome and population data remain insufficient. *Triplophysa bleekeri*, another member of the Nemacheilidae family, is mainly distributed in the stem streams and tributaries of the Yangtze and Jinsha rivers [22]. It exhibits different ecological and physiological characteristics compared with its relatives, *T. siluroides* and *T. tibetana* [23]. *T. bleekeri* has a wide distribution, from 200 m to 3,000 m [24], whereas *T. tibetana* and *T. siluroides* occur at elevations of 4,000 ~

5,000 m, and 3,000 ~ 4,000 m, respectively [17, 25]. Apart from altitude of habitation, there is a significant difference in habitat environments. *T. bleekeri* lives in the fast-flowing rivers, whereas *T. tibetana* and *T. siluroides* inhabit lakes and slow flowing rivers [26]. Reproduction biology in these *Triplophysa* species is also different; *T. tibetana* and *T. siluroides* spawn once a year (from June to July, and July to August, respectively), whereas *T. bleekeri* can spawn twice a year, with peak breeding seasons occurring from October to December and March to April [24]. The primary food source of *T. bleekeri* and *T. tibetana* are *Chironomus* larvae, caddis fly larvae, and diatoms, whereas *T. siluroides* feeds smaller fishes [25]. The genome resource for *T. bleekeri* will contribute to understanding its evolution and environmental adaption and explore the convergent genetic mechanisms of *Triplophysa* species in high-elevation adaption.

In this study, we generated the first chromosomal genome sequence of *T. bleekeri* using the combined technology of the Illumina, PacBio, and Hi-C. Evolutionary and comparative genomic approaches were applied to clarify the origin of *T. bleekeri*, and to investigate the potential signals of adaption. Further, the population genetics of *T. bleekeri* were also investigated to reveal the genetic divergence among different populations.

Materials and Methods

Samples and tissue collection

Triplophysa bleekeri individuals (**Fig. 1a;** NCBI: txid595395; fishbase ID: 56059) were obtained from the Daning River (31°09′26.58″N, 109°53′31.68″E), a tributary in the upper reaches of the Yangtze River, using brail nets (**Fig. 1b**). The fish were then

transferred to the Aquaculture Laboratory of Southwest University and reared in indoor tanks. To collect enough tissues for the genome and transcriptome sequencing, the largest female individual was used for library construction and sequencing. The fish was anesthetized with tricaine MS-222, and was immediately dissected to collect 12 types of tissues viz., brain, eye, skin, gill, heart, liver, trunk kidney, spleen, gut, muscle, gallbladder, and gonad. Tissues were quickly frozen in liquid nitrogen for more than one hour, and then stored at −80℃. Among these tissues, muscle tissue was used for genomic DNA sequencing and Hi-C library construction. Meanwhile, all tissue samples were used in the application of transcriptome sequencing to comprehensively characterize transcriptome. To understand the population structures of *T. bleekeri*, a total of 28 individuals were collected from three different reaches of Daning River, i.e., eleven, eleven, and six individuals from Lianghekou (LHK), Xixi (XX), and Baiyang (BY), respectively (**Fig. 1b**). These individuals were anesthetized with tricaine MS-222, and muscle tissue of each fish was collected as aforementioned.

Genome DNA extraction and sequencing library construction

DNA was extracted from muscle tissue using the phenol-chloroform DNA extraction method [27]. The Qubit (Thermo Fisher Scientific, Waltham, MA, USA) and Agilent Bioanalyzer 2100 (Agilent Technologies, Palo Alto, CA, USA) were used for evaluating the quantity and quality of DNA. For sequencing based on the Illumina HiSeq technology, a short-read sequencing library with an insert size of 250 bp was constructed using 1 μg of DNA. For sequencing on the PacBio SEQUEL platform (Pacific Biosciences, Menlo Park, CA, USA), the muscle DNA was used to construct

the long-read sequencing library. Briefly, 10 μg of *T. bleekeri* genomic DNA was used for 20-kb library preparation following the manufacturer's protocol (Pacific Biosciences), and the BluePippin Size Selection system (Sage Science, Beverly, MA, USA) was used for library size selection. DNA molecules from the largest individual were sequenced using the PacBio and Illumina platforms for genome assembly, and other samples were subjected to short-read whole-genome resequencing on the Illumina platform.

RNA extraction and sequencing library construction

RNA sequencing data provide important evidences for gene prediction in the genome [28]. To include as many expressed genes as possible, 12 tissue types, mentioned above, were used for the RNA sequencing library construction. RNA was isolated from the 12 tissue samples using TRIzol reagent (Invitrogen, USA). The quantity and quality of extracted RNA were determined using the Nanodrop ND-1000 spectrophotometer (LabTech, Holliston, MA, USA) and 2100 Bioanalyzer (Agilent Technologies, Palo Alto, CA, USA). Samples with a total RNA concentration ≥ 10 μg, and RNA integrity number ≥ 8 were used for sequencing. RNA molecules extracted from tissues were mixed in equal proportions for the following RNA library construction. RNA sequence library was constructed following the protocol of Paired-End Sample Preparation Kit (Illumina Inc., San Diego, CA, USA), which was identical to that employed in our previous study [29].

DNA and RNA library sequencing

The short-read DNA and RNA sequencing libraries were sequenced with the 150 bp paired-end (150PE) mode using the Illumina HiSeq X Ten platform (Illumina Inc.). The 20 kb long-read genome DNA SMRTbell libraries sequencing library was sequenced with the PacBio SEQUEL platform (Pacific Biosciences). The raw sequencing data were quality checked before the bioinformatics analysis. The high-throughput quality control (HTQC v0.90.8) package [30] was used to filter low-quality bases and reads, and sequences with adapters or low quality (average quality score < 20) were removed.

Genome size estimation

The genome size was estimated based on Illumina sequencing data using the *K*mer method before genome assembly. Raw Illumina reads were processed to remove adapter sequences, reads with more than 10% N bases, and reads with more than 50% low-quality bases (≤5). All filtered reads were used for *K*mer frequency analysis [31]. Using *K*mer size of 17, the *K*mer frequencies were obtained using Jellyfish v2.0 software [32]. *K*mers with a frequency of lower than 3 were eliminated as those likely resulted from sequencing errors. The genomic size was estimated based on the following formula: $G = (L - K + 1) \times n_{base} / (C_{Kmer} \times L)$, in which G is the estimated genome size, n_{base} is the total count of bases, C_{Kmer} is the expectation of *K*mer depth, L indicates the read length, and K represents *K*mer size. The revised genome size was calculated as follows: Revised Genome size = Genome size × (1-Error Rate).

***De novo* assembly of the *T. bleekeri* genome**

Long reads generated from the PacBio sequencing platform were used for *T.*

bleekeri genome assembly with the Falcon v0.3.0 package [33]. The assembled genome sequences were further polished with Arrow using long-read sequencing [34]; thereafter, two rounds of polishing using next-generation sequencing (NGS) short reads were performed with Pilon (Pilon, RRID:SCR_014731) v1.23 [35]. Finally, redundant genomic sequences were eliminated using Redundans v0.14a with the parameter overlap of 0.95 and an identity of 0.95 [36]. Completeness of the assembled genome was evaluated using BUSCO (BUSCO, RRID:SCR_015008) v3.0 [37]. The database of actinopterygii_odb9 was used for the BUSCO analysis.

Chromosome assembly using Hi-C technology

Muscle tissue (1 g) of *T. bleekeri* was collected for PacBio sequencing and was used for Hi-C library construction. The Hi-C processes, including crosslinking, lysis, chromatin digestion, biotin marking, proximity ligations, crosslinking reversal, and DNA purification, were performed using the protocol described in previous studies [38]. The purified and enriched DNA was used for sequencing library construction. The library was sequenced using the Illumina HiSeq X Ten platform (Illumina), and the short-reads were then mapped to the polished genome of *T. bleekeri* with Bowtie (Bowtie, RRID:SCR_005476) v1.2.2. The chromosomal assembly using interaction frequency matrix extracted from the Hi-C read mapping was performed according to a previously-reported methodology [38].

Repetitive element annotation

The *de novo* prediction and homology prediction were combined to annotate the repetitive sequences in the *T. bleekeri* genome. RepeatModeler (RepeatModeler,

RRID:SCR_015027) v2.0.1 [39] was used for the detection of *de novo* repetitive elements in the *T. bleekeri* genome. The detected genome repeats were combined with RepBase library [40], as a comprehensive library for the final repetitive elements prediction in the *T. bleekeri* genome, using the RepeatMasker (RepeatMasker, RRID:SCR_012954) v4.1.1 software [41]. Transposons were predicted using ProteinMask, and the tandem repeats were identified in the genome using Tandem Repeat Finder v4.10 [42].

Protein coding and non-coding gene prediction

The *ab initio* prediction, homology prediction, and RNA-sequencing-based methods were used for protein-coding gene annotation. Gene models for protein-coding genes were first predicted in the *T. bleekeri* genome using Augustus (Augustus: Gene Prediction, RRID:SCR_008417) v2.5.5 [43]. Five closely related fish species, viz., common carp (*Cyprinus carpio*), zebrafish (*Danio rerio*), Japanese medaka (*Oryzias latipes*), green spotted puffe (*Tetraodon nigroviridis*), and threes pined sticklebacks (*Xiphophorus maculatus*), were used for the homology-based protein-coding gene prediction. Protein sequences from those species, available in public databases, were mapped to the genome using TBLASTN [44] and GeneWise (GeneWise, RRID:SCR_015054) [45]. Thereafter, comprehensive transcriptome sequencing data for multiple-tissues were aligned to the genome, and gene models were generated using the TopHat (TopHat, RRID:SCR_013035) v2.1.1 package [46] and Cufflinks (Cufflinks, RRID:SCR_014597) v2.2.1 [47]. The integration and redundancy elimination for the gene models predicted using the above methods were performed

using the MAKER package (MAKER, RRID:SCR_005309) [48, 49]. We only selected genes with start and stop codons, and genes with internal stop codons were removed. Only genes with complete sequences and 70% overlaps among different gene model prediction methods will be retained as high-quality gene models.

Four types of non-coding RNAs, including microRNAs (miRNA), transfer RNAs (tRNA), ribosomal RNAs (rRNA), and small nuclear RNAs (snRNA), were also predicted in the *T. bleekeri* genome using tRNAscan-SE (tRNAscan-SE, RRID:SCR_010835) v1.3.1 [50] and using Infernal (Infernal, RRID:SCR_011809) v1.1.3 [51] with the Rfam database [52].

Functional annotation of protein-coding genes

The NCBI non-redundant protein, Swissport, and TrEMBL databases [53] were used as protein databases for the biological function annotation using BLAST v2.10.1 packages [54]. The E-value of 1e-5 was used as the threshold for homolog identification. Gene Ontology (GO) [55] and Kyoto Encyclopedia of Genes and Genomes (KEGG) [56] assignments were performed using Blast2GO (Blast2GO, RRID:SCR_005828) software [57].

Gene family clustering and phylogenetic analysis

Coding sequences annotated from whole-genome sequences for the closely related species were extracted from genome sequences. Gene family clustering was performed for *T. bleekeri* with eight fish species living in non-QTP regions, viz., zebrafish, Japanese medaka, elephant shark (*Callorhinchus milii*), spotted gar (*Lepisosteus oculatus*), Atlantic cod (*Gadus morhua*), platyfish (*X. maculatus*), tiger puffer

(*Takifugu rubripes*), and large yellow croaker (*Larimichthys corcea*) by the Orthomcl v1.2 pipeline [58] with default settings. The single-copy orthologs across all species were selected for gene family, phylogenetic, and evolutionary analyses. Briefly, proteins of these genes were aligned with MUSCLE (MUSCLE, RRID:SCR_011812) v3.8.31 [59] and were then transformed into alignments of nucleotide sequences with pal2nal [60] on the basis of the corresponding coding sequences. Next, non-conservative regions were removed using Gblocks (Gblocks, RRID:SCR_015945) [61] with default settings, and the conservative regions were concatenated and fed in RaxML (RAxML, RRID:SCR_006086) v8.2.10 [62] to deduce the phylogenetic relationships of these species using a GTRGAMMA model. Rapid bootstrap runs (100 times) were performed to test the robustness of the topology [63]. Based on the topology and the alignment matrix, their divergence times were deduced using MCMCTREE included in the PAML (PAML, RRID:SCR_014932) v1.3.1 package [64] with calibration points set by consulting the TimeTree database. *Danio rerio* and *Larimichthys crocea* (255-205 Ma), *Oryzias latipes* and *Larimichthys crocea* (115-105 Ma), *Lepisosteus oculatus* and *Danio rerio* (338-291 Ma) and *Callorhinchus milii* and *Danio rerio* (497-450 Ma) were used as calibration points for the divergence time estimation for other species.

To investigate the evolutionary relationships within genus *Triplophysa*, we also added another four *Triplophysa* genus fish species to the phylogenetic analysis. Since the genome of *T. xichangensis* and *T. scleroptera* have not been reported, we downloaded the short-reads of the transcriptomes of those two species from the NCBI sequence read archive (SRA) and conducted *de novo* assembly using Trinity (Trinity,

RRID:SCR_013048) v2.11.0 [65] with default settings. Longest transcripts for each gene were used in the following phylogenetic analysis. The single-copy orthologs across all species were used for phylogenetic tree reconstruction and divergence time estimation following the same method as described above.

Gene family expansion and contraction in the *T. bleekeri* genome

To identify expanded and contracted gene families in the *T. bleekeri* genome, we compared gene families in the *T. bleekeri* genome to those fish species living in non-QTP regions viz., including elephant shark, spotted gar, zebrafish, Japanese medaka, platyfish, tiger puffer, large yellow croaker, Atlantic cod, green spotted puffe, and threes pined sticklebacks. CAFE v4.2.1 [66] was used to analyze the expansion and contraction of gene clusters in the *T. bleekeri* genome using a probabilistic model. A GO enrichment analysis was performed on expanded and contracted genes using the topGO v2.40.0 package [67]. The enrichment of genes in KEGG pathways was also analyzed using the KOBAS (KOBAS, RRID:SCR_006350) v1.2.0 [68].

Positively selected genes (PSGs) in genomes of *Triplophysa* species

MUSCLE v3.8.31 was used for multi-protein sequence alignment among the *T. bleekeri* genes and their orthologs, and compared to eight fish species living in non-QTP regions used in the gene family clustering analysis. Conserved coding sequence (CDS) alignments of each single-copy gene family were extracted using Gblocks [69] and used for further identification of PSGs. The ratios of nonsynonymous to synonymous substitutions (K_A/K_S, or ω) were estimated for each single-copy orthologous gene using the CodeML program with the branch-site model as

implemented in the PAML package. A likelihood ratio test was conducted, and the false discovery rate (FDR) correction was performed for multiple comparisons. Genes with a corrected *P*-value < 0.05 were defined as PSGs. The genes putatively influenced by positive natural selection of *T. tibetana* and *T. siluroides* were also identified using the identical method. The functional annotation of PSGs for *T. bleekeri*, *T. tibetana*, and *T. siluroides* was also conducted using the same approach with the gene family expansion and contraction analysis.

Whole-genome re-sequencing and population genetics

Raw reads of samples subjected to resequencing were quality controlled as mentioned previously. The filtered short reads were mapped using BWA mem (BWA, RRID:SCR_010910) v0.7.17-r1188 with default settings for each individual, followed by the marking of duplicates with Picard (Picard, RRID:SCR_006525). Regions near INDELs were thought to be poorly aligned and were identified and realigned using GATK (GATK , RRID:SCR_001876) v4.1.8.1 [70]. GATK was also used to call SNPs and INDELs based on the alignments. The SNPs and INDELs were then filtered by these parameters: QUAL (phred quality) > 30, QD (quality score divided by depth to comprehensively evaluate the quality and depth) > 2, DP (read depth)> 5, FS (phred-scaled p-value using Fisher's Exact Test to detect strand bias for reads) < 60, MQ (mapping quality to evaluate read alignment) >40, SOR (strand odds ratio to evaluate strand bias for reads) < 4.0. The identified SNPs were filtered using SNPhylo v20180901 [71] with default settings, except for LD_threshold and Minimum_depth_of_coverage, which were set to 0.8 and 5, respectively. Next, the

principal components analysis (PCA) clusters and population structure for these individuals were deduced with Plink (PLINK, RRID:SCR_001757) v1.9 [72] and Admixture [73, 74] with default settings, respectively. Their phylogenetic relationships were recovered using the neighbor-joining (NJ) method with MEGA4 [74], and bootstrap resampling (100 times) was performed to test the robustness of the tree topology.

Historical effective population size inference for *T. bleekeri*

Historical effective population size of *T. bleekeri* was estimated based using Pairwise Sequentially Markovian Coalescent (PSMC) v0.6.5 software [75].We used the data for whole-genome variants of individuals for the genome assembly. The consensus sequences were generated using vcfutils.pl (vcf2fq -d 10 -D 300). The fq2psmcfa tool was used to create the input file for PSMC modelling. Sequences were used as the input for the PSMC estimates using 'psmc' with the options -N25 -t15 -r5. The reconstructed population history was plotted using 'psmc_plot.pl' with the generation time of 2 year and rate of 4×10^{-9} substitutions per synonymous site per year. The mutation rate was estimated from the gene comparison of *T. bleekeri* and *D. rerio*. Bootstrapping was conducted by randomly sampling with replacement 5- Mb sequence segments and 100 bootstrap replicates were performed.

Selection sweep analysis for populations

To identify genome-wide selective sweeps among populations, we calculated the genome-wide distribution of fixation index (F_{ST}) values and θπ ratios using SNPs from different populations. The F_{ST} values were Z-transformed as follows: Z (F_{ST}) = (F_{ST} -

μF_{ST}) / σF_{ST}, in which μF_{ST} was the mean F_{ST}, and σF_{ST} was the standard deviation of F_{ST}. The $\theta\pi$ ratios were log2-transformed. Subsequently, we scanned the genome in a 1 kb sliding scale, and estimated and ranked the empirical percentiles of Z (F_{ST}) and $\log_2$ ($\theta\pi$ ratio) in each window. We considered the windows with the top 1% Z (F_{ST}) and $\log_2$ ($\theta\pi$ ratio) as candidate outliers under strong selective sweeps. Genes residing in the outlier regions were considered as the candidate functional genes. The GO and KEGG enrichment were carried out by cluster Profiler v3.14.3 [76] and DAVID v6.8 [77].

Results

DNA and RNA library sequencing

81.69 Gb genomic (~120X) and 10.6 Gb transcriptomic short-reads were generated for the following genome size estimation and annotation (**Table 1**). We also obtained 100.87 Gb genomic long-reads from the PacBio platform, with a rough coverage of 160X for the *T. bleekeri* genome (**Table 1**). The mean and N50 length of the long-reads were 5.8 kb and 16 kb, respectively (**Table 1** and **Supplementary Fig. S1**).

Genome size estimation

To determine the possible sample contamination, 10,000 NGS short-reads were randomly selected for an NCBI nt database search. *Cyprinus*, *Danio*, and *Sinocyclocheilus* represent the top three sources of best hits, ruling out the obvious contamination during library construction and sequencing. Using genomic short-reads generated from the Illumina platform, 59.8 million *K*mers were generated. The genome of *T. bleekeri* was estimated as 632.5 Mb, with a heterozygosity ratio of 0.26% and

repeat content of 42.2% (**Supplementary Fig. S2**). Based on the above genome character estimation, the genome of *T. bleekeri* was mid-sized with low heterozygosity.

De novo **assembly of the *T. bleekeri* genome**

Using genomic PacBio long-reads for *T. bleekeri*, we assembled a 628 Mb genome with 856 contigs and an N50 length of 3.82 Mb (**Table 2**). Among these contigs, the longest contig for the genome was 15.5 Mb. The completeness of the assembled genome was evaluated using BUSCO v3.0 [37] with the actinopterygii_odb9 database, indicating that 92.9% of BUSCO genes were identified in the assembled genome (**Supplementary Fig. S3**).

Chromosome assembly using Hi-C technology

Hi-C technology recruits interaction information among different chromosome regions and assumes that the interactions for nearby regions are more prevalent than distant regions. In this study, 82.9 Gb sequencing data were obtained via Hi-C library sequencing. Based on the interacting information, a chromosome assembly of 628 Mb with a scaffold N50 length of 22.9 Mb was obtained (**Supplementary Fig. S4**). More than 596.9 Mb sequences were anchored upon 25 chromosomes, highlighting a high chromosome anchoring rate of 96.2% on the base level.

Repetitive element annotation

The annotation pipeline showed that more than 17.9 Mb of the genome sequences were predicted as tandem repeats, covering about 2.8% of the genome, and finally 203.2 Mb, accounting for roughly 32.4% of the genome, were annotated as repetitive elements

in the *T. bleekeri* genome (**Supplementary Table S1**). Specifically, there are 17.2% DNA transposons (107.8 Mb), 5.8% of long interspersed nuclear elements (LINE) (36.4 Mb), 0.68% short interspersed nuclear elements (SINE) (4.3 Mb), and 6.93% long terminal repeats (LTR) (43.5 Mb).

Protein- and non-coding gene prediction, and functional annotation

For predicting protein-coding genes in the *de novo* assembled genome, 10.6 Gb short-read transcriptome data from 12 tissues was generated. Based on the *de novo*, homolog, and RNA-seq data methods, a total of 20,274, 27,243, and 15,875 protein-coding genes were predicted, respectively. After integration and redundancy elimination, 21,198 protein-coding genes were predicted in the *T. bleekeri* genome (**Supplementary Table S2**).

Of the 21,198 protein-coding genes, roughly 93.0%, 96.9%, and 90.9% displayed homologous sequences in the NCBI NR, TrEMBL, and Swissprot databases, respectively. Additionally, 89.2% contained InterPro domains, and 46.9% were assigned with GO terms. Overall, more than 97.3% of the protein-coding genes were functionally annotated by at least one method (**Supplementary Fig. S5**). The non-coding genes have received increased attention in the recent years, since accumulating evidence suggests that many of them play crucial roles in a variety of biological process [78]. In this study, all the possible non-coding DNA sequences were predicted based on the *de novo* prediction strategies, and are summarized in **Supplementary Table S3**.

Gene family clustering and phylogenetic analysis of *T. bleekeri*

Using the whole-genome and transcriptome data of four other *Triplophysa* species,

viz., *T. tibetana*, *T. siluroides*, *T. scleroptera*, and *T. xichangensis*, and the eight other fish species living in non-QTP regions, we performed the gene family clustering for those species. As a result, we identified 1, 364 single-copy orthologs among those fish species.

We then investigated the evolutionary relationship of *T. bleekeri* with respect to other *Triplophysa* and the non-QTP species. Using single-copy genes among species, a concatenated alignment matrix, was generated with a total length of 73,887 bps, which was used for the phylogenetic analysis and divergence time estimation. The result showed that *Triplophysa* species are phylogenetically closer to *D. rerio*, and that *T. siluroides* is a basal species within the *Triplophysa* group. Divergence time estimation showed that *T. bleekeri* diverged from their common ancestor, *T. scleroptera* and *T. xichangensis*, around 25.2 million years ago (Ma) (**Fig. 2**).

Genes under natural positive selection

We identified 788 PSGs in the *T. bleekeri* genome. The functional analysis on the KEGG and GO parameters showed that several categories associated with nucleotide metabolism and DNA repair were significantly enriched (**Supplementary Table S4 and S5**). Additionally, the PSGs were also enriched in immune response, such as MyD88-dependent toll-like receptor signaling pathway (**Supplementary Table S4)**. Concomitantly, 969 and 1,253 PSGs were identified for *T. tibetana* and *T. siluroides*, respectively. Among those genes, 197 genes were identified as shared PSGs for the three *Triplophysa* species (**Fig. 3a**).

To detect candidate PSGs for *Triplophysa* ancestral lineage, we also performed the

PSG identification for the common ancestor of the *Triplophysa* with the branch-site model in the PAML. As a result, we identified 439 PSGs for *Triplophysa* ancestral lineage. Interestingly, we found that only 35 shared PSGs for the three *Triplophysa* species were identical with *Triplophysa* lineage PSGs (**Fig. 3b**). The functional analysis with respect to biological pathways for the three *Triplophysa* species showed that those genes were significantly enriched for various processes including protein digestion and absorption, Fanconi anemia pathway, and salivary secretion (**Fig. 3c**). Twenty-five biological pathways, including peroxisome, autophagy, non-homologous end-joining, homologous recombination, basal transcription, ribosome biogenesis and spliceosome, were enriched for PSGs of *Triplophysa* ancestral lineage (**Fig. 3c**). The homologous recombination and basal transcription factor pathways were both enriched for *Triplophysa* lineage PSGs and the three *Triplophysa* species shared PSGs (**Fig. 3c**).

Gene family expansion and contraction in the *T. bleekeri* genome

Following the Orthomcl pipeline, 21,862 ortholog groups were obtained after gene family clustering with ten fish species from non-QTP regions. Gene family analysis showed that 1,533 and 2,401 gene families were significantly expanded and contracted in *T. bleekeri*, respectively (**Supplementary Fig. S6**). The functional enrichment of expanded gene families was analyzed using GO and KEGG. The expanded gene families were primarily enriched in categories of metabolism and immune regulation (**Supplementary Tables S6** and **S7**). The categories of metabolism include fatty acid metabolism (arachidonic acid metabolism and glycosphingolipid biosynthesis), carbohydrate metabolism (glycosaminoglycan biosynthesis and glycan degradation),

and amino acid metabolism (RNA transport). The categories of immune regulation include the Hippo signaling pathway (corrected *p*-value = 2.40E-03), necroptosis, and Vitamin B6 metabolism (corrected *p*-value = 8.90E-03). The contracted gene families were mainly made up of several signaling pathways, including the MAPK signaling pathway, calcium signaling pathway, adrenergic signaling in cardiomyocytes, GnRH signaling pathway, and retrograde endocannabinoid signaling (**Supplementary Tables S8** and **S9**).

Historical effective population size for *T. bleekeri* during the QTP formation

We used the whole-genome short-read sequencing data based on the sample used for genome assembly to obtain the genome-wide genotype data. Then, those variants were used to probe the profiles of historical effective population size for *T. bleekeri* during the QTP formation. We used the gene comparison between *T. bleekeri* and *D. rerio* to estimate the mutation rate. As a result, we estimated the mutation rate of 4×10^{-9} for *T. bleekeri*. PSMC analysis performed using the above data showed that the effective population size of *T. bleekeri* increased more than 0.7 Ma, and reached a peak of 70×10^4 around 0.6–0.7 Ma. However, the *T. bleekeri* population size experienced a dramatic drop afterwards to 1×10^4 from 0.6 Ma to 60, 000 years ago (**Fig. 4**). The effective population size decline was consistent with the accelerating QTP uplifts around 1 Ma [79] and the quaternary glaciation spaning the Pleistocene (2.6–0.11 Ma) and Holocene (0.11–0 Ma) [19, 80]. We speculate that both the geotectonic movements and temperature fluctuations during the period exerted intense survival pressure for the ancient *T. bleekeri* populations, leading to the roughly 70 times effective population

size drop during the period.

Population genetics analysis of *T. bleekeri*

The high-quality SNPs were obtained according to the filtering criteria set previously, and were used to deduce the population structures of *T. bleekeri*. As a result, more than 34 million short-reads were obtained for 28 individuals, and more than 3 million SNPs were detected for all individuals. The phylogeny reconstruction analyses based on whole-genome SNPs showed that individuals from population LHK and XX clustered together forming two neighboring groups, whereas individuals from population BY formed another cluster (**Fig. 5a**). The PCA clusters (**Fig. 5b**) also suggested that the first two principal components could successfully separate the individuals in population BY from those in population LHK and XX. In addition, genetic structure analysis also indicated that gene flow between population BY and the other two populations was might be limited (**Fig. 5c**).

To identify putative signals of differential selection among populations, we also performed the selective sweep analysis for BY, LHK and XX populations (**Fig. 6a**). Based on Fst comparison among those populations (**Supplementary Table S10**), we identified genomic regions (~1 kb in length) that scored in the top 1% (**Supplementary Fig. S7**). As a result, 1, 734, 3, 009, and 3, 244 regions (1 kb), harboring 474, 878, and 957 functional candidate genes were identified to be significantly genetically differentiated for LHK-XX, LHK-BY, and XX- BY comparisons, respectively. Genomic regions with less differentiation identified in LHK-XX comparison were consistent with the above phylogenetic analysis. The GO and KEGG pathway

functional analyses showed 20, 25, and 31 significant biological pathway enrichments for LHK-XX, XX-BY, and LHK-BY comparisons, respectively **(Fig. 6b**, **Supplementary Table S11, S12, and S13)**. Six enriched biological pathways were shared in LHK-BY and XX-BY comparisons but not in LHK-XX one, viz., ubiquitin mediated proteolysis, tight junction, starch and sucrose metabolism, melanogenesis, longevity regulating pathway – mammal, and circadian rhythm (**Fig. 6c, Supplementary Table S12 and S13**). Five enriched biological pathways were shared for all comparisons, viz., axon guidance, long-term potentiation, Rap1 signaling pathway, circadian entrainment, and calcium signaling pathway (**Fig. 6b**). Besides, the alpha-trehalose glucohydrolase (treh), β-catenin (ctnnb1), and lymphoid enhancer-binding factor 1 (lef1) gene exhibited significant genetic differentiation in LHK-BY and XX-BY comparison but not in LHK-XX, implying these genes might be related to the living environments for BY (Fig. 6d).

Discussion

In this study, we presented the chromosome-level genome assembly of *T. bleekeri* with a contig N50 of 3.1 Mb and a scaffold N50 of 22.9 Mb. The N50 lengths of contigs of *T. bleekeri* genome assembly were much longer than previously reported genome assemblies of *T. tibetana* [17]. Twenty-five chromosomes were obtained with the mounting rate up to 96.2%, and the assembled chromosome number was consistent with the karyotype of *T. bleekeri* (unpublished data), which suggests that the present analysis resulted in successful assembly of *T. bleekeri* genome to the chromosome level. The completeness of the genome was also evaluated, confirming the high quality of the

assembled *T. bleekeri* genome. The combined results of the homology-based and *de novo* predictions showed that repetitive sequences accounted for 32.4% of the genome. Among them, DNA transposons represented the most abundant tandem repeats, which was also observed in *T. tibetana* [17]. Within the genome, 21,198 protein-coding genes were predicted, of which 97.3% could be functionally annotated. Overall, this genome assembly and annotation provides valuable data to the genomic resources currently available for the study of phylogeny and environmental adaptations of *Triplophysa* species.

The phylogenetic analysis results indicated that the *Triplophysa* genus formed a clade with *D. rerio*, and that *T. bleekeri* was most closely related to *T. tibetana* and *T. scleroptera*. The divergence time estimation indicated that *T. siluroides* diverged from their common ancestor roughly 38.8 Ma, occupying a basal position in the *Triplophysa* genus. The extensive QTP was elevated by more than 4,000 m about 40 Ma [81], and this time is consistent with the divergence of *T. siluroides*. Therefore, we speculated that the speciation of *Triplophysa* was likely triggered by the uplifting of the QTP [82].

Upliftment of the QTP profoundly induced on climatic and environmental changes of the plateau and its peripheral regions, including low oxygen and low temperature [83]. The oxygen content of air is inadequate in the QTP, while investigations into water quality indicated that high dissolved oxygen concentration exists in the QTP water [84-87]. Therefore, we speculated that thermal stress may present a major factor in natural selection for fish species in the QTP and its peripheral regions. Although *Triplophysa* species are widely distributed in different regions, these regions are all generally

characterized by a cold environment [23, 88]. However, to the best of our knowledge, only a few studies have been conducted to explore the genetic basis of adaptation of *Triplophysa* species to low temperatures. Through the comparative analysis of the genome with other fish species, we found that the expanded gene families of *T. bleekeri* were significantly (p < 0.05) enriched in fatty acid metabolism, including glycosphingolipid biosynthesis and arachidonic acid metabolism pathways. The glycosphingolipid located in the bilayer lipid membrane is a major structural component of cell membranes [89], whereas arachidonic acid, an integral constituent of biological cell membranes, aids in the maintenance of cell membrane fluidity even at low temperatures [90]. Our results suggest that the increased number of genes related to fatty acid metabolism might be responsible for maintaining membrane structure and improving membrane fluidity under cold environments.

In the genome of *T. bleekeri*, significant expansion was also observed in Hippo signaling pathway gene family which participates in regulating innate immunity [91, 92]. These results suggest that *T. bleekeri* may tend to increase gene numbers in certain families related to immune response for improving the defense against pathogens. It is notable that genes involved in innate immunity, such as toll-like receptor signaling pathway genes, all underwent positive selection in *T. bleekeri*, *T. tibetana,* and *T. siluroides*. Similar results were also observed in previous transcriptomic studies of Tibetan Schizothoracinae species, *Gymnocypris przewalskii,* and *G. przewalskii ganzihonensis* [93, 94]. These results indicated that the adaptive evolution of innate immunity might play crucial roles in the highland adaption of fish.

Low temperatures and UV radiation can cause DNA damage [95], and DNA damage response and repair pathways may show functional adaption. Within the three *Triplophysa* species, the PSGs were enriched in the functional categories of nucleotide excision repair, non-homologous end-joining, homologous recombination, and Fanconi anemia pathways (**Supplementary Table S4 and S5**). These pathways all participate in DNA repair, of which non-homologous end-joining and homologous recombination are the two main pathways for repairing double-strand break [96], and Fanconi anemia pathway is essential for the repair of DNA interstrand crosslinks [97]. PSGs influencing DNA repair may contribute to DNA integrity and genomic stability under high-altitude environments with low temperatures and intense UV radiation. Our results suggest that *Triplophysa* species have evolved an integrated DNA-repair mechanism to adapt to high-altitude environments. The previous studies also showed that genes involved in the DNA repair were under positive selection pressure in many species living at high altitudes, such as the snub-nosed monkey [98] and the Tibetan hot-spring snake [11]. It indicated that DNA damage caused by the environment is a common stress that animals in high-altitude regions need to cope with. We also identified 197 PSGs shared by the three *Triplophysa* species (**Fig. 3A**), indicating that those naturally selected genes might have originated from their common ancestor, and that *Triplophysa* species were genetically convergent on PSGs. We found many species-specific PSGs for three *Triplophysa* species. The result implies the requirement of a distinct ecological niche for *T. bleekeri*, *T. tibetana*, and *T. siluroides*. Based on the generally used genomic comparison methods, hundreds of PSGs for *Triplophysa* species were identified in this

investigation. However, a previous study has shown that ancient demographic fluctuation could generate severe overestimation of selective signatures [99]. Therefore, PSG identification in this work might have been influenced by the demographic scenarios of *Triplophysa* species. It is worth estimating the demographic fluctuation to PSG identification, and comparing the current methods for potential biases.

In addition to comparative genomics analyses, the relationships among populations of *T. bleekeri* were analyzed to probe possible differences in genetic structures. Population structure analysis divided 28 *T. bleekeri* samples into two clusters, with individuals from the LHK and XX population grouped together, and individuals from BY population forming the other cluster. Both PCA and structure analyses corroborated these findings. The BY population was separated from the LHK and XX population, and the observed admixture of genetic lineages was limited (K=3). These results could be because LHK and XX are directly connected by the river, and gene flow between individuals residing in the two places occurs more frequently. The difference between BY population and the LHK and XX populations might be attributed to the relatively limited gene flow caused by natural and artificial barriers among those populations. The Daning River measures a height of up to 1,648 m, which flows through many narrower canyons[100]. Therefore, the geographical barriers formed by canyons and shallows could contribute to the diminished interaction among those populations. More importantly, artificial barriers, such as cities and dams, could also weaken the migrations between the BY and LHK/XX populations. Therefore, the whole-genome resequencing data of *T. bleekeri* provided a valuable genetic resource to reveal that

geographical and artificial barriers could distinctly influence genetic exchange among populations.

The selective sweep analysis showed that genomic differentiation of LHK-XX was nonintensive compared to that of the BY population, which is consistent with the above population phylogenetic analysis. Notably, we identified six shared enriched biological pathways for LHK-BY and XX-BY comparisons but not in LHK-XX. The natural gorge might change the water flow and biodiversity of environments, and human activity could as well influence the nutrition supplies and circadian rhythm for local fish populations directly.

In conclusion, we present a chromosomal-scale genome assembly of *T. bleekeri*, a representative high-altitude fish. Evolutionary, comparative, and population genomic analyses were performed to investigate the evolution, environmental adaption, and genetic diversity of *T. bleekeri*. Our results provide insights into how fish adapt to the high-altitude environment, and the genomic data serves as a valuable resource for further study on functional validation of candidate genes contributing to environmental adaption.

Ethics Statement

All experimental protocols were approved by the School of Life Sciences, Southwest University (Chongqing, China), and the studies were carried out in accordance with the Guidelines of Experimental Animal Welfare from Ministry of Science and Technology of People's Republic of China (2006) and the Institutional Animal Care and Use Committee protocols from Southwest University (2007).

Acknowledgement

This work was supported by the Financial Program of Ministry of Agriculture and Rural Affairs of China (Grant No. YYJZHC201921301350063), National Natural Science Foundation of China (Grant No. 31602207), and Research Innovation Program for College Graduates of Chongqing (Grant No. CYB19079).

Author contributions

ZJ Wang conceived and designed the study; DY Yuan and SJ Xiao collected the samples; DY Yuan and SJ Xiao performed molecular experiments; SJ Xiao performed the bioinformatics analysis, including genome size estimation, genome assembly, annotation, and gene prediction; DY Yuan, SJ Xiao, and ZJ Wang wrote the manuscript. All authors read and approved the final manuscript for submission.

Competing interests

All authors declare that they have no competing interests.

Data accessibility

The genomic, transcriptome, and Hi-C sequencing reads generated from the PacBio and Illumina platforms are available in the NCBI SRA database under the Accession no. SRP200140. The final chromosome assembly was submitted to NCBI (BioProject ID PRJNA545014, assembly VFQW00000000). Supporting data, including assembly and annotation files, are also available via the GigaScience database, GigaDB [101].

Abbreviations

BUSCO: Benchmarking universal single-copy orthologs; CAFE: Computational analysis of gene family evolution; CDS: Conserved coding sequence; FDR: False discovery rate; GO: Gene ontology; Hi-C: High-throughput chromosome conformation capture; KEGG: Kyoto encyclopedia of genes and genomes; LINE: Long interspersed nuclear elements; LTR: Long terminal repeats; QTP: Qinghai-Tibetan Plateau; PAML: Phylogenetic analysis by maximum likelihood; PCA, Principal components analysis; PSG: Positively selected genes; PSMC: Pairwise Sequentially Markovian Coalescent; SINE: Short interspersed nuclear elements; SRA: Sequence read archive.

References

1. Myers N, Mittermeier RA, Mittermeier CG, et al. Biodiversity hotspots for conservation priorities. *Nature*. 2000;403(6772):853.
2. Zhao Z and Li S. Extinction vs. Rapid radiation: The juxtaposed evolutionary histories of coelotine spiders support the Eocene–Oligocene orogenesis of the Tibetan Plateau. *Syst Biol*. 2017;66(6):988-1006.
3. Beall CM. Adaptation to high altitude: phenotypes and genotypes. *Annu Rev Anthropol*. 2014;43:251-72.
4. Monge C and Leonvelarde F. Physiological adaptation to high altitude: oxygen transport in mammals and birds. *Physiol Rev*. 1991;71(4):1135-72.
5. Wu T and Kayser B. High Altitude Adaptation in Tibetans. *High Alt Med Biol*. 2006;7(3):193-208.
6. Beall CM. Two routes to functional adaptation: Tibetan and Andean high-altitude natives. *Proc Natl Acad Sci U.S.A*. 2007;104:8655-60.
7. Ding CZ, Jiang XM, Chen L, et al. Growth variation of *Schizothorax dulongensis* Huang, 1985 along altitudinal gradients: implications for the Tibetan Plateau fishes under climate change. *J Appl Ichthyol*. 2016;32(4):729-33.
8. Deng H, Yue X, Chen D, et al. Growth characteristics and feed habit of *Triplophysa stenura* in Nujiang River. *Freshw Fisheries*. 2010;40(1):26-33.
9. Li M, Tian S, Jin L, et al. Genomic analyses identify distinct patterns of selection in domesticated pigs and Tibetan wild boars. *Nat Genet*. 2013;45(12):1431.
10. Qiu Q, Zhang G, Ma T, et al. The yak genome and adaptation to life at high altitude. *Nat Genet*. 2012;44(8):946.
11. Li JT, Gao YD, Xie L, et al. Comparative genomic investigation of high-elevation adaptation in ectothermic snakes. *Proc Natl Acad Sci U.S.A*. 2018;115(33):8406-11.
12. Liu ZJ, Liu SK, Yao J, et al. The channel catfish genome sequence provides insights into the evolution of scale formation in teleosts. *Nat Commun*. 2016;7:11757.

13. Sun YB, Fu TT, Jin JQ, et al. Species groups distributed across elevational gradients reveal convergent and continuous genetic adaptation to high elevations. *Proc Natl Acad Sci U.S.A*. 2018;115(45):E10634-E41.

14. Wu YF and Tan QJ. Characteristics of the fish-fauna of the characteristics of Qinghai-Xizang plateau and its geological distribution and formation. *Acta Zool Sinica*. 1991;37:135-52.

15. Liu HP, Liu QY, Chen ZQ, et al. Draft genome of *Glyptosternon maculatum*, an endemic fish from Tibet Plateau. *GigaScience*. 2018;7(9):giy104.

16. Liu HP, Xiao SJ, Wu N, et al. The sequence and de novo assembly of *Oxygymnocypris stewartii* genome. *Sci Data*. 2019;6:190009.

17. Yang X, Liu H, Ma Z, et al. The chromosome-level genome assembly of *Triplophysa tibetana* , a fish adapted to the harsh high-altitude environment of the Tibetan plateau. *Mol Ecol Resour*. 2019;19(4):1027-36.

18. Yang L, Wang Y, Wang T, et al. A chromosome-scale reference assembly of a Tibetan loach, *Triplophysa siluroides*. *Front Genet*. 2019;10:991.

19. Xiao S, Mou Z, Fan D, et al. Genome of Tetraploid Fish Schizothorax o'connori Provides Insights into Early Re-diploidization and High-Altitude Adaptation. *iScience*. 2020;23(9):101497.

20. Nelson JS, Grande TC and Wilson MV. Fishes of the World. John Wiley & Sons; 2016.

21. He CL, Song ZB and Zhang E. *Triplophysa* fishes in China and the status of its taxonomic studies. *Sichuan J Zool*. 2011;30(1):150-5.

22. He XF, He JS and Yan TM. Reproductive characteristic of *Triplophysa bleekeri* in mabian river. *J Southwest China Norm Univ*. 1999;24(1):69-73.

23. Xiao H and Dai YG. A Review of Study on Diversity of *Triplophysa* in China. *Fisheries Sci*. 2011;30(1):53-7.

24. Wang ZJ, Huang J and Zhang YG. The reproductive traits of *Triplophysa bleekeri* in the Daning River. *Freshw Fisheries*. 2013;43(5):8-12.

25. Zhu S. The loaches of the subfamily Nemacheilinae in China (Cypriniformes: Cobitidae). Jiangsu Science and Technology Publishing House; 1989.

26. Wu YT, Tang QJ. Characteristics of the fish-fauna of the characteristics of Qinghai-Xizang Plateau and its geological distribution and formation. *Acta Zool Sinica*. 1991;2:135-152.

27. Xiao SJ, Wang PP, Dong LS, et al. Whole-genome single-nucleotide polymorphism (SNP) marker discovery and association analysis with the eicosapentaenoic acid (EPA) and docosahexaenoic acid (DHA) content in *Larimichthys crocea*. *PeerJ*. 2016;4:e2664.

28. Denoeud F, Aury J-M, Da Silva C, et al. Annotating genomes with massive-scale RNA sequencing. *Genome Bio*. 2008;9(12):R175.

29. Xiao SJ, Han ZF, Wang PP, et al. Functional marker detection and analysis on a comprehensive transcriptome of large yellow croaker by next generation sequencing. *PloS One*. 2015;10(4):e0124432.

30. Yang X, Liu D, Liu F, et al. HTQC: a fast quality control toolkit for Illumina sequencing data. *BMC Bioinformatics*. 2013;14(1):33.

31. Liu B, Shi Y, Yuan J, et al. Estimation of genomic characteristics by analyzing k-mer frequency in de novo genome projects. *arXiv: Genomics*. 2013.

32. Marcais G and Kingsford C. A fast, lock-free approach for efficient parallel counting of occurrences of k-mers. *Bioinformatics*. 2011;27(6):764-70.

33. Chin CS, Peluso P, Sedlazeck FJ, et al. Phased diploid genome assembly with single-molecule real-time sequencing. *Nat Methods*. 2016;13(12):1050.

34. Chin CS, Alexander DH, Marks P, et al. Nonhybrid, finished microbial genome assemblies from long-read SMRT sequencing data. *Nat Methods*. 2013;10(6):563.

35. Walker BJ, Abeel T, Shea T, et al. Pilon: an integrated tool for comprehensive microbial variant detection and genome assembly improvement. *PloS One*. 2014;9(11):e112963.

36. Pryszcz LP and Gabaldón T. Redundans: an assembly pipeline for highly heterozygous genomes. *Nucleic acids Res*. 2016;44(12):e113.

37. Simão FA, Waterhouse RM, Ioannidis P, et al. BUSCO: assessing genome assembly and annotation completeness with single-copy orthologs. *Bioinformatics*. 2015;31(19):3210-2.

38. Gong GR, Dan C, Xiao SJ, et al. Chromosomal-level assembly of yellow catfish genome using third-generation DNA sequencing and Hi-C analysis. *GigaScience*. 2018;7(11):giy120.

39. Smit A, Hubley R and Green P. RepeatModeler Open-1.0. 2008–2015. http://www repeatmasker.org. Accessed 1 May, 2018.

40. Jurka J, Kapitonov VV, Pavlicek A, et al. Repbase Update, a database of eukaryotic repetitive elements. *Cytogenet Genome Res*. 2005;110(1-4):462-7.

41. Tarailo- Graovac M and Chen NS. Using RepeatMasker to identify repetitive elements in genomic sequences. *Curr Protoc Bioinform*. 2009;25(1):4.10.1- 4.10.14.

42. Benson G. Tandem repeats finder: a program to analyze DNA sequences. *Nucleic Acids Res*. 1999;27:573.

43. Stanke M, Keller O, Gunduz I, et al. AUGUSTUS: ab initio prediction of alternative transcripts. *Nucleic Acids Res*. 2006;34(suppl_2):W435-W9.

44. Lobo I. Basic local alignment search tool (BLAST). *Nat Educ*. 2008;1(1).

45. Birney E, Clamp M and Durbin RJ. GeneWise and Genomewise. *Genome Res*. 2004;14(5):988.

46. Trapnell C, Pachter L and Salzberg SL. TopHat: discovering splice junctions with RNA-Seq. *Bioinformatics*. 2009;25:1105-11.

47. Ghosh S and Chan CK. Analysis of RNA-Seq Data Using TopHat and Cufflinks. *Methods Mol Biol*. 2016;1374:339.

48. Campbell MS, Holt C, Moore B, et al. Genome Annotation and Curation Using MAKER and MAKER-P. *Curr Protoc Bioinform*; 2014;48(1): 4.11.1-4.11.39.

49. Cantarel BL, Korf I, Robb SM, et al. MAKER: an easy-to-use annotation pipeline designed for emerging model organism genomes. *Genome Res*. 2008;18(1):188-96.

50. Lowe TM and Eddy SR. tRNAscan-SE: a program for improved detection of transfer RNA genes in genomic sequence. *Nucleic Acids Res*. 1997;25(5):955-64.

51. Nawrocki EP and Eddy SR. Infernal 1.1: 100-fold faster RNA homology searches. *Bioinformatics*. 2013;29(22):2933-5.

52. Griffiths-Jones S, Bateman A, Marshall M, et al. Rfam: an RNA family database. *Nucleic Acids Res*. 2003;31(1):439-41.

53. Boeckmann B, Bairoch A, Apweiler R, et al. The SWISS-PROT protein knowledgebase and its supplement TrEMBL in 2003. *Nucleic Acids Res*. 2003;31(1):365-70.

54. McGinnis S and Madden TL. BLAST: at the core of a powerful and diverse set of sequence analysis tools. *Nucleic Acids Res*. 2004;32(suppl_2):W20-W5.

55. Harris MA, Clark J, Ireland A, et al. The Gene Ontology (GO) database and informatics resource. *Nucleic Acids Res*. 2004; 32(suppl_1): D258-D61.

56. Ogata H, Goto S, Sato K, et al. KEGG: Kyoto Encyclopedia of Genes and Genomes. *Nucleic Acids Res*. 2000; 27:29-34.

57. Conesa A, Götz S, García-Gómez JM, et al. Blast2GO: a universal tool for annotation, visualization and analysis in functional genomics research. *Bioinformatics*. 2005;21(18):3674-6.

58. Li L, Stoeckert CJ and Roos DS. OrthoMCL (OrthoMCL DB: Ortholog Groups of Protein Sequences, RRID:SCR_007839): identification of ortholog groups for eukaryotic genomes. *Genome Res*. 2003;13(9):2178-89.

59. Edgar RC. MUSCLE: multiple sequence alignment with high accuracy and high throughput. *Nucleic Acids Res*. 2004;32(5):1792-7.

60. Suyama M, Torrents D and Bork P. PAL2NAL: robust conversion of protein sequence alignments into the corresponding codon alignments. *Nucleic Acids Res*. 2006;34:609-12.

61. Castresana J. Selection of Conserved Blocks from Multiple Alignments for Their Use in Phylogenetic Analysis. *Mol Biol Evol*. 2000;17(4):540-52.

62. Stamatakis A. RAxML version 8: a tool for phylogenetic analysis and post-analysis of large phylogenies. *Bioinformatics*. 2014;30(9):1312-3.

63. Stamatakis A, Hoover P and Rougemont J. A Rapid Bootstrap Algorithm for the RAxML Web Servers. *Syst Biol*. 2008;57(5):758-71.

64. Yang Z. PAML 4: Phylogenetic Analysis by Maximum Likelihood. *Mol Biol Evol*. 2007;24(8):1586-91.

65. Grabherr M, Haas BJ, Yassour M, et al. Full-length transcriptome assembly from RNA-Seq data without a reference genome. *Nat Biotechnol*. 2011;29(7):644-52.

66. De Bie T, Cristianini N, Demuth JP, et al. CAFE: a computational tool for the study of gene family evolution. *Bioinformatics*. 2006;22(10):1269-71.

67. Alexa A and Rahnenfuhrer J. topGO: enrichment analysis for gene ontology. https://bioconductor.org/packages/topGO. Accessed 12 September 2020.

68. Xie C, Mao X, Huang J, et al. KOBAS 2.0: a web server for annotation and identification of enriched pathways and diseases. *Nucleic Acids Res*. 2011;39(suppl_2):W316-W22.

69. Talavera G and Castresana J. Improvement of Phylogenies after Removing Divergent and Ambiguously Aligned Blocks from Protein Sequence Alignments. *Syst Biol*. 2007;56(4):564-77.

70. Mckenna A, Hanna M, Banks E, et al. The Genome Analysis Toolkit: A MapReduce framework for analyzing next-generation DNA sequencing data. *Genome Res*. 2010;20(9):1297-303.

71. Lee T, Guo H, Wang X, et al. SNPhylo: a pipeline to construct a phylogenetic tree from huge SNP data. *BMC Genomics*. 2014;15(1):162.
72. Purcell S, Neale BM, Toddbrown K, et al. PLINK: A Tool Set for Whole-Genome Association and Population-Based Linkage Analyses. *Am J Hum Genet*. 2007;81(3):559-75.
73. Alexander DH, Novembre J and Lange K. Fast model-based estimation of ancestry in unrelated individuals. *Genome Res*. 2009;19(9):1655-64.
74. Tamura K, Dudley JT, Nei M, et al. MEGA4: Molecular Evolutionary Genetics Analysis (MEGA) Software Version 4.0. *Mol Biol Evol*. 2007;24(8):1596-9.
75. Liu S and Hansen MM. PSMC (pairwise sequentially Markovian coalescent) analysis of RAD (restriction site associated DNA) sequencing data. *Mol Ecol Resour*. 2017;17(4):631-41.
76. Yu M and He S. Phylogenetic relationships and estimation of divergence times among Sisoridae catfishes. *Sci China Life Sci*. 2012;55(4):312-20.
77. Huang DW, Sherman BT and Lempicki RA. Systematic and integrative analysis of large gene lists using DAVID bioinformatics resources. *Nat Protoc*. 2009;4(1):44-57.
78. Hombach S and Kretz M. Non-coding RNAs: classification, biology and functioning. Non-coding RNAs in colorectal cancer. Springer. 2016. p. 3-17.
79. Fang XM. Phased uplift of the Tibetan Plateau. *Sci Technol Rev*. 2017;6:42-50.
80. Ehlers J and Gibbard P. Quaternary Glaciation. In: Singh VP, Singh P and Haritashya UK, editors. Encyclopedia of Snow, Ice and Glaciers. Dordrecht: Springer Netherlands. 2011. p. 873-82.
81. Valdes PJ, Lin D, Farnsworth A, Spicer RA, Li S-H and Tao S. Comment on "Revised paleoaltimetry data show low Tibetan Plateau elevation during the Eocene". *Science*. 2019;365(6459):eaax8474.
82. Chang MM and Miao D. Review of the Cenozoic fossil fishes from the Tibetan Plateau and their bearings on paleoenvironment. *Chinese Sci Bull*. 2016;61(9):981-95.
83. Li J, Fang X, Song C, et al. Late Miocene–Quaternary rapid stepwise uplift of the NE Tibetan Plateau and its effects on climatic and environmental changes. *Quaternary Res*. 2014;81(3):400-23.
84. Murakami T, Terai H, Yoshiyama Y, et al. The second investigation of Lake Puma Yum Co located in the Southern Tibetan Plateau, China. *Limnology*. 2007;8(3):331-5.
85. Li S, Xia X, Zhou B, et al. Chemical balance of the Yellow River source region, the northeastern Qinghai-Tibetan Plateau: Insights about critical zone reactivity. *Appl Geochem*. 2018;90:1-12.
86. Li H, Zhang N and Lin X. Spatio-Temporal Characteristics of Yarlung Zangbo River in Tibet. *J Henan Norm Univ*. 2010;38(002):126-130.
87. Zhang N, Li H, Wen Z, et al. Spatio-Temporal Characteristics of Niyang River in Tibet. *J Henan Norm Univ*. 2009; 037(006):79-82.
88. Chen Y, Chen Y and Liu H. Studies on the position of the Qinghai-Xizang Plateau region in zoogeographic divisions and its eastern demarcation line. *Acta Hydrobiol Sinica*. 1996;20(2):97-103.
89. Van Meer G, Voelker DR and Feigenson GW. Membrane lipids: where they are and how they behave. *Nat Rev Mol Cell Biol*. 2008;9(2):112-24.
90. Hanna VS and Hafez EAA. Synopsis of arachidonic acid metabolism: A review. *J*

Adv Res. 2018;11:23-32.

91. Liu B, Zheng Y, Yin F, et al. Toll receptor-mediated Hippo signaling controls innate immunity in Drosophila. *Cell*. 2016;164(3):406-19.

92. Hong L, Li X, Zhou D, et al. Role of Hippo signaling in regulating immunity. *Cell Mol Immunol*. 2018;15(12):1003-9.

93. Tong C, Tian F and Zhao K. Genomic signature of highland adaptation in fish: a case study in Tibetan Schizothoracinae species. *BMC genomics*. 2017;18(1):1-9.

94. Tong C, Fei T, Zhang C, et al. Comprehensive transcriptomic analysis of Tibetan Schizothoracinae fish *Gymnocypris przewalskii* reveals how it adapts to a high altitude aquatic life. *BMC Evol Biol*. 2017;17(1):1-11.

95. Macfadyen EJ, Williamson CE, Grad G, et al. Molecular response to climate change: temperature dependence of UV- induced DNA damage and repair in the freshwater crustacean *Daphnia pulicaria*. *Global Change Biol*. 2004;10(4):408-16.

96. Ensminger M and Lobrich M. One end to rule them all: Non-homologous end-joining and homologous recombination at DNA double-strand breaks. *Brit J Radiol*. 2020; 93: 20191054.

97. Kim H and Dandrea AD. Regulation of DNA cross-link repair by the Fanconi anemia/BRCA pathway. *Gene Dev*. 2012;26(13):1393-408.

98. Yu L, Wang G, Ruan J, et al. Genomic analysis of snub-nosed monkeys (*Rhinopithecus*) identifies genes and processes related to high-altitude adaptation. *Nat Genet*. 2016;48(8):947-52.

99. Rousselle M, Mollion M, Nabholz B, et al. Overestimation of the adaptive substitution rate in fluctuating populations. *Biol Letters*. 2018;14(5):20180055.

100. Chongqing Water Resources Bureau: Daning River http://www.cqwater.gov.cn/swgg/hkgk/Pages/2017/08/20170807165431.aspx Accessed 7 August 2017.

101. Yuan D, Chen X, Gu H, Zou M, Zou Y, Fang J, Tao W et al. Supporting data for "Chromosomal genome of Triplophysa bleekeri provides insights into its evolution and environmental adaptation" GigaScience Database 2020. http://dx.doi.org/10.5524/100823

Figure legends

Figure 1. Morphology and geographic distribution of *T. bleekeri*. (a) *T. bleekeri* used in this study. (b) Geographic distribution of the sampling locations for *T. bleekeri*. The red circles, green triangle, yellow trapezoid, and dotted ellipse represent the sampling sites, gorge, artificial dam, and Wuxi Town, respectively.

Figure 2. Phylogenetic relationships and divergence time estimation for *T. bleekeri* and other fish species. All nodes were completed and supported by 100 cycles of bootstrap resampling. Numbers near the nodes (shown in blue) indicate the estimated

divergence times with a 95% confidence interval. Divergences used for the recalibration of time estimation are indicated with red dots.

Figure 3. Natural positively selected gene (PSG) identification and functional analysis for *T. bleekeri*, *T. tibetana*, and *T. siluroides*. (a) Venn diagram for PSGs for the three fish species. (b) Venn diagram for PSs identified from species- and lineage-based method. (c) Enrichment analysis on the biological pathways for candidate PSGs identified from the species- and lineage-based method.

Figure 4. Historical effective population size profile deduced from the whole-genome sequencing data. One hundred bootstrap replicates were performed for the effective population size estimation.

Figure 5. The population genetics analysis for *T. bleekeri*. (a) Neighbor-joining phylogenetic tree of individuals based on whole-genome SNP loci. Note that samples from population LHK, XX, and BY are labeled with red, green and blue, respectively. (b) Principal component (PC) analysis plots of the first two components. The fraction of the variance obtained was 14.5% for PC1 and 6.4% for PC2. The samples from population LHK, XX and BY are represented by red, green and blue color, respectively. (c) Population structure plots of *T. bleekeri*. The samples from population LHK, XX and BY are represented by red, green and blue color, respectively. We assume that there were three populations for the analysis (K=3). The y axis quantifies the proportion of the individual's genome from inferred ancestral populations, and x axis shows the different populations.

Figure 6. Selective sweep analysis to identify candidate selected functional genes among populations. (a) Manhattan plot to show the whole-wide genomic differentiation between LHK and BY populations. (b) The venn plot for shared enriched biological pathway for candidate selected functional genes from the selective sweep analysis among population comparisons. (c) The shared enriched biological pathway from LHK-BY and XX-BY comparisons. (d) The Fst profiles for genomic regions containing *treh*, *ctnnb1* and *lef1* gene. Note that color scheme for population comparison is identical for (a), (b) and (c).

 Click here to access/download;Table;Table 1.docx

Table 1. A summary of sequencing data used in genome assembly and gene annotation.

Source	Platform	Clean Data (Gb)	Mean Read Length (bp)	Sequence Coverage (X)
genome	Illumina HiSeq X Ten	81.7	150	129
genome	PacBio SEQUEL	100.87	5,827	160
genome (Hi-C)	Illumina HiSeq X Ten	83.5	150	132
transcriptome	Illumina HiSeq X Ten	11.1	150	-

 Click here to access/download;Table;Table 2.docx

Table 2. The length statistics for contig assembly for the *T. bleekeri* genome

	Assemble	Total Length (bp)	Sequence Number	Contig N50 (Mb)	Scaffold N50 (Mb)
contig assembly using long-read data	Falcon	657,392,105	1,357	3.31	3.31
	Arrow	660,275,268	1,357	3.33	3.33
	Pilon	659,964,583	1,357	3.33	3.33
	Redundans	628,132,429	856	3.82	3.82
chromosome assembly using Hi-C data	all sequences	620,272,795	181	3.11	22.89
	chromosomes	596,964,218	25	3.23	23.21
	unanchored sequences	23,308,577	156	0.17	1.01

Figure 1 Click here to access/download;Figure;Figure 1.docx

Figure 1. Morphology and geographic distribution of *T. bleekeri*

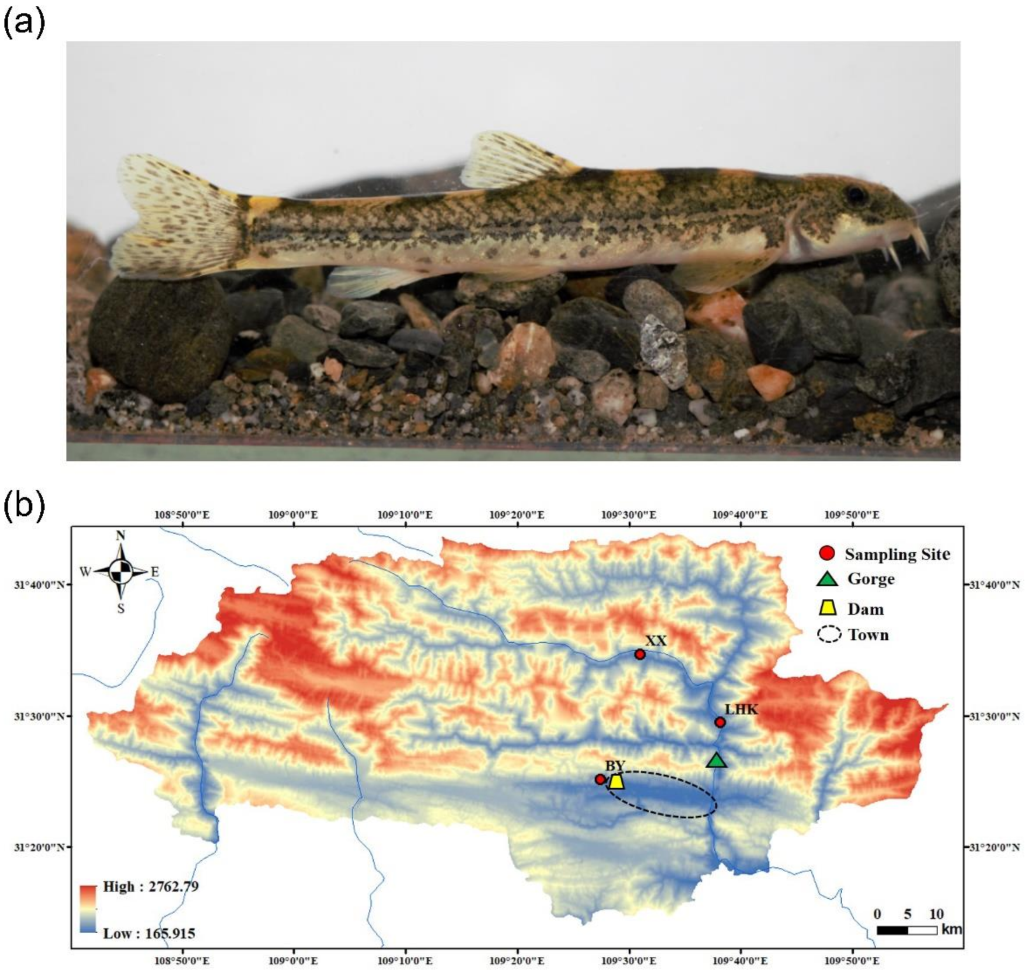

Click here to access/download;Figure;Figure 2.docx

Figure 2. Phylogenetic relationships and divergence time estimation for *T. bleekeri* and other fish species.

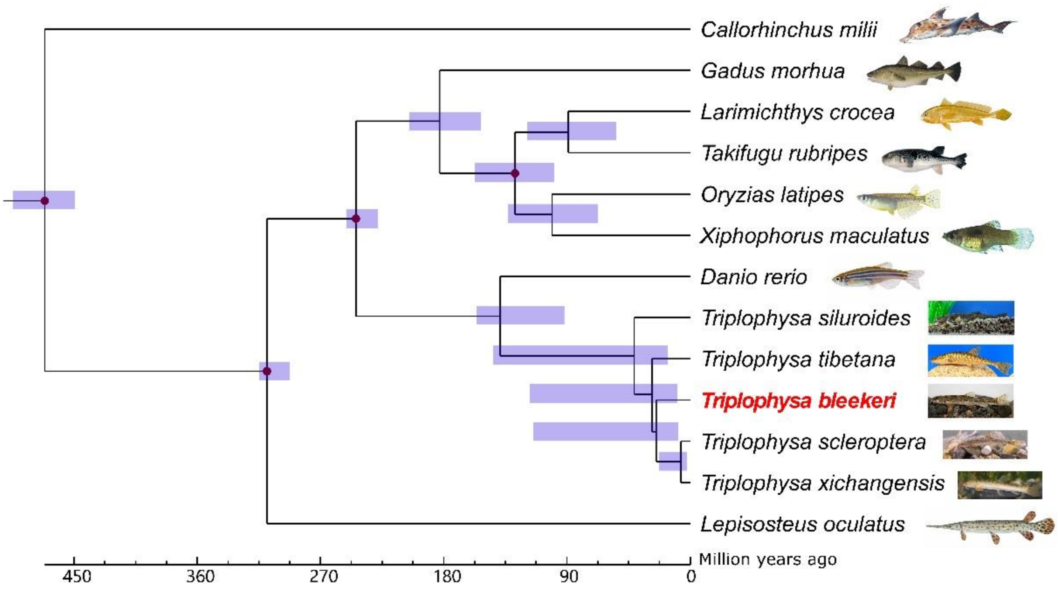

 Click here to access/download;Figure;Figure 3.docx

Figure 3. Natural positively selected gene (PSG) identification and functional analysis for *T. bleekeri*, *T. tibetana*, and *T. siluroides*.

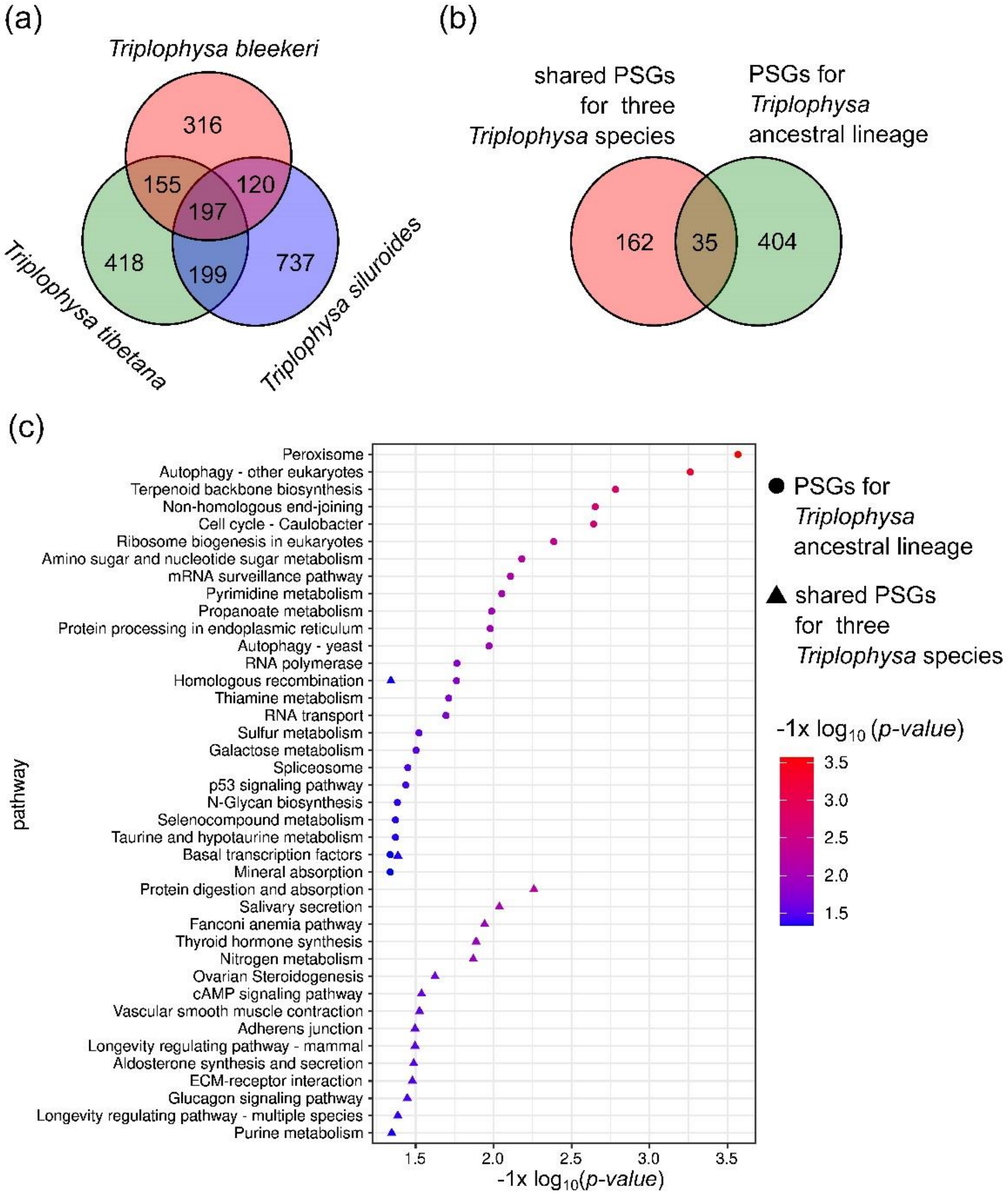

Figure 4 Click here to access/download;Figure;Figure 4.docx

Figure 4. Historical effective population size profile deduced from the whole-genome sequencing data.

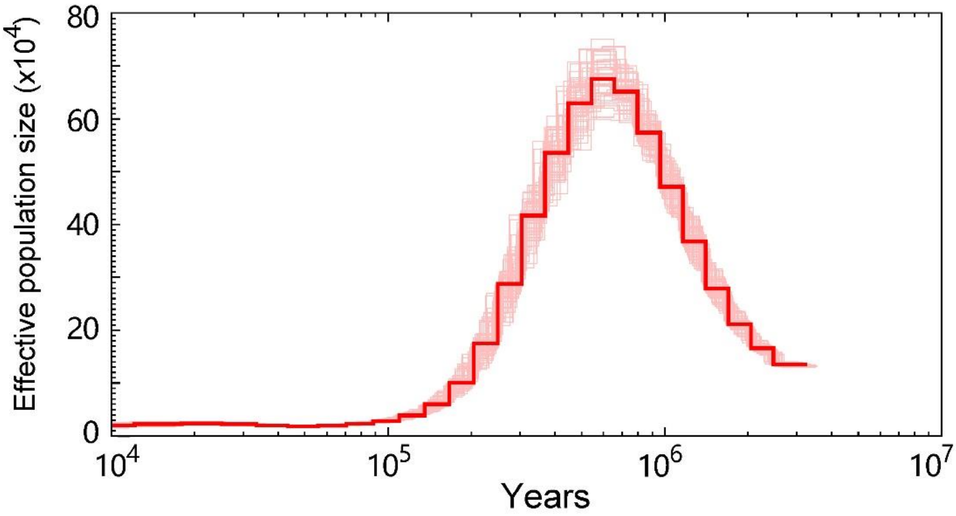

 Click here to access/download;Figure;Figure 5.docx

Figure 5. The population genetics analysis for *T. bleekeri*.

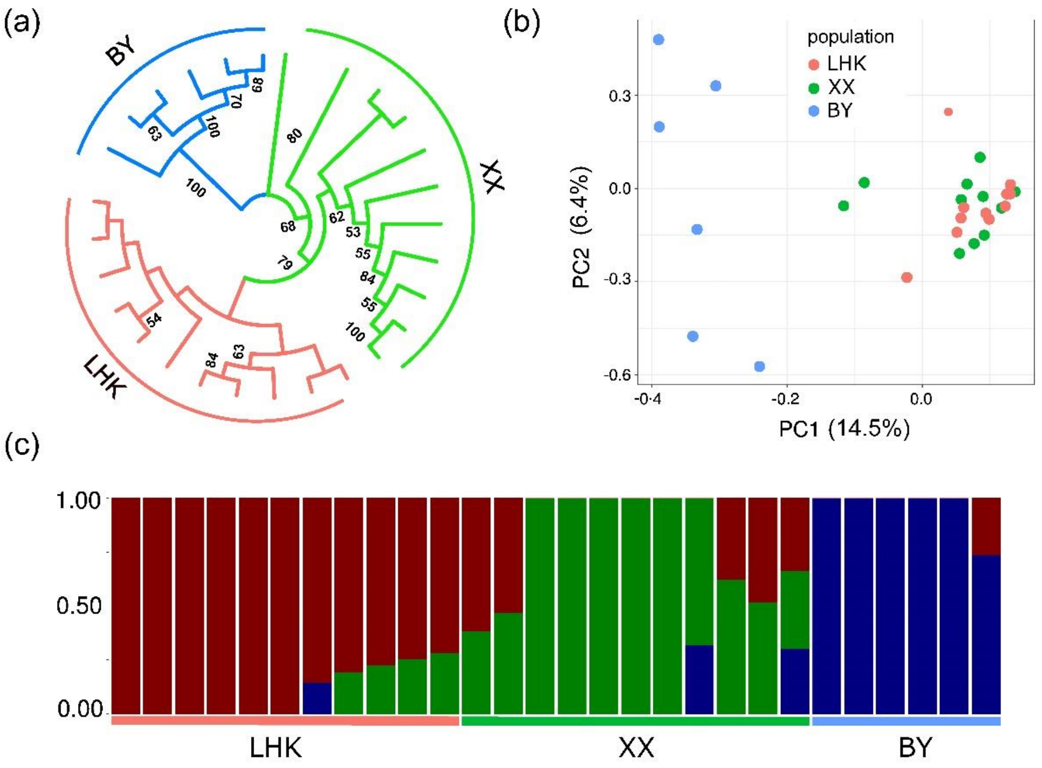

Figure 6 Click here to access/download;Figure;Figure 6.docx

Figure 6. Selective sweep analysis to identify candidate selected functional genes among populations.

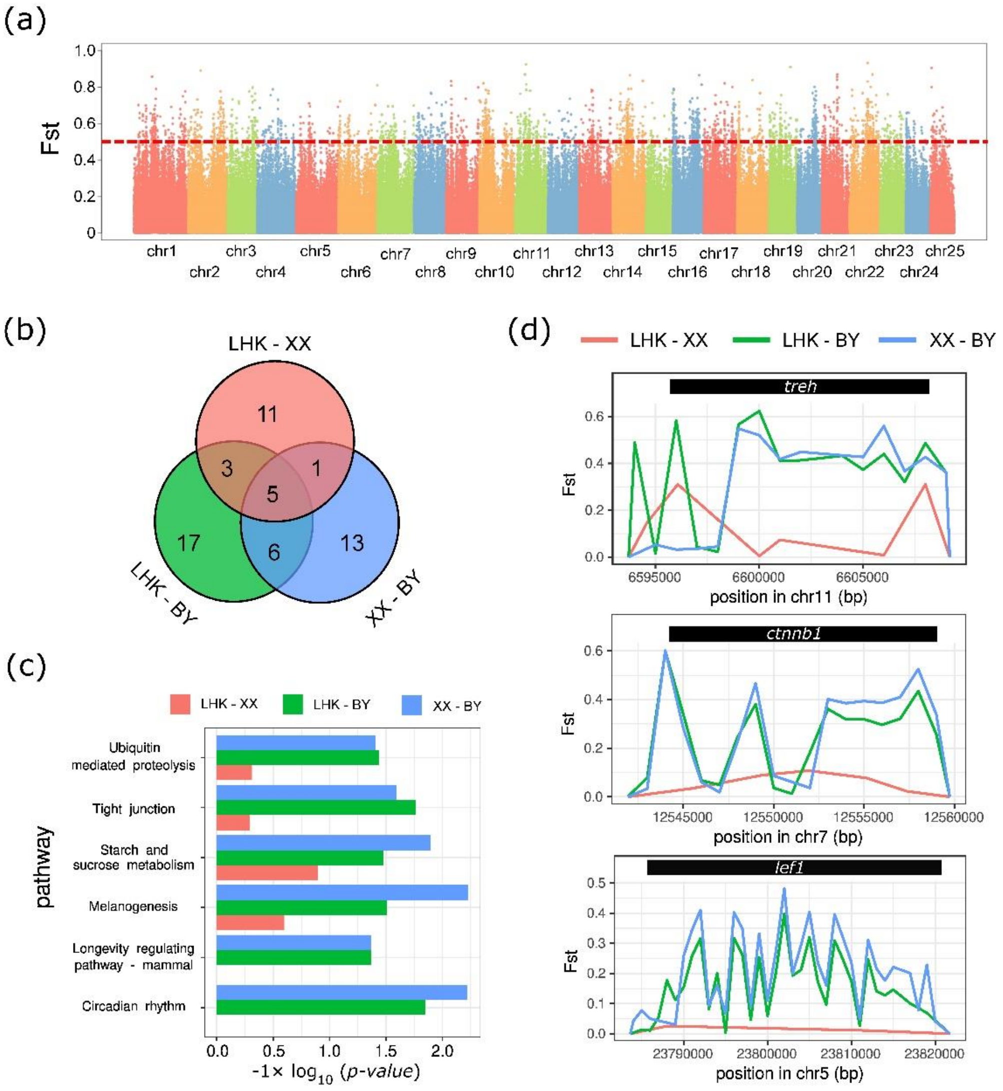

Click here to access/download
Supplementary Material
Additional file 1.docx

Supplementary Material 2

Click here to access/download

Supplementary Material

Additional file 2-Supplementary Table 4.xls

Click here to access/download
Supplementary Material
Additional file 3-Supplementary Table 5.xls

Click here to access/download
Supplementary Material
Additional file 4-Supplementary Table 11.xlsx

Click here to access/download
Supplementary Material
Additional file 5-Supplementary Table 12.xlsx

Click here to access/download
Supplementary Material
Additional file 6-Supplementary Table 13.xlsx

Personal Cover Click here to access/download;Personal Cover;Cover letter-Triplophysa bleekeri.docx

Dear Editor,

Attached is our recent manuscript entitled "**Chromosomal genome assembly of *Triplophysa bleekeri* provides insights into its evolution and environmental adaptation on the Qinghai-Tibetan Plateau**." We would like to send for your consideration to publish as a resource article in ***GigaScience***.

The continuous uplifts and climatic changes during the formation of Qinghai-Tibetan Plateau (QTP) posed profound influences on the evolution of endemic organisms. Fish species on the QTP are more susceptible to tectonic movements and temperature fluctuations since it strictly constrained by water ecology and drainage connectivity in habitats. Living in extreme environments, including low temperature and high UV exposure, highland endemic fishes have been subjected to severe natural selections; however, few studies were performed to investigate the molecular mechanism of the adaptation formation for fish species on the QTP. The genome and population resources of endemic fish species play an essential role in adaptive evolution studies. Although several genomes of fish species on the QTP, including *Glyptosternon maculatum, Oxygymnocypris stewartii*, *Triplophysa tibetana*, and *Triplophysa siluroides*, have been reported, the genetic resource, especially for whole-genome population data, is still insufficient, largely hindered the evolution and conservation genetics studies of endemic fish.

Triplophysa bleekeri (*T. bleekeri*), a typical fish species occurring at an elevation from 200 m to 3,000 m, provides us an excellent model to investigate the adaption mechanism and population genetics for fish on the QTP. In this study, we assembled a chromosome genome for *T. bleekeri* using Illumina, PacBio sequencing platform, and Hi-C technique. Based on more than 160 X coverage of long sequencing data, we generated a 628 Mb *T. bleekeri* genome with a contig N50 length of 3.82 Mb. Using interaction frequencies among contigs from the Hi-C technique, a chromosome genome was assembled with a scaffold N50 length of 22.9 Mb, and more than 96.2% of the genome on the base level was successfully anchored upon 25 chromosomes. A

total of 21,198 protein-coding genes were predicted in the *T. bleekeri* genome, of which 97.3% of the protein-coding genes were functionally annotated.

We explored the environmental adaptation of *T. bleekeri* from the perspective of functional genes in the genome. We found that gene families related to lipid metabolism, necroptosis, and immune response were significantly expanded in the *T. bleekeri* genome, comparing to those non-highland fish species. Genes involved in DNA repair and protein digestion underwent strong natural positive selections for *T. bleekeri*, *T. siluroides* and *T. tibetana*. Our result implied that *T. bleekeri* might under severe stress with the cold environment, and *Triplophysa* species might be under similar natural selections. We also performed whole-genome resequencing for 28 samples from three populations in the Daning River. We illuminated the difference between their genetic structures, which can be explained by the relatively limited gene flow hampered by natural gorges and artificial barriers, such as cities and dams, among those populations.

Our work provided important reference genome and population variation resource and preliminary investigation of the environmental adaptation and population structures of *T. bleekeri*. Those data will not only be used for further ecological and conservation studies for the species but also offered valuable information for the evolutionary researches based on the comparative analysis among endemic fish and vertebrates.

All authors have read and approved this version of the article. No part of this paper has been published or submitted elsewhere. No conflict of interest exists in the submission of this manuscript.

We are looking forward to receiving a favorable response from you regarding the acceptance of the manuscript. Thank you for your help.

Sincerely Yours,

Prof. Zhijian Wang
School of Life Sciences, Southwest University

Beibei, Chongqing, PR China 400715
Tel: 86-023-68253005
Fax: 86-023-68253005
Email: wangzj1969@126.com
