# Supplementary material for: Chromosomal genome of Triplophysa bleekeri provides insights into its evolution and environmental adaptation
Source: Gigascience. 2020 Nov 24;9(11):giaa132. doi: 10.1093/gigascience/giaa132 (PMC7684707; doi:10.1093/gigascience/giaa132)
Supplement: giaa132_Supplemental_Files [file giaa132_supplemental_files.zip › Additional file 1.docx]

**Supplementary Information for**

**Chromosomal genome of *Triplophysa bleekeri* provides insights into its evolution and environmental adaptation**

Dengyue Yuan^1^, Xuehui Chen^1^, Haoran Gu^1^, Ming Zou^2^, Yu Zou^2^, Jian Fang^2^, Wenjing Tao^1^, Xiangyan Dai^1^, Shijun Xiao^2,*^, Zhijian Wang^1,*^

^1^ Key Laboratory of Freshwater Fish Reproduction and Development (Ministry of Education), Key Laboratory of Aquatic Science of Chongqing, School of Life Sciences, Southwest University, Chongqing 400715, China

^2^ School of Computer Science and Technology, Wuhan University of Technology, Wuhan, Hubei 430000, China

* Correspondence to: Prof. Dr. Zhijian Wang (wangzj1969@126.com) and Dr. Shijun Xiao (shijun_xiao@163.com)

**Supplementary Tables**

**Supplementary Table S1. The repetitive element annotation for the *T. bleekeri* genome.**

|  | **repBase Tes** | | **TE Proteins** | | **De novo** | | **Combined** | |
| --- | --- | --- | --- | --- | --- | --- | --- | --- |
|  | **Length**  **(bp)** | **Percent (%)** | **Length**  **(bp)** | **Percent**  **(%)** | **Length**  **(bp)** | **Percent (%)** | **Length**  **(bp)** | **Percent (%)** |
| DNA | 66,051,674 | 10.52 | 5,775,230 | 0.92 | 59,523,179 | 9.48 | 107,824,571 | 17.17 |
| LINE | 19,348,006 | 3.08 | 14,193,095 | 2.26 | 24,252,179 | 3.86 | 36,404,601 | 5.8 |
| SINE | 2,526,055 | 0.4 | 0 | 0 | 1,914,570 | 0.3 | 4,274,901 | 0.68 |
| LTR | 18,462,738 | 2.94 | 10,755,386 | 1.71 | 33,739,320 | 5.37 | 43,514,459 | 6.93 |
| Satellite | 1,274,550 | 0.2 | 0 | 0 | 1,308,975 | 0.21 | 2,510,477 | 0.4 |
| Simple_repeat | 995,026 | 0.16 | 0 | 0 | 1,478,768 | 0.24 | 2,415,315 | 0.38 |
| Other | 325 | 0 | 0 | 0 | 0 | 0 | 325 | 0 |
| Unknown | 778,801 | 0.12 | 0 | 0 | 4,627,852 | 0.74 | 5,371,935 | 0.86 |
| Total | 106,947,343 | 17.03 | 30,706,305 | 4.89 | 135,942,261 | 21.64 | 203,273,127 | 32.36 |

**Supplementary Table S2. The protein-coding gene annotation in the *T. bleekeri* genome**

| **Gene set** | | **Protein**  **coding**  **gene**  **number** | **Average gene length (bp)** | **Average CDS length (bp)** | **Average exon per gene** | **Average exon length (bp)** | **Average intron length (bp)** |
| --- | --- | --- | --- | --- | --- | --- | --- |
| *De novo* | AUGUSTUS | 20,274 | 14781.7 | 1723.0 | 10.1 | 170.0 | 1429.8 |
| Homolog | Genscan | 27,243 | 16189.9 | 1617.1 | 8.6 | 187.4 | 1909.9 |
|  | *C. carpio* | 45,358 | 6563.3 | 973.8 | 5.1 | 191.2 | 1365.3 |
|  | *D. rerio* | 36,100 | 7449.4 | 1002.5 | 5.1 | 196.6 | 1572.6 |
|  | *O. latipes* | 36,890 | 7390.9 | 933.9 | 5.2 | 178.9 | 1530.4 |
|  | *T. nigroviridis* | 31,809 | 7576.6 | 987.3 | 5.9 | 168.8 | 1358.8 |
|  | *X. maculatus* | 33,971 | 7611.8 | 1003.2 | 5.8 | 172.3 | 1370.9 |
| RNA seq | | 15,875 | 14522.5 | 1548.6 | 10.4 | 294.7 | 1226.8 |
| MAKER | | 21,198 | 14454.4 | 1685.2 | 10.3 | 248.2 | 1283.9 |

**Supplementary Table S3. The non-coding genes predicted in the genome.**

| **Type** | | **Number** | **Average Length(bp)** | **Total Length(bp)** |
| --- | --- | --- | --- | --- |
| miRNA | | 335 | 83.91 | 28,112 |
| tRNA | | 8,406 | 74.41 | 625,491 |
| rRNA | rRNA | 292 | 154.18 | 45,022 |
|  | 18S | 11 | 1,262.72 | 13,890 |
|  | 28S | 0 | 0 | 0 |
|  | 5.8S | 8 | 143.5 | 1,148 |
|  | 5S | 273 | 109.83 | 29,984 |
| snRNA | snRNA | 480 | 169.17 | 81,204 |
|  | CD-box | 301 | 186.34 | 56,091 |
|  | HACA-box | 26 | 161.84 | 4,208 |
|  | splicing | 147 | 133.87 | 19,679 |
|  | scaRNA | 4 | 279.00 | 1,116 |

**Supplementary Table S4. GO pathway enrichment analyses for natural positively selected genes [see Additional file 2].**

**Supplementary Table S5. KEGG enrichment analyses for natural positively selected genes [see Additional file 3].**

**Supplementary Table S6. KEGG pathway enrichment analyses for the members of gene families subject to expansions during the evolution of the genome.**

| **Pathway** | **Gene Number** | **Background Gene Number** | **Rich Factor** | **Qvalue** |
| --- | --- | --- | --- | --- |
| Indole alkaloid biosynthesis | 1 | 1 | 1 | 0.00E+00 |
| Necroptosis | 54 | 149 | 0.3624 | 7.10E-09 |
| Hippo signaling pathway | 45 | 168 | 0.2679 | 2.40E-03 |
| RNA transport | 40 | 145 | 0.2759 | 2.40E-03 |
| Signaling pathways regulating pluripotency of stem cells | 38 | 143 | 0.2657 | 7.00E-03 |
| Glycosphingolipid biosynthesis - globo and isoglobo series | 5 | 8 | 0.625 | 8.90E-03 |
| Vitamin B6 metabolism | 5 | 8 | 0.625 | 8.90E-03 |
| Glycosaminoglycan biosynthesis - keratan sulfate | 7 | 14 | 0.5 | 1.10E-02 |
| Notch signaling pathway | 17 | 55 | 0.3091 | 2.00E-02 |
| RNA degradation | 23 | 82 | 0.2805 | 2.00E-02 |
| Arachidonic acid metabolism | 14 | 42 | 0.3333 | 2.00E-02 |
| Sulfur relay system | 4 | 7 | 0.5714 | 2.40E-02 |
| Caprolactam degradation | 4 | 7 | 0.5714 | 2.40E-02 |
| Cell cycle - Caulobacter | 2 | 3 | 0.6667 | 5.90E-02 |
| Other glycan degradation | 7 | 19 | 0.3684 | 7.00E-02 |

**Supplementary Table S7. GO enrichment analyses for the members of gene families subject to expansions during the evolution of the genome.**

| **GO Term** | **Gene Number** | **Background Gene Number** | **Rich Factor** | **Qvalue** |
| --- | --- | --- | --- | --- |
| nucleosome | 151 | 215 | 0.7023 | 1.20E-67 |
| protein-DNA complex | 151 | 217 | 0.6959 | 9.60E-67 |
| DNA packaging complex | 151 | 217 | 0.6959 | 9.60E-67 |
| chromatin | 154 | 231 | 0.6667 | 5.80E-64 |
| chromosomal part | 154 | 268 | 0.5746 | 3.70E-51 |
| chromosome | 155 | 284 | 0.5458 | 1.80E-47 |
| nucleic acid binding | 623 | 2,460 | 0.2533 | 8.60E-30 |
| heterocyclic compound binding | 872 | 4,112 | 0.2121 | 1.10E-15 |
| non-membrane-bounded organelle | 221 | 774 | 0.2855 | 1.20E-15 |
| intracellular non-membrane-bounded organelle | 221 | 774 | 0.2855 | 1.20E-15 |
| organic cyclic compound binding | 872 | 4,117 | 0.2118 | 1.60E-15 |
| DNA binding | 304 | 1,191 | 0.2552 | 5.10E-14 |
| nucleosome assembly | 30 | 44 | 0.6818 | 7.90E-14 |
| chromatin assembly | 30 | 44 | 0.6818 | 7.90E-14 |
| nucleosome organization | 30 | 45 | 0.6667 | 2.10E-13 |
| protein-DNA complex assembly | 34 | 57 | 0.5965 | 8.40E-13 |
| chromatin assembly or disassembly | 30 | 47 | 0.6383 | 1.30E-12 |
| protein-DNA complex subunit organization | 34 | 58 | 0.5862 | 1.80E-12 |
| olfactory receptor activity | 33 | 56 | 0.5893 | 3.00E-12 |
| DNA packaging | 30 | 48 | 0.625 | 3.00E-12 |

**Supplementary Table S8. KEGG pathway enrichment analyses for the members of gene families subject to contractions during the evolution of the genome.**

| **Pathway** | **Gene Number** | **Background Gene Number** | **Rich Factor** | **Qvalue** |
| --- | --- | --- | --- | --- |
| GABAergic synapse | 9 | 102 | 0.0882 | 2.30E-07 |
| Cardiac muscle contraction | 7 | 69 | 0.1014 | 1.30E-06 |
| Retrograde endocannabinoid signaling | 9 | 150 | 0.06 | 2.90E-06 |
| Serotonergic synapse | 8 | 116 | 0.069 | 2.90E-06 |
| Tight junction | 9 | 168 | 0.0536 | 5.50E-06 |
| Cholinergic synapse | 8 | 140 | 0.0571 | 9.60E-06 |
| Aldosterone synthesis and secretion | 6 | 82 | 0.0732 | 2.30E-05 |
| Adrenergic signaling in  cardiomyocytes | 8 | 165 | 0.0485 | 2.80E-05 |
| Carbohydrate digestion and  absorption | 4 | 34 | 0.1176 | 3.60E-05 |
| GnRH signaling pathway | 6 | 93 | 0.0645 | 3.70E-05 |
| Calcium signaling pathway | 9 | 233 | 0.0386 | 4.90E-05 |
| Glutamatergic synapse | 7 | 141 | 0.0496 | 5.20E-05 |
| Insulin secretion | 6 | 103 | 0.0583 | 5.60E-05 |
| MAPK signaling pathway | 9 | 250 | 0.036 | 7.10E-05 |
| Dopaminergic synapse | 7 | 157 | 0.0446 | 9.20E-05 |

**Supplementary Table S9. GO enrichment analyses for the members of gene families subject to contractions during the evolution of the genome.**

| **GO Term** | **Gene Number** | **Background Gene Number** | **Rich Factor** | **Qvalue** |
| --- | --- | --- | --- | --- |
| DNA alkylation | 1 | 1 | 1 | 0.00E+00 |
| DNA methylation | 1 | 1 | 1 | 0.00E+00 |
| mucus layer | 1 | 1 | 1 | 0.00E+00 |
| gamma-aminobutyric acid:sodium  symporter activity | 2 | 2 | 1 | 0.00E+00 |
| taurine transmembrane transporter  activity | 3 | 3 | 1 | 0.00E+00 |
| taurine:sodium symporter activity | 3 | 3 | 1 | 0.00E+00 |
| DNA-methyltransferase activity | 1 | 1 | 1 | 0.00E+00 |
| gamma-aminobutyric acid transmembrane transporter activity | 2 | 2 | 1 | 0.00E+00 |
| glycine binding | 2 | 2 | 1 | 0.00E+00 |
| xenobiotic transporter activity | 3 | 3 | 1 | 0.00E+00 |
| alkanesulfonate transporter activity | 3 | 3 | 1 | 0.00E+00 |
| myosin complex | 17 | 56 | 0.3036 | 5.30E-24 |
| actin cytoskeleton | 18 | 102 | 0.1765 | 2.20E-20 |
| transmembrane transporter activity | 36 | 751 | 0.0479 | 7.60E-20 |
| ion transmembrane transporter  activity | 32 | 587 | 0.0545 | 3.10E-19 |
| substrate-specific transmembrane  transporter activity | 32 | 602 | 0.0532 | 6.30E-19 |
| neurotransmitter transporter  activity | 12 | 34 | 0.3529 | 1.70E-18 |
| neurotransmitter:sodium symporter  activity | 12 | 34 | 0.3529 | 1.70E-18 |
| transporter activity | 36 | 862 | 0.0418 | 6.00E-18 |
| solute:sodium symporter activity | 12 | 39 | 0.3077 | 1.30E-17 |

**Supplementary Table S10. Fst calculation for populations**

| **Comparison** | **Mean Fst value** | **Max Fst value** | **1% Fst** | **5% Fst** |
| --- | --- | --- | --- | --- |
| LHK-XX | 0.1037 | 0.9320 | 0.4816 | 0.3253 |
| LHK-BY | 0.1012 | 0.9341 | 0.4595 | 0.3101 |
| XX-BY | 0.0977 | 0.819 | 0.3935 | 0.2667 |

**Supplementary Table S11. KEGG pathway enrichment analyses for candidate selected genes by comparing LHK and XX populations [see Additional file 4].**

**Supplementary Table S12. KEGG pathway enrichment analyses for candidate selected genes by comparing LHK and BY populations [see Additional file 5].**

**Supplementary Table S13. KEGG pathway enrichment analyses for candidate selected genes by comparing XX and BY populations [see Additional file 6].**

**Supplementary Figures**

**
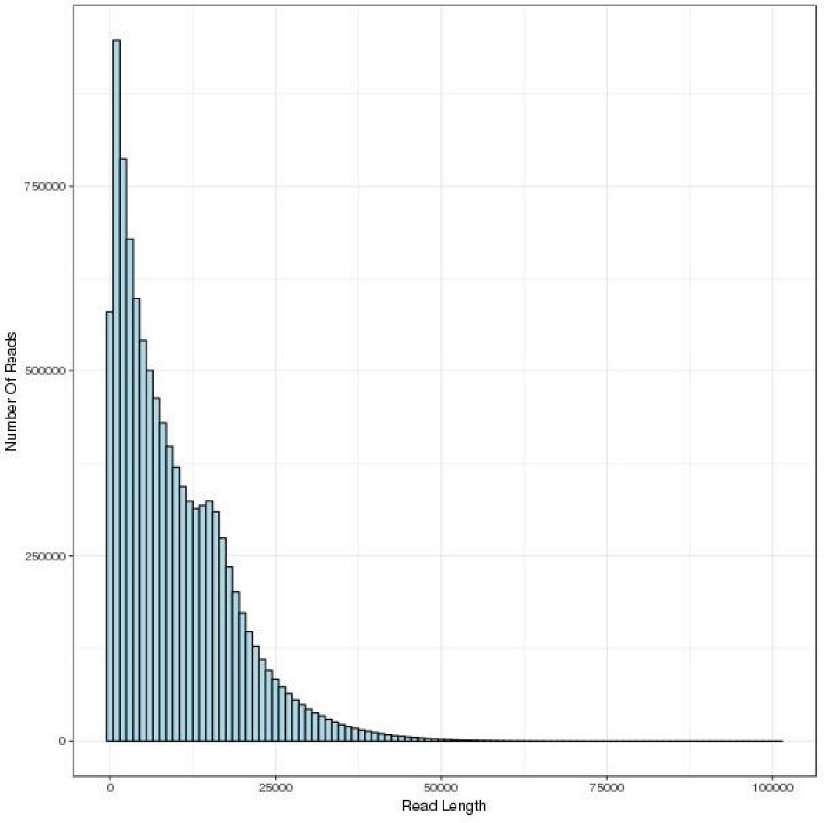
**

**Supplementary Figure S1. Read length distribution for PacBio long read sequencing.**

**
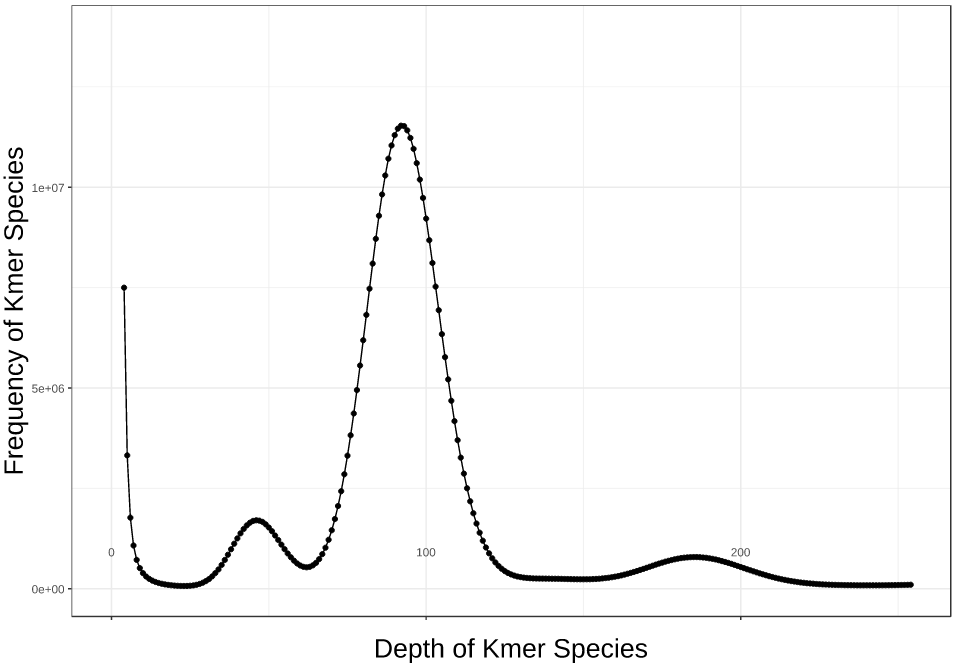
**

**Supplementary Figure S2. *K*mer frequency distribution from NGS short-read sequencing data.**


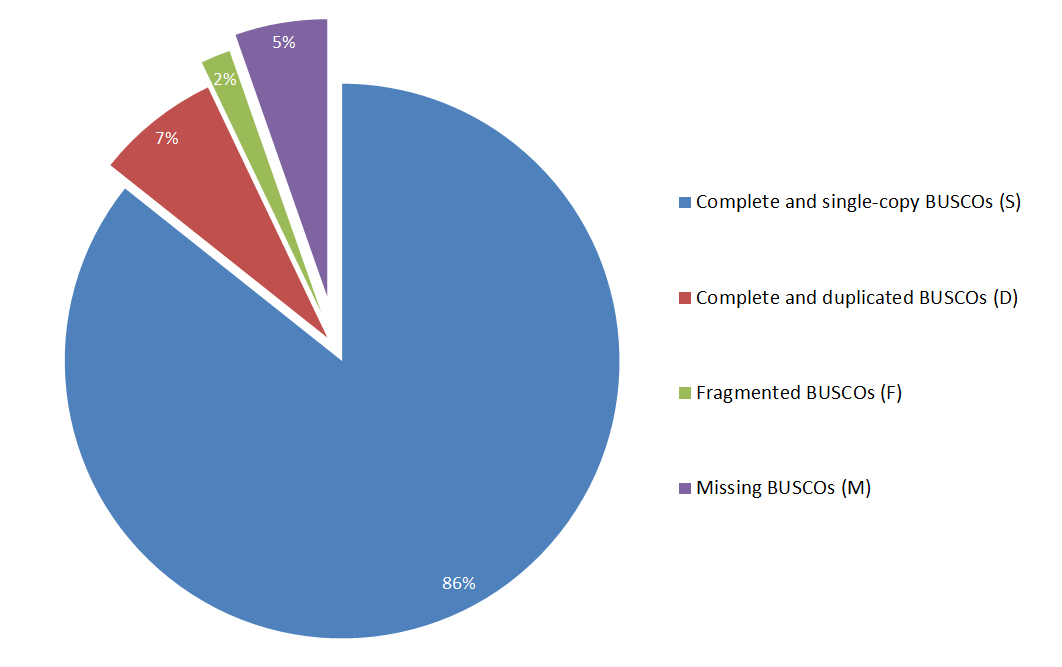


**Supplementary Figure S3. The identification of BUSCO genes in the assembled genome**

**
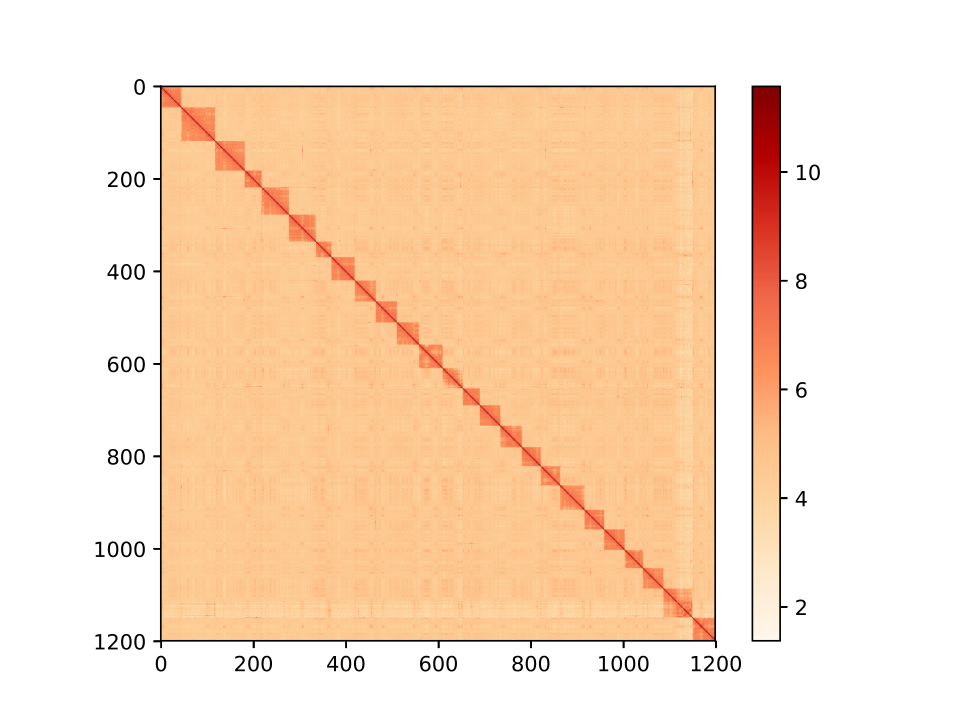
**

**Supplementary Figure S4. The interaction frequency among contigs for chromosome assembly.** The red blocks along diagonal illuminated the intra-chromosome interactions. The color scheme showed the logarithm of the contact density from red (high) to white (low) in the plot.


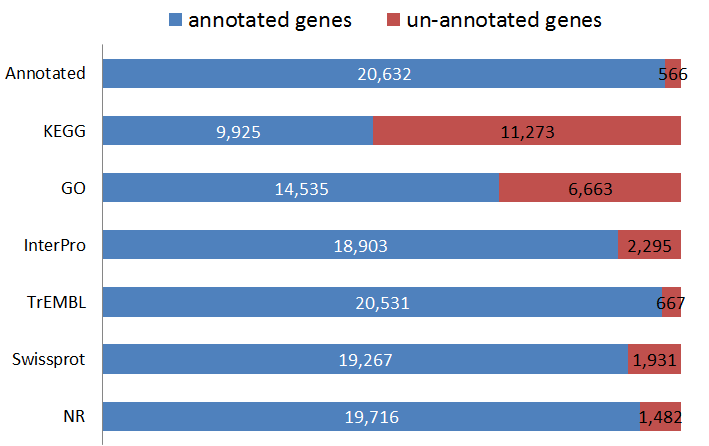


**Supplementary Figure S5.** **Functional annotations for predicted protein-coding genes for *T. bleekeri*.** Note that a gene found at least one hit in public databases, we call the gene was annotated.


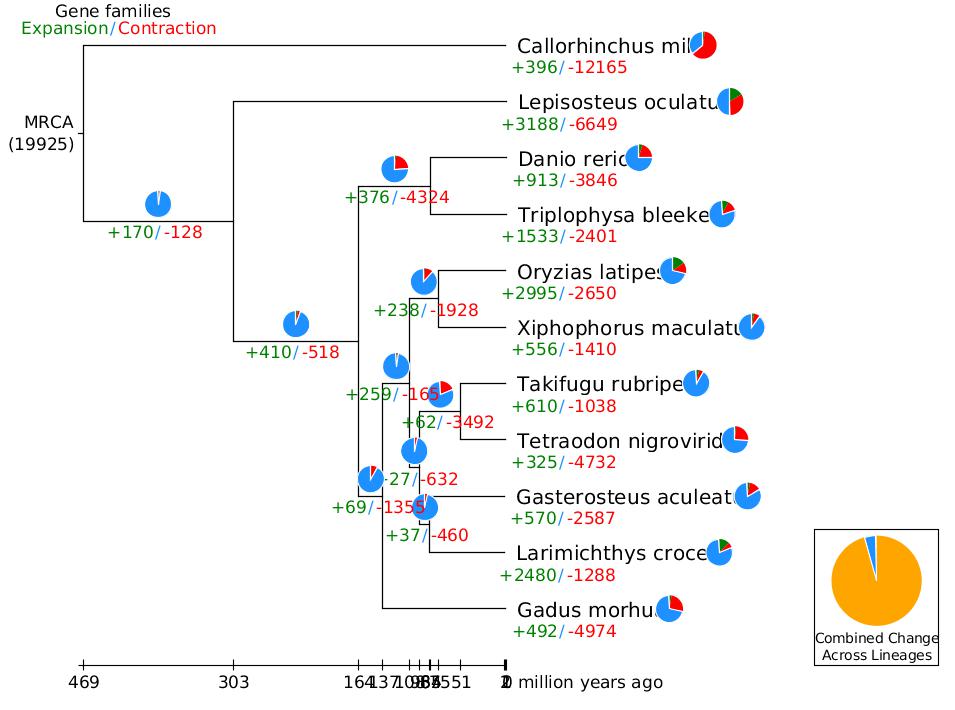


**Supplementary Figure S6. The number of expanded and contracted gene families deduced using cafe for each branch.**


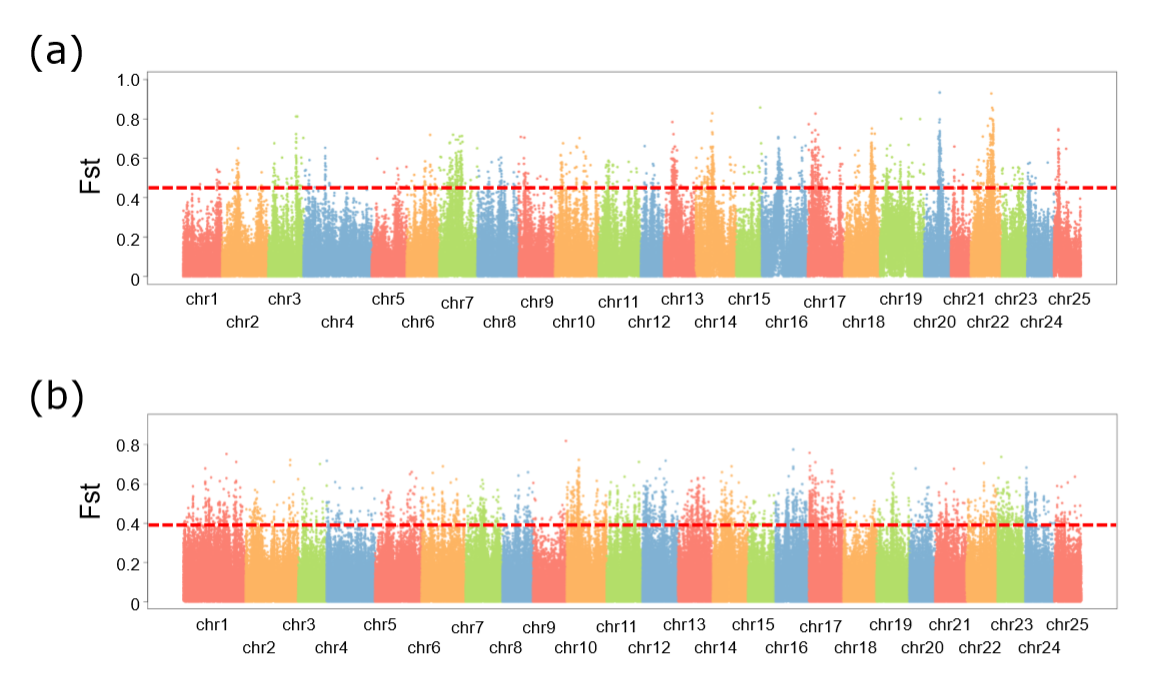


**Supplementary Figure S7. Manhattan plot showing the whole-wide genomic differentiation for LHK-XX (a) and XX-BY (b) comparisons.** The dashed red line showed the top 1% Fst threshold value.
