# Supplementary material for: Chromosomal genome of Triplophysa bleekeri provides insights into its evolution and environmental adaptation
Source: Gigascience. 2020 Nov 24;9(11):giaa132. doi: 10.1093/gigascience/giaa132 (PMC7684707; doi:10.1093/gigascience/giaa132)
Supplement: giaa132_Response_to_Reviewer_Comments_Original_Submission [file giaa132_response_to_reviewer_comments_original_submission.pdf]

Author's Response To Reviewer Comments

Close

Manuscript number: GIGA-D-20-00124
Article Type: DataNote
Title: Chromosomal genome of Triplophysa bleekeri provides insights into its evolution and environmental adaptation
Correspondence Author: Shijun Xiao; Zhijian Wang

Dear editor,
Thank you very much for your supervision in the reviewing process of my manuscript. We also highly appreciate the reviewer's carefulness, conscientiousness, and broad knowledge of the relevant research fields since they have given us many beneficial suggestions. Based on these comments and suggestions, we have made careful modifications to the original manuscript. Besides, the language was improved by Wiley Editing Services. The Enclosed herein, please find the revised manuscript entitled "Chromosomal genome of Triplophysa bleekeri provides insights into its evolution and environmental adaptation" (MS: GIGA-D-20-00124), and the modified parts of the manuscript were highlighted by using the track changes mode in MS Word. We sincerely hope this manuscript will be finally acceptable to be published on GigaScience. Thank you very much for all your help, and looking forward to hearing from you soon.
Best regards.
Sincerely Yours,
Dr. Zhijian Wang

Detailed responses to Reviewers
Reviewer #1
Major Comment:
The manuscript by Ding et al describes the chromosome-level assembly of an endemic highland fish Triplophysa bleekeri. The authors used the combined technology of the Illumina, PacBio, and Hi-C platforms and both DNA and RNA sequencing from 12 tissues for generating 628 Mb highly continuous genome assembly with the scaffold and contig N50 values reaching 22.9 Mb and 3.1 Mb, respectively. More than 596.9 Mb sequences were anchored upon 25 chromosomes. The authors identified 21,198 protein-coding genes, of which 97.3% genes were functionally annotated. The manuscript further characterises the phylogenetic relationships between T. bleekeri and other species with the genus Triplophysa, describes gene family expansion and contraction in the T. bleekeri genome, identifies genes potentially affected by positive selection by estimating the ratios of nonsynonymous to synonymous substitutions and estimates population divergence among three populations based on short-read sequencing data of 28 individuals.
Overall, the assembly and annotation of the genome is well-described and current manuscript is one of several similar works on fish species living on Tibetan plateau. In contrast to genome assembly and annotations, some additional analysis parts of the manuscript, such as population genetic analysis of 28 are rather brief and do not add considerably to the current manuscript. Hence, it would have been interesting if the authors would put more emphasis here, for example by using demographic modelling (e.g. using fastsimcoal2 or other software). Also, it would be interesting if the authors would carry out PSMC analysis to further shed light on temporal changes of effective population size in T. bleekeri which would be very relevant in the context of Qinghai-Tibetan Plateau uplift. My main criticism of the manuscript relates to language and grammar. Clearly, the language of the manuscript needs serious editing. In the detailed comments/suggestions, I have pinpointed some, but not all, of the most obvious cases. I suggest that the authors will use competent native-speaker language assistance to improve the language throughout the paper. In addition, several conclusions that are based on weak reasoning should be removed or softened (see below).

Response: Dear reviewer, thank you for your kind comments for our manuscript. We appreciate your valuable comments and suggestions to improve our paper. With regard to your comments and suggestions, we wish to reply as follows:
(1) To take further advantage of the whole-genome resequencing data, we have performed the PSMC analysis to investigate the historical effective population size dynamics for T. bleekeri. Meanwhile, we also carried out selective sweep analysis to identify selected genes among the population.
(2) As you pointed out, the grammatical, typographical, and formatting errors in this manuscript have been revised. Additionally, the language of this manuscript was improved by Wiley Editing Services.
(3) According to your kind suggestions, some inappropriate conclusions were revised. The specific changes were marked by using the track changes mode in MS Word and were elucidated in the part of the response to your detailed comments.
Overall, we hope that the changes having been made to the manuscript meet to your satisfaction. Special thanks to you for all your good comments.

Detailed comments:
Comment 1: Lines 25-26: Consider rewording, "These environmental changes induced strong stresses for several organisms,.."
Response: Thanks for your suggestion. We revised this sentences in the abstract (lines: 28~29, Marked MS).

Comment 2: Lines 37-38: Replace "underwent strong natural positive selections..." with "have experienced positive selection".
Line 39: replace "samples from populations" to "samples from three populations".
Line 41: Replace "can be attributed to the disrupted gene exchanges among those populations" to alternative description that gene flow is restricted between populations. Based on the data from 3 locations, it is not possible to identify if these changes have been caused by man made changes (dam) or are occurring naturally.
Response: We revised this sentences mentioned above in lines 43, 46, and 48~49 (Marked MS). Special thanks to you for your good suggestions.

Comment 3: Lines 51-53: Consider rewording "The endemic species of the QTP present a great adaptability to the harsh environments of low temperature and low oxygen". It is not clear what does "a great adaptability" means in this context. Also, if the land rise have increased the river slopes at QTP, it is possible that the oxygen levels in the water are actually reduced. Do you have data on oxygen concentration in QTP rivers to demonstrate that high altitude causes low concentration of O2 in water? I think this is actually quite important, as the high-altutude adaptations in fish because of low oxygen maybe not so widespread at all (you introduce the theme later in the intro).
Response: Thanks for your kind comments. We are very sorry for our inappropriate description, and we revised this sentence in lines 64~67 (Marked MS).
QTP is the highest and largest plateau in the world, imposing an extremely inhospitable environment, including low temperature, oxidative stress, and UV radiation, on native creatures. This harsh environment has resulted in the evolution of similar phenotypic adaptations in humans, mammals, birds, and so on, for example, cold tolerance, hypoxic resistance, enhanced metabolic capacity, and increased body masses 1-3. However, different organisms adapt to high altitudes via multiple genetic routes 4-6. The dissolved oxygen level in the water of QTP rivers is not as low as we predicted. Reaeration is one of the main sources of dissolved oxygen in the water of QTP rivers, given that the flow velocity is relatively fast 7. The previous studies showed that the minimum dissolved oxygen level is about 6.5 mg/L even in the elevation of 4,000 m in many rivers of QTP 7-9. Therefore, we think that low temperature is a typical feature of the river environment on the QTP, which faced up this challenge for all aquatic animals. We also explain this viewpoint in the part of the Discussion (Lines 625~629, Marked MS).

Comment 4: Lines 79-80: Consider rewording "environmental adaptation mechanism of Triplophysa species is far from fully understood".
Response: Thanks for your kind suggestions. We reworded this sentence in lines 98~99 (Marked MS).

Comment 5: Line 90-109: This section need substantial rewriting as it contains numerous language issues. E.g. Line 90-109: Replace "was" to "is"; Line 92: Remove "just", replace foods to food, is still crucial to is crucial, larva to larvae etc.
Response: These sentences were revised based on your kind suggestions (lines: 112, 115, 123~124, 127, and 124; Marked MS).

Comment 6: Line 115: How were the individuals caught?

Response: The fish were caught by brail nets, and this information was added in the manuscript (line: 148; Marked MS).

Comment 7: Line 120: Did you anesthetize fish or use overdosage of MS22 to euthanize fish?
Response: Yes. We use the tricaine MS-222 (60 mg/L) to anesthetize fish before dissection and sampling. We referenced the previous study 10 and conducted the preliminary experiment to determine the dose.

Comment 8: Line 126: Replace "to obtain a comprehensive transcript" with "to comprehensively characterize transcriptome".
Response: Thanks for your kind suggestion. This sentence was revised in lines 158~159.

Comment 9: Lines 128-129: Did you use any anesthetic? Did you use samples from multiple individuals as indicated in text i.e. "muscle tissues were collected"
Response: Yes. In this case, we also use the tricaine MS-222 to anesthetize fish, as mentioned before. Besides, the muscle tissue of each fish was collected for resequencing. We are sorry for our unclear delineation, and we added the related information in the manuscript (line: 164~165; Marked MS).

Comment 10: Lines 183-184: Change T. bleekeri to italics.
Line 192: Change wording "from the above fish"
Lines 243-245: Remove the Latin names in brackets
Line 261 Remove dot.
Line 276-277: Confusing sentence, consider rewording "the natural selected genes" to " "genes putatively influenced by positive natural selection".
Line 282: Change to "Evolutionary relationships"
Line 292: Replace "genetic structures" to "genetic divergence among T. bleekeri populations"
Response: Thank you very much for your kind review. We revised these sentences according to your kind suggestions (lines: 221, 230, 254~257, 317, 333~334, 338 and 348; Marked MS).

Comment 11: Line 300: The minimum depth seems very low (3) which suggests that heterozygote genotypes are not called correctly.
Response: Thanks a lot for reminding. This is the depth filtering parameters for the SNPhylo, but not for filtering parameters during the SNP calling process. We have added the detailed parameters used for the SNP calling. As we showed in the revised manuscript, the minimal depth for SNP calling is 5 in this analysis, which is higher than the Minimum_depth_of_coverage of 3 in SNPhylo analysis. We have increased the minimal depth requirement to 5 and re-preform the analysis, leading to an identical result. Meanwhile, the parameters of SNPhylo would not influence the phylogenetic relationship, and the following population genetics analysis were not significantly influenced (line: 362; Marked MS).

Comment 12: Lines 305-306: Replace "was performed to test the topology robust" to " was performed to test the robustness of the tree topology"
Line 309: Change "transcriptome" to "transcriptomic"
Line 350: Change to "transcriptome data from 12 tissues was generated".
Lines 359-360: Consider rewording: "Meanwhile, the important function of the non-coding genes received further attention."
Line 361: Replace "non-coding gene loci" to "non-coding DNA sequences"
Line 366: no need to repeat two times significantly
Response: Thanks for your kind suggestions. We revised these sentences in lines 367~368, 394, 435, 445~447, 449, 514~516 (Marked MS).

Comment 13: Both Supplementary Table S4 and S5 indcate gene family expansions in the heading. Should one of the table represent gene family contractions? With Rich factor, do you mean enrichment factor?
Supplementary Table S4. In the first row, both the gene number and background gene number is one and Q value is 0?
Response: We are very sorry for our negligence. We added the tables of gene family contractions in the Additional File.
(1) Yes, the rich factor here is the enrichment factor, indicating the ratio of selected genes with one GO/KEGG annotation divided by the total number of genes with the identical annotation.
(2) Yes. Both the gene number (in Table S5) and background gene number (in Table S4) are one means the function was extremely enriched. However, the statistics here are not reliable since the sample size is too small here. We have removed the lines with gene number smaller than 5 in Supplementary Table

S4 and S5.

Comment 14: Line 381: As this type of analysis relies heavily on what species were included to the analysis, please describe here what species were used.
Response: To identify positively selected genes (PSGs) in the T. bleekeri genome, we compared gene families in the T. bleekeri genome to eight fish species living in non-QTP regions, including Callorhinchus milii, Lepisosteus oculatus, Gadus morhua, Danio rerio, Oryzias latipes, Xiphophorus maculatus, Takifugu rubripes, and Larimichthys corcea. Similarly, the PSGs of T. tibetana and T. siluroides were obtained in the same way. We introduced this information in the section of materials and methods (lines: 324~325, 333~335; Marked MS). Thank you for your kind review.

Comment 15: Line 407: Please change "obtained according to serious filtering criteria". It is not clear what "serious" filtering means here. Based on the relaxed coverage, actually the filtering seems to be very relaxed? Also, did you checked for Hardy-Weinberg equilibrium and was that included as a filtering parameter?
Response: We have applied the filtering criterion during SNP calling according to the suggestion from GATK manual: QUAL (phred quality) > 30, QD (quality score divided by depth to comprehensively evaluate the quality and depth) > 2, read depth (DP > 5), FS (phred-scaled p-value using Fisher's Exact Test to detect strand bias for reads) < 60, MQ (mapping quality to evaluate read alignment) >40, SOR (strand odds ratio to evaluate strand bias for reads) < 4.0. We have added detailed SNP filtering during SNP calling process in the revised manuscript (lines: 355~360; Marked MS). We did not include the Hardy-Weinberg equilibrium test in the SNP filtering.

Comment 16: Lines 411-414: IN the population genetics, the term sister group is not frequently used. Also, what do you mean with monophyly here? I suggest removing this term.
Response: Thanks a lot for the reviewer's suggestion. We have changed the "sister group" to "neighboring group", and we have removed the term of monophyly from the revised manuscript (lines: 566~567; Marked MS).
Comment 17: Line 448: Try to avoid superlatives like "was enormously influenced by uplifts"
Response: Thank you for your kind suggestion. We deleted the inappropriate rhetoric in lines 625~626 (Marked MS).

Comment 18: Line 451: Again, it is not clear to me if the oxygen concentrations in the water have actually deceased in the fast flowing rivers. One can argue that eutrophication likely creates much more frequently decrease of O2 in aquatic environments.
Response: Thank you for your kind review. As I mentioned before, the dissolved oxygen level of the water of QTP rivers is high. The fast flow rate can contribute to the high dissolved oxygen level in the water since reaeration is a primary source of dissolved oxygen. Additionally, a smaller number of aquatic plants and phytoplankton in the water of QTP rivers, especially in some big rivers, such as the Shiquan River 11. Therefore, the eutrophication barely occurs in the water of QTP rivers.

Comment 19: Lines 452-454: This is very confusing and contradictory, you state here that in QTP rivers the oxygen concentrations are high! (which supports my earlier point).
Response: Thank you for your kind review. We are very sorry for making you confused due to our imperfect information. We added the related information in lines 629~631 (Marked MS).
The oxygen concentration of air is scarce in the QTP. However, many previous studies showed that the dissolved oxygen in the water of QTP rives is sufficient7-9. Hypoxia is a considerable living challenge for the terrestrial animal of QTP. Compared with the terrestrial animal, the primary living challenge for the fish is low temperature rather than hypoxia, given that the dissolved oxygen level is not low, and that the body temperature of fish depends on water temperature.

Comment 20: Lines 458-459: This study does not really study "the mechanisms by which Triplophysa species adapt to low temperatures". Consider rewording.
Response: We revised this part according to the reviewer's kind suggestion (lines 635~637; Marked MS).

Comment 21: Lines 466-475: The description of the links between low temperatures, ROS and apoptosis is quite speculative and not easy to follow.
Response: Thank you for your kind review. It is true that the link between low temperature and ROS/apoptosis is weak. We have deleted the part of ROS and apoptosis in the manuscript (lines 657~667; Marked MS).

Comment 22: Line 462: Based on supplementary tables and results section, there were also many other gene families that were enriched?
Response: Thanks for your kind review. According to your instruction, we added some key information about expanded gene families in this paragraph (lines 640~642, 648~649; Marked MS).

Comment 23: Lines 484-485: This is highly speculative. Instead, common ancestry seems to be more parsimonious explanation.
Response: Thanks for your kind comments. We have revised this whole paragraph and pointed out that those naturally selected genes might originate from their common ancestor (lines 670~694; Marked MS).

Comment 24: Line 488: Again, please try to avoid superlatives. Not sure what "massive genes" means. Also not sure what "uniquely naturally selected" means.
Response: According to your kind suggestions, we deleted this inappropriate modifier in the sentences (line: 696; Marked MS).

Comment 25: Line 493: Consider removing "sister group".
Response: We deleted these words and reworded the sentence in lines 709~710 (Marked MS).
Comment 26: Line 508: Reword "hypoxia environment". The question is if the habitat of T. bleekeri really has low concentrations of dissolved oxygen.
Response: Thanks for your kind suggestion. We deleted the inappropriate description and reworded the whole paragraph of the Conclusion in lines 756~762 (Marked MS).

Comment 27: Lines 509-510: Consider rewording "It is fascinating to investigate the molecular mechanisms for their environmental adaptation to the QTP" as this sentence does not fit conclusions section.
Response: Thanks for your kind suggestion. We deleted this sentence, and revised the whole paragraph of Conclusion (lines: 756~762; Marked MS).

Comment 28: Lines 520-521: Consider softening the claim as the evidence to link genomic features and environmental parameters is weak at best.
Response: Thanks for your kind suggestion. We deleted the inappropriate description and reworded the whole paragraph of the Conclusion (lines: 759~762; Marked MS).

Comment 29: Lines 521-523: Consider rewording. This claim can be states basically for every freshwater fish without any genetic data.
Response: Thanks for the referee's kind advice. We revised these sentences in the part of the Conclusion (lines 759~762; Marked MS).

Comment 30: Lines 528-529: Consider rewording high land fish"
Response: As the reviewer suggested that we deleted the inappropriate description and reworded the whole paragraph of the Conclusion (lines: 759~762; Marked MS).

Reviewer #2
Major comment:
The paper by D. Yuan and collaborator entitled "Chromosomal genome of Triplophysa bleekeri provides insights into its evolution and environmental adaptation" is a "genome" paper aiming to published a good reference genome for the species T. bleekeri. It also provides a description of potential adaptation to altitude/temperature in Tibetan plateau.

I think that the main aims of producing a good genome for the species is achieved by this paper. However, I find the analyses rather descriptive and therefore the paper is a bit heavy to read. In addition, the gene with potential signal of positive selection should be discuss with more caution see https://royalsocietypublishing.org/doi/full/10.1098/rsbl.2018.0055 that describe some bias of the method use in this paper, but many other paper like this exist)

As the introduction rely a lot on the comparison of different Triplophysa species; I was hoping more comparative discussion in between the different species of Triplophysa that is not really done at the moment, or at least in a confusing way.

In addition, I think that the population genomics analyses could be pushed further. At the moment, the 28 WGS are basically not used in the current paper. I think that classical pop. genomics analyses such as detection of selective sweep, Fst outlier scan and visual variation of statistic such as pi or Fst between pair of populations across T. bleekeri genome would help to frame a nice storytelling to describe the adaptation to the altitude. They would be obvious comparison to do with their other selection test based on dN/dS ratio.
However, this would need some substantial modification of the paper. If producing a genome is still enough to publish a paper nowadays, then, you don't necessarily need to include this change. However, I feel that including better pop. genomic analysis would make the paper more interesting.

Some sentences are also a little bit wordy sometime. I suggested some changes below but I might not be the best candidate for this as English is not my native language. Response: Dear reviewer, we sincerely appreciate the valuable comments and suggestions.
Thanks a lot for the reviwer's comments. The caution should be taken when interpreting the positively selected genes since the biases of selection signature generated from the ancient demographic fluctuation might lead to false positive of selected gene identification. To comprehensively describe the natural selection result of genomic comparison and to avoid unnecessary misunderstanding, we have included a statement to reveal the potential biases from this study in the Discussion section.
According to your kind suggestions, we added some comparative discussion in between the different species of Triplophysa in the Discussion section. Besides, we performed the PSMC and selective sweep analysis using genome resequencing data. Meanwhile, we have tried our best to polish the language in the revised manuscripts, and the language was edited by Wiley Editing Services. We hope that our manuscript can meet your standards. Thanks again.

Detailed comments:
Comment 1: line 26 : why especially fish ? this is not clear to me. Because they are ectotherm? Maybe this is not really interesting here, every organism are constrain in one way or another by their environment.
Response: Thank you for your kind review. We deleted the inappropriate description and revised this part according to your comment (lines 28~31; Marked MS).

Comment 2: line 82: what is a "typical" species of a given family? maybe remove this.
Response: Thanks for the referee's kind advice. We deleted this word in lines 101~102 (Marked MS).

Comment 3: Line 84: this sentence doesn't flow correctly we the one above. Considered changing "although" by something else.
Response: Thanks for referee's kind suggestion. We deleted this word and revised the sentences in lines 106~107 (Marked MS).

Comment 4: line 86: need a ref here
Response: Thanks for your kind review. We added the reference in the line 107 (Marked MS).

Comment 5: line 92-> 96= this is wordy, rephrase.
Suggestion: The reproduction biology of Trilophysa fishes is also different, while T. tibetana and T. siluroides spawns once a year (from XX to XX and XX to XX, respectively), T. bleekeri can spawn twice a year with peak breeding seasons occurring from October to December and March to April (Wang et al., 2013)
Response: We revised this sentence according to your kind suggestion (lines 118~122; Marked MS). Special thanks to you.

Comment 6: line 100: why is this "crucial"? I would like to see the specific aims of the study mentioned in here before summarizing the results.
Response: Thanks for your comment. According to your kind guidance, we reworded this part in lines 128~130 (Marked MS).

Comment 7: line 108-109 crucial again. This sentence could be removed as it doesn't provide any relevant information. In my opinion, we should always let reader make their mind about what is "crucial" or not…
Response: We deleted this sentence according to your kind suggestions (lines 139~143; Marked MS). Thanks.

Comment 8: line 128: reword sacrificed: suggestion: To understand the population structures of the species, a total of 28 individuals were collected from three sampling sites, including eleven, seven, and six individuals from populations 1, 2 and 3, respectively (Fig. 2).
Response: Thanks for your kind review. According to your suggestions, we reworded these sentences (lines 161~164; Marked MS).

Comment 9: line 174: precise that this is a software
Response: Thanks for your review. We added the related description in line 211 (Marked MS).

Comment 10: I think that line 179-180 repeat line 174-175
Response: Thanks for the referee’s kind comment. We are very sorry for our negligence, and this sentence was deleted (lines 215~217; Marked MS).

Comment 11: line 300: need more explanation about the filtration step. You should not do filtration for LD but for physical linkage. Lots of information are found in the LD information. Was LD performed overall? A depth of 3 is also really low
Response: Thanks for your kind review. Sorry that we have missed the SNP filtering details in the manuscript. According to the suggestion from GATK manual, the filtering criterion was QUAL (phred quality) > 30, QD (quality score divided by depth to comprehensively evaluate the quality and depth) > 2, DP (read depth) > 5, FS (phred-scaled p-value using Fisher's Exact Test to detect strand bias for reads) < 60, MQ (mapping quality to evaluate read alignment) >40, SOR (strand odds ratio to evaluate strand bias for reads) < 4.0. We have added detailed SNP filtering during SNP calling process in the revised manuscript (lines: 357~361; Marked MS). Since the minimal depth parameters of 3 for SNPhylo is smaller than the depth requirement of 5 during SNP calling, therefore the minimal depth parameters of 3 for SNPhylo does not work. We have increased the minimal depth parameters to 5 and reperformed the SNPhylo analysis and obtained identical result. We have revised the manuscript for the depth of 5 (line: 362; Marked MS).
Meanwhile, we are sorry that our method description for SNP filtering is confusing. The Linkage Disequilibrium (LD) filtering was only used for phylogenetic tree construction using SNPhylo utility.

Comment 12: I would like to see a PCA in your main paper and more analyses showing the variation of classical pop. gen. statistic such as Fst and pi along the genome.
Response: Thanks for the comment. We have added the statistics analysis in the revised manuscript (SI Table 10).

Comment 13: line 444: Nothing is "primitive" when species are still living nowadays, refers as "outgroup", "most divergence species" or something else.
Response: Thanks for the referee’s kind advice. We reworded this sentence in line 622~623 (Marked MS).

Comment 14: line 448 : double space. You could also rephrase this sentence
Response: We revised the format and rephrased this sentence according to your suggest (line 626; Marked MS).

Comment 15: line 494:498 this should be back up by value of average Fst
Response: Thanks for the comment. We have calculated the Fst value for LHK-XX, LHK-BY, BY-XX population comparison. As a result, 1,734, 3,009 and 3,244 regions (1 kb), harboring 474, 878, and 957 candidate functional selected genes, were identified to be the significantly genetic differentiated among LHK-XX, LHK-BY and BY-XX comparisons, respectively. Less genomic differentiated regions and candidate genes identified in LHK - XX comparison were consistent with our previous phylogenetic analysis. We have added the result in the revised manuscript (lines: 576~584; Marked MS).

Comment 16: line 503: this is preliminary but you have the data to explore this further. I don't understand why this is not done. At the moment, the population genetic analyses add really little to no information to your current paper.
Response: Thanks a lot for reviewer’s comments. We have added a selective sweep analysis using genome resequencing data and identified candidate functional genes in differentiated genomic regions. The GO and KEGG pathway functional analyses were performed to identify the functional enrichment of those candidate genes. The relationship of gene functions to local environments was discussed in the Discussed section (lines: 728~752; Marked MS).

Reviewer #3
General comment:
In this manuscript Yuan and colleagues report the chromosome-level genome assembly of the fish species Triplophysa bleekeri and carried out several comparative genomics analyses in order to investigate the genomic mechanisms that facilitated its adaptation to high-altitude environment. To date, two other chromosome-level genome assemblies are available for the Triplophysa genus, but I found the addition of the T. bleekeri genome a valuable resource considering its wider altitudinal distribution range.

The authors used a state-of-the-art "recipe" to obtain chromosome-level assemblies by relying on the use of PacBio long reads, followed by Illumina short reads quality correction and Hi-C scaffolding technique. Notably, very high depth of coverage was used for the three sequencing types applied, and it allowed Yuan et al. to obtain very impressive statistics (e.g. contigs N50 = 3.1 Mb, scaffold N50 = 22.9 Mb).
The authors then predicted protein-coding and non-coding genes in the newly generated assembly and they included these data in a phylogenetic framework. Among the main findings, they identified several genes under positive selection in T. bleekeri (many of them also positively selected in the other two species of the same genus) and a bunch of expanded and contracted gene families with functions potentially related to the adaptation to high-altitude environment. Finally, they re-sequenced 28 T. bleekeri individuals representative of three different populations to provide preliminary data on the population structure of this species. In general, the methods used are sound.

That said, I see room for improvement. The manuscript currently is plagued by some disorganization in presented materials and suffers from some clarity issues in analysis descriptions. Additionally, I feel that the manuscript is not particularly well written and I find very hard to follow some sections. Many sentences would benefit from being re-written and in general all the manuscript should be proofread before to be resubmitted.
Response: Thanks a lot for reviewer’s comments and suggestions. We have added more details for the method and re-phased the whole manuscript for a better understanding. Sentences that might lead to obscurity for readers have been rewritten in the revised manuscript. Additionally, the language of this manuscript was improved by Wiley Editing Services. We hope that our manuscript can meet your standards. Special thanks to you.

Major comment:
Comment 1: The methods used are robust for the many analyses the authors carried out. However, I think the results could be presented in a better way by at least re-organizing the order of appearance of the different sections, including more information and refining the main figures. The Results section starts with the genome assembly, but I would then describe ortholog detection immediately followed by the phylogenetic analysis. It would help the reader to contextualize their species with the others included in the phylogeny. I would then go with positive selected genes and only after them describe the expansion/contraction of gene families.
Response: Thanks for your kind suggestions. We re-organized the order of the Results section and revised figures and languages. We very appreciate your suggestion and review.

Comment 2: I am not sure I see the value of the re-sequencing analysis. How the three populations were selected? Do they live in different environments? I would have chosen three populations living at different altitudes (T. bleekeri has a wide distribution ranging from 200 to 3,000 m), as it would have helped, for example, to identify genomic regions of higher divergence in comparison to the background (is it the case?). This in turn would have helped to identify candidate regions potentially harboring genes responsible for adaptation to different high-altitude environment. These candidate genes could have then been compared to the ones identified with the positive selection analyses using the multi-species approach. To sum up, the rationale behind populations selection needs to be better described, and this dataset better explored (e.g. PCA and Admixture are OK, but I would like to see some basic population genetic metrics like e.g. Fst, and Fst values can be shown using Manhattan plot-like style for each pairwise comparison). Finally, I am not sure the tree of Fig. 3 (quite poor graphically) is the best way to report these results. I would suggest the author to produce a more comprehensive new Figure made of 3 panels showing the tree plus the PCA and the Admixture plot (now Suppl. Fig 6 and 7).
Response: Thanks for your kind review.
For the genome sequencing, we collected the T. bleekeri from the high altitude region of the Daning River. For the population re-sequencing, T. bleekeri populations were selected from three different reaches of the Daning River (i.e., Lianghekou, Xixi, and Baiyang) because they are separated by natural or artificial barriers. We would like to investigate how those barriers block gene exchange and diversity among those populations. We have performed the PSMC and selective sweep analysis using the population data and added results into the revised manuscript.
We also had planned to collect T. bleekeri from regions with different altitudes, such as the Zhougong River (~2,000 m) and Qingyi River (~3,000 m). However, it is hard to get a permit to fish in these areas because fishing is forbidden for the whole year. In Daning River (~1,000 m), fishing is just banned in spring, so we chose to collect fish in the Daning River. For the genome sequencing, we collected the fish from the high altitude region of the Daning River.
We have performed selective sweep analysis, and Manhattan plot was generated to show the result. We also re-generated a figure for the population genetics analysis according to the reviewer’s suggestion.
If there is any chance, we’d like to collect fishes living at different altitudes to study the genetic divergence among different populations. Thanks for your good suggestion.

Comment 3: The Discussion is very redundant. Frequently the results are reported exactly as they are reported in the Results section, and only very briefly discussed. I would shorten the Discussion by highlighting the main points and including more interpretation of the results and real discussion. The Conclusion section, again, is a repetition of what have been said before in the Discussion. I would delete this section and implement the few original sentences in the Discussion.
Response: Thanks for your kind suggestions. We deleted the redundant part and revised the whole section of the Discussion and Conclusion. The modified parts of the manuscript were highlighted by using the track changes mode. We hope that the correction will meet with approval.

Minor points:
Comment 1: L. 27: "Triplophysa bleekeri (T. bleekeri)"
The "(T. bleekeri)" is not necessary here. Use T. bleekeri later in the text in the same section, and maybe re-use the full name the first time you mention it in a new section.
Response: We deleted the "(T. bleekeri)" in the sentences in accordance with your advice (line 31; Marked MS).

Comment 2: L. 127.128: 11+7+6=24, but here I read 28 individuals.
Response: We are very sorry for our negligence. We sampled eleven, eleven, and six individuals from three reaches of Daning Rivers (i.e., Lianghekou, Xixi, and Baiyang). We revised this clerical error in lines 161~164 (Marked MS).

Comment 3: L. 32-34: "Phylogenetic analysis showed that T. bleekeri diverged 38.8 and 28.6 million years ago (Ma) from their common ancestors of Triplophysa siluroides and Triplophysa tibetana, respectively"
This sentence can be removed from the abstract, or simply replaced with something mentioning that you resolved the phylogeny of the genus Triplophysa, without numbers.
Response: We removed this sentence from the abstract in conformity with your kind instructions (lines 37~39; Marked MS). Thanks very much.

Comment 4: L. 77: change "board" to broad"

Response: We are very sorry for our negligence, and we revised this spelling mistake (line 91; Marked MS).

Comment 5: L. 82 and 84-85: see my comment above.
Response: We have made corrections according to your comments (lines 101, 107; Marked MS).

Comment 6: L. 103: Hi-C is not a platform, it is a method. Please rephrase it.
Response: We deleted this word in the sentence (line 132; Marked MS). Thanks for your advice.

Comment 7: L. 115: Full name here.
Response: We changed “T. bleekeri” to “Triplophysa bleekeri” in conformity with your kind instructions (line 146; Marked MS). Thanks very much.

Comment 8: L. 126: change "comprehensive transcript" to "comprehensive transcript set" or "comprehensive transcriptome".
Response: Thanks for the referee’s kind advice. We reworded these words in line 158~159 (Marked MS).

Comment 9: L. 128: See my main comment above. Again, can you use a name for the populations instead of 1, 2 and 3? Both here and in the Figure (now Fig. 6).
Response: Thanks for your kind suggestions. We fished the T. bleekeri form different river sections, including Lianghekou (LHK), Xixi (XX), and Baiyang (BY). Therefore, we use the LHK, XX, and BY instead of 1, 2, and 3. We revised the population names in the whole manuscript.

Comment 10: Most of the versions of the program used are missing. Please revise it, choose a style (e.g. v1.2; version 1.2; v 1.2 etc) and be consistent.
Response: Thanks a lot for reviwers’ reminding. We have added software version information in the revised manuscript.

Comment 11: L. 135: Change "Hiseq" to "HiSeq", and check this and other names for consistency along the text.
Response: We revised this clerical error and checked the all names of software in the manuscript (line 171; Marked MS).

Comment 12: L. 137: "Pacific Biosciences of California," "of California" not required.
Response: We deleted these words in the line 173 (Marked MS). Thanks for your kind review.

Comment 13: L. 183: species name in italics
Response: We are very sorry for our incorrect writing, and we revised this error (line 221; Marked MS).

Comment 14: L. 190: The database used for the BUSCO analysis should be specified here.
Response: The database of actinopterygii_odb9 was used for the BUSCO analysis. We have added it to the revised manuscript (lines: 227~228). Thanks for reminding.

Comment 15: L. 213: change "de novo" with "ab initio"
Response: Thanks for your kind suggestion. We changed "de novo" to "ab initio" in the line 251 (Marked MS).

Comment 16: L 226: No details are reported on how you filtered the genes predicted by Maker. I would add the quality filtering step here (genes predicted in repetitive regions were removed? Any overlapping threshold used? Genes with internal stop codons removed? etc).
Response: Thanks for your comment. The quality of gene prediction was filtered as the following steps: We only selected genes with the start and stop codons, and gene with internal stop codons was removed. Only genes with completed sequences and 70% overlap among different gene model prediction methods will be retained as high-quality gene models. We have added the filtering details in the revised manuscript (lines 265~267; Marked MS).

Comment 17: L. 243-245: I would use common names for each species here, and scientific full names in parenthesis.
Response: Thanks for your kind advice. We revised this part according to your instructions (lines 282~285; Marked MS).

Comment 18: L. 253: Which model did you use in RaxML?

Response: Thanks for reminding. Phylogenetic analyses were performed using RAxML with a GTRGAMMA model. We have added the information to the revised manuscript (line 294; Marked MS).

Comment 19: L. 257: This is too generic. Which calibration points did you use?
Response: We used the divergence time of Danio rerio and Larimichthys crocea (255-205 Ma), Oryzias latipes and Larimichthys crocea (115-105 Ma), Lepisosteus oculatus and Danio rerio (338-291 Ma), and Callorhinchus milii and Danio rerio (497-450 Ma) as calibration points for the divergence time estimation for other species. We have added the information to the revised manuscript (lines 297~301; Marked MS).

Comment 20: L. 288-291: I would include more details here. For example, which Trinity transcripts did you use for the ortholog detection analysis? The longest transcript for each gene? The pipeline you used should be better described.
Response: Thanks for your reminding. The longest transcripts for each gene were used for the phylogenetic analysis. We have reordered sections in the method and added more details for the analysis pipeline (lines 302~310; Marked MS).

Comment 21: L. 426: In the abstract the contigs N50 is 3.1, here 3.82. Please use the right value and be consistent. Again, I would report the scaffold N50 here? Talking about chromosomes: is the karyotypic number known for this genus or this species, for example from cytogenetic studies? In case, I think it is worth mentioning and discussing it (karyotypic number from cytogenetic vs. assembled chromosomes).
Response: Sorry that we should clarify here that contig N50 of 3.82 Mb is the assembly result from sole PacBio sequencing data. However, contigs might be broken during the scaffolding procedure using Hi-C data analysis. Therefore, we reported the contig N50 after Hi-C based assembly in the abstract. To clarify and to keep the consistent description of the assembly, we revised the contig N50 length and added scaffold N50 value here (line 601; Marked MS).
Our laboratory studied the karyotype of Triplophysa bleekeri, revealing that Triplophysa bleekeri has 50 chromosomes (2n=50). The results are as follows:

We referenced this results when we assembled the genome of Triplophysa bleekeri. Finally, 96.2% of the total contig bases were anchored to the 25 chromosomes. According to your suggestion, we added the related discussion in lines 603~606 (Marked MS).
Comment 21: L. 444: what "primitive" means? Do you mean "basal"?
Response: We are very sorry for our incorrect writing. The phylogeny results indicated Triplophysa siluroides is a basal species in the Triplophysa genus. We revised the sentence in line 622~623 (Marked MS) according to your kind instruction.

Comment 22: I would combine Fig. 1 and 2 in a single figure.
Response: Thanks for your kind suggestion. We combined Fig.1 and Fig.2 into one figure.

Comment 23: Fig. 3: can be moved to supplementary material.
Response: We moved the Fig.3 to supplementary material according to your kind suggestion.

Comment 24: Fig. 4: Did you carry out the positive selection analysis for each of the three species independently and then check for overlap? If so, I would also include a PAML analysis to identify positive selected genes in the lineage that includes all Triplophysa species.
Response: Yes, we performed the positive selection analysis for each of the three species independently. According to the reviewer's suggestion, we also performed the PAML analysis and identified 439 PSGs for Triplophysa ancestral lineage. We have compared the species- and lineage-based PSGs and found that only 35 PSGs from lineage-based identified PSGs were identical with those from species-based identified ones. The homologous recombination and basal transcription factor pathways were both enriched for lineage- and species-based PSG identifications. We have added the new results in the revised manuscript (lines 496~508; Marked MS).

Comment 25: I would suggest to make the phylogenetic tree the new Fig 2 and improve its quality. Small images of fishes could make the figure nicer and easier to read. Additionally, the confidence intervals at each node could be shown graphically and not with numbers (very difficult to read and interpret).
Response: We have revised the time bars in phylogenetic tree and added the fish images in Figure 2.

References
1 Monge, C. & Leonvelarde, F. Physiological adaptation to high altitude: oxygen transport in mammals and birds. Physiological Reviews 71, 1135-1172 (1991).
2 Wu, T. & Kayser, B. High Altitude Adaptation in Tibetans. High Altitude Medicine & Biology 7, 193-208 (2006).
3 Beall, C. M. Two routes to functional adaptation: Tibetan and Andean high-altitude natives. Proceedings of the National Academy of Sciences of the United States of America 104, 8655-8660 (2007).
4 Li, M. et al. Genomic analyses identify distinct patterns of selection in domesticated pigs and Tibetan wild boars. Nature genetics 45, 1431 (2013).
5 Hao, Y. et al. Comparative transcriptomics of 3 high-altitude passerine birds and their low-altitude relatives. Proceedings of the National Academy of Sciences of the United States of America 116, 11851-11856 (2019).
6 Li, J. et al. Comparative genomic investigation of high-elevation adaptation in ectothermic snakes. Proceedings of the National Academy of Sciences of the United States of America 115, 8406-8411 (2018).
7 Lyu, L., Li, Z., Cui, C., amp, T. A. & University, A. H. Study on the Variation of Dissolved Oxygen in the Plateau River. Environmental Science & Technology (2018).
8 Zhang, N. et al. Spatio-Temporal Characteristics of Niyang River in Tibet. Journal of Henan Normal University (2009).
9 Li, H., Zhang, N. & Lin, X. Spatio-Temporal Characteristics of Yarlung Zangbo River in Tibet. Journal of Henan Normal University (2010).
10 Zhang, J. M., Guo, B. F., Yuan, T. & Tian, T. Anesthetic Effect of MS-222 on Elongate Loach Leptobotia elongata. Chinese Journal of Fisheries (2014).
11 Wang, D. The geography of aquatic vascular plants of Qinghai-Xizang (Tibet) Plateau (in Chinese). Dissertation for the Doctoral Degree. Wuhan: Wuhan University 85 (2003).

Close
