# Supplementary material for: Chromosomal genome of Triplophysa bleekeri provides insights into its evolution and environmental adaptation
Source: Gigascience. 2020 Nov 24;9(11):giaa132. doi: 10.1093/gigascience/giaa132 (PMC7684707; doi:10.1093/gigascience/giaa132)
Supplement: giaa132_Response_to_Reviewer_Comments_Revision_1 [file giaa132_response_to_reviewer_comments_revision_1.pdf]

Author's Response To Reviewer Comments

Close

Manuscript number: GIGA-D-20-00124
Article Type: Data Note
Title: Chromosomal genome of Triplophysa bleekeri provides insights into its evolution and environmental adaptation
Correspondence Author: Shijun Xiao; Zhijian Wang

Dear editor,
Thank you very much for your hard work in processing our manuscript. We have carefully read the suggestions from the reviewer and modified the manuscript based on these suggestions. We submit a revised manuscript here. The following are the correspondences to your reviewer concerning the comments and suggestions about the manuscript.
We wish to take this opportunity to thank your consideration of our paper published in your journal, GigaScience.

Best regards.
Sincerely Yours,
Dr. Zhijian Wang

Detailed responses to Reviewers
Reviewer #1
General Comment:
Compared to previous version, the authors have considerably improved both the content, presentation and readability of the manuscript. However, there are still issues that should be solved. For example, the discussion of the top 1% signals of the selection among 3 populations and specific candidate genes sounds extremely subjective and speculative. (see below). Hence I suggest to delete these parts in the discussion. In addition, there are still some issues with the language (see some suggestions for correction below, line numbers correspond to the version with changes visible).
Response: Dear reviewer, thank you for your kind comments for our manuscript. We appreciate your valuable comments and suggestions to improve our paper. We carefully revised the manuscript based on your kind suggestions. We hope that changes having been made to the manuscript meet to your satisfaction.

Detailed comments:
Comment 1: Line 266: Consider replacing „Only genes with completed sequences.." with „Only genes with complete sequences...".
Response: Thanks for your suggestion. We have revised this sentence (line 215, Marked MS).

Comment 2: Line 300: Consider replacing „...milii and Danio rerio (497-450 Ma) as calibration points for the divergence time..." with „...milii and Danio rerio (497-450 Ma) were used as calibration points for the divergen"ce time..."
Response: We revised this sentence based on your good suggestion (line 249, Marked MS).

Comment 3: Line 302: Consider replacing „To investigate the evolutionary relationship of species in Triplophysa genus.." with „To investigate the evolutionary relationships within genus Triplophysa...."
Response: Thanks for your kind comments. We revised this sentence in line 251 (Marked MS).

Comment 4: Line 446-447: Add references to following claim and replace „The non-coding genes have received increased attention in the recent year, since accumulating evidence suggests that many of them play crucial roles in a variety of biological process." With "The non-coding genes have received increased attention in the recent years, since accumulating evidence suggests that many of them play crucial roles in a variety of biological process.
Response: Thanks for your kind suggestions. We revised this sentence and added the reference (lines: 378~381, Marked MS).

Comment 5: Line 530: Do you mean: "We used the whole-genome short-read sequencing data based on the sample used for genome assembly to obtain the genome-wide genotype data."
Response: We are very sorry for our inappropriate description. We revised this sentence base on your comment (lines: 441~443, Marked MS).

Comment 6: Lines 534-537: I am not sure if I understood it correctly. If the authors used gene regions to estimate mutation rate between T. bleekeri and D. rerio and applied this estimate to analyze SNP data from the whole genome, it is certainly an underestimate for whole genome as gene regions are under functional constraint.
Response: Thanks for your good comment. It is a widely used model to estimate the mutation rate from the Ks estimation based on Ks=2μT, where Ks is the synonymous mutation rate, T is the divergence time between species, and μ is the mutation rate (Eyre-Walker, 2000; Koch et al., 2000; Zhang et al., 2013).

Comment 7: Lines 576: Consider replacing: "To investigate the possible natural selection among populations" with " To identify putative signals of differential selection among populations".
Response: We revised this sentence based on your excellent suggestion (line 468, Marked MS).

Comment 8: Lines 731-734: Pinpointing süecific selection agents as gorge or human activities is highly speculative given that only 3 populations were studied. I suggest to remove this part.
Response: We have removed this part according to the reviewer's suggestion (lines 592~595, Marked MS).

Comment 9: Lines 736-752: Give that the selection threshold for identifying potential footprints of selection was rather relaxed (the empirical top 1% of the distribution) and not based on any simulations to identify the likelihood of being under selection, I think the discussion on individual selection targets is way too speculative and selective (why the authors selected to discuss these few genes out of all other 1% genes?). Hence, I suggest to remove it.
Response: We have removed this part according to the reviewer's suggestion (lines 597~613, Marked MS).

Special thanks for your good comments and suggestions.

References

Eyre-Walker, A. (2000). Fundamentals of Molecular Evolution (2nd edn). Heredity 84, 735-735.
Koch, M.A., Haubold, B., and Mitchell-Olds, T. (2000). Comparative evolutionary analysis of chalcone synthase and alcohol dehydrogenase loci in Arabidopsis, Arabis, and related genera (Brassicaceae). Molecular biology and evolution 17, 1483-1498.
Zhang, L., Yan, H.-F., Wu, W., Yu, H., and Ge, X.-J. (2013). Comparative transcriptome analysis and marker development of two closely related Primrose species (Primula poissonii and Primula wilsonii). BMC genomics 14, 329.

Close
