# Supplementary material for: Chromosomal genome of Triplophysa bleekeri provides insights into its evolution and environmental adaptation
Source: Gigascience. 2020 Nov 24;9(11):giaa132. doi: 10.1093/gigascience/giaa132 (PMC7684707; doi:10.1093/gigascience/giaa132)
Supplement: giaa132_Reviewer_1_Report_Original_Submission — Anti VasemÃ¤gi -- 6/5/2020 Reviewed [file giaa132_reviewer_1_report_original_submission.pdf]

Reviewer Report

Title: Chromosomal genome of Triplophysa bleekeri provides insights into its evolution and environmental adaptation

Version: Original Submission **Date:** 6/5/2020

Reviewer name: Anti VasemÃ¤gi

Reviewer Comments to Author:

The manuscript by Ding et al describes the chromosome-level assembly of an endemic highland fish Triplophysa bleekeri. The authors used the combined technology of the Illumina, PacBio, and Hi-C platforms and both DNA and RNA sequencing from 12 tissues for generating 628 Mb highly continuous genome assembly with the scaffold and contig N50 values reaching 22.9 Mb and 3.1 Mb, respectively. More than 596.9 Mb sequences were anchored upon 25 chromosomes. The authors identified 21,198 protein-coding genes, of which 97.3% genes were functionally annotated. The manuscript further characterises the phylogenetic relationships between T. bleekeri and other species with the genus Triplophysa, describes gene family expansion and contraction in the T. bleekeri genome, identifies genes potentially affected by positive selection by estimating the ratios of nonsynonymous to synonymous substitutions and estimates population divergence among three populations based on short-read sequencing data of 28 individuals.

Overall, the assembly and annotation of the genome is well-described and current manuscript is one of several similar works on fish species living on Tibetan plateau. In contrast to genome assembly and annotations, some additional analysis parts of the manuscript, such as population genetic analysis of 28 are rather brief and do not add considerably to the current manuscript. Hence, it would have been interesting if the authors would put more emphasis here, for example by using demographic modelling (e.g. using fastsimcoal2 or other software). Also, it would be interesting if the authors would carry out PSMC analysis to further shed light on temporal changes of effective population size in T. bleekeri which would be very relevant in the context of Qinghai-Tibetan Plateau uplift. My main criticism of the manuscript relates to language and grammar. Clearly, the language of the manuscript needs serious editing. In the detailed comments/suggestions, I have pinpointed some, but not all, of the most obvious cases. I suggest that the authors will use competent native-speaker language assistance to improve the language throughout the paper. In addition, several conclusions that are based on weak reasoning should be removed or softened (see below).

Detailed comments/suggestions

Lines 25-26: Consider rewording, "These environmental changes induced strong stresses for several organisms,.."

Lines 37-38: Replace "underwent strong natural positive selections..." with "have experienced positive selection".

Line 39: replace "samples from populations" to "samples from three populations".

Line 41: Replace "can be attributed to the disrupted gene exchanges among those populations" to alternative description that gene flow is restricted between populations. Based on the data from 3

locations, it is not possible to identify if these changes have been caused by man made changes (dam) or are occurring naturally.
Lines 51-53: Consider rewording "The endemic species of the QTP present a great adaptability to the harsh environments of low temperature and low oxygen". It is not clear what does "a great adaptability" means in this context. Also, if the land rise have increased the river slopes at QTP, it is possible that the oxygen levels in the water are actually reduced. Do you have data on oxygen concentration in QTP rivers to demonstrate that high altitude causes low concentration of O2 in water? I think this is actually quite important, as the high-altutude adaptations in fish because of low oxygen maybe not so widespread at all (you introduce the theme later in the intro).
Lines 79-80: Consider rewording "environmental adaptation mechanism of Triplophysa species is far from fully understood".
Line 90-109: This section need substantial rewriting as it contains numerous language issues. E.g. Line 90-109: Replace "was" to "is"; Line 92: Remove "just", replace foods to food, is still crucial to is crucial, larva to larvae etc.
Line 115: How were the individuals caught?
Line 120: Did you anesthetize fish or use overdosage of MS22 to euthanize fish?
Line 126: Replace "to obtain a comprehensive transcript" with "to comprehensively characterize transcriptome".
Lines 128-129: Did you use any anesthetic? Did you use samples from multiple individuals as indicated in text i.e. "muscle tissues were collected"
Lines 183-184: Change T. bleekeri to italics.
Line 192: Change wording "from the above fish"
Lines 243-245: Remove the Latin names in brackets
Line 261 Remove dot.
Line 276-277: Confusing sentence, consider rewording "the natural selected genes" to " "genes putatively influenced by positive natural selection".
Line 282: Change to "Evolutionary relationships"
Line 292: Replace "genetic structures" to "genetic divergence among T. bleekeri populations"
Line 300: The minimum depth seems very low (3) which suggests that heterozygote genotypes are not called correctly.
Lines 305-306: Replace "was performed to test the topology robust" to " was performed to test the robustness of the tree topology"
Line 309: Change "transcriptome" to "transcriptomic"
Line 350: Change to "transcriptome data from 12 tissues was generated".
Lines 359-360: Consider rewording: "Meanwhile, the important function of the non-coding genes received further attention."
Line 361: Replace "non-coding gene loci" to "non-coding DNA sequences"
Line 366: no need to repeat two times significantly
Both Supplementary Table S4 and S5 indcate gene family expansions in the heading. Should one of the table represent gene family contractions? With Rich factor, do you mean enrichment factor?
Supplementary Table S4. In the first row, both the gene number and background gene number is one and Q value is 0?

Line 381: As this type of analysis relies heavily on what species were included to the analysis, please describe here what species were used.
Line 407: Please change "obtained according to serious filtering criteria". It is not clear what "serious" filtering means here.. Based on the relaxed coverage, actually the filtering seems to be very relaxed? Also, did you checked for Hardy-Weinberg equilibrium and was that included as a filtering parameter?
Lines 411-414: IN the population genetics, the term sister group is not frequently used.Also, what do you mean with monophyly here? I suggest removing this term.
Line 448: Try to avoid superlatives like "was enormously influenced by uplifts"
Line 451: Again, it is not clear to me if the oxygen concentrations in the water have actually deceased in the fast flowing rivers. One can argue that eutrophication likely creates much more frequently decrease of O2 in aquatic environments.
Lines 452-454: This is very confusing and contradictory, you state here that in QTP rivers the oxygen concentrations are high! (which supports my earlier point)
Lines 458-459: This study does not really study "the mechanisms by which Triplophysa species adapt to low temperatures". Consider rewording.
Lines 466-475: The description of the links between low temperatures, ROS and apoptosis is quite speculative and not easy to follow.
Line 462: Based on supplementary tables and results section, there were also many other gene families that were enriched?
Lines 484-485: This is highly speculative. Instead, common ancestry seems to be more parsimonious explanation.
Line 488: Again, please try to avoid superlatives. Not sure what "massive genes" means. Also not sure what "uniquely naturally selected" means.
Line 493: Consider removing "sister group".
Line 508: Reword "hypoxia environment". The question is if the habitat of T. bleekeri really has low concentrations of dissolved oxygen.
Lines 509-510: Consider rewording "It is fascinating to investigate the molecular mechanisms for their environmental adaptation to the QTP" as this sentence does not fit conclusions section.
Lines 520-521: Consider softening the claim as the evidence to link genomic features and environmental parameters is weak at best.
Lines 521-523: Consider rewording. This claim can be states basically for every freshwater fish without any genetic data.
Lines 528-529: Consider rewording high land fish"

Level of Interest

Please indicate how interesting you found the manuscript: Choose an item.

Quality of Written English

Please indicate the quality of language in the manuscript: Choose an item.

Declaration of Competing Interests

Please complete a declaration of competing interests, considering the following questions:

- Have you in the past five years received reimbursements, fees, funding, or salary from an organisation that may in any way gain or lose financially from the publication of this manuscript, either now or in the future?
- Do you hold any stocks or shares in an organisation that may in any way gain or lose financially from the publication of this manuscript, either now or in the future?
- Do you hold or are you currently applying for any patents relating to the content of the manuscript?
- Have you received reimbursements, fees, funding, or salary from an organization that holds or has applied for patents relating to the content of the manuscript?
- Do you have any other financial competing interests?
- Do you have any non-financial competing interests in relation to this paper?

If you can answer no to all of the above, write 'I declare that I have no competing interests' below. If your reply is yes to any, please give details below.

I declare that I have no competing interests

I agree to the open peer review policy of the journal. I understand that my name will be included on my report to the authors and, if the manuscript is accepted for publication, my named report including any attachments I upload will be posted on the website along with the authors' responses. I agree for my report to be made available under an Open Access Creative Commons CC-BY license (http://creativecommons.org/licenses/by/4.0/). I understand that any comments which I do not wish to be included in my named report can be included as confidential comments to the editors, which will not be published.

Choose an item.

To further support our reviewers, we have joined with Publons, where you can gain additional credit to further highlight your hard work (see: https://publons.com/journal/530/gigascience). On publication of this paper, your review will be automatically added to Publons, you can then choose whether or not to claim your Publons credit. I understand this statement.

Yes Choose an item.
