# Supplementary material for: Chromosomal genome of Triplophysa bleekeri provides insights into its evolution and environmental adaptation
Source: Gigascience. 2020 Nov 24;9(11):giaa132. doi: 10.1093/gigascience/giaa132 (PMC7684707; doi:10.1093/gigascience/giaa132)
Supplement: giaa132_Reviewer_1_Report_Revision_1 — Anti VasemÃ¤gi -- 10/8/2020 Reviewed [file giaa132_reviewer_1_report_revision_1.pdf]

Reviewer Report

Title: Chromosomal genome of Triplophysa bleekeri provides insights into its evolution and environmental adaptation

Version: Revision 1 **Date:** 10/8/2020

Reviewer name: Anti VasemÃ¤gi

Reviewer Comments to Author:

Compared to previous version, the authors have considerably improved both the content, presentation and readability of the manuscript. However, there are still issues that should be solved. For example, the discussion of the top 1% signals of the selection among 3 populations and specific candidate genes sounds extremely subjective and speculative. (see below). Hence I suggest to delete these parts in the discussion. In addition, there are still some issues with the language (see some suggestions for correction below, line numbers correspond to the version with changes visible).
Line 266: Consider replacing â€žOnly genes with completed sequences.." with â€žOnly genes with complete sequences...".
Line 300: Consider replacing â€ž...milii and Danio rerio (497-450 Ma) as calibration points for the divergence time..." with â€ž...milii and Danio rerio (497-450 Ma) were used as calibration points for the divergen"ce time..."
Line 302: Consider replacing â€žTo investigate the evolutionary relationship of species in Triplophysa genus.." with â€žTo investigate the evolutionary relationships within genus Triplophysa...."
Line 446-447: Add references to following claim and replace â€žThe non-coding genes have received increased attention in the recent year, since accumulating evidence suggests that many of them play crucial roles in a variety of biological process." With "The non-coding genes have received increased attention in the recent years, since accumulating evidence suggests that many of them play crucial roles in a variety of biological process.
Line 530: Do you mean: "We used the whole-genome short-read sequencing data based on the sample used for genome assembly to obtain the genome-wide genotype data."
Lines 534-537: I am not sure if I understood it correctly. If the authors used gene regions to estimate mutation rate between T. bleekeri and D. rerio and applied this estimate to analyse SNP data from the whole genome, it is certainly an underestimate for whole genome as gene regions are under functional constraint.
Lines 576: Consider replacing: "To investigate the possible natural selection among populations" with " To identify putative signals of differential selection among populations".
Lines 731-734: Pinpointing sÃ¼ecific selection agents as gorge or human activities is hghly speculative given that only 3 populations were studied. I suggest to remove this part.
Lines 736-752: Give that the selection threshold for identifying potential footprints of selection was rather relaxed (the empirical top 1% of the distribution) and not based on any simulations to identify the likelihood of being under selection, I think the discussion on individual selection targets is way too speculative and selective (why the authors selected to discuss these few genes out of all other 1%

genes?). Hence, I suggest to remove it.
I declare that I have no competing interests

Level of Interest

Please indicate how interesting you found the manuscript: Choose an item.

Quality of Written English

Please indicate the quality of language in the manuscript: Choose an item.

Declaration of Competing Interests

Please complete a declaration of competing interests, considering the following questions:

- Have you in the past five years received reimbursements, fees, funding, or salary from an organisation that may in any way gain or lose financially from the publication of this manuscript, either now or in the future?
- Do you hold any stocks or shares in an organisation that may in any way gain or lose financially from the publication of this manuscript, either now or in the future?
- Do you hold or are you currently applying for any patents relating to the content of the manuscript?
- Have you received reimbursements, fees, funding, or salary from an organization that holds or has applied for patents relating to the content of the manuscript?
- Do you have any other financial competing interests?
- Do you have any non-financial competing interests in relation to this paper?

If you can answer no to all of the above, write 'I declare that I have no competing interests' below. If your reply is yes to any, please give details below.

I declare that I have no competing interests

I agree to the open peer review policy of the journal. I understand that my name will be included on my report to the authors and, if the manuscript is accepted for publication, my named report including any attachments I upload will be posted on the website along with the authors' responses. I agree for my report to be made available under an Open Access Creative Commons CC-BY license (http://creativecommons.org/licenses/by/4.0/). I understand that any comments which I do not wish to be included in my named report can be included as confidential comments to the editors, which will not be published.

Choose an item.

To further support our reviewers, we have joined with Publons, where you can gain additional credit to further highlight your hard work (see: https://publons.com/journal/530/gigascience). On publication of this paper, your review will be automatically added to Publons, you can then choose whether or not to claim your Publons credit. I understand this statement.

Yes Choose an item.
