# Supplementary material for: Chromosomal genome of Triplophysa bleekeri provides insights into its evolution and environmental adaptation
Source: Gigascience. 2020 Nov 24;9(11):giaa132. doi: 10.1093/gigascience/giaa132 (PMC7684707; doi:10.1093/gigascience/giaa132)
Supplement: giaa132_Reviewer_2_Report_Original_Submission — Alan Le Moan -- 6/17/2020 Reviewed [file giaa132_reviewer_2_report_original_submission.pdf]

Reviewer Report

Title: Chromosomal genome of Triplophysa bleekeri provides insights into its evolution and environmental adaptation

Version: Original Submission **Date:** 6/17/2020

Reviewer name: Alan Le Moan

Reviewer Comments to Author:

The paper by D. Yuan and collaborator entitled "Chromosomal genome of Triplophysa bleekeri provides insights into its evolution and environmental adaptation" is a "genome" paper aiming to published a good reference genome for the species T. bleekeri. It also provides a description of potential adaptation to altitude/temperature in Tibetan plateau.

I think that the main aims of producing a good genome for the species is achieved by this paper. However, I find the analyses rather descriptive and therefore the paper is a bit heavy to read. In addition, the gene with potential signal of positive selection should be discuss with more caution see https://royalsocietypublishing.org/doi/full/10.1098/rsbl.2018.0055 that describe some bias of the method use in this paper, but many other paper like this exist)

As the introduction rely a lot on the comparison of different Triplophysa species; I was hoping more comparative discussion in between the different species of Triplophysa that is not really done at the moment, or at least in a confusing way.

In addition, I think that the population genomics analyses could be pushed further. At the moment, the 28 WGS are basically not used in the current paper. I think that classical pop. genomics analyses such as detection of selective sweep, Fst outlier scan and visual variation of statistic such as pi or Fst between pair of populations across T. bleekeri genome would help to frame a nice storytelling to describe the adaptation to the altitude. They would be obvious comparison to do with their other selection test based on dN/dS ratio.

However, this would need some substantial modification of the paper. If producing a genome is still enough to publish a paper nowadays, then, you don't necessarily need to include this change. However, I feel that including better pop. genomic analysis would make the paper more interesting.

Some sentences are also a little bit wordy sometime. I suggested some changes below but I might not be the best candidate for this as English is not my native language.

line 26 : why especially fish ? this is not clear to me. Because they are ectotherm? Maybe this is not really interesting here, every organism are constrain in one way or another by their environment.

line 82: what is a "typical" species of a given family? maybe remove this.

Line 84: this sentence doesn't flow correctly we the one above. Considered changing "although" by something else.

line 86: need a ref here

line 92-> 96= this is wordy, rephrase.

Suggestion: The reproduction biology of Trilophysa fishes is also different, while T. tibetana and T. siluroides spawns once a year (from XX to XX and XX to XX, respectively), T. bleekeri can spawn twice a

year with peak breeding seasons occurring from October to December and March to April (Wang et al., 2013)
line 100: why is this "crucial"? I would like to see the specific aims of the study mentioned in here before summarizing the results.
line 108-109 crucial again. This sentence could be removed as it doesn't provide any relevant information. In my opinion, we should always let reader make their mind about what is "crucial" or not…
line 128: reword sacrificed: suggestion: To understand the population structures of the species, a total of 28 individuals were collected from three sampling sites, including eleven, seven, and six individuals from populations 1, 2 and 3, respectively (Fig. 2).
line 174: precise that this is a software
I think that line 179-180 repeat line 174-175
line 300: need more explanation about the filtration step. You should not do filtration for LD but for physical linkage. Lots of information are found in the LD information. Was LD performed overall? A depth of 3 is also really low
I would like to see a PCA in your main paper and more analyses showing the variation of classical pop. gen. statistic such as Fst and pi along the genome.
line 444: Nothing is "primitive" when species are still living nowadays, refers as "outgroup", "most divergence species" or something else.
line 448 : double space. You could also rephrase this sentence
line 494:498 this should be back up by value of average Fst
line 503: this is preliminary but you have the data to explore this further. I don't understand why this is not done. At the moment, the population genetic analyses add really little to no information to your current paper.

Level of Interest

Please indicate how interesting you found the manuscript: Choose an item.

Quality of Written English

Please indicate the quality of language in the manuscript: Choose an item.

Declaration of Competing Interests

Please complete a declaration of competing interests, considering the following questions:

- Have you in the past five years received reimbursements, fees, funding, or salary from an organisation that may in any way gain or lose financially from the publication of this manuscript, either now or in the future?
- Do you hold any stocks or shares in an organisation that may in any way gain or lose financially from the publication of this manuscript, either now or in the future?

- Do you hold or are you currently applying for any patents relating to the content of the manuscript?
- Have you received reimbursements, fees, funding, or salary from an organization that holds or has applied for patents relating to the content of the manuscript?
- Do you have any other financial competing interests?
- Do you have any non-financial competing interests in relation to this paper?

If you can answer no to all of the above, write 'I declare that I have no competing interests' below. If your reply is yes to any, please give details below.

I declare that I have no competing interests

I agree to the open peer review policy of the journal. I understand that my name will be included on my report to the authors and, if the manuscript is accepted for publication, my named report including any attachments I upload will be posted on the website along with the authors' responses. I agree for my report to be made available under an Open Access Creative Commons CC-BY license (http://creativecommons.org/licenses/by/4.0/). I understand that any comments which I do not wish to be included in my named report can be included as confidential comments to the editors, which will not be published.

Choose an item.

To further support our reviewers, we have joined with Publons, where you can gain additional credit to further highlight your hard work (see: https://publons.com/journal/530/gigascience). On publication of this paper, your review will be automatically added to Publons, you can then choose whether or not to claim your Publons credit. I understand this statement.

Yes Choose an item.
