# Supplementary material for: Chromosomal genome of Triplophysa bleekeri provides insights into its evolution and environmental adaptation
Source: Gigascience. 2020 Nov 24;9(11):giaa132. doi: 10.1093/gigascience/giaa132 (PMC7684707; doi:10.1093/gigascience/giaa132)
Supplement: giaa132_Reviewer_3_Report_Original_Submission — Paolo Franchini -- 6/18/2020 Reviewed [file giaa132_reviewer_3_report_original_submission.pdf]

Reviewer Report

Title: Chromosomal genome of Triplophysa bleekeri provides insights into its evolution and environmental adaptation

Version: Original Submission **Date:** 6/18/2020

Reviewer name: Paolo Franchini

Reviewer Comments to Author:

In this manuscript Yuan and colleagues report the chromosome-level genome assembly of the fish species Triplophysa bleekeri and carried out several comparative genomics analyses in order to investigate the genomic mechanisms that facilitated its adaptation to high-altitude environment. To date, two other chromosome-level genome assemblies are available for the Triplophysa genus, but I found the addition of the T. bleekeri genome a valuable resource considering its wider altitudinal distribution range.
The authors used a state-of-the-art "recipe" to obtain chromosome-level assemblies by relying on the use of PacBio long reads, followed by Illumina short reads quality correction and Hi-C scaffolding technique. Notably, very high depth of coverage was used for the three sequencing types applied, and it allowed Yuan et al. to obtain very impressive statistics (e.g. contigs N50 = 3.1 Mb, scaffold N50 = 22.9 Mb).
The authors then predicted protein-coding and non-coding genes in the newly generated assembly and they included these data in a phylogenetic framework. Among the main findings, they identified several genes under positive selection in T. bleekeri (many of them also positively selected in the other two species of the same genus) and a bunch of expanded and contracted gene families with functions potentially related to the adaptation to high-altitude environment. Finally, they re-sequenced 28 T. bleekeri individuals representative of three different populations to provide preliminary data on the population structure of this species. In general, the methods used are sound.
That said, I see room for improvement. The manuscript currently is plagued by some disorganization in presented materials and suffers from some clarity issues in analysis descriptions. Additionally, I feel that the manuscript is not particularly well written and I find very hard to follow some sections. Many sentences would benefit from being re-written and in general all the manuscript should be proofread before to be resubmitted.
Major comments:
- The methods used are robust for the many analyses the authors carried out. However, I think the results could be presented in a better way by at least re-organizing the order of appearance of the different sections, including more information and refining the main figures. The Results section starts with the genome assembly, but I would then describe ortholog detection immediately followed by the phylogenetic analysis. It would help the reader to contextualize their species with the others included in the phylogeny. I would then go with positive selected genes and only after them describe the expansion/contraction of gene families.
- I am not sure I see the value of the re-sequencing analysis. How the three populations were

selected? Do they live in different environments? I would have chosen three populations living at different altitudes (T. bleekeri has a wide distribution ranging from 200 to 3,000 m), as it would have helped, for example, to identify genomic regions of higher divergence in comparison to the background (is it the case?). This in turn would have helped to identify candidate regions potentially harboring genes responsible for adaptation to different high-altitude environment. These candidate genes could have then been compared to the ones identified with the positive selection analyses using the multi-species approach. To sum up, the rationale behind populations selection needs to be better described, and this dataset better explored (e.g. PCA and Admixture are OK, but I would like to see some basic population genetic metrics like e.g. Fst, and Fst values can be shown using Manhattan plot-like style for each pairwise comparison). Finally, I am not sure the tree of Fig. 3 (quite poor graphically) is the best way to report these results. I would suggest the author to produce a more comprehensive new Figure made of 3 panels showing the tree plus the PCA and the Admixture plot (now Suppl. Fig 6 and 7).

- The Discussion is very redundant. Frequently the results are reported exactly as they are reported in the Results section, and only very briefly discussed. I would shorten the Discussion by highlighting the main points and including more interpretation of the results and real discussion. The Conclusion section, again, is a repetition of what have been said before in the Discussion. I would delete this section and implement the few original sentences in the Discussion.

Minor points:

- L. 27: "Triplophysa bleekeri (T. bleekeri)"

The "(T. bleekeri)" is not necessary here. Use T. bleekeri later in the text in the same section, and maybe re-use the full name the first time you mention it in a new section.

- L. 127.128: 11+7+6=24, but here I read 28 individuals.
- L. 32-34: "Phylogenetic analysis showed that T. bleekeri diverged 38.8 and 28.6 million years ago (Ma) from their common ancestors of Triplophysa siluroides and Triplophysa tibetana, respectively"

This sentence can be removed from the abstract, or simply replaced with something mentioning that you resolved the phylogeny of the genus Triplophysa, without numbers.

- L. 77: change "board" to broad"
- L. 82 and 84-85: see my comment above.
- L. 103: Hi-C is not a platform, it is a method. Please rephrase it.
- L. 115: Full name here.
- L. 126: change "comprehensive transcript" to "comprehensive transcript set" or "comprehensive transcriptome".
- L. 128: See my main comment above. Again, can you use a name for the populations instead of 1, 2 and 3? Both here and in the Figure (now Fig. 6).
- Most of the versions of the program used are missing. Please revise it, choose a style (e.g. v1.2; version 1.2; v 1.2 etc) and be consistent.
- L. 135: Change "Hiseq" to "HiSeq", and check this and other names for consistency along the text.
- L. 137: "Pacific Biosciences of California," "of California" not required.
- L. 183: species name in italics
- L. 190: The database used for the BUSCO analysis should be specified here.
- L. 213: change "de novo" with "ab initio"

- L 226: No details are reported on how you filtered the genes predicted by Maker. I would add the quality filtering step here (genes predicted in repetitive regions were removed? Any overlapping threshold used? Genes with internal stop codons removed? etc).
- L. 243-245: I would use common names for each species here, and scientific full names in parenthesis.
- L. 253: Which model did you use in RaxML?
- L. 257: This is too generic. Which calibration points did you use?
- L. 288-291: I would include more details here. For example, which Trinity transcripts did you use for the ortholog detection analysis? The longest transcript for each gene? The pipeline you used should be better described.
- L. 426: In the abstract the contigs N50 is 3.1, here 3.82. Please use the right value and be consistent. Again, I would report the scaffold N50 here? Talking about chromosomes: is the karyotypic number known for this genus or this species, for example from cytogenetic studies? In case, I think it is worth mentioning and discussing it (karyotypic number from cytogenetic vs. assembled chromosomes).
- L. 444: what "primitive" means? Do you mean "basal"?
- I would combine Fig. 1 and 2 in a single figure.
- Fig. 3: can be moved to supplementary material.
- Fig. 4: Did you carry out the positive selection analysis for each of the three species independently and then check for overlap? If so, I would also include a PAML analysis to identify positive selected genes in the lineage that includes all Triplophysa species.
- I would suggest to make the phylogenetic tree the new Fig 2 and improve its quality. Small images of fishes could make the figure nicer and easier to read. Additionally, the confidence intervals at each node could be shown graphically and not with numbers (very difficult to read and interpret).

Level of Interest

Please indicate how interesting you found the manuscript: Choose an item.

Quality of Written English

Please indicate the quality of language in the manuscript: Choose an item.

Declaration of Competing Interests

Please complete a declaration of competing interests, considering the following questions:

- Have you in the past five years received reimbursements, fees, funding, or salary from an organisation that may in any way gain or lose financially from the publication of this manuscript, either now or in the future?
- Do you hold any stocks or shares in an organisation that may in any way gain or lose financially from the publication of this manuscript, either now or in the future?

- Do you hold or are you currently applying for any patents relating to the content of the manuscript?
- Have you received reimbursements, fees, funding, or salary from an organization that holds or has applied for patents relating to the content of the manuscript?
- Do you have any other financial competing interests?
- Do you have any non-financial competing interests in relation to this paper?

If you can answer no to all of the above, write 'I declare that I have no competing interests' below. If your reply is yes to any, please give details below.

I declare that I have no competing interests

I agree to the open peer review policy of the journal. I understand that my name will be included on my report to the authors and, if the manuscript is accepted for publication, my named report including any attachments I upload will be posted on the website along with the authors' responses. I agree for my report to be made available under an Open Access Creative Commons CC-BY license (http://creativecommons.org/licenses/by/4.0/). I understand that any comments which I do not wish to be included in my named report can be included as confidential comments to the editors, which will not be published.

Choose an item.

To further support our reviewers, we have joined with Publons, where you can gain additional credit to further highlight your hard work (see: https://publons.com/journal/530/gigascience). On publication of this paper, your review will be automatically added to Publons, you can then choose whether or not to claim your Publons credit. I understand this statement.

Yes Choose an item.
